# 20th Congress of the European Burns Association (EBA)

**DOI:** 10.3390/ebj4030030

**Published:** 2023-09-04

**Authors:** Nadia Depetris, Alette De Jong, Clemens Schiestl, Franck Duteille, Jill Meirte, Joan P. Barret-Nerin, Paul Van Zuijlen, Jyrki Vuola, Stian Almeland

**Affiliations:** 1 Burn Center, CTO Hospital, Città Della Salute e Della Scienza, 10126 Turin, Italy; 2 Burn Center, Rode Kruis Ziekenhuis, 1942 LE Beverwijk, The Netherlands; aeedejong@rkz.nl; 3 Department of Surgery, University Children’s Hospital, Zurich, 8032 Zürich, Switzerland; clemens.schiestl@kispi.uzh.ch; 4 Department of Plastic, Reconstructive and Burn Surgery, University Hospital of Nantes, 44000 Nantes, France; pr.duteille@gmail.com; 5 Department of Rehabilitation Sciences and Physiotherapy, REVAKI-MOVANT, Faculty of Medicine and Health Sciences, University of Antwerp, 2610 Wilrijk, Belgium; jill.meirte@uantwerpen.be; 6 Oscare, Organisation for Burns, Scar After-Care and Research, 2170 Antwerp, Belgium; 7 Department of Plastic Surgery and Burns, Hospital, Universitari Vall d’Hebron Vall d’Hebron Barcelona Hospital Campus, Passeig Vall d’Hebron 119-129, 08035 Barcelona, Spain; 8 Department of Plastic Reconstructive and Hand Surgery, Movement Sciences (AMS) Institute, Amsterdam UMC, Location VUmc, 1081 HZ Amsterdam, The Netherlands; paulvanzuijlen@me.com; 9 Burn Center and Department of Plastic and Reconstructive Surgery, Red Cross Hospital, 1942 LE Beverwijk, The Netherlands; 10 Association of Dutch Burn Centers (ADBC), 1941 AJ Beverwijk, The Netherlands; 11 Paediatric Surgical Center, Emma Children’s Hospital, Amsterdam UMC, 1105 AZ Amsterdam, The Netherlands; 12 Helsinki Burn Centre, Department of Plastic Surgery, Jorvi Hospital, Helsinki University Hospital, University of Helsinki, Karvasmäentie 8, 02740 Espoo, Finland; jyrki.vuola@helsinki.fi; 13 Department of Plastic, Hand and Reconstructive Surgery, Norwegian National Burn Center, Haukeland University Hospital, NO-5021 Bergen, Norway; stian.almeland@uib.no; 14 Department of Clinical Medicine, University of Bergen, NO-5021 Bergen, Norway

**Keywords:** burns, burn care, burn center

## Abstract

Abstracts of the plenary and special interest sessions, workshops, and oral and poster presentations of the 20th EBA Congress in Nantes, France from 6–9 September 2023.

## 1. Introduction

With a profound sense of accomplishment and pride, we present the official conference report of the 20th European Burns Association (EBA) Congress. This comprehensive report looks into the diverse sessions, interactive workshops, and special meetings the 20th EBA Congress provides.

In Nantes, EBA joins the Société Francophone de Brûlologie for a combined congress, reflecting that mutual support and collective actions are the keys to improving care. Only a durable and cohesive cooperation of different burn societies may allow burn care professionals to have a stronger voice in advocating for policy changes at both national and international levels, increasing funding and public awareness about burn injuries. This advocacy can lead to better resources, improved facilities, and enhanced support systems for burn patients and their families, facilitating rapid and coordinated response in the face of large-scale disasters.

Moreover, the 20th EBA Congress provides a unique multidisciplinary platform where burn care experts and researchers from Europe and beyond may share insights, exchange ideas, and collaborate on advancing the science and practice of burn treatment and recovery.

The whole program of the EBA Nantes Congress has a significant “fil rouge”: “Life beyond survival,” recognizing that the healing of burns extends beyond physical wounds. It encompasses emotional and psychological well-being, social integration, and a renewed sense of purpose. By providing comprehensive care that addresses these aspects, healthcare professionals and support networks can contribute to the complete recovery of burn survivors.

Finally, with great anticipation and honor, we announce the distinguished speaker for this year’s Rudy Hermans Lecture: Mr. Peter Moortgat. His profound dedication to burns and scars has left an indelible mark, and inspires all who aspire to make a difference in the field of burn care.

## 2. Acknowledgments

Thanks are due to all the EBA Committees cooperating for this meeting. The assistance of all the staff members of Congress Care, GL Events, and the Editorial Office of the European Burns Journal in preparing this congress is gratefully recognized. All the industries and companies which supported this congress are also appreciatively acknowledged. Moreover, deep gratitude must be shown to all the professionals, researchers, burn survivors’ associations, and family members who actively and incessantly work to improve burn care.

## 3. Plenary and Special Interest Sessions

**PS.001**
**Wednesday 6 September 10 am–12:30 pm, Auditorium 800**
**Interactive Educational Course: Wounds, a clinical cases discussion**
Gregoire Bondu (France), Franck Duteille (France), Jill Meirte (Belgium)

Reviewing clinical cases together, we will explore the assessment and examination of various types of wounds and the principles of wound management and treatment. Moreover, special attention will be dedicated to the possible complications, their preventions, and the different factors affecting the clinical and healing process. A multidisciplinary approach will ensure our patients the best possible treatment.

**PS.002**
**Wednesday 6 September 2 pm–3:30 pm, Auditorium 800**
**We all are standing on the shoulders of giants—History of Burns**
Clemens Schiestl (CH), Naiem Moiemen (UK), Paul Van Zuijlen (The Netherlands), Nadia Depetris (Italy), Sophie Böttcher (CH)

Modern burn care was built not only upon scientific discoveries and changes in techniques, but also upon the passion and dedication of great women and men that came before us. By sharing the key points and portraits of the personalities who made the history of burn care, we will discover their inspiring messages for today, and the future of burn care.

**PS.003**
**Wednesday 6 September 4 pm–6 pm, Auditorium 800**
**Organization and Leadership of the Burn Center**
Stian Almeland (Norway), Jyrki Vuola (Finland) Peter Dziewulski (UK) Thomas Leclerc (France) Sophia Papadopoulou-Athina Lavrentieva (Greece) Folke Sjöberg (Sweden) David Harrington (US)

The overall aim of a burn center is to provide comprehensive, high-level, and specialized burn care to patients. However, this common aim might be met with a variety of organizational models and structures, reflecting the high degree of variation in country or region-specific factors, such as the structure of the healthcare system, economics, availability and structure of staffing, education, and the specific needs of the population being served. The speakers will reflect on the specific organizational challenges they have met, the solutions created within their specific setting, and the potential generalizability of their experiences. By summarizing their experiences and lessons learned, important core elements for the successful organization of burn care might be extracted.

**PS.004**
**Thursday 7 September 8 am–9 am, Room 200**
**Coffee with the expert: Is this scar scary?**
Paul van Zuijlen, Beverwijk and Amsterdam, The Netherlands

The choice among the different management options for a burn scar depends on the specific characteristics of the scar, the patient’s preferences and goals, and the expert clinicians ‘assessments. Paul van Zuijlen will discuss the different elements to evaluate while deciding the best treatment for a burn scar, presenting interactive clinical cases, and using a multidisciplinary approach.

**PS.005**
**Thursday 7 September 9 am–10:30 am, Auditorium 800**
**Burn Care at a Global Level**
Clemens Schiestl (CH), Folke Sjöberg (Sweden), Laura Pompermaier (Sweden), Christian Stoppe (Germany), Alette de Jong (the Netherlands), Dominique Potokar (France), Habib Rahim (Afghanistan)

Improving global burn care is crucial due to the worldwide high impact of burn injuries in terms of mortality, disability, and for mitigating economic burdens. International societies may play an essential role by addressing different ranges of intervention, from research to education, enhancement of medical management, and promoting international cooperation among burn centers.

**PS.006**
**Thursday 7 September 11 am–12:30 pm, Room 300**
**Burn Out in the Burn Team**
Jill Meirte (Belgium), Alette de Jong (the Netherlands), Heather Lynn Rogers (Spain), Margot van Mol (the Netherlands), Helma Hofland (the Netherlands)

Burn care professionals face challenging ethical situations, and experience high patient mortality rates and demanding workloads on a daily basis. All these factors may lead to excessive stress and eventually burnout syndrome, which severely affects professionalism, the quality of care delivery, efficiency, and overall quality of life. Therefore, it is critical to identify, prevent and mitigate these work-related risk factors to protect the mental health and wellbeing of the whole burn team.

**PS.007**
**Thursday 7 September 11 am–12:30 pm, Auditorium 800**
**Best Wishes from the Petri Dishes: Cell therapy in Massive Burns, History, Current Practice, and Future Perspectives**
Naiem Moyemen (UK), Jyrki Vuola (Finland), Clemens Schiestl (CH), Esther Middelkoop (the Netherlands), Sophie Böttcher (CH), Sol Ruiz (Spain)

Cell therapy for burns is a promising research field involving the use of stem cells or other types of cells to regenerate damaged skin tissue and promote healing in burn victims. Several clinical trials and research projects exploring the use of cell therapy for burns are being developed in Europe. Moreover, some clinical applications are already in place, and may become available for European patients. In this session, we will learn about the clinical opportunities and protocols for burn patients all over Europe. What are the guidelines for cell therapies in burns at the moment? Are they available in Europe? How can we obtain them for our patients?

**PS.008**
**Thursday 7 September 2 pm–4 pm, Auditorium 800**
**Skin Substitutes, Present and Future Perspectives**
Rocio G. Valencia (CH), Miguel Alaminos (Spain), H. Ibrahim Korkmaz (The Netherlands),
Marina Trouillas (France), Céline Auxenfans (France), Anna Pignet (Austria), Clemens Schiestl (CH), Gloria Carmona (Spain), Sophie Böttcher (CH)

Whether allogenic or autologous, skin substitutes are a successful way to regenerate damaged skin tissue and promote healing in burn victims. In this session, we will learn about skin substitutes that are currently used in the clinic, as well as research products focused on improving the quality of current treatments.

**PS.009**
**Thursday 7 September 4:30 pm–6 pm, Auditorium 800**
**Patient and Family-Centered Care**
Jill Meirte (Belgium), Anna Schildt (Finland), Christelle Jung (CH), Jonathan Bayuo (China), Christine Rosch (CH), Lina Sophie Toft Lernevall (Norway)

Patient and family-centered care (PFCC) is an approach to healthcare that prioritizes patients’ and their families’ needs and preferences. This approach recognizes that patients and their families are the most crucial healthcare team members and should be actively involved in all aspects of care. PFCC has ben shown to improve patient satisfaction and health outcomes, and to reduce medical errors. In this session, we will learn how this approach may be integrated in the care of burn patients.

**PS.010**
**Friday 8 September 10:20 am–12 pm, Auditorium 800**
**Resilience: what are the key factors?**
Fredrik Huss (Sweden), Paul Van Zuijlen (the Netherlands), Amira Allahham (Australia), Titus and Diane Radstake (the Netherlands), Marta Allue (Spain)

Adapting and recovering are essential to healing after a burn injury and regaining control and purpose in life. In this session, we will discuss and better understand the key factors to address in order to help burn survivors develop resilience and achieve a better quality of life after their injury.

**PS.011**
**Friday 8 September 1:30 pm–3 pm, Auditorium 800**
**Formula One (Grand-Prix of Burn Aftercare)**
Mark Fisher (US), Peter Moortgat (Belgium), Paul van Zuijlen (The Netherlands)

Helping burn patients reach their goals of reconstruction and reintegration requires expert teams working in smooth coordination, much like an F1 pit crew. Importantly, social, emotional, financial, developmental, and physical considerations must all be taken into account when developing the plan. In this session, small multidisciplinary teams will compete in a rapid succession of cases to develop an analysis of the patient and a patient-centered plan for aftercare. Please join us for a fun and friendly competition to see who will take home top honors.

**PS.012**
**Friday 8 September 3:30 pm–6 pm, Auditorium 800**
**Functional and Aesthetic Reconstruction of the Face: from grafts to transplants and beyond**
Franck Duteille (France), Andrew Lindford (Finland), Julien Verdier (France), Delphine Vouilliaume (France), Jean Michel Rives (France), Juan Barret (Spain), Andrew Lindford (Finland), Thomas Leclerc (France), Arthur Caplan (US)

The functional and aesthetic reconstruction of the face is a complex and multifaceted field involving various techniques and approaches, from autologous tissue graft and allograft reconstructive surgery to face transplantation and the possibilities offered by new technologies. Multiple options are now available to enhance a person’s appearance and restore their facial form and function. Short and long-term sequelae and ethical and psychological issues should be considered when choosing the best option for the patient.

**PS.013**
**Saturday 9 September 7:30 am–8:30 am, Room 200**
**Debride or not debride: when and how?**
Jyrki Vuola (Finland)

The debridement of a burn wound can help promote healing and prevent infection. Whether or not debridement is necessary and the specific method used depends on various factors, such as the type of wound, its depth, the extent of tissue damage, and the patient’s overall condition. Jyrki Vuola will discuss the elements used to evaluate a case while deciding the best treatment for a burn wound, presenting interactive clinical cases and using a multidisciplinary approach.

**PS.014**
**Saturday 9 September 8:30 am–10 am, Auditorium 800**
**Ethical Dilemmas in Burn Care**
Laura Pompermaier (Sweden), Frank Siemers (Germany), Thomas Leclerc (France), Folke Sjöberg (Sweden), George Agich (US)

Ethical dilemmas can arise in various aspects of burn care, involving decisions related to treatment, resource allocation, patient autonomy, and end-of-life care.

Ethical dilemmas in burn care can be highly complex and context-dependent. They require input from all the multidisciplinary team members, involving healthcare providers, bioethicists, psychologists, and social workers, among others, to navigate these challenging situations while upholding ethical principles and providing the best possible care for patients.

**PS.015**
**Saturday 9 September 10:30 am–12 pm, Auditorium 800**
**Working Together: Interprofessional Communication is the key to Success in Burn Care**
Jill Meirte (Belgium), Sophie Böttcher (CH), Teresa Tredoux (UK), Folke Sjöberg (Sweden), Peter Dziewulski (UK), Frank Faulhaber (CH), Yvonne Kröger (CH), Elvira Lang (US), David Harrington (US)

Effective interprofessional communication is essential to success in burn care. By working together, healthcare professionals can provide patients with the comprehensive care they need to recover from burn injuries and achieve the best possible outcomes.

We all want the best outcomes for our patients, and this can be achieved by sharing information, exchanging knowledge and experience, and collaborating on patient care plans.
What is the best way to communicate?

## 4. Meetings

**M.001 Wednesday 6 September 12:30–13:30, Room 200**
**Research Committee Meeting: Presentation and Discussion of Research Committee activities**

The primary objective of the Research Committee is to design, plan and support multidisciplinary research activities contributing to Burn Care advancements. All the professionals interested in supporting and conducting research in burn care are invited to the meeting on Wednesday 6th of September, to discover and discuss the present and future activities of the Research Committee, and to join it. If you are interested in joining the Research Committee and contributing to its activities, get in touch with Clemens Schiestl. Write an email introducing yourself and expressing your interest directly to clemens.schiestl@kispi.uzh.ch.

**M.002 Thursday 7 September 1 pm–2 pm, Room 200**
**PAM Committee Meeting: Presentation and Discussion of PAM Committee activities**
Stefania Simone (CH), Sigrid Brokke (Norway), Anna Schildt (Finland), Dominique Potokar (France), Lottie Armitage (UK), Christelle Jung (CH), Jill Meirte (Belgium)

The PAM (Professionals Allied to Medicine) Committee covers a wide professional group of non-physicians associated with burn care (Nurses, Physiotherapists, Occupational Therapists, Social Workers, Psychologists, Dieticians, and other professionals). 

All PAM professionals are invited to the meeting on Thursday 7th of September to discover and discuss the past and future activities of the PAM Committee, and to join it, to cooperate in better and more comprehensive European Burn Care.

Moreover, during the PAM Committee meeting, new members will be elected. If you are interested in joining the PAM Committee and contributing to its activities, get in touch with Jill Meirte. Write an email introducing yourself and expressing your interest directly to jill.meirte@uantwerpen.be.

**M.003 Thursday 7 September 1 pm–2 pm, Room 150**
**VICToRY Trial meeting**
Christian Stoppe (Germany), Lee Cancio (US)

The VICToRY trial is a multicenter international randomized trial designed to investigate the role of vitamin C in the early phases of burn injury. Gain a first insight into this fundamental ongoing multicenter study and join the open investigators’ meeting during the EBA on Thursday between 1–2, Room 150. Christian Stoppe and Lee Cancio will review the background rationale for using vitamin C in managing burns and the status of the VICToRY study to date, explaining all its key methodological and operational issues. 

The VICToRY trial is still actively recruiting sites!

If you are interested in joining the VICToRY trial, contact Christian Stoppe. Write an email introducing yourself and expressing your interest directly to christian.stoppe@gmail.com.

**M.004 Thursday 7 September 2 pm–4 pm, Room D**
**Burn Dietitians meeting**
Frederiek Bosch (The Netherlands), Josefin Dimander (Sweden), Nienke Slikker (The Netherlands), Marjolijn Lantman-Gommers (The Netherlands), Yvonne Verweij-Tilleman (The Netherlands), Helena Kneppers (The Netherlands)

All dietitians involved in burn care may participate in this meeting to discuss up-to-date information regarding nutritional therapy in burn patients. Moreover, new cooperation activities among European burn dietitians will be presented and discussed. If you are interested in getting in contact with the group and contributing to its activities, get in touch with Frederiek Bosch. Write an email introducing yourself and expressing your interest directly to f.g.bosch@pl.hanze.nl.

**M.005 Friday 8 September 12:30 pm–13:30 pm Room 200**
**Prevention Committee meeting: Presentation and Discussion of Prevention Committee activities**
Mamta Shah (UK), Daniela Oumard (Germany), Laetitia Goffinet (France)
Koen Maertens (Belgium)

The Prevention Committee aims to prevent burn injuries in Europe and beyond.

All burn care professionals, educationalists, and community workers wishing to promote burns prevention are invited to the meeting on Friday 8 September. 

Come to the meeting to discover the present and future activities of the EBA Prevention Committee, and start your cooperation. If you are interested in joining the Prevention Committee and contributing to its activities, get in touch with Mamta Shah. Write an email introducing yourself and expressing your interest directly to mamta.shah@manchester.ac.uk.

## 5. Workshops

**W001**
**Wednesday 6 September 4 pm–6 pm**
**Thursday 7 September 4 pm–6 pm**
**Comfort Talk in Burn Care**
Elvira Lang (US)

Burn injuries are not only physically painful but also emotionally and psychologically challenging for patients. By adopting the principles of comfort talk, participants will learn techniques to alleviate patient distress, promote relaxation, and support the healing process.

**W002**
**Thursday 7 September 9 am–10:30 am**
**Friday 8 September 1:30 pm–3 pm**
**Hands-on treating wounds and scars from a multidisciplinary perspective**
Stefania Simone (CH), Sigrid Brokke (Norway), Anna Schildt (Finland), Dominique Potokar (France), Lottie Armitage (UK), Christelle Jung (CH), Jill Meirte (Belgium)

This practically oriented workshop promotes an interdisciplinary exchange among all disciplines, and encourages all participants to present different approaches to (1) early compression therapy, (2) different dressing fixation techniques in challenging body locations, and (3) a special focus on the challenges involved in treating the face. We start with a theoretical introduction followed by different learning skill stations to practice the skills hands-on, and to learn from each other and with each other. The workshop will include practicing these techniques and skills on each other.

**W003**
**Friday 8 September 8 am–9:50 am**
**How to harvest a split-thickness skin graft—history, practice, hands-on, tips and tricks**
Clemens Schiestl (CH), Naiem Moiemen (UK), Jyrki Vuola (Finland), Habib Rahman Qasim (Afghanistan)

The success of a split-thickness skin graft (STSG) depends on several factors, including proper patient and site selection, surgical technique, postoperative care, close monitoring, and follow-up. 

During this workshop, you will have the opportunity to learn how to ensure optimal outcomes, starting with theoretical basics, before turning over to practical examples and discussing tips and tricks from an international and multidisciplinary faculty. 

## 6. Oral Presentations

**Wednesday 6 September 4–6 pm**
**Session: Nursing**
**O1.4.1**
**A Toolkit for Creating a Therapeutic Relationship between Patients after Self-Immolation and Burns Nurses**
Lisa Schoone and Alette de Jong
Rode Kruis Ziekenhuis, Beverwijk, The Netherlands
**Aim**

Self-immolation in patients suffering psychiatric disorders is one reason for admission to the burn center. Usually, non-psychiatric trained nurses care for these patients in psychiatric crises and with severe burns. For burns nurses, creating a therapeutic relationship is not natural, due to a lack of expertise and training. This limits optimal care for these complex patients, since this relationship is important for contact and cooperation, wherein patients can express feelings, and nurses can control their situation. The aim of this study is therefore to provide insight into how nurses can establish therapeutic relationships with patients after self-immolation.

**Method**

We used a design-oriented study to investigate how nurses can build a therapeutic relationship. Through a literature review, we searched for effective interventions. Additionally, four experts in the field of psychiatric nursing were interviewed to determine effective and appropriate approaches to creating therapeutic relationships.

**Results**

According to the literature and the interviews, three themes for the development of a good therapeutic relationship emerged: (1) creating a beneficial climate by investing in contact; (2) supervising in order to bear the emotional impact; and (3) increasing knowledge via continuous learning.

**Conclusions**

These themes are connected to practical instructions in a toolkit designed specifically for burns nurses, helping to shape therapeutic relationships from the first day of admission. It includes instructions on investing in contact, on paying attention to the emotional impact on nurses, and training elements to increase knowledge in psychiatry. This toolkit will be added to the electronic patient record in order to integrate it into daily care.

**O1.4.2**
**Effects of a Nurse-Led Aftercare Telehealth Program for Adult Burn Survivors: A Pilot Randomized Controlled Trial**
Jonathan Bayuo, Frances Kam Yuet Wong
The Hong Kong Polytechnic University, Hung Hom, Hong Kong
**Aim**

To examine the effects of a newly developed aftercare telehealth program on quality of life, sleep, pain, itchiness, scarring, psychological and physical role functioning for adult burn survivors in Lanzhou, China.

**Methods**

A prospective, two-arm, pilot randomized controlled approach was employed. A total of 60 adult burn survivors aged ≥18 years were randomly allocated to either the control or treatment group, with 30 participants per arm. Participants in the intervention group received an 8-week aftercare support program coordinated by burn care nurse case managers. The program comprised two phases: a pre-discharge phase, and a follow-up phase delivered via the WeChat app following discharge. Quality of life was the primary outcome, and secondary outcomes included sleep, pain, itchiness, scarring, and psychological and physical role functioning. All outcomes were evaluated at three time points: T0 (baseline), T1 (immediately post-intervention at 8 weeks), and T2 (4 weeks from T1). A generalized estimating equation was employed to ascertain group, time, and interaction effects.

**Results**

Statistically significant higher scores regarding quality of life (*p* = 0.015), HADS-Depression (*p* = 0.013), HADS-Anxiety (*p* = 0.023), total HADS (*p* = 0.009), and physical role functioning (*p* = 0.041) were observed at T1 and T2. No statistically significant findings were observed regarding sleep, scarring, and itchiness.

**Conclusions**

Aftercare support is central to the post-burn recovery process, and it is possible to deliver this support via a locally available telehealth platform. The mean scores, however, highlight a need to extend the program in the long-term, considering the chronicity associated with the post-burn recovery process.

**O1.4.3**
**The Standard Process and Safe Care of Burned Patients at University Hospital Vall d’Hebron**
**Elena Villanueva Montero,** Pol Miguel Puigbarraca, Laura González Gilarte, Marta Aguayo-Álvarez, Rocio Tabernero Gallego
Ics, Barcelona, Spain
**Aim**

To improve the patient care process at the burns unit of Vall d’Hebron to achieve high-quality care and provide adequate clinical safety for patients during their healthcare process.

**Methods**

A team of 24 burn unit professionals from different categories was created. They carried out a 33 h analysis to elucidate the different circuits present the burn center. The analysis was carried out under the supervision of a process manager expert nurse, and following the lean methodology. In addition, adult and pediatric patients explained their experiences; these were analyzed in order to ascertain their point of view. Six different circuits were detected within the burn patient care process: emergency, critical, surgical block, hospitalization, outpatient care, and rehabilitation. Finally, different work lines were identified, some actions were prioritized, and their implementation started.

**Results**

A total of 146 lines of improvement were detected, 46 of which were prioritized and planned; safety was one of them. Different professionals within the unit handled the following items: awareness and set-up of alarms, quick answers to patient calls, security rounds and encouraging notifications of near misses, no-harm incidents and harmful incidents. The study found that safety culture has developed.

**Conclusions**

Following a standardized methodology for analyzing the process that burned patients go through has allowed the prioritization and implementation of improvement lines. All the workers’ involvement in establishing good care practices acts to consolidate the development of safety culture and strengthen new work dynamics. As a consequence, the communication between the staff has increased, and so has the patient’s quality of experience.

**O1.4.4**
**The use of immersive virtual reality for pain and anxiety control in burned patients**
**Pol Miguel Puigbarraca,** Elena Villanueva Montero, Laura González Gilarte, Mireia González López, Maria José Sánchez Garcia
ICS, Barcelona, Spain
**Aim**

To reduce pain and anticipatory anxiety levels in adult and pediatric admitted patients at Vall d’Hebron Hospital burns units during dressing changes, using immersive virtual reality sessions.

**Methods**

We started a study to assess patient satisfaction after the use of virtual reality for wound care. We evaluated levels of anxiety and pain prior, during, and after the dressing change. Nursing staff performed sessions of immersive virtual reality of different durations with the use of virtual reality glasses with adult and pediatric patients during routine cures, cures for staple removal, and complex cures.

**Results**

With the use of virtual reality, cleansing wounds and changing burn dressings was less irritating and anxiety-inducing, as reported by the patients. This results in a decrease in the use of painkillers and anxiolytics prior to dressing changes.

**Conclusions**

The use of virtual reality glasses as a non-pharmacological measure in pediatric and adult patients seems to be useful for pain and anxiety control in routine care. It is necessary, however, to continue with data collection to complete the study and to be able to analyze the results in order to create a new protocol for using this technique.

**O1.4.5**
**Training Nurses to Improve Burn Care in Africa**
**Mrs Ziphilly Chiumia**
Queen Elizabeth Central Hospital, Blantyre, Malawi
**Abstract**
**Introduction**

Burn injuries in Malawi contribute greatly to morbidity and mortality (Samuel J.C et al.). One of the major factors in these burn injuries is poor management (Kasenda S. et al.), which is one result of a lack of trained nurses in burn care. There is an urgent need to train nurses to promote and disseminate quality care for burn patients. There is an existing approach that succeeds in improving the quality of burn nursing in low-resource settings, and creating leaders to disseminate this approach, as outlined below.

Personal inter-burns timeline

2017—Participant in Advanced Burn Care (Nursing)

2019–2020—Participant in Implementation Science for Nurses: A Quality Improvement Project

2019—Essential Burn Care (EBC) Trainer of Trainers (ToT)

2019/2022—Faculty member ABC (Nursing) Ethiopia and EBC Mwanza, Tanzania

2023—Course Lead, ABC (Nursing) Dar es Salaam

**Benefits and Achievements**

This timeline, from participant to course lead, resulted in increased knowledge and skills in burn care; for instance, use of the aseptic technique in wound dressing and improved burn nutrition both led to a reduction in burn mortality from 26% to less than 10% in our unit. The acquired knowledge and skills increased staff’s level of confidence in the execution of patient care and leadership.

**Challenges**

The frequent rotation of burn-trained nurses results in the loss of valuable resources within the burn unit, and compromises the quality of patient care.

**The Way Forward**

To train more nurses in East Africa, and build up complete training faculty in burn care in order to enhance care.

**O1.4.6**
**The Reliability and Validity of a Frailty Assessment Tool in Specialized Burn Care: a Retrospective Multicenter Cohort Study**
**Charlotte I. Cords** ^1,2^, Margriet E. van Baar ^1,3^, Marianne K. Nieuwenhuis ^4,5,6^, Anouk Pijpe ^7,8,9^, Cornelis H. van der Vlies ^1,2^
^1. ^ Association of Dutch Burn Centers, Maasstad Hospital, Rotterdam, The Netherlands
^2. ^ Trauma Research Unit Department of Surgery, Erasmus MC, Rotterdam, The Netherlands
^3. ^ Department of Public Health, Erasmus MC, Rotterdam, The Netherlands
^4. ^ Association of Dutch Burn Centers, Martini Hospital, Groningen, The Netherlands,
^5. ^ Hanze University of Applied Sciences, Research Group Healthy Ageing, Allied Health Care and Nursing, Groningen, The Netherlands
^6. ^ University of Groningen, University Medical Center Groningen, Department of Human Movement Sciences, Groningen, The Netherlands
^7. ^ Association of Dutch Burn Centers, Red Cross Hospital, Beverwijk, The Netherlands
^8. ^ Amsterdam UMC location Vrije Universiteit Amsterdam, Plastic, Reconstructive and Hand Surgery, De Boelelaan 1117, Amsterdam, The Netherlands
^9. ^ Amsterdam Movement Sciences, Tissue Function and Regeneration, Amsterdam, The Netherlands
**Aim**

To assess the Canadian Study of Health and Aging Clinical Frailty Scale (CFS) inter-rater reliability and validity (predictive validity, known group validity, and convergent validity) in patients with burn injuries in specialized burn care.

**Methods**

A retrospective multicenter cohort study was conducted in specialized burn care. Patients aged ≥50 with burn injuries and a primary admission in 2015–2018 were included. A research team member retrospectively scored the CFS. Inter-rater reliability (IRR) was calculated using Krippendorff’s α. Validity was assessed using a logistic regression analysis. Patients with a CFS ≥ 5 were considered frail.

**Results**

In total, 540 patients were included. The mean age was 65.8 years, and mean TBSA burned was 8.5%. The mean CFS was 3.4 (SD 2.0). According to the CFS, 28% of all patients were frail. The CFS’s IRR was adequate (Krippendorff’s α = 0.69, 95% CI 0.62–0.74, n = 212). Positive CFS was predictive of adverse outcomes, including non-home discharge (OR = 3.57, 95% CI 2.16–5.93), higher in-hospital mortality, and mortality < 12 months post-discharge (OR = 3.05 95% CI 1.06–8.77 and OR = 4.61, 95% CI 1.99–10.65), after adjustment for age, TBSA, and inhalation injury. Frail patients were more likely to be older (OR = 2.88, 95% CI 1.95–4.25) and have more severe comorbidities (OR = 6.43, 95% CI 4.26–9.70). The CFS was significantly related (rSpearman = 0.55) to the Dutch Safety Management System frailty screening, indicating a fair-to-good correlation between the two scales.

**Conclusions**

The CFS is reliable and has shown its validity, including its association with adverse outcomes in patients in specialized burn care. The CFS could be considered for the early recognition and treatment of frailty.

**O1.4.7**
**Introduction of a Academic Online UK Regional Advanced Burns Module**
Nicole Lee, Niall Martin
Chelsea and Westminster, London, UK
**Aim**

The introduction of a regional online academic Advanced Burns Module would improve and standardize education across a region, enabling improved access to a burns academic module.

**Methods**

In partnership with University of East Anglia, an online Advanced Burns Module was released, regionally, in May 2022 to the London and South East Burns Network MDT. The course was designed around the journey of patient from admission through to discharge and follow up, with 6 online study days taught by MDT professionals from across the region. Course competencies, weekly MCQ tests, and a student service improvement project were agreed assessment criteria.

**Results**

The pilot group from May 2022 received great feedback, and a second module in September 2023 saw the introduction of an overseas student. The third module in January 2023 saw students from outside the region and another overseas student. Over the first academic year, 36 students completed the module. Overall feedback was positive; junior staff enjoyed the mix of course leads and MDT teaching from across different services. The development of podcasts, case studies, and increasing up-to-date content continues new research and student feedback.

**Conclusions**

Bringing regional, national and international students on to the same module opens up more in-depth discussions and learning in relation to the care of our burn survivors. Running online modules reduces costs, allowing educational training budgets to send more students into the workforce, thereby increasing the overall burns-trained workforce. The continued development of modules and the introduction of military students are the next steps in the development of a sustainable module.

**O1.4.8**
**The Effect of Simulation-Based Training on Caregivers of Burn Patients’ Preparedness of Care and Caregiving Burden**
Sabri Karahan 1, Zahide Tuncbilek 2
^1 ^ Harran University, Sanlıurfa, Türkiye,
^2 ^ Hacettepe University, Ankara, Türkiye
**Aim**

This research was carried out to determine the effect of the discharge training given using the scenario-based simulation method on the care readiness and caregiving burden of the home caregivers of burn patients.

Method: The study was conducted as a randomized controlled study. The sample of the study consisted of caregivers of 60 burn patients. In the study, the participants were assigned to the groups using the block randomization method. In the implementation of the study, standard discharge training was given to the caregivers in the control group, and a booklet was given before discharge. Simulation-based training was given to the simulation group after the standard training. The Preparedness for Caregiving Scale (PCS) was applied to all participants before and after the training. One month and three months after discharge, the Caregiving Burden Scale (CBS) was administered. 

Results: There was no significant difference between the two groups in terms of socio-demographic data. While there was no statistically significant difference in terms of the PCS scores of the caregivers before the training, it was found that the mean score of the simulation group after the training was statistically significantly higher (*p* < 0.05). The CBS score was higher in the control group in the first and third months after discharge than in the simulation group (*p* < 0.05). 

Conclusions: It has been determined that simulation-based discharge training is more effective than standard training in preparing caregivers for care and reducing the burden of caregiving. It is recommended that simulation-based training be used in discharge training.

**O1.4.9**
**The Importance of the Checklist for Patient Safety**
Nicoletta Cederle, Elena Romani, Martina Bussola
Azienda Azienda Ospedaliera Universitaria Integrata Di Verona, Verona, Italy
**Aim**

Constant innovation and development in modern medicine is making it more difficult for professionals to carry out and memorize all the steps involved in their work. We hypothesized that the introduction of the checklist as a simple and reliable tool could help to avoid errors and improve inter-professional exchange.

**Methods**

Enzymatic debridement was introduced to burns units in 2017. After initial training and organizational change, we established a checklist for an enzymatic debridement procedure. To provide better information for whole team, we divided it into four parts: medical prescription, consent to treatment and dosage, patient preparation, and application and product removal. On the basis of first year experience, we realized that it was mandatory to select a team leader for every step of the procedure. This is a novelty that has strongly influenced interdisciplinarity and interprofessionality in our team. The allocation of different work steps between numerous specialties and disciplines has supported various approaches and agreements, thereby permitting the transfer of responsibility. All that lead to better team cohesion. In 2022, as a result of staff turnover, there was an adjustment of the same through a training course in the field, and revision of the AIFA information notes.

**Results**

From February 2017, we have managed 127 adult patients from 18 to 88 years, with a percentage of TBSA from 7 to 65%. All checklists have been compiled in real time and no critical issues have emerged.

**Conclusions**

Creation of the checklist has allowed us to avoid mistakes and reduce risks, ensuring the safety of the burned patient.

**Wednesday 6 September 4–6 pm**
**Session: Critical Care, Anesthesia and Nutrition**
**O1.5.1**
**The value of Intravascular Volume Measurement by Transthoracic Echocardiography in Fluid Resuscitation of Children with Major Burns**
**Sabri Demir** ^1^, Secil Sayin ^2^, Selin Cayhan ^1^, İbrahim Ece ^2^, Emrah Senel ^3^

^1^  University of Health Sciences, Ankara Bilkent City Hospital, Children Hospital, Department of Pediatric Surgery, Pediatric Burn Center, Ankara, Turkiye,
^2^  University of Health Sciences, Ankara Bilkent City Hospital, Department of Pediatric Cardiology, Ankara, Türkiye,
^3^  Ankara Yildirim Beyazit University, School of Medicine, Department of Pediatric Surgery, Ankara, Türkiye

Aim: Transthoracic echocardiography (TTE) is a non-invasive and reliable diagnostic tool for evaluating hemodynamic parameters related to myocardial dysfunction and intravascular volume status. Our study aims to determine the predictive value of TTE in managing fluid resuscitation in children with major burns.

Methods: Forty patients (TBSA > 20%) were included in the study. They were divided into two groups (n = 20): TTE (TG) and a control group (CG). Initial fluid resuscitation was started according to the Galveston formula. Follow-up in CG was performed according to vital signs, body weight, and urine output. In addition, in TG, cardiac functions and the diameter of the inferior vena cava (IVC Min-max), IVC collapsibility index (IVCCI), the descending aorta (Ao) diameter, and the ratio of IVC/Ao were evaluated. Additional fluid was given to patients with an IVC/Ao ratio < 0.8, while fluid was decreased in patients with an IVC/Ao ratio > 1.2. It was not changed in patients with a normal IVC/Ao (0.8–1.2).

Results: While the IVC/Ao ratio was low in eight (40%) patients in TG, it was high in three patients (15%). During the follow-up, 10% of the TG and 60% of the CG needed diuretic treatment (*p* < 0.05). No difference was found between the mortality rates of both groups.

**Conclusions**

Vital signs and urine output may not always reflect the actual volume status of patients with major burns. Our study showed that IVC/Ao ratio could effectively evaluate intravascular volume in burn patients for whom fluid resuscitation is critical. As a non-invasive and safe tool, TTE could be routinely used in fluid management in burn patients.

**O1.5.2**
**Inhalation Injury—Is There Always an Indication for Treatment in an Intensive Care Unit? The Use of Scintigraphy: A Modern Method for Diagnosing Inhalation Injuries**
**Przemysław Strzelec**, Piotr Wróblewski, Karolina Ziółkowska, Mariusz Trzaska, Michał Kalemba
Dr. Stanisław Sakiel Center for Burn Treatment In Siemianowice Śląskie, Siemianowice Śląskie, Poland

Aim: Thermal injuries may be a concomitant or isolated injuries to both the upper and lower airways. In such cases, the question always arises as to whether a patient should be intubated, and if so, when?

Methods: A review of the literature on inhalation injuries does not exhaustively answer this question. There are no unambiguous procedures, and individual centers or countries have their own procedures for the management of confirmed or suspected inhalation injuries. 

Results: The vast majority of studies have proven that early intubation of a burnt patient is in many cases unnecessarily performed, and the risk of complications associated with mechanical ventilation increases significantly. One of the most effective methods of assessing the presence of an inhalation injury is still bronchoscopy, which in the case of lung parenchyma burns cannot assess their extent, and the number of centers wherein it is possible to diagnose this type of inhalation injury with the use of scintigraphy is very limited.

**Conclusions**

Depending on the area of injury, the depth of the burn and the presence of symptoms of respiratory failure, it is recommended to intubate or monitor the patient and adopt a waiting attitude while implementing bronchodilation, anti-edematous treatment, oxygen therapy, or the use of hyperbaric oxygen. Each patient with a suspected thermal injury or due to the location and depth of the burn is at risk of respiratory failure, and should be consulted by an anesthesiologist.

**O1.5.3**
**Microsurgical Reconstruction of Burned Patients at the Traumatology Hospital “Dr. Victorio De La Fuente Narváez” IMSS, Mexico**
**Daniel Ponce Franco**, CPR David Paralta, CPR Raúl Granados Martínez
Hospital de Traumatología y Ortopedia “Dr. Victorio De La Fuente Narvaez” IMSS, Ciudad De México, Mexico

Aim: To describe the microsurgical reconstruction techniques performed in the last 9 years in a specialized burns unit in Mexico.

Methods: This is a retrospective and descriptive study of cases of microsurgical reconstruction performed in the last 9 years in the burns unit of the Traumatology Hospital “Dr. Victorio de la Fuente Narváez”, and presentation of the results of each technique performed.

Results: A total of 15 microsurgical flaps were performed for the reconstruction of wounds due to burns, as well as the correction of scar sequelae, of which a survival rate of 86.6% is reported. The diversity of microsurgical techniques used and the different anatomical areas reconstructed are presented below:

Three anterolateral thigh (ALT) flaps for a retractable neck scar;

One lateral brachial flap for the middle third of the face;

One radial flap for a bloody area in the skull;

One ALT flap for skull wound;

One radial flap for penile reconstruction;

Two ALT flaps for foot reconstruction;

Two medial plantar flaps for hand reconstruction;

One latissimus dorsi flap for the reconstruction of a skull wound;

Two ALT flaps for hand reconstruction;

One SIEA flap for a retractable neck scar.

The preoperative and postoperative photographic comparisons of the reconstructed patients will also be presented.

**Conclusions**

Reconstruction of special areas in burn patients is one of the most complex challenges facing the plastic surgeon today. Microsurgical techniques are currently still extraordinary tools for this purpose, and in specialized centers the success rate is quite reasonable.

**O1.5.4**

**The Effects of Burn-Specific Venous Thromboembolism (VTE) Prophylaxis Guideline on Outcomes and the Peak Anti Factor Xa Levels of Patients with Burns >20% TBSA**
**Dane Holden** ^1,2^, Yvonne Singer ^1^, Hadley Bortz ^1^, Sharon Selanayakam ^1^
^1 ^ Alfred Health, Melbourne, Australia,
^2 ^ Monash University, Melbourne, Australia
**Aims:**

1. To monitor the incidence of VTE events and the subsequent complications of being anti-coagulated;
2. To assess adherence with the severe burns VTE prophylaxis guideline;
3. To monitor patients anti-factor Xa (AFXa) in patients > 100 gs or BMI > 30, to quantify the therapeutic adequacy of VTE prophylaxis.

**Methods:**

- This is a prospective observational cohort study.
- All patients who were admitted with severe burns >/=20% TBSA to the Victorian adult burns service between January 2022 and January 2024 will be eligible for inclusion in this study. Some 40–60 patients will be included in this study.
- The efficacy of this prophylaxis guideline will be evaluated by prospectively recording rates of VTE events. Complications arising from enoxaparin administration will also be collected, including bleeding/haematoma development requiring an intervention, and adverse drug reactions.
- Anti-factor Xa (AFXa) levels

To quantify the therapeutic adequacy of the LMWH VTE prophylaxis guideline for all patients who weigh >/=100 kgs or have a BMI >/=30 kg/m^2^, laboratory monitoring of patients’ AFXa activity will be measured after three or four doses of enoxaparin, 4–6 h after dose administration (the target range is 0.2–0.5 IU/mL). The number of dose adjustments required to reach therapeutic adequacy will be recorded.

**Results:**

Pending.

**Conclusions:**

Pending.

**O1.5.5**
**Predicting Blood Loss in Burn Excisional Surgery**
**Rolf Gigengack** ^1,2^, Diman Taha ^3^, Martijn Kuijper ^4^, Seppe S.H.A. Koopman ^3^, Cornelis H. van der Vlies ^5^
^1 ^ Burn Center, Maasstad Hospital, Rotterdam, The Netherlands
^2 ^ Departments of Intensive care and Anesthesiology, Amsterdam UMC, Amsterdam, The Netherlands
^3 ^ Departement of Anesthesiology, Maasstad Hospital, Rotterdam, The Netherlands
^4 ^ Maasstad Academy, Maasstad Hospital, Rotterdam, The Netherlands
^5 ^ Departments of Trauma and Burn surgery, Maasstad Hospital, Rotterdam, The Netherlands
**Aim:**

Blood loss during burn excisional surgery remains an important factor, as it is associated with significant comorbidity, mortality and a longer length of stay. The goal of this study is to investigate blood loss and develop a prediction model to identify patients at risk of blood loss.

**Methods:**

This retrospective study included adult patients undergoing burn excisional surgery of ≤10% body surface area (2013–2018). Duplicates, missing data, and delayed surgeries were excluded. The primary outcome was blood loss. A prediction model for per-operative blood loss (>250 mL) was built using a multivariable logistic regression analysis with stepwise backward elimination. Discriminative ability was assessed by the area under the ROC curve in conjunction with optimism and calibration.

**Results:**

269 patients were included. Median blood loss was 50 mL (0–150)/% body surface area (BSA) and 0.28 (0–0.81) mL/cm^2^ excised. Blood loss of >250 mL was present in 39% of patients. The model can predict blood loss > 250 mL based on %BSA excised, length of surgery and ASA-score, with an AUC of 0.922 (95% CI 0.883–0.949) and an AUC after optimism correction of 0.915. The calibration curve showed an intercept of 0.0 (95% CI −0.36–0.36) with a slope of 1.0 (95% CI 0.78–1.22).

**Conclusions:**

The median blood loss during burn excisional surgery is 50 mL/% BSA excised. However, a substantial proportion of patients are at risk for higher blood loss. The model can be used to identify patients at risk of significant blood loss (>250 mL).

**O1.5.6**
**Comparison of Analog Methods versus a PorTable 2D Application for Calculating Burned Body Surface: A Retrospective Study**
**Juan Bosco Ruiz-Padilla** ^1^, Héctor Xavier Pantoja Gómez ^2^
^1 ^ Plastic Surgery & Burn Unit, SMA, MX,
^2 ^ Centro Estatal de Cuidados Crìticos, Salamanca, Mx
**Introduction:**

Accurately estimating the burned body surface is critical in the management of burned patients, as an overestimation of the burned area can lead to overhydration, which increases morbidity and mortality. Currently, analog methods are commonly used for estimating the burned body surface, but they are subjective, dependent on the observer’s experience, and particularly inaccurate in pediatric and overweight patients.

**Objective:**

This study aimed to demonstrate that a porTable 2D application is more accurate in calculating the burned body surface than analog methods.

**Methods:**

This retrospective study from the years 2018–2019 reviewed the records of 150 burned patients from two specialized institutions. Only patients with complete records including burned body surface spreadsheets and the method used for estimation were included in the study. The results obtained through the E-Burn application were compared with those obtained analogically during the pre-operative exploration.

**Results:**

The study revealed an overestimation ranging from 5% to 60% in patients whose burned body surface was initially calculated analogically, compared to those calculated with the E-Burn application (*p* < 0.05, Student’s t analysis).

**Conclusions:**

Accurately estimating the burned body surface is crucial in ensuring proper hydration of burned patients. Analog methods are subjective, dependent on observer experience, and particularly inaccurate in pediatric and overweight patients. Therefore, it is recommended to use 2D and 3D devices for estimating the burned body surface, such as the E-Burn application, to improve accuracy and minimize morbidity and mortality in burned patients.

**O1.5.7**
**Documented Nutritional Therapy in Relation to Nutritional Guidelines Post-Burn Injury**
**Josefin Dimander** ^1,2^, Agneta Andersson ^1^, Catarina Lindqvist ^3^, Adriana Miclescu ^1,2^, Fredrik Huss ^1,2^
^1 ^ Uppsala University, Uppsala, Sweden,
^2 ^ Uppsala University Hospital, Uppsala, Sweden,
^3 ^ Karolinska Institutet, Stockholm, Sweden
**Aim:**

To evaluate documented nutritional therapy in relation to international guidelines for patients after burn injury.

**Methods:**

A retrospective observational study at a burn center in Sweden was performed. The patients enrolled in the study were admitted between 2017–2019, ≥18 years old, and in need of hospital care for ≥72 h post-burn injury. The patients were divided according to total body surface area burnt (TBSA%) into minor burn injuries (TBSA < 20%) and major burn injuries (TBSA ≥ 20%). Documented treatment was compared to 24 nutritional therapy recommendations. The documented nutritional therapy’s degree of adherence with nutritional guidelines was defined as high ≥ 80%, moderate 60–79.9%, or low < 59.9%.

**Results:**

Some 90 patients with minor burn injuries and 44 patients with major burn injuries were included. Documented adherence to the nutritional guidelines was low overall. After minor burn injuries, 8% (2/24) of recommendations showed a high adherence to nutritional therapy guidelines, 17% (4/24) a moderate adherence, and 75% (18/24) a low adherence. Two items were documented as having high adherence in patients after major burn injury. Approximately one-fourth of the items in major burn patients (6/24) had a moderate adherence, and the remaining 67% (16/24) of items were documented as having low adherence.

**Conclusions:**

This study revealed low adherence to nutritional guidelines in patients treated post-burn injury. Given the disparity between guidelines and documented nutritional therapy, there could be a considerable risk of inadequate nutritional therapy post-burn injury.

**O1.5.8**
**Correlation of Body Mass Index with Outcome in Burn Patients**
**Agnieszka Surowiecka**, Tomasz Korzeniowski, Katarzyna Pecka, Kamil Torres, Jerzy Strużyna
East Center of Burns Treatment and Reconstructive Surgery, Krasnystawska 52, Poland
**Aim:**

There is an increasing number of obese patients in burn intensive care units (ICUs), and obesity is becoming an important problem in burn care. Obesity is often related to serious comorbidities that might affect the healing process and may predispose patients to lethal complications. Body Mass Index (BMI) is not a perfect index for measuring obesity and the tissue composition of the body; however, it is easy to evaluate and still recognized as a screening tool. The aim of the study was to verify the correlation of BMI with the clinical parameters of burn patients.

**Method:**

A total of 201 patients admitted to the burn unit of the East Center of Burns Treatment and Reconstructive Surgery between January 2019 and January 2020 were enrolled into the study. The exclusion criteria included age under 18, severe pemphigus, and severe cutaneous adverse reactions, e.g., toxic epidermal necrolysis.

**Results:**

There were 149 patients enrolled into the study. LOS decreased by approximately 0.586 days with an increase in BMI by one unit, *p* = 0.034. With an increase in BMI, we observed a higher HGB upon admission, by a mean level of HGB by 1 unit, (*p* = 0.044). Upon discharge, a higher BMI was correlated with a higher HGB level (*p* = 0.001). The overweight subgroup had a lower level of phosphate difference (phosphates) by 0.7 units, compared to patients with a normal BMI (*p* = 0.006).

**Conclusions:**

This study showed the ‘obesity paradox’, and the protective effect of a higher BMI.

**O1.5.9**
**Factors Associated with Post-Intensive Care Syndrome: A Follow-Up Study in a Military Burn Center**
**Nicolas Donat**, Julie RENNER, Thibault BAUDIC, Thomas LECLERC, Matthieu LAURENT
Burn Center, HIA Percy, Clamart, France
**Aim:**

ICU patients can develop late complications called post-intensive care syndrome (PICS). PICS is poorly characterized in burn ICU (BICU) patients. A 3-month post-BICU consultation can help detect late complications and improve care practice and patient course. We used its observations to identify PICS-associated factors in our BICU.

**Methods:**

After ethical approval, the following data from 3-month post-BICU consultations were retrospectively analyzed: burn severity, management features, early acute stress disorder (ASD); 3-month functional, pain, and mental status (HAD and PCLS scores); and specific complications, and quality of life (SF-12). Correlations were tested with a chi-squared or Pearson’s test as appropriate, and the dependency of continuous variables on factors were tested with linear regression models.

**Results:**

Over 50 months, 51 patients were analyzed, mostly males (N = 31.61%), aged 43 ± 16 years, with ABSI 7 ± 2, median TBSA 20% [IQR 13–32], length of stay 29.6 days [IQR 19–54], and two [IQR 1–3] surgical procedures. Post-traumatic stress disorder was significantly correlated with ASD and acute neuropathic pain. Anxiety and depression (HAD A&D > 11) were, respectively observed in 16 and 8% of patients, with only 28 patients (54%) having no symptoms. Physical and mental quality of life was overall good (45% and 48%, respectively). Only PCLS and HAD-D were independently associated with the SF-12 mental component. One out of three patients required specialist referral and treatment modification.

**Conclusions:**

Despite ASD and neuropathic pain prevention, PICS is frequent after BICU and should be detected. Its mental components prevail (45%), apparently without correlation with burn severity.

**O1.5.10**
**Evaluation of Factors Related to Early Acute Kidney Injury in Patients with Severe Burns Admitted to Burn Intensive Care Units**
**Sheyda Rimaz** ^1^, Siamak Rimaz ^2^, Ali Ashraf ^3^, Mohammadreza Mobayen ^4^, Sajjad Roshanfar ^5^
^1 ^ Burn and Regenerative Medicine Research Center, Guilan University of Medical Sciences, Rasht, Iran,
^2 ^ Anesthesiology Research Center, Department of Anesthesiology, Guilan University of Medical Sciences, Rasht, Iran,
^3 ^ Clinical Research Development Unite of Poursina Hospital, Guilan University of Medical Sciences, Rasht, Iran,
^4 ^ Burn and Regenerative Medicine Research Center, Guilan University of Medical Sciences, Rasht, Iran,
^5 ^ Burn and Regenerative Medicine Research Center, Guilan University of Medical Sciences, Rasht, Iran
**Aim:**

In this study, we aimed to investigate the incidence of early AKI and the factors associated with early AKI in patients with severe burns.

**Methods:**

This retrospective cross-sectional study was performed on burn patients with TBSA ≥ 20% between March 2016 and November 2020. KDIGO criteria were used to define early AKI in the first 5 days of hospitalization. Multivariable logistic regression was used to model association between baseline risk factors and the risk of AKI.

**Results:**

Of the 194 patients included, the mean age of the subjects was 42.99 ± 17.58. Some 138 patients (71.1%) were male. The mean TBSA% was 49.18 ± 24.71. According to KDIGO criteria, 43 patients (22.2%) developed early AKI during the first 5 days of hospitalization as follows: Stage I (12.4%) 24 patients, Stage II (7.2%) 14 patients, Stage III (2.6%) 5 patients. Some 85 patients (43.8%) died. Patients who developed AKI had more age, mechanical ventilation and ICU stay days, sepsis, a higher Baux score, and a modified Baux score and mortality rate when compared to those patients who did not develop AKI (*p* = 0.001). A multivariable logistic regression demonstrated association between AKI and the following variables: gender (OR = 2.872, *p* = 0.032), age (OR = 1.047, *p* = 0.000), and TBSA% > 60% (OR = 6.134, *p* = 0.001), which are independent risk factors for developing early AKI. Our study also showed that TBSA% significantly increases the severity of AKI.

**Conclusions:**

The results of this study showed that early AKI is common in patients with a major burn injury. Age, gender and TBSA% are the strongest independent predictors of early AKI.

**O1.5.11**
**Intubation and Extubation Criteria of Patients with Burn Injuries**
**Eugene Koh**, Elizabeth Concannon, Nicholas Solanki, Marcus Wagstaff, John Greenwood
Royal Adelaide Hospital, Adelaide, Australia
**Aim:**

This study reviewed indications for intubation compared with internationally accepted criteria (ABA/Denver criteria), duration of intubation, and complications arising from the intubation of burn patients treated at the Royal Adelaide Hospital (RAH) burns unit between 2017–2020.

**Methods:**

Burn patients who were intubated on arrival to the RAH or in a pre-hospital setting were identified using the BRANZ database. Indications for intubation were compared to the ABA and Denver criteria. Data pertaining to patient demographics, burn characteristics, and nasendoscopy/bronchoscopy findings were collected.

**Results:**

A total of 62 patients were identified with a mean total body surface area of 17.8%. Some 74–91% of patients met the ABA and Denver criteria. The most common reason for intubation was singed facial hair or extensive facial burns. A total of 58% of patients were intubated pre-transfer, and 61% in the pre-hospital setting. Ventilator-associated pneumonia developed in 22.5% of patients, 93% of whom were intubated for more than 48 h. Moreover, 52% of patients were extubated within 48 h.

**Conclusions:**

Most adult patients with burns admitted to the RAH are intubated as per published criteria. However, over half of the patients were extubated within 48 h, suggesting potentially avoidable intubation. This study supports the sentiment that current intubation criteria may over-estimate the risk of airway compromise. Early nasendoscopy/bronchoscopy may be useful in determining patients who can be safely extubated in less than 48 h.

**Wednesday 6 September 4–6 pm**
**Session: Wounds and Geriatrics**
**O1.6.1**
**How Early is the Early Management of Deep Periorbital Burns?**
**Katya Kalinova**^1^, Ralitsa Raycheva ^2^, Petar Uchikov ^1^

^1^  Medical University, Plovdiv, Bulgaria,
^2^  Medical University, Plovdiv, Bulgaria

**Background:**

Deep periorbital burns are an important issue mainly due to the presence of the eyes in the region, and the crucial importance of preservation of vision. Recently, the early approach to their surgical management has progressively prevailed over the conservative approach. According to the literature, early surgery extends from the 3rd to the 18th day after the trauma, which is a considerably wide range for periorbital burn management. 

The aim of this study was to explore the right timing of early surgery for deep periorbital burns, with a view to the frequency and severity of early and late sequelae.

**Materials and Methods:**

A retrospective analysis of the treatment and outcome of 446 deep periorbital burns hospitalized in the Department of Burns of St George’s University Hospital in Plovdiv, Bulgaria, over 10 years, was conducted.

**Results:**

Deep periorbital burns accounted for 74.8% of hospitalized deep facial burns. Concomitant ocular pathology was diagnosed in 14% (n = 63) of the patients. An early, staged and precise surgical approach was favored. Follow-up time ranged from 3 months to 5 years. Late ocular sequelae occurred in 7.4% (n = 33) of the patients. There was no incidence of secondary corneal perforation or definitive loss of vision.

**Conclusions:**

Timely and adequate treatment during the acute period can minimize initial damage and late sequelae. An early, balanced surgical approach aimed at rapid wound closure between day 2 and 10 post burn is most beneficial. Preservation of vision is a determining factor for the significance of trauma and the effectiveness of treatment.

**O1.6.2**
**The Timing of Surgery in Acute Burn Care: a Dutch Retrospective Repository Study**
**Roos Salemans**^1,2^, Denise van Uden ^1^, Eelke Bosma ^3^, Cornelis H van der Vlies ^2,4^, Dutch Burn Repository group, the National Burn Care, Education & Research group the Netherlands
^1 ^ Association of Dutch Burn Centers, Maasstad Hospital, Rotterdam, the Netherlands,
^2 ^ Trauma Research Unit, Department of Surgery, Erasmus MC, University Medical Center Rotterdam, Rotterdam, the Netherlands,
^3 ^ Department of Trauma and Burn Surgery, Martini Hospital, Groningen, the Netherlands,
^4 ^ Department of Trauma and Burn Surgery, Maasstad Hospital, Rotterdam, the Netherlands

The timing of surgery in acute burn care remains a topic of debate. An analysis of the current state of surgical timing is required.

**Aim:**

The aim of this study is to analyze the timing of burn surgery in three Dutch burn centers in order to provide insights for future treatment decisions and research.

**Methods:**

This retrospective study included adult patients with acute burns who underwent at least one surgical intervention in three Dutch burn centers from 2009 to 2021. Data are derived from the prospective Dutch Burn Repository R3. Factors related to timing of surgery, including patient, injury, and treatment characteristics, are analyzed. Clinical outcomes for early (≤7 days) and delayed (>7 days) surgery groups are described and compared, and trends over time are analyzed.

**Preliminary results:**

The preliminary results from approximately 3400 patients show a median time to first surgical intervention of 14 days. Some 19.8% of patients received their first surgery within 7 days post-burn. Tangential excision with knife appeared to be the most commonly used wound debridement method (65%), with meshed split-skin graft being the most popular transplantation technique (80%). The mean number of operations, length of hospital stay, and mortality will be presented for early and delayed surgery groups. Complete data will be presented at the congress.

**Conclusions:**

The Dutch burn centers have placed focus on delayed surgery. A better understanding of the relationship between surgical timing and outcomes will help in the development of evidence-based treatment strategies for burn patients in the Netherlands and beyond.

**O1.6.3**
**Closing of Mine-Shrapnel Combat Defects of Lower Extremities with Free and Local Perforator Flaps**
Oleh Rudenko ^1^, **Pavlo Badiul** ^1,2^, Sergii Sliesarenko ^1^, Tetyana Buriak ^1^, Kirill Sliesarenko ^1,2^
^1 ^ Burn and Plastic Surgery Center, Dnipro, Ukraine,
^2 ^ Dnipro state medical university, Dnipro, Ukraine
**Aim:**

Analysis of the results of plastic reconstruction of military injuries of cover and soft tissues on the lower extremities using free and local perforator flaps.

**Methods:**

The authors conducted a retrospective review of 139 flaps for 108 patients (103 men and 5 women) with mine-shrapnel and bullet combat injuries treated in the clinic from 2014 to 2022.

**Results:**

In all cases, extensive wound defects were completely closed in one stage, and the patients were discharged with recovery. Non-critical complications occurred in eight cases (5.7%), and additional care or secondary sutures were required. In six cases (4.3%), when we faced critical complications and flaps were lost, we extended plan B, which included the use of another type of perforator flap or skin graft. The treatment time was extended by 27 days.

**Conclusions:**

The authors’ results reflect a high rate of successful reconstruction of military wound defects on the lower extremities using free and local perforator flaps. The presented method demonstrates at the same time the possibility of reconstructing lower limb defects based on the concept of “like for like” replacement, and minimizing morbidity at the donor site, thereby achieving the best possible aesthetic and functional outcome.

In most cases, perforator-free and local flaps allow the primary closure of a large defect in one stage on the thigh, in the area of the knee joint, and on the lower leg, in the absence of secondary defects characteristic of donor sites when choosing alternative techniques.

**O1.6.4**
**Improving Burn Care: Lessons Learned from Treating the First 300 Patients with NexoBrid^®^ Enzymatic Debridement**
**Antonio Bulla**, Jon Ander Aguirrezabala, Danilo Antonio Rivas Nicolls, Jacinto Baena Caparrós, Jordi Serracanta Domènech
Hospital Universitari Vall d’Hebron, Barcelona, Spain, Barcelona, Spain
**Aim:**

The aim of this retrospective study was to evaluate our experience of the first 300 burn patients treated with NexoBrid^®^, a debridement agent based on Bromelain, a concentrate of proteolytic enzymes found in the stem of the pineapple plant.

**Methods:**

The first 300 patients treated with NexoBrid^®^ at Vall d’Hebron Hospital in Barcelona were included in this study.

We collected data on patients’ demographics, burn characteristics, need for surgery, time to healing, complications, and scar outcome. 

The study was authorized by the Institutional Ethics Committee.

**Results:**

Between 2015 and 2021, 229 (76%) men and 71 (24%) women were treated with NexoBrid^®^. The median age was 41 (sd: 16), and the median TBSA burned was 8%. Flame burns caused 46.5% of the burns, deflagration 21.6%, and boiling liquids 18.3%. Upper and lower extremities accounted for 64.6% of all treated areas, and face for 20%. The median treated area was 5%. The man time to complete debridement was 1.2 days (sd:1.07); only 2.66% of patients needed escharotomies. The mean burned area treated was 67%. 59% of patients needed skin grafting. Infections were observed in 10% of patients, mostly by St. Aureus and Ps. Aeruginosa.

**Conclusions:**

Our study confirms NexoBrid^®^ as an effective treatment for burns, especially those caused by flames and localized in limbs and face. It significantly reduces the time to complete debridement, the need for graft surgery, and the need for escharotomies. These benefits can lead to better healing outcomes for burn patients. 

However, the risk of post-NexoBrid^®^ infections should be carefully monitored.

**O1.6.5**
**Confirmation of our helpful hints for treatment decisions after enzymatic debridement with NexoBrid^®^**
**Karel Claes**, Ignace De Decker, Petra De Coninck, Jos Verbelen, Stan Monstrey
Ghent University Hospital, Ghent, Belgium
**Aim:**

Wound bed evaluations after NexoBrid^®^ removal and the corresponding treatment decisions are prone to subjectivity and failure. The purpose of this study was to evaluate whether our helpful hints (clinical wound bed characteristics and laser Doppler imaging (LDI) flux values) were consistent with the treatment decision taken for a more recent population of burn patients (1).

**Methods:**

All patients with one or more mainly LDI-blue regions of interest (ROI, healing potential < 21 d) treated with NexoBrid^®^ (01/12/2019–01/08/2022) were included in this retrospective study.

**Results:**

Thirty-seven patients (mean age 36 y) had a NexoBrid^®^ procedure for their 73 mainly LDI-blue ROI (with a mean surface area of 54.3 cm^2^). After NexoBrid^®^ treatment, 28 ROI (38.3%) could be treated conservatively until complete reepithelialization, while 44 ROI (60.3%) needed autografting. One ROI (1.4%) was treated with a combination of both. Regarding the decision to operate, 4/20 patients (20%) with LDI flux values 145-200PU (LDI-light blue) had surgery, 2 of them had a ‘step-off’; 41/53 patients (77%) with LDI flux values < 145 PU (LDI-dark blue) had surgery, and 32 of them (32/41, 78%) had clinical signs (step-off, visible/translucent fat). The overall mean healing time for all ROI was 40.6 d. Hypertrophic scarring (mean follow-up 395.9 d) was only seen in 12 ROI (16.4%): 8 treated surgically and 4 treated conservatively.

**Conclusions:**

The wound bed characteristics after NexoBrid^®^ removal in combination with the LDI flux values of the ROI are still considered valuable tools for decision making. The experience of the rating physician can also not be underestimated.

(1) Claes et al., Burns, 2022

**O1.6.6**
**Ex Vivo Model of a Skin Burn**
**Anthony de Buys Roessingh**, Anthony Applegate, Wassim Raffoul, Ania Labouchère
CHUV, Lausanne, Switzerland
**Purpose of the Study:**

A burn model is important for testing new treatments. Currently, these models are created mainly on animals. For obvious ethical, anatomical and physiological reasons, these models should eventually disappear. We propose the creation of a burn model on human skin using a pulsed dye laser (PDL).

**Methods:**

The excess abdominal skin of nine women was obtained following an elective breast reconstruction operation. Within one hour after surgery, burns were induced using a PDL on skin samples (595 nm), at different fluences (7 and 13 J/cm^2^), number of pulses (5–54) and durations (3 and 40 ms). A total of 53 burns were performed before being analyzed histologically. Skin samples were graded according to a newly created code; samples were inspected after 14 and 21 days to assess their ability to spontaneously heal and re-epithelialize.

**Results:**

We have determined the parameters of a PDL inducing first-, second- and third-degree burns on human skin. After 21 days, a neo-epidermis was formed.

**Conclusions:**

Our results show that this simple, fast and user-independent process creates reproducible and uniform burns of different, predictable degrees, close to clinical reality. With fixed parameters, first-, second- and third-degree burns were induced. In vivo models of human skin can easily replace animal testing, especially for large-scale screening. This model could be used to encourage experimentation with new treatments on known degrees of burns, and thus to improve therapeutic strategies.

**O1.6.7**
**Treatment of Superficial and Partial-Thickness Facial Burns Using a Nanocellulose Face Mask: First Retrospective Study**
**Jose Joel Casas Beltran**
Mexican Institute of Social Security, Hermosillo, Mexico
**Aim:**

To evaluate clinical aspects (pain, re-epithelialization time, infection rates, long-term scarring and complications) using a nanocellulose face mask (Epicite hydro^®^) treatment for superficial and partial-thickness facial burns

**Materials and Methods:**

Records of patients attending for facial burns and treated with nanocellulose face mask, demographics (age, sex, cause of burn, type of burn, and total body surface area), measures of pain (verbal rating scale 1–10), time of re-epithelialization, and complications were recorded. The VSS (Vancouver scar scale) was applied at 6 months. The exclusion criteria were infection prior to application and full-thickness burns.

**Results:**

Some 115 files of patients with facial burns treated with a nanocellulose face mask were collected. They were 78% male and 22% female; the median age was 29 years (ranging from 1 to 87 years), total body surface area 15% (range 1% to 50%), 94% second-degree burns, 6% third-degree burns (excluded). The cause of burns was 78% fire, 16% scald, 3% chemical, and 3% contact. The pain scale median was 2 (range 1–4), and the mean re-epithelialization time was 8 days (range 5–14 days). No wound complications were registered, including infections or allergies. The score for VSS was 0 (range 0–8).

**Conclusions:**

The nanocellulose face mask (Epicite hydro^®^) has demonstrated it can be safely used on face burns on patients of all ages, and on all type of burns. We have demonstrated that its use reduces pain and re-epithelialization time, and prevents infections. It can help to improve quality of life after a burn injury to the face by preventing hypertrophic scarring, which makes it a great novel treatment for facial burns.

**O1.6.8**
**A Decade Since European Approval of Bromelain-Based Enzymatic Burn Debridement: Lessons Learned**
**Yaron Shoham** ^1^, Adam Singer ^2^, Yuval Krieger ^1^, Eldad Silberstein ^1^, Jeremy Goverman ^3^
^1 ^ Soroka University Medical Center, Beer Sheba, Israel,
^2 ^ Stony Brook University, Stony Brook, New York, USA,
^3 ^ Mass General Hospital, Boston, MA, USA
**Aim:**

The aim of this report is to review the literature published about NexoBrid^®^ (NXB) enzymatic burn debridement since its initial approval in the European Union in December 2012, and provide an overview of the main lessons learned during the past decade.

**Methods:**

We conducted a literature search using the terms “NexoBrid” and “burn enzymatic debridement” in Pubmed and Google Scholar, published during the decade between December 2012 to December 2022.

**Results:**

After excluding industry-based trials, a total of 103 publications were found. The main issues reported in these publications include the learning curve associated with use of NXB; treatment efficacy, including treatment of hands and faces; post-debridement wound bed diagnosis and wound bed coverage; prevention and release of elevated compartment pressure; pain management; safety issues; cost efficacy; consensus guidelines; use during the COVID-19 pandemic; off-label treatment, including use in children and large burns; its potential implications for burn mass casualty events; and literature reviews. We comprehensively review these issues.

**Conclusions:**

During the past decade more than 10,000 patients have been treated with NXB around the world. This constitutes a great deal of knowledge learned, much of which has been shared with the scientific community in over 100 peer-reviewed publications. It appears there is a substantial body of evidence supporting the safety and efficacy of NXB as a valid non-surgical eschar removal agent. We expect the recent approvals in various regions around the world, including the United States, will contribute to the continuing growth of this body of evidence.

**O1.6.9**
**Long-Term Outcomes after Treatment of Deep Dermal to Totally Dermal Burns with a Polylactide-Based Matrix (Supra SDRM^®^) as Dermal Skin Substitute with Two-Sided Split-Skin Coverage**
**Matthias Rapp**, Robert Schappacher, Ulrich Liener
Marienhospital Stuttgart, Stuttgart, Germany
**Aims:**

Previously established dermal skin substitutes are mostly of xenogeneic, animal, or allogeneic origin, with risk of immunologic reaction or disease transmission. The timing for two-stage split-thickness skin coverage with currently known dermal substitutes is 14–32 days. We aimed to investigate the extent to which SupraSDRM^®^ is suitable as dermal substitute.

**Methods:**

The polylactide-based matrix SupraSDRM^®^ for guided wound healing is purely synthetic and hydrolytically resorbable. The bimodal foam–membrane structure promotes increasing cell migration, progressive vascularization, and collagen deposition, resulting in matrix remodeling with the formation of dermal granulation tissue. Lactate degradation promotes angiogenesis and dermis formation, and reduces inflammation and oxidative stress.

**Results:**

In 11 patients with a mean age of 62.7 y, 18% TBSA, and ABSI 8, SupraSDRM^®^ was applied on 22 sites after deep dermal and epifascial necrectomy or decortication on five different wound beds (fat, fascia, muscle, tendon, bone). Split-thickness skin grafts could be performed after a mean time of 14.4 days. So far, treatment courses of 37–579 days, mean 264.7 days, can be considered.

This showed extensive healing of split-thickness skin grafts with good displacement, little-to-no hypertrophic scarring or scar keloids, no significant shrinkage of scars, and good functional, mechanical, and aesthetic results.

**Conclusions:**

Progressive vascularization of SupraSDRM^®^ with increasing cell migration results in matrix remodeling with formation of dermal granulation tissue, which can be covered with split-thickness skin grafts in a two-step procedure. Long-term results show a good displacement of soft tissues, absent or only slight hypertrophic scar formation or scar keloids, as well as good functional, mechanical, and aesthetic results.

**O1.6.10**
**A Novel Skin Grafting Modality That Significantly Boosts Efficiency: Prefabricated Large Graft Sheet of Postage-Stamp Autografts and Allografts to Repair Extensive Deep Burn Wounds**
**Chuanan Shen**, Bohan Zhang, Xinzhu Liu, Huageng Yuan
Chinese People’s Liberation Army General Hospital, Beijing, China
**Aim:**

To introduce a novel grafting modality that makes intermingled transplantation postage-stamp auto- and allografts, an excellent modality per se, but one that has been limited to repairing small residual wounds; it is now feasible to use this modality to repair extensive deep burn wounds.

**Methods:**

Allogeneic skin was cut into small postage-stamp grafts and laid on a sterilized plate with sites for autografts reserved according to the predesigned optimal layout. Then, the patient’s available autologous skin was harvested, cut up, and fitted into the reserved sites. After this, the grafts received a fine spray of biological adhesive before a single-layer absorbent gauze was gently pressed onto it to obtain a large sheet of evenly laid grafts (hereinafter called the prefabricated large sheet) ready for use. A total of 21 operations using this modality were performed on 11 patients with extensive deep burns (86.27 ± 8.82%TBSA; II–IV degree), and the grafting time per unit area (10 cm × 10 cm) was calculated to compare with that of conventional piece-by-piece grafting. Eventually the take rates of the two modalities were compared.

**Results:**

The average time of prefabricated large sheet grafting and piece-by-piece grafting per unit area was (0.41 ± 0.09) min and (7.46 ± 1.07) min, respectively, and the difference was statistically significant (*p <* 0.001). The average take rate of the large sheets was (85.43 ± 6.14)%, and that of the piece-by-piece transplanted grafts was (87.29 ± 5.23)%, and there is no significant difference (*p >* 0.05).

**Conclusions:**

The novel modality carries out the laborious graft-positioning process before operation, significantly reduces the intraoperative time, and makes the intermingled transplantation of small auto- and allografts now feasible for repairing extensive deep burn wounds.

**O1.6.11**
**The Benefits of Using Human Keratinocyte Allografts in Older Burn Patients**
**Gerardo Lujan Alvarez**, Israel De Jesus Silva Saucedo, Claudia Berenice Hernandez Valverde, Jose Maria Zepeda Torres
Plastic Surgery Department, UMAE Dr. Victorio de la Fuente Narvaez, Mexico City, Mexico

An older person is considered to be a person with a lifetime of 60 years and over. Burns in older adults are an important public health problem, due to the susceptibility to complications that these patients present. It is common for them also to suffer from concomitant diseases, malnutrition, and neglect by their relatives, which aggravates their situation as predicted. A serious burn in an older adult is considered to be one that affects 10% or more of their total body surface, involving a depth of the second degree and even more superficial when involving special areas. In the burn unit of the VFN Mexico City trauma hospital, we are carrying out an early management protocol both medical and surgical, and using cultured human keratinocyte allografts to comprehensively treat these patients. Keratinocyte allografts are a culture of human cells taken from the foreskins of newborns and developed with tissue engineering. Allografts were used in the affected areas with a depth of the second superficial degree, in mixed areas with a superficial predominance, and in graft donor areas of partial thickness in older adult patients with a diagnosis of severe mixed burns who were admitted to the burn unit within a one-year period. The management protocol of early fluid resuscitation, antibiotic therapy, adequate nutrition, and surgical cleansing was followed, in addition to the use of allografts.

**Thursday 7 September 9–10:30 am**
**Session: Rehabilitation**
**O2.1.1**
**‘A Systematic Review on the Working Mechanisms of Signaling Pathways in Fibrosis during Shockwave Therapy’.**
**Lot Demuynck**, Ulrike Van Daele, Eric Van Breda, Peter Moortgat, Jill Meirte
University of Antwerp, Research Group MOVANT, OSCARE, Organisation for Burns, Scar Aftercare and Research, Antwerp, België
**Aim:**

Fibrosis is typically characterized by scarring and hardening of tissues and organs. It can affect every organ system and so can result in organ failure. Previous studies have suggested that mechanical forces (such as shockwave therapy, SWT) initiate a process of mechanotransduction, and thus could regulate fibrosis. Nevertheless, it is largely unexamined which pathways exactly are involved in the application of SWT. For this reason, the present article seeks to elucidate the underlying effect of SWT on fibrosis.

**Methods:**

A systematic review was conducted; this was achieved by gathering articles in three different databases: PubMed, Embase and Web of Science. As a result, a total of 3363 articles concerning the research question were extracted.

**Results:**

Preliminary evidence shows that SWT activates macrophage activity, fibroblast activity, collagen amount and orientation, TLR-3, TGF ß1/Smad, mTOR-FAK, YAP/TAZ, and apoptosis. The included articles reveal that depending on the energy levels of SWT, other proteins and pathways can be activated. Moreover, different frequency levels can have an influence on other proteins and pathways.

**Conclusions:**

These findings demonstrate that SWT has beneficial effects on fibrosis. Based on these data, which highlight the underlying mechanisms, we can make preliminary conclusions about the treatment modalities of SWT in scar formation, such as the energy levels and frequencies that are necessary to prevent or treat fibrotic tissue. The findings of this study have practical implications for the development of a more standardized SWT treatment.

**O2.1.2**
**A Pilot Study of Centralized Collection of Patient-Reported Outcome Measures in a Burns Population in the Australian State of Victoria**
**Heather Cleland** ^1^, Lincoln Tracy ^2^, Warwick Teague ^3^, Belinda Gabbe ^4^
^1 ^ Alfred Health, Melbourne, Australia
^2 ^ School of Public Health and Preventive Medicine, Monash University, Melbourne, Australia
^3 ^ Burns Service, The Royal Children’s Hospital, Melbourne, Australia
^4 ^ School of Public Health and Preventive Medicine, Monash University, Melbourne, Australia
**Aim:**

The aims of this initial analysis are to quantify patient preference for telephone or online follow-up, and to compare response rates and data completeness at the 12-month time point, based on follow-up method.

**Methods:**

This is a prospective cohort study of patients recruited from those registered by the Burns Registry of Australia and New Zealand (BRANZ) over a 12-month period commencing in 2021. Participants were given the opportunity to complete the questionnaires by telephone interview or to self-complete the questionnaires via an emailed link.

Adults completed the Burn-Specific Health Scale-Brief (BSHS-B), 5-D Pruritus Scale (5D) and 5-Level EuroQoL 5 Dimensions Questionnaire (EQ-5D-5L). 

Pediatric patients (aged <16 years) completed the Health Outcomes Burn Questionnaire (HOBQ), Children Burn Outcomes Questionnaire (CBOQ, and 5-Level EuroQoL 5 Dimensions Questionnaire (EQ-5D-Y) at 3, 6 and 12 months post-injury.

**Results:**

484 patients were approached and 450 consented to follow up, consisting of 423 adults and 27 pediatric patients. Most patients elected for follow-up by telephone. The 12-month follow up rates were 81% (22/27) for children and 78% (321/410) for adults. More patients preferred and then completed follow up via telephone than by email.

**Conclusions:**

Telephone follow-up is resource-intensive, but results in higher follow up rates than email completion. However, data completeness was high at all timepoints. These results encourage us to examine options for sustainable incorporation of long-term follow-up data into routine registry data collection.

**O2.1.3**
**Outcomes in the Dutch Value-Based Healthcare Burns Core Set: Looking beyond the Horizon**
**Denise Van Uden** ^1^, I Spronk ^1^, C.A. Lansdorp ^2^, C.H. van der Vlies ^1,3^, The National Burn Care, Education & Research group the Netherlands ^1,4,5^
^1 ^ Association of Dutch Burn Centers, Maasstad Hospital, Rotterdam, The Nederland
^2 ^ Amsterdam UMC location Vrije Universiteit Amsterdam, Department of Plastic Reconstructive and Hand Surgery, Amsterdam, The Netherlands
^3 ^ Trauma Research Unit Department of Surgery, Erasmus MC, Rotterdam, The Netherlands
^4 ^ Association of Dutch Burn Centers, Red Cross Hospital, Beverwijk, The Netherlands
^5 ^ Association of Dutch Burn Centers, Martini Hospital, Groningen, The Netherlands
**Aim:**

The Dutch burn care education and research program has developed a value-based healthcare (VBHC) burns core set consisting of outcomes and indicators that are important for burn patients, based on a national Delphi study. We assessed whether patients’, healthcare professionals’ (HCPs) and researchers’ views on the importance of outcomes differ between the subgroups in the different Delphi rounds.

**Method:**

A three-round modified Delphi study, including 45 outcomes and 23 quality indicators, was conducted in the three Dutch burn centers. Items could be rated on a 9-point Likert scale ranging from unimportant to important. Rankings of the importance of outcomes were compared between different rounds and different subgroups.

**Results:**

In the first round, ‘having a contact person’, ‘wound healing’, and ‘information on expected recovery’ were the top three most important outcomes according to 27 patients, while ‘pain’, ‘physical activity’, and ‘self-care’ were rated most important by 63 multidisciplinary HCPs. Researcher views (n = 23) were a mix of the other subgroups, with a top three containing ‘pain’, ‘wound healing’, and ‘patient-reported questionnaires’. The top three of the second round largely differed between the subgroups. The top three ranked items of the third round were similar between patients and HCP, while researchers views deviated.

**Conclusions:**

This study shows that patients, HCPs and researchers have an unique view on the most important burn care outcomes. Given the goal of VBHC is to learn from each patient by analyzing (patient-relevant) outcomes, it is crucial to include views of all parties involved, and to continuously discuss results as interpretation can change over time.

**O2.1.4**
**The Use of Deep Oscillation Therapy for the Treatment of Mature Burn Scars: A Pilot Study**
**Jeniffer Sánche**z ^1^, Marianela Rivero ^1^, Cynthia Arancibia ^1^, Ulrike Van Daele ^2,3^, Jill Meirte ^2,3^
^1 ^ COANIQUEM, Pudahuel, Chile
^2 ^ University of Antwerp, Rehabilitation Sciences and Physiotherapy, Antwerp, Belgium
^3 ^ Oscare, Antwerp, Belgium
**Aim:**

To explore the treatment effect of deep oscillation therapy (DO) on the treatment of burn scars.

**Methods:**

This is a pilot interventional study. Patients with mature burn scars were recruited at least 2 years after injury. Scar elasticity and thickness were assessed using DermaLab Combo at baseline, after the intervention, and at 4 weeks follow-up. The intervention consisted of five treatments within 2 weeks with DO Evident^®^, (Physiomed, Germany). The treatment duration was 15 min, using a combination of high-frequency (160 Hz, 8 min), intermediate-frequency (60 Hz, 4 min), and low-frequency (20 Hz, 3 min) modalities, according to the manufacturer’s indications.

**Results:**

Eight patients (age 13.6 ± 5.3 years), with mature burn scars (mean time from injury up to therapy 11.0 ± 6.1 years) were recruited. The median elasticity varied from 93.5% in the baseline to 96.0% after the intervention, (*p* = 0.63). The thickness of burn scars varied from 1343 µm to 1165 µm after the intervention (*p* = 0.14). The follow-up data showed that no further changes were observed. No adverse events or uncomfortable feelings in the scars were reported during the whole study period.

**Conclusions:**

The use of DO did not lead to significant changes in flexibility or thickness at the end of the treatment. The intervention appears to be safe and well tolerated by patients. A randomized controlled trial recruiting burn patients at different scarring stages and evaluating the effectiveness of objective, subjective scar characteristics as well as patient-reported outcomes is warranted to assess the efficacy of DO therapy.

**O2.1.5**
**The Role of Exercise in Wound Healing: Results of a Feasibility Study of Exercise Training.**
**David R. Schieffelers** ^1^, Dorien Dombrecht ^1^, Eric van Breda ^1^, Ulrike Van Daele ^1,2^
^1 ^ Department of Rehabilitation Sciences and Physiotherapy, Faculty of Medicine and Health Sciences, University of Antwerp, Antwerp, Belgium
^2 ^ OSCARE, Organization for burns, scar after-care and research, Antwerp, Belgium
**Aim:**

The catabolic and hyperglycemic response to burn injury has been shown to interfere with wound healing. Conversely, anabolic and anti-hyperglycemic interventions can considerably improve wound healing. Exercise is such an intervention strategy, but is nevertheless underused in acute burn care. Resistance and aerobic exercise have traditionally only been administered after wound closure. The aim of this investigation was to study the feasibility and safety of a resistance and aerobic exercise program during a burn center stay.

**Methods:**

Burned adults ≥ 10%TBSA received either standard care or additional resistance and aerobic exercises, commenced as early as possible. The number of graft failures and feasibility (as the ratio of attempted to successfully completed exercise sessions) were analyzed.

**Results:**

Some 57 subjects (51 ± 15 years; 23 ± 15% TBSA) were recruited, of which 28 underwent exercises for a median length of 22 days [IQR 15–31] with a mean exercise intensity of 7.9 ± 1 on a 0–10 Borg scale of perceived exertion. There was no significant difference in graft failures between groups (exercise group: two failures in one subject vs. control group: three failures in three subjects, *p* = 0654). Of 412 planned exercise sessions, 330 were successfully commenced (80% feasibility), and 264 were completed according to protocol (64%). The main reasons for incomplete or failed sessions were surgery or postsurgical immobilization (60 sessions), and pain (44 sessions). Besides five episodes of dizziness and one episode of vomiting, no adverse events occurred.

**Conclusions:**

The early provision of resistance and aerobic exercise is safe and feasible. The potential benefits for wound healing should be investigated.

**O2.1.6**
**Explorative Study Investigating Burn Survivors’ Perspectives on Quality of Care Aspects**
Raaba Shagari Miriyam Thambithurai ^1,2^, **Lotte van Dammen** ^3^, Paul van Zuijlen ^4,5^, Nancy Van Loey ^1,6^, The National Burn Care, Education & Research group The Netherlands
^1 ^ Maasstad Hospital, Rotterdam, The Netherlands
^2 ^ Erasmus University Rotterdam, Rotterdam, The Netherlands
^3 ^ Dutch Burns Foundation, Beverwijk, The Netherlands
^4 ^ Red Cross Hospital, Beverwijk, The Netherlands
^5 ^ Amsterdam UMC, Amsterdam, The Netherlands
^6 ^ Utrecht University, Utrecht, The Netherlands
**Aim:**

Quality indicators are used to monitor and improve quality of care and for benchmark purposes. The perspectives of burn survivors, however, are not recognized in current sets of quality indicators, while patient-centered care gains importance.

The aim of this study was to explore burn survivors’ perspectives on quality aspects of burn care, and to translate their perspectives into patient-centered quality-of-care indicators.

**Methods:**

An explorative qualitative study was conducted in a patient panel group. First, a thematic analysis was applied to the focus groups to identify overarching themes. Second, patient-centered process quality indicators, informed by burn survivors’ valued aspects of care, were defined.

**Results:**

Ten burn survivors (mean age 48 years and mean% TBSA of 14%) participated in two focus groups. Four overarching themes were identified: (1) the importance of information tailored to the different phases of recovery; (2) the importance of significant others’ wellbeing and involvement during recovery; (3) the importance of a therapeutic relationship and low-threshold access to healthcare professionals to ensure care continuity; and 4) the importance of participating in decision making. Eighteen patient-centered process quality-of-care indicators within nine aspects of care were formulated. For example, regarding the aspect of pain assessment: ‘was pain medication evaluated?’.

**Conclusions:**

The overarching themes are reflected in patient-centered process quality indicators, which present a broadened and complementary view of existing clinical quality indicators. Evaluating these patient-centered quality indicators may increase quality of care and may refine patient-centered care.

**O2.1.7**
**Which Physiotherapist Takes Care of the Patient after Discharge from the Burn Center? A Survey, by Questionnaire, on the Training of Territorial Physiotherapists in the Treatment of Burn Patients**
Ilaria Galgani, Manola Bacchis
Università di Pisa, Italy
**Aim:**

Given the need to continue physiotherapy treatment long after hospital discharge, it is necessary to know what training and experience territorial physiotherapists have in the treatment of scars and burns.

**Methods:**

A questionnaire consisting of 14 questions was developed: 6 questions referred to personal details, work setting, and the frequency of treatment of burns and scars; 5 questions to the assessment and treatment of scars; and 3 questions to the training received and perceived training needs in burns and scars.

The questionnaire was sent by e-mail to physiotherapists of public and private institutions in Tuscany.

**Results:**

The questionnaire was sent to 63 physiotherapists. Some 62 of them answered the questionnaire; 58.7% have been physiotherapists for more than 10 years. Briefly, 82.2% of the respondents treat patients with scars “often” and “sometimes”, compared with 55.6% who have been trained on “scars”, “burns”, or “both”. Assessment and treatment differ widely among the respondents, but 82.5% believe they need further training on ‘scars’, ‘burns’, or ‘both’.

**Conclusions:**

The analysis shows that in spite of the high percentage of territorial physiotherapists taking care of patients with scars, assessment and treatment differ greatly from practitioner to practitioner. Training therefore becomes essential in order to guarantee quality and homogeneous physiotherapy for patients throughout the territory.

In the future, it would be interesting to provide training courses from the burn center to territorial staff, and to verify their effectiveness.

**O2.1.8**
**The Reliability and Validity of Elasticity and Color Measurements in Surgical Scars.**
**Lot Demuynck**, Ulrike Van Daele, Eric Van Breda, Jill Meirte
Research Group MOVANT, Department of Rehabilitation Sciences and Physiotherapy, Antwerp, België
**Purpose:**

To examine the reliability and validity of the Cutometer and Mexameter in the measurement of, respectively, the elasticity and redness of surgical scars.

**Method:**

Participants with linear surgical scars of a minimum of 2 cm long and between 2 weeks–8 months old were enrolled in this study. Some 32 participants (12 male; 20 female) were enrolled, aged 19–73 years (mean 40.91 ± 17.66), with scars on various locations, for instance knees, backs, and hands. The participants were asked to come to Antwerp University for measurement of the scars. After filling in the informed consent and questionnaires (patient information and POSAS), measurements with the Cutometer and Mexameter were performed by two researchers.

**Results:**

The intra-rater reliability of color measurement with the Mexameter is excellent (ICC > 0.9), the inter-rater reliability of color measurement with the Mexameter ranges between fair and excellent (ICC = 0.5–1). The inter-assessor reliability of elasticity measurement with the Cutometer is good to excellent (ICC = 0.6–1). No correlation was found between OSAS and color or elasticity measurements.

**Conclusions:**

The Cutometer and Mexameter are reliable and objective scar assessment tools to evaluate linear scars and the healthy skin. These devices are suitable for follow-up measurements.

**Thursday 7 September 9–10:30 am**
**Session: Basic research**
**O2.2.1**
**An In Silico Modeling Approach to Understanding the Dynamics of the Post-Burn Immune Response.**
**H. Ibrahim Korkmaz** ^1,2,3,4^, Vivek M. Sheraton ^5,6^, Anouk Pijpe ^1,3^, Bouke B. Boekema ^1,4^, Evelien de Jong ^7^, Stephan G.F. Papendorp ^7^, Esther Middelkoop ^1,4^, Peter M.A. Sloot ^5,8^, Paul P.M. van Zuijlen ^1,3,9^

^1^  Department of Plastic Reconstructive and Hand Surgery, Amsterdam Movement Sciences (AMS) Institute, Amsterdam UMC, location VUmc, Amsterdam, The Netherlands
^2^  Department of Molecular Cell Biology and Immunology, Amsterdam UMC, location VUmc, Amsterdam, The Netherlands
^3^  Burn Center, Red Cross Hospital, Beverwijk, The Netherlands
^4^  Association of Dutch Burn Centers (ADBC), Beverwijk, The Netherlands
^5^  Informatics Institute, Institute of Advanced Studies, University of Amsterdam, Amsterdam, The Netherlands
^6^  Laboratory for Experimental Oncology and Radiobiology, Center for Experimental and Molecular Medicine, Cancer Center Amsterdam and Amsterdam Gastroenterology Endocrinology and Metabolism; Oncode Institute, Amsterdam UMC, location AMC, Amsterdam, The Netherlands
^7^  Department of Intensive Care, Red Cross Hospital, Beverwijk, The Netherlands
^8^  ITMO University, Saint Petersburg, Russian Federation
^9^  Paediatric Surgical Center, Emma Children’s Hospital, Amsterdam UMC, location AMC, Amsterdam, The Netherlands

**Aim:**

The aim of this study is to develop simulation models for the post-burn immune response based on existing (pre)clinical data from the literature.

**Methods:**

The simulation domain was separated into blood and tissue compartments. Each of these compartments contained solutes and cell agents. Solutes comprise pro-inflammatory cytokines, anti-inflammatory cytokines and inflammation-triggering factors, e.g., damage-associated molecular patterns (DAMPs). The solutes diffuse around the domain based on their concentration profiles. The cells include mast cells, neutrophils, and macrophages, and were modeled as independent agents. The cells are motile and exhibit chemotaxis based on concentrations gradients of the solutes. In addition, the cells secrete various solutes that in turn alter the dynamics and responses of the burn wound system. Endothelial cells were modeled as fixed positions inside the burned area from where solutes and cell agents enter the wound area, and vice versa.

**Results:**

We have developed an agent-based model to capture the complexity associated with the post-burn dynamics of inflammation [1,2], including changes in cell counts and cytokine concentrations. The initial endothelial cell number, which refers to the number of blood vessels, exhibits the greatest influence on inflammation due to assumptions, with the lower numbers exhibiting more acute inflammation as a result of higher levels of the pro-inflammatory cytokine IL-6. In addition, the chemotactic affinity of the cells affects the inflammation response after burns too.

**Conclusions:**

The current model successfully simulates significant factors influencing the post-burn immune response, i.e., the initial endothelial cell number, the chemotaxis threshold, and chemotaxis intensity.

**O2.2.2**
**3D Bioprinting in Reconstructive Surgery—An Approach for Creating Bioactive Dressing as a Base for Skin Substitute**
**Iren Bogeva-Tsolova** ^1^, Miroslav Dobrev ^2^, George Altankov ^3^, Dobromir Dimitrov ^4^
^1 ^ Department of Surgical diseases, Clinic of Plastic and reconstructive surgery, Medical University Pleven, Bulgaria
^2 ^ Department of Anatomy, histology and embryology, Medical University Pleven, Bulgaria
^3 ^ Center of competence Medical University Pleven, Bulgaria
^4 ^ Clinic of Oncology surgery, Georgi Stranski University Hospital, Pleven, Bulgaria, Medical University Pleven, Bulgaria
**Introduction:**

In recent years, three-dimensional bioprinting has made great progress in the creation of tissues. The problem with the created products occurs with their translation into clinical practice, and their survival rate once transferred. The eventual success of bioprinted tissues would be an exceptional contribution to the fields of regenerative medicine and reconstructive surgery.

**Aim:**

At Medical University Pleven, we are aiming to follow the best researchers in the tissue engineering field. Our preliminary results present different approaches to creating the first layer of a three-dimensional bioprinted skin substitute which acts as bioactive dressing.

**Methods:**

The process includes the bioprinting of collagen type I droplets with embedded cells. As a scaffold, polypropylene or a silicone mesh is used, upon which the collagen, polycaprolactone, and mesenchymal stem cells are printed. Before translation, the patches underwent sterilization. For the experiment, both male and female Wistar rats were used.

**Results:**

The follow up showed great acceptance by the organism and no rejection signs. The average time for complete recovery of the created full-thickness defect was 10 days. A month after grafting, all the experimental animals showed no difference in appearance than before the defect was created. A skin biopsy was taken to document the newly formed skin.

**Conclusions:**

The first line of 3D bioprinted patches showed great endurance on the animal organism and no signs of rejection, infection, or any other disturbances. The results of our experiment will be used to proceed with the next layer of skin substitute, adding nanofibers to resemble the organization of the extracellular matrix.

**O2.2.3**
**The Impact of Age on the Immune Response and Angiogenesis in Full-Thickness Burns**
**Anna-Lisa Pignet** ^1,2,3^, Manuel Prevedel ^1,2,3^, Julia Fink ^1,2,3^, Johanna Einsiedler ^1,2,3^, Petra Kotzbeck ^1,2,3^
^1 ^ Division for Plastic, Aesthetic and Reconstructive Surgery, Medical University of Graz, Graz, Austria
^2 ^ JOANNEUM RESEARCH Forschungsgesellschaft mbH, COREMED—Center of Regenerative and Precision Medicine, Graz, Austria
^3 ^ Research Unit for Tissue Regeneration, Repair and Reconstruction, Division for Plastic, Aesthetic and Reconstructive Surgery, Medical University of Graz, Graz, Austria
**Background:**

Although the survival rate after extensive burns is diminished in the elderly, the age factor has often been neglected in previous in vivo studies.

**Aim:**

Here, we characterized systemic and local reactions following burn injuries in 11-, 27- and 56-week-old Wistar rats (n = 36).

**Methods:**

The animals either received four full-thickness contact burns or served as unwounded controls. Non-invasive imaging methods were applied, and blood was collected from the tail vein to detect differences in plasma cytokine concentrations. Body weight and food intake were measured daily. At 7 days post burn (7 dpb), tissue biopsies were collected and analyzed on a histologic and molecular level (qPCR).

**Results:**

Tissue perfusion was significantly impaired in 27- and 56-week-old rats compared to 11-week-old rats. Moreover, 7 dpb immune cell infiltration into wounds was increased in the 27 and 56-week-old rats. KC-GRO was also increased in the serum of the 27-week-old animals already before the burn, as well as 1 and 7 dpb. In contrast, Ccl-2 gene expression was elevated in the punch biopsies of the 11-week-old rats, both in the burnt and the control animals. Nevertheless, there were no significant differences in wound sizes, nor in weight loss after burns.

**Conclusions:**

Although there are age-related differences in angiogenic potential and immune response, they do not seem to affect the healing of small burn wounds significantly.

**O2.2.4**
**Monocyte, Lymphocyte, and Neutrophil Extracellular Traps Are Present in the Dermal Microvasculature of Burn Wounds and Coincide with a Procoagulant Phenotype**
**Britt Van Der Leeden** ^1,2^, H. Ibrahim Korkmaz ^3,4,5^, Bouke Boekema ^3,4^, Paul van Zuijlen ^3,4,7^, Hans Niessen ^1,6^, Susan Gibbs ^5^, Paul Krijnen ^1,6^
^1 ^ Department of Pathology, Amsterdam UMC, Amsterdam, The Netherlands
^2 ^ Amsterdam Infection and Immunity, Amsterdam UMC, Amsterdam, The Netherlands
^3 ^ Association of Dutch Burn Centers, Beverwijk, The Netherlands
^4 ^ Department of Plastic, Reconstructive and Hand Surgery, Amsterdam, The Netherlands
^5 ^ Department of Molecular Cell Biology and Immunology, Amsterdam, The Netherlands
^6 ^ Amsterdam Cardiovascular Sciences, Amsterdam, The Netherlands
^7 ^ Red Cross Hospital, Beverwijk, The Netherlands
**Aim:**

This study aimed to investigate the presence of monocyte extracellular traps (METs), lymphocyte extracellular traps (LETs), and neutrophil extracellular traps (NETs) in the burn wound coinciding with the local pro-coagulant phenotype in the microcirculatory endothelium after burn injury.

**Methods:**

Eschar was operatively obtained from burn wound patients (n = 21) with a mean total body surface area (TBSA) burned of 29%. Herein, the coagulation factors, i.e., tissue factor (TF) and factor XII (FXII), together with the endothelial cell marker CD31, were studied using immunohistochemistry. The presence of NETs, METs and LETs was analyzed using immunofluorescence. For this, CD31 and the extracellular trap (ET) marker histone 3 citrullin were combined with immune cell markers for monocyte-, lymphocyte- and neutrophil extracellular traps: CD14 (METs), CD45 (LETs) and myeloperoxidase (MPO: NETs).

**Results:**

Increased expression of TF, FXII and CD31-positive thrombi was found intravascularly in all eschar samples compared to uninjured skin. Neutrophils were the most predominant cell type of the immune cell infiltrate in eschar. NETs, METs and LETs were found in the lumen of the dermal microvasculature in the eschar tissue 7 up to 40 days post-burn. The presence of NETs was the most predominant, and significantly correlated with coagulatory factors TF and FXII and the percentage of CD31-positive thrombi in CD31+ vessels.

**Conclusions:**

This study shows that ETs are present in the microcirculation of burn wounds and coincide with increased CD31-positive thrombi, FXII and TF expression, which may contribute to the hyper-coagulatory state after burns and burn wound conversion.

**O2.2.5**
**Prognostic Factors in Burned Pregnant Women**
Hana Fredj, Bahija Gasri, **Amel Mokline**, Sarra zarrouk, Imen Jemi, Manel Saad, Amen Allah Messadi
Burns Intensive Care Department, Traumatology and Burn Center, Ben Arous, Tunisia
**Aim:**

The aim of our study was to identify the predictive factors of maternal and fetal mortality in patients burned during pregnancy

**Methods:**

This is a descriptive retrospective study conducted over a period of 15 years (2007–2022), including all burned pregnant women admitted to the Burn Intensive care department at the trauma and burn center in Tunisia. Demographic (circumstances of burns), clinical (age, burned surface area), and evolutionary data were collected. Uni and multivariate analyses were performed using SPSS22.

**Results:**

Twenty six pregnant females were included. The mean age was 28 ± 5 years. The mean total body surface area (TBSA) was 32%. The term of the pregnancy was greater than 24 weeks in 11 cases. The maternal mortality rate was 31% (n = 8), secondary to septic shock in all cases. The fetal mortality rate was also 31%. Shock on admission (*p*: 0.02), use of mechanical ventilation (*p*: 0.04), initial hyperglycemia (*p*: 0.02), occurrence of sepsis (*p*: 0.03), burns secondary to a suicide attempt (*p*: 0.02), and deep burns (*p*: 0.03) were factors in poor fetal and maternal prognosis. TBSA greater than 35% was identified as a predictor of maternal mortality (*p*: 0.001; AUC: 0.993; 95% CI: 0.97–1) and fetal mortality (*p*: 0.04; ASC: 0.792; 95% CI: 0.59–0.98).

**Conclusions:**

Burns in pregnant women are associated with high maternal and fetal mortality. The prognosis essentially depends on the extent of the burns.

**O2.2.6**
**Blood Lactate Levels in Predicting the Mortality of Patients with Severe Burns**
**Dorotea Zagorac**, Ana Mesić, Agata Škunca, Tihana Magdić Turković
Sestre milosrdnice University Hospital Center, Zagreb, Croatia
**Aim:**

To analyze blood lactate levels in patients with severe burns.

**Methods:**

This retrospective study included patients admitted to the burn intensive care unit (BICU) between January 2016 and December 2022 with a total body surface area (TBSA) burned ≥ 20%. Out of 193 patients admitted to the BICU, 87 patients were included in the study. Blood lactate levels were analyzed within 7 days after the injury. We analyzed the prediction value of adding the blood lactate level and number of comorbidities to the rBaux score.

**Results:**

Regression coefficients for lactate on 7th day after the injury and the presence of two or more comorbidities compared to none and the rBaux score were found to be statistically significant. The odds ratio for lactate on the 7th day was estimated at 6.016 (95% CI of 1.430–25.311), the presence of two or more comorbidities compared to none 4.378 (95% CI of 1.083–17.690), and the rBaux score 1.047 (95% CI of 1.083–17.690). Based on these results, we concluded that with an increase of 1 in lactate, the odds of a lethal outcome are six times higher, and patients with two or more comorbidities have a four times higher chance of a lethal outcome compared to those with none; with an increase of 10 rBaux points, the odds of a lethal outcome are expected to increase by 58.8%. The area under the ROC curve was 0.85.

**Conclusions:**

the addition of lactate levels and the presence of comorbidities to the rBaux score improves mortality prediction in severe burns.

**O2.2.7**
**A Multi-Scale Scar Model in Burn Care: From Micro- to Macroscale**
**H. Ibrahim Korkmaz** ^1,2,3,4^, Ginger Egberts^5,6^, Anouk Pijpe ^1,3^, Fred Vermolen^6^, Paul P.M. van Zuijlen ^1,3,7^
^1 ^ Department of Plastic Reconstructive and Hand Surgery, Amsterdam Movement Sciences (AMS) Institute, Amsterdam UMC, location VUmc, Amsterdam, The Netherlands
^2 ^ Department of Molecular Cell Biology and Immunology, Amsterdam UMC, location VUmc, Amsterdam, The Netherlands
^3 ^ Burn Center, Red Cross Hospital, Beverwijk, The Netherlands
^4 ^ Association of Dutch Burn Centers (ADBC), Beverwijk, The Netherlands
^5 ^ Delft Institute of Applied Mathematics, Delft University of Technology, Delft, The Netherlands
^6 ^ Research Group Computational Mathematics (CMAT), Department of Mathematics and Statistics, University of Hasselt, Hasselt, Belgium
^7 ^ Paediatric Surgical Center, Emma Children’s Hospital, Amsterdam UMC, location AMC, Amsterdam, The Netherlands
**Aim:**

This study aims to develop a multi-scale scar model in burn care incorporating data from micro- to macroscale from preclinical and clinical research, regular care, quality and outcome registrations.

**Methods:**

By forming an interdisciplinary network between various disciplines, e.g., intensive care, surgery, therapists, researchers (both clinical and fundamental), and with the participation of patient representatives, processes that play a role during scar formation after burns will be studied using computational modeling. Data will be collected from scars (formation) from preclinical (in vitro) models and correlated to clinical data, i.e., data from the Patient and Scar Assessment Scale (POSAS), and integrated into the multi-scale scar model. In this way, dynamic processes and tissue organization occurring during scar formation can be investigated.

**Anticipated results:**

We expect to develop dynamic multi-scale scar models for burn wounds, which will be continuously fed by new (generated) data from micro- to macroscale, it being a continuous learning model for scar formation after burns. Eventually, this will deepen our knowledge about processes that play a role during scar formation after burns, and may serve as a predictive tool for post-burn dermal evolution, with the major goal of optimizing clinical practice.

**Conclusions:**

Because of the complex nature of scars, which implies the urgency of collaboration between many disciplines, and also patients, a multi-scale scar model is indispensable. Here, we introduce a basic conceptual model for a multi-scale scar model in burn care.

**O2.2.8**
**The Effects of Fish Skin on Wound Healing Progression in a Standardized Pig Model**
**Anna-Lisa Pignet** ^1,2,3^, Elisabeth Hofmann ^1,2,3^, David Hahn ^1,2,3^, Lars-Peter Kamolz, Petra Kotzbeck ^1,2,3^
^1 ^ Medizinische Universität Graz, Graz, Austria
^2 ^ Division of Plastic Aesthetic and Reconstructive Surgery, Research Unit for Tissue Regeneration, Repair and Reconstruction, Graz, Austria
^3 ^ JOANNEUM RESEARCH Forschungsgesellschaft mbH, COREMED—Center of Regenerative and Precision Medicine, Graz, Austria
^4 ^ Research Unit for Tissue Regeneration, Repair and Reconstruction, Division for Plastic, Aesthetic and Reconstructive Surgery, Medical University of Graz, Graz, Austria

Introduction: Fish skin grafts have proven to be effective in acute and chronic wound healing, as well as burns. However, their underlying mechanisms promoting healing are still unknown.

**Aim:**

The aim was to investigate the effects of fish skin grafts on wound healing properties under standardized conditions in a full-thickness skin defect pig model.

**Materials and Methods:**

Fish skin grafts were tested on a full-thickness skin defect (3 cm × 3 cm) pig model (n = 6; male landrace pigs) and compared intra-individually to control wounds (foam dressing). The experiment lasted for 21 days. Reapplication of the dressings was carried out after 9 days post-wounding. Wound scoring, photo-documentation and non-invasive imaging methods were carried out 5, 9, 14, and 21 days post wounding. Tissue biopsies were sampled at the same time points, and subjected to histologic analysis (HE and Masson’s trichrome stains).

**Results:**

As soon as on day 5, wounds treated with fish skin grafts showed more granulation tissue compared to control wounds. Planiometry revealed lower residual wound areas of the wounds treated with fish skin, especially on day 9 and 14 post-wounding. On day 21, the control wounds showed severe signs of wound contraction compared to the treated wounds. Thermography revealed significant higher surface temperatures in fish skin-treated wounds on day 9 and day 14.

**Conclusions:**

Fish skin grafts seem to support wound healing due to their accelerated formation of granulation tissue and their prevention of wound contraction in a pre-clinical full-thickness wound model.

**Thursday 7 September 9–10:30 am**
**Session: Prevention**
**O2.3.1**
**An Artificial Intelligence Language Model Can Improve the Readability of Burns First Aid Information**
Alexander Baldwin
Department of Burns and Plastic Surgery, Buckinghamshire Healthcare NHS Trust, Aylesbury, UK
**Aims:**

To assess whether an artificial intelligence (AI) language model can improve the readability of burns first aid information.

**Methods:**

The top 50 English-language webpages containing burns first aid information were compiled. An AI language model (ChatGPT) was prompted to rewrite these so that they could be understood by an 11-year-old, the target advised by the American Medical Association (AMA) and Health Education England (HEE) for patient education materials (PEMs). Readability was assessed using five tools: the Flesch Reading Ease Score (FRES), Flesch-Kincaid Grade Level (FKGL), Gunning Fog Index (GFI), Coleman-Liau Index (CLI), and Simple Measure of Gobbledygook Index (SMOG).

**Results:**

The mean readability scores of the unmodified PEMs were: FRES 73.6, where the target was ≥80; FKGL 6.0; GFI 8.2; CLI 8.3; SMOG 6.1, where the target grade was ≤6.9. Post-modification mean scores were FRES 82.2; FKGL 4.9; GFI 7.4; CLI 6.9; SMOG 6.1. Once rewritten using AI, a paired t-test demonstrated that all readability scores improved significantly (*p* < 0.001). Two (4%) of the unmodified PEMs were judged to be at the target reading level using all tools. The average ‘median grade score’ was 6.9 (SD = 1.1). A one-sample one-tailed t-test demonstrated that this was not significantly below the target level (*p* = 0.31). Following AI modification, nine (18%) PEMs were at the target level using all tools. The average ‘median grade score’ improved to 6 (SD = 0.9, *p* < 0.001).

**Conclusions:**

Much of the burns first aid information available online is written above the recommended reading level. An AI language model can improve this information’s readability to better meet the level advised by AMA and HEE.

**O2.3.2**
**The Epidemiology of Burn Injuries in Pediatric Patients with and without Migrant Backgrounds in Chile**
Kaitlin Danziger ^1^, Paige Bartholomew ^1^, Rodrigo Fuentes ^2^, **Orlando Flores** ^2^
^1 ^ School of Medicine, University of Southern California, Los Angeles, CA, United States
^2 ^ COANIQUEM, Santiago, Chile
**Aim:**

To describe the epidemiological differences among migrant and non-migrant children admitted to COANIQUEM burn centers in Chile.

**Methods:**

This is a retrospective, observational study of pediatric burn patients admitted to COANIQUEM facilities in Chile between January 2019 and December 2022. Patients were categorized as migrants if they or at least one of their parents reported non-Chilean nationality. Categorical and continuous variables were presented as counts and frequencies. Categorical variables were compared between patient groups using chi-square, Fisher’s exact, and proportion tests.

**Results:**

A total number of 9345 patients were enrolled during the study period. Of these, 699 were migrants. The migrant patients were younger, received sub-optimal first aid practices more frequently, took longer to seek medical care, and suffered larger burns overall. Some 20% of migrant patients were missing insurance information. Significant differences in burn mechanisms between migrant and non-migrant patient groups were found.

**Conclusions:**

This study showed the need for injury prevention programs that are sensitive to the cultural and sociodemographic differences of the migrant population. Further investigation is required to assess the accessibility of and opportunity for burn care for the migrant population within the Chilean healthcare system.

**O2.3.3**
**Transforming Epidemiological Data into Burns Prevention Campaigns: The COANIQUEM Experience**
**Jorge Rojas-Zegers**, Carmina Domic, Rodrigo Fuentes, Orlando Flores
Coaniquem, Santiago de Chile, Chile
**Aim:**

To describe the process followed at COANIQUEM, a specialized burn center in Chile, to produce prevention campaigns using epidemiological data.

**Methods:**

A phenomenological, qualitative approach was followed. Semi-structured interviews were conducted with decision-makers on six levels (clinicians, epidemiologists, outreach specialists, outreach directors, designers, and the communication team) using a purposive sample. The interviews were recorded, transcribed and analyzed using thematic analysis. Themes were constructed to understand the sources of information and decision-making process used to build the prevention campaigns and evaluate the results obtained.

**Results:**

The process of producing a prevention campaign in COANIQUEM is described, emphasizing the decision-making process performed at each level. The sources of information used in each stage of the process are standard and contribute to collaborative decision-making. The process was based on the expertise of the involved professionals, and concluded with the evaluation of the campaign. The campaigns were considered successful.

**Conclusions:**

COANIQUEM’s approach to producing prevention campaigns has proved to be effective. The transdisciplinary collaborative team works in a productive chain, obtaining a high-impact campaign as a result. Continuous feedback across all levels of the process is warranted to continue improving the quality of the prevention campaigns. This model might be helpful for other burn prevention teams.

**O2.3.4**
**Epidemiological Characteristics of Burn Injuries in Chilean Adolescent Patients**
Paige Bartholomew ^1^, Kaitlin Danziger ^1^, Rodrigo Fuentes ^2^, **Orlando Flores** ^2^
^1 ^ School of Medicine, University of Southern California, Los Angeles, CA, United States
^2 ^ Research Department, COANIQUEM, Santiago, Chile
**Aim:**

To investigate the epidemiological characteristics of Chilean adolescent patients and to compare them to patients aged 0–4 years old.

**Methods:**

A retrospective observational study of all acute burn injuries admitted to COANIQUEM facilities from January 2019, to December 2022. Pediatric patients with acute burn injuries aged 0–4 or 10–19 years old were included in the analysis. Demographic and injury characteristics were analyzed using descriptive statistics, and comparisons between the two age groups were made using a Wilcoxon rank sum test and chi-squared test or Fisher’s exact test. A proportion test was used to identify differences within variable categories by age group.

**Results:**

A total of 7482 patients were included in the analysis. The majority of the adolescent patients were females (56%, n = 1054) who sustained burn injuries to their lower body (37%, n = 696) and were mostly injured by a hot liquid (67%, n = 1262). Statistically significant differences in epidemiological characteristics between children 0–4 years old and adolescents were found for sex (*p* < 0.001), the patient’s living situation (*p* < 0.001), their mother’s education (*p* < 0.001), the injury season (*p* < 0.001), total body surface area (*p* = 0.005), the injury agent (*p* < 0.001) and mechanism (*p* < 0.001), first aid treatment (*p* < 0.001), and injury location (*p* < 0.001).

**Conclusions:**

Our data showed that there are significant differences in the demographic and clinical characteristics of adolescents suffering burn injuries compared to their younger peers. These findings can guide future research and prevention campaigns.

**O2.3.5**
**Implementing Outcomes of Aetiological Research in an Online Programme to Prevent Burn Accidents in Children under 5 Years of Age**
**Eva Van Zoonen** ^1^, A. Meij-de Vries ^2,3,4^, M.E. van Baar ^3,5^, C.H.M. van Schie ^1^, C.H. van der Vlies ^6,7^
^1 ^ Dutch Burns Foundation, Beverwijk, The Netherlands
^2 ^ Burn Center, Red Cross Hospital, Beverwijk, The Netherlands
^3 ^ Association of Dutch Burn Centers (ADBC), Beverwijk, The Netherlands
^4 ^ Pediatric Surgical Center, Emma Children’s Hospital, Amsterdam, The Netherlands
^5 ^ Department of Public Health, Erasmus MC, Rotterdam, The Netherlands
^6 ^ Burn Center, Maasstad Hospital, Rotterdam, The Netherlands
^7 ^ Erasmus MC, Trauma Research Unit, Department of Surgery, Rotterdam, The Netherlands
**Aim:**

The aim was to implement the outcomes of an aetiological prospective cohort study in an online programme to prevent burn accidents in children under 5 years of age.

**Methods:**

Data on the percentage- and aetiology of burn accidents per age group were obtained from an aetiological cohort study. The data were linked to relevant development stages in children under 5 years of age from the Dutch Development Instrument (van Wiechenonderzoek). Subsequently, a milestone alert matrix was developed, in which age (in months) was linked to the percentage of burn accidents, development stages, and associated safety hazards in that age group.

**Results:**

For each relevant development stage in the milestone alert matrix, a specific digital newsletter was created in collaboration with communication experts. Information on the characteristics of a specific development stage together with information on how to prevent burn accidents in that particular stage were provided in an positively phrased and easy-to-read newsletter format. With targeted advertisements, parents were encouraged to enrol in the newsletter flow. As of now, approximately 14,500 parents have subscribed. The effectiveness of the campaign was measured using an online questionnaire, and showed an increase in knowledge and self-reported safety behaviour.

**Conclusions:**

This study shows that epidemiological data can successfully be implemented in an online prevention program, with an increase in the knowledge and safety behaviour in parents of children under 5 years of age.

**O2.3.6**
**Assessing the Effect of the Cost-of-Living Crisis on Hot Water Bottle-Related Burns in the United Kingdom, a Single-Center Retrospective Observational Study**
**Mahaveer Sangha**, Miss Michelle Baker, Mr Alexander Baldwin, Miss Alexandra Murray
Stoke Mandeville Hospital, Department of Plastic & Reconstructive Surgery, Aylesbury, UK
**Aim:**

To assess whether the Cost-of-Living Crisis (CoLC), an ongoing economic period in the United Kingdom (UK) in which the cost of essential commodities exceeds household income, is associated with an increase in hot water bottle-related (HWBR) burns.

**Methods:**

Records of patients admitted with HWBR burns between December 2019 and March 2023 were reviewed and the following data collected: patient demographics, burn depth and surface area, patient comorbidities, and patient index of multiple deprivation (IMD). The incidence of admissions, IMD, and severity of injury were compared prior to and during the CoLC using either an independent T-test or a Kruskal–Wallis H test. The start of the CoLC was defined as October 2021, the first month in which the UK Office of National Statistics identified rising energy costs.

**Results:**

Between December 2019 and March 2023, 177 patients were admitted with HWBR burns, 79 prior to the CoLC and 98 during. 55 patients were male and 122 female. An independent T-test comparing average monthly admissions prior and during the CoLC identified a significant difference (*p* = 0.042), with a mean increase of 1.85 cases (95% CI: 0.71–3.63). Additionally, a Kruskal–Wallis H test showed statistically significant difference in the number of patients admitted with HWBR burns between the seasons (*p* = 0.001). An independent T-test comparing average patient IMD prior and during the CoLC identified no difference (*p* = 0.583).

**Conclusions:**

The increase in HWBR burns coincides with the rise in energy costs and the general cost of living that has occurred in the UK since October 2021.

**O2.3.7**
**Electrical Burns in Train Climbers Treated in the Helsinki Burn Center during the Last Thirty Years**
**Eve Kinnunen**, Aliisa Korkiamäki, Andrew Lindford, Jyrki Vuola
Helsinki Burn Center, Department of Plastic and Reconstructive Surgery, Helsinki University Hospital and University of Helsinki, Helsinki, Finland
**Aim:**

To review electrical burns and their outcomes in train climbers treated in the Helsinki Burn Center during the last thirty years.

**Methods:**

This is a retrospective study of electrical burn patients admitted to the Helsinki Burn Center between November 1993 and December 2022. Of 138 patients with electrical burns, 16 (11.6%) had climbed onto the roof of a train and were included in the study. Patients’ charts were reviewed, and several trauma- and outcome-related variables were collected.

**Results:**

Some 14 (87.5%) of the 16 patients were male. The mean age of the patients was 16.9 years (range: 13–29 years). All the burns were high-voltage electrical burns. One of the burns was occupational. Ten (62.5%) patients had an additional suspected flame burn. None of the incidents were a suicide attempt. The mean burn size was 46% of the total body surface area. Three (18.8%) patients died during their in-hospital stay. The mean length of in-hospital stay was 51 days. On average, the patients required six operations (range: 0–32) during the index hospitalization. Nine (56.3%) patients needed escharotomies, seven (43.8%) fasciotomies, twelve (75.0%) skin grafting, two (12.5%) a local or pedicled flap, one (6.3%) a microvascular flap, one (6.3%) a minor amputation, and three (18.8%) a major amputation. Two (12.5%) patients required temporary renal replacement therapy.

**Conclusions:**

Train climbers represent a rare group of young patients with electrical burns. Precautionary strategies should be implemented to prevent these injuries that are associated with a significantly high morbidity and mortality.

**O2.3.8**
**The Use of Different Social Media Platforms to Deliver Burns Prevention Information**
**Mrs Nicole Lee**
Chelsea and Westminster, London, UK
**Aim:**

To review the efficacy of different social media platforms for delivering burn prevention messages in the UK.

**Method:**

Different burn prevention messages were released on different social media platforms, and views and interactions were reviewed to see if there was one platform which was better for publishing prevention campaigns. Facebook, Twitter, LinkedIn and TikTok were used.

**Results:**

Numbers of interactions and views were related to followers, likes, topics and hashtags. Seasonally related topics drove up views and interactions with one viral prevention campaign release. Media interest was seen with topical subjects, which lead to a message’s inclusion in government policy changes in relation to one hot topic.

**Conclusions:**

An overall increase in views and interactions was seen, with seasonal and hot topics leading to viral content. The use of different social media platforms allows access to different people, so even low numbers of interactions over time will increase followership, thereby leading to the generation of wider reach.

**Thursday 7 September 2–4 pm**
**Session: Pediatrics**
**O2.7.1**
**How Have the German AWMF Guideline and the Quality Certification Influenced the Distribution of Pediatric Burn Patients Across Germany?**
**Katharina Schriek**, Steffen Wahler, Mechthild Sinnig
Pediatric Hospital Auf Der Bult, Hannover, Germany

This retrospective study aims to determine the influence of the guideline and quality certification on the care structure for children with burns over the 2013–2021 period.

**Methods:**

We evaluated the quality reports of all German hospitals provided by the joint federal committee for burn wounds as the main research, further subdividing these based on burn depth and body region.

**Results:**

The relative distribution of burn depth in 2021 for specialized clinics and centers versus peripheral clinics (total number) was for grade 2a: 46.9%/45.6% (1929/3971), grade 2b: 35.2%/28.4% (1447/3915) grade 3: 15.8%/17.7% (650/1541), and not specified: 1.5%/4.9% (63/411).

In 2013–2014, specialized clinics and centers treated 24.3% of all pediatric burns, in 2016–2018 this was 28.2%, and in 2020–2021 it was 31.6%. 

Concerning the special indicator “hand burns”, there was also an increase in the total number of patients treated in children’s departments, from 2157 patients (2013) to 2488 patients (2021), with a maximum number of 2931 cases in 2017. The proportion of second/third-degree burns of the hand treated at centers was 23.7%/31.2% in 2013–2014, 27.1%/28.9% in 2016–2018, and 34.1%/33.3% in 2020–2021.

**Summary:**

Comparing the periods before and after the implementation of the guideline and quality certification revealed a 7.3% increase in the treatment of all thermal injuries in childhood in specialized clinics and centers compared to treatments in peripheral hospitals, although no significant shift (+2%) could be detected for third-degree burns.

**O2.7.2**
**Burns of Immigrants and Refugees/Asylum Seeker Children: The Experience of a Single Pediatric Burn Center**
**Sabri Demir** ^1^, Fahri Akkaya ^1^, Suleyman Arif Bostanci ^1^, Irem Akbas ^1^, Emrah Senel ^2^
^1 ^ University of Health Sciences, Ankara Bilkent City Hospital, Children Hospital, Ankara, Turkey
^2 ^ Ankara Yildirim Beyazit University, School of Medicine, Department of Pediatric Surgery, Ankara, Türkiye
**Aim:**

We aimed to share our data about burns to immigrants and refugees/asylum seeker children who had to leave their homes due to the civil war, as treated in our pediatric burn center (PBC).

**Methods:**

Children who immigrated or lived in refugee camps in their own countries and were under the status of asylum seeker/refugee in Türkiye and were treated in our PBC included in the study. Demographic and clinical data were evaluated retrospectively and compared with Turkish patients. *p* < 0.05 was considered significant.

**Results:**

Between 01 January 2011 and 31 March 2023, 2036 burned-children treated. Of these, 317 (15.6%) were immigrants or refugees/asylum seekers. Their length-of-stay at PBC was longer than Turkish (24.9 vs. 15.9 days, *p* < 0.001), total-burned-body-surface-area was bigger (19.4 vs. 14.1%, *p* < 0.001), the incidence of fire/flame burns was higher (32.7% vs. %17.5, *p* < 0.001), and grafting rates were higher (45.0% vs. 32.1%, *p* < 0.001). Their mortality rate was four-times higher (7.5% vs. 2.0%, *p* < 0.001). Of these, 79.8% were Syrian, 10.1% Iraqi, 8.5% Afghan, and 1.6% Somalian. Syrian victims were injured mostly in the winter months and by the flame/fire burns caused by the fuel-stove used for heating in refugee camps. The second most common cause was the fires caused by other reasons in the tents/barracks, followed by bomb explosions.

**Conclusions:**

Immigrants and asylum-seekers/refugees escaping from the war live in terrible conditions in the camps. Fuel-stoves and related fires are the most common cause of burns in refugee camps. Therefore, international organizations should find a solution to the heating in camps other than fuel-stoves.

**O2.7.3**
**Burn-Specific Health-Related Quality of Life of Children 5–7 Years after Burns: A National Multicenter Study**
**Marscha Heijblom** ^1^, J.N. Dijkshoorn ^1^, M.E. van Baar ^2,3^, C.H. van der Vlies ^2,4^, I. Spronk ^2,3^
^1 ^ Burn Center, Maasstad Hospital, Rotterdam, The Netherlands,
^2 ^ Association of Dutch Burn Centers, Maasstad Hospital, Rotterdam, The Netherlands,
^3 ^ Erasmus MC, University Medical Center Rotterdam, Department of Public Health, Rotterdam, The Netherlands,
^4 ^ Trauma Research Unit Department of Surgery, Erasmus MC, University Medical Center Rotterdam, Rotterdam, The Netherlands
**Aim:**

To study the long-term burn-specific health-related quality of life (HRQL) of children 5–7 years post-burn and assess factors associated with long-term HRQL.

**Methods:**

Parents of children (5– < 18 years) who were hospitalized or had surgery for their burns between August 2011 and September 2012 completed the Burn Outcomes Questionnaire. HRQL and factors associated with HRQL were studied for the total sample, and outcomes were compared between children with mild/intermediate burns and children with severe burns (>10% total body surface area (%TBSA) burned).

**Results:**

Some 102 children were included (M: 7.4%, SD: 6.4). Many parents rated their child’s health as excellent (46.1%) or very good (35.3%) 5–7 years post-burn. Hardly any children had problems with pain (2.3%), physical function and sports (1.6%), and upper extremity function (0.9%). Parents of children with severe burns indicated significantly more problems with ‘appearance’ (89.2% versus 71.5%; *p* = 0.014) and ‘parental concern’ (94.1% versus 84.8%; *p* = 0.021). Full-thickness burns were related to a lower HRQL in four BOQ domains, whereas the number of surgeries was related to two BOQ domains.

**Conclusions:**

The majority of the children in our sample had a good HRQL 5–7 years post burn. Children with full-thickness burns and those who have undergone surgery have a higher risk of a lower long-term HRQL. These results give children with burns, their parents, and healthcare professionals insights into long-term burn-specific HRQL.

**O2.7.4**
**A Meek Micrografting Technique as a Salvage Surgical Technique for Extensive Burns without Skin Substitutes**
**Dan Mircea Enescu** ^1,2^, Iulia Nacea ^1,2^, Cristina Stoica ^2^, Maria Tomiță ^2^, Raluca Tatar ^1,2^
^1 ^ Carol Davila University of Medicine and Pharmacy, Bucharest, Romania
^2 ^ Grigore Alexandrescu Clinical Emergency Hospital for Children, Bucharest, Romania
**Aims:**

The coverage of burns exceeding 50% TBSA raises many challenges for burn teams, and using the Meek micrografting technique represents a reliable choice for these highly difficult cases.

**Methods:**

The Plastic Reconstructive Surgery and Burns Department of the “Grigore Alexandrescu” Hospital for Children, Bucharest is the national referral center for all major pediatric burns in Romania. The Meek technique has been available in our department since July 2019, so we performed a retrospective review of burn patients admitted from July 2019 till June 2022. The inclusion criteria were the presence of burn wounds and a surgical protocol including this specific procedure.

**Results:**

Considering the inclusion criteria, 29 patients were identified (boys 72.41%). The patients’ age varied between 1year 9 months and 17 years 10 months. The median TBSA was 57.5 ± 19.96% (ranging from 20–90%). The main etiology for this patient group was flame (19 cases), followed by electrical burns and scalds. The patients in the scald group were significantly younger (ranging from 1 year 9 months–3 years 1 month) than in other cases. All of them underwent early and seriate burn wound excision and covering with Meek micrografts at expansion rates from 1:3 to 1:9 in the most severe patients.

**Conclusions:**

Despite not having access to a skin bank or to synthetic skin substitutes, we have managed to treat burn patients successfully with limited donor sites for autografts. The Meek technique proved to be a reliable tool for salvaging massive pediatric burns, allowing early excision of extensive burn wound areas and prompt covering.

**O2.7.5**
**Long-Term Follow-up Results of the Pediatric NexoBrid Enzymatic Debridement RCT**
**Stan Monstrey** ^2^, Henk Hoeksema ^2^, Yaron Shoham ^1^
^1 ^ Soroka University Medical Center, Beer Sheba, Israel
^2 ^ University Hospital Gent, Gent, Belgium
**Aim:**

The aim of this study was to assess the long-term effects of NexoBrid^®^ (NXB) vs. the standard of care (SOC) in children.

**Methods:**

Some 145 children suffering from deep thermal burns were enrolled in a multicenter, multinational, open-label, randomized, controlled phase III study. Briefly, 72 children were randomized to NXB and 73 to SOC debridement methods. Wound care after achieving complete debridement was carried out according to routine methods, at the investigators’ discretion. Patients continued follow up for at least 30 months post wound closure.

**Results:**

The acute stage results reported previously included 12-month MVSS scores of 3.83 for NXB and 4.86 for SOC. The 24-month results corroborate the 12-month results. Scar assessments were performed on 107/145 patients. The mean MVSS score was 3.21 for NexoBrid and 3.8 for SOC, establishing non-inferiority (*p* < 0.0001). The mean POSAS scores were also lower in the NexoBrid group, 22.9 vs. 27.7 in SOC, but not statistically significant (*p* = 0.08). When excluding remote assessments (performed due to COVID-19), NXB patients had a lower mean POSAS total score of 25.3 vs. 33.4 in SOC (*p* = 0.035), and a lower observer score, 11.7 vs. 15.5 (*p* = 0.046). Functionality and quality of life evaluations were comparable between the groups.

**Conclusions:**

NXB was shown to be safe without deleterious effects on wound healing, scarring, functionality, and quality of life in pediatric burns. 

* Funded by MediWound Ltd. and by US Federal funds from the Office of the Assistant Secretary for Preparedness and Response, BARDA, under Contract No. HHSO100201500035C

**O2.7.6**
**Amputation in Burned Children: Experience at a Tertiary Pediatric Burn Center**
**Sabri Demir** ^1^, Elif Emel Erten ^1^, Sarper Muftuogullari ^1^, Yakup Bilal Aydin ^1^, Emrah Senel ^2^
^1 ^ University of Health Sciences, Ankara Bilkent City Hospital, Children Hospital, Department of Pediatric Surgery, Pediatric Burn Center, Ankara, Turkey
^2 ^ Ankara Yildirim Beyazit University, School of Medicine, Department of Pediatric Surgery, Ankara, Türkiye
**Aim:**

We aimed to share our clinical data and experience with amputation among inpatient burned children in our pediatric burn center (PBC).

**Methods:**

Records of patients between January 2005 and March 2023 were reviewed retrospectively. Age, gender, length of stay, total burned surface area, cause of burn, grafting, amputation, and mortality rate were evaluated. Patients who underwent amputation were identified, and their data were compared with patients whose amputation was not performed. *p* < 0.05 was considered significant.

**Results:**

Some 167 amputations were performed in 53 of 2537 patients. The fingers and toes were amputated most frequently, followed by the forearms. The mean age of amputated victims was found to be higher than non-amputated patients (7.50 years vs. 4.45 years; *p* < 0.001), the length of stay at PBC was longer (15.17 vs. 65.60 days; *p* < 0.001), the total burned surface area was larger (29.40% vs. 14.93%, *p* < 0.001), the grafting rate was higher (94.3% vs. 28.7%; *p* < 0.001), and the male ratio was higher (81.1% vs. 60.3; *p* = 0.004). Of the amputated victims, 29 (54.7%) had flame burns, and 22 (43.4) had electrical burns. The mortality rate was 3.8% in the amputation group and 2.4% in the non-amputation group (*p* < 0.001).

**Conclusions:**

Although flame and electrical burns are less common in children compared to scald burns, amputation is performed at much higher rates. In order to prevent these amputations, it is crucial to make the first evaluation immediately, and to perform the necessary fasciotomies and escharotomies before the compartment syndrome develops. In addition, children and parents should be educated about prevention.

**O2.7.7**
**Development of a Value-Based Healthcare Core Outcome Set for Children after Burn Injuries**
**Robin Verwilligen** ^1^, Marjolein van der Vlegel ^2^, Lotte van Dammen ^2^, Paul van Zuijlen ^1,5,6,7^, The National Burn Care, Education and Research Group The Netherlands ^1,3,4^
^1 ^ Burn Center Red Cross Hospital, Beverwijk, The Netherlands
^2 ^ Dutch Burns Foundation, Beverwijk, The Netherlands
^3 ^ Burn Center Maasstad Hospital, Rotterdam, The Netherlands
^4 ^ Burn Center Martini Hospital, Groningen, The Netherlands
^5 ^ Amsterdam UMC location Vrije Universiteit Amsterdam, Department of Plastic Reconstructive and Hand Surgery, Amsterdam, The Netherlands
^6 ^ Amsterdam Movement Sciences (AMS) Institute, Amsterdam UMC, Amsterdam, The Netherlands
^7 ^ Amsterdam UMC location University of Amsterdam, Paediatric Surgical Center, Emma Children’s Hospital, Amsterdam, The Netherlands
**Aim:**

The three Dutch Burn Centers aim to optimize person-centered burn care and improving patient-relevant outcomes by adapting value-based healthcare (VBHC). The transparent measurement and reporting of patients’ relevant outcomes is essential in VBHC, and requires a core set of the most important and relevant outcomes. The aim of our study was to develop a VBHC burns core set for pediatric patients.

**Methods:**

We conducted a two-round modified Delphi study to reach consensus among (parents of) pediatric patients (<18 years old), healthcare professionals, and researchers. In each round, items were selected if 70% of each group considered an item ‘important’. In the first round, a 9-point Likert scale, and in the second round, a yes/no scale was used to rate if items are important.

**Results:**

A total of 62 items were included in the Delphi study. Some 141 participants completed the first round. In this round, 10 items were included in the VBHC burns core set: physical functioning, scar contraction, quality of life, pain, itchiness, sleep quality, mobility of functional areas, mental health, depression, and the possibility of executing hobbies. The second and last Delphi survey was completed by 68 participants, and resulted in the addition of three more items: anxiety, self-confidence, and return to school.

**Conclusions:**

Using the Delphi method, we established a VBHC core outcome set consisting of outcomes and indicators that are important for pediatric burn patients. In the near future, these outcomes, quality indicators, and process indicators will be systemically monitored and analyzed in our care to improve patient relevant outcomes.

**O2.7.8**
**Electrical Burns in Our Pediatric Patients from 2004–2022**
**Peter Lengyel**, Eugen Frišman, Erik Eliáš, Sylvia Hyseniová
Hospital AGEL Košice-Šaca, Košice-Šaca, Lucna 52, Slovakia
**Aim:**

This is a retrospective study of electrical burns in children treated over the last 19 years in the Burns and Reconstructive Surgery Clinic of Košice-Šaca.

**Methods:**

The studied group were patients suffering electrical burns from the age 0–18 years of age, hospitalized between 1.1.2004 and 31.12.2022 in our workplace. If the treatment started in our clinic, in the case of burned children, fluid administration was performed according to the Galveston Shriners Burns Hospital formula. The patients’ age, mechanism of injury, tension of current (up to 1000 Volts equals low-tension, and more than 1000 Volts equals high-tension injury), and the count and type of surgical treatment (amputation or reconstructive surgery) were included in the data collected for study.

**Results:**

Some 27 pediatric patients had low-tension injuries, and 11 had high-tension injuries. The mechanism of injury was direct contact with the source of electrical current in 34 cases, electrical arc (flash) injury in 4 cases, and no cases of lightning injury to children occurred. After necrectomy, we performed 29 skin grafting operations, 8 flap surgeries, and 6 amputations. No case of death occurred in our group of electrically burned children.

**Conclusions:**

Electrical injuries are rare among thermal injuries, but can be of life-threatening severity. The majority of high-tension injuries occur during adolescent age. 

The treatment of electrical injury is based on multidisciplinary cooperation to save the patient, allowing them to recover and maintain their functioning.

**O2.7.9**
**Electrical Burn Injuries in Children: A Report of 30 Cases**
**Amina Karray**, Hana Fredj, Bahija Gasri, Amel Mokline, Amen Messadi
Intensive Care Burn Department, Traumatology and Burn Center, Tunisia, Ariana, Tunisia
**Introduction:**

Electrical burn injuries are rare in the pediatric population. Most often, they are associated with a poor prognosis. Their severity is linked to the risk of life-threatening complications and the severity of functional sequelae. 

The aim of this study was to determine the epidemiological profile of children admitted to our department for electrical burn injuries.

**Methods:**

A retrospective descriptive study was conducted over a period of five years (2018–2022), including all patients admitted for electrical burns. Demographic, clinical, therapeutic, and evolutionary data were collected and analyzed.

**Results:**

A total of 30 children were admitted for electrical burns among 300 children admitted for burns (10%). The average age was 11 years [4–17]. The gender ratio was 14. The majority were victims of high-voltage electrical burn injuries (93% of cases). The most common circumstances were a domestic accident in 21 cases, and a work accident in 7 cases (23%). The accident took place on the roof of the house in 17 cases. The total burn surface area was 20.5% on average. The united burn standard was 32 on average. Rhabdomyolisis was noted in 63% of cases. Ten amputations were performed. The mortality rate was 23%.

**Conclusions:**

Pediatric electrical burn injuries still carry high rates of mortality and limb amputation. Educating parents and children may help to reduce the incidence of this dramatic accidental pathology.

**O2.7.10**
**Paying Attention to the Social Aspects of Child Burns**
**Sabriye Dayi**, Özdecan Bezirci Ödek, Beyza Dede, Selenay Işçimen
University of Health Sciences, Bursa, Turkey
**Aim:**

Medical and social teams work together in child burns to maximize the child’s best interests. This study aims to reveal the results we obtained regarding burns of children, upon which we consulted with social workers; we aim to elucidate how preventive studies can be carried out, and to determine the social aspects of child burns that can be helped.

**Materials and Methods:**

We retrospectively analyzed the records of children who were burned and hospitalized in the last year in our burn center.

**Results:**

Social services were consulted in 72 (26 girls; 46 boys) of 208 children (2 months-14 years)’s cases, in order to reveal the family’s social status, suspicious findings with a history of burns, and possible causes of child abuse and neglect. Tea and hot water burns were prominent. The percentage of burns was between 0.5–56.5%. Of the 136 children not consulted by social services, 13 had an operation requiring a graft. Of the 72 children consulted by social services, 26 required grafts. Two children consulted by social services were given counseling support, three educational support, five health support, two social and economic support, and one identity card. One child was also separated from her family and taken into custody. However, the results of 32 consultations with social workers could not be followed.

**Conclusions:**

The importance of social services is better understood day by day. Positive steps include planning preventive work in child burns and implementing protective decisions for events that may cost the child’s life beyond burn treatment. It should be remembered that inter-institutional communication should be faster and more professional, which will mean greater help for these children.

**O2.7.11**
**The Dermal Matrix and Meek Micrograft Technique for Reconstructive Treatment of Giant Spina Bifida in a Neonate: Case Report**
**Jose Cordova-Orrillo**, Eliana Lopez–Sanchez, Luis Enrique Castaneda–Peralta, Ruth Victoria Lobaton–Rosas
Clinica Delgado Auna, LIMA, Peru
**Aim:**

Dermal substitutes and the Meek micrograft technique are used in the reconstructive treatment of complex and extensive soft-tissue defects. The objectives of this article are to describe the technique and benefits of using the dermal matrix together with the Meek micrograft technique in the reconstruction of a case of giant spina bifida in a neonatal patient.

**Method:**

The patient is a newborn of 6 days old with a weight of 3198 g, who has, due to a giant spina bifida a defect of 8 cm × 7 cm; this was initially treated with a bilayer acellular dermal matrix, associated with a negative pressure system for a period of 4 weeks. Then, the Meek technique was used for autografting on an expansion scale of 2:1. The clinical evolution is described, taking into account the presence of alarm and contingency factors.

**Results:**

The neodermis provided good functional and aesthetic coverage results in the neonate. The use of the tissue expansion system with the Meek technique decreased the morbidity and mortality of the donor area.

**Conclusions:**

The dermal matrix and Meek micrograft system as reconstructive tools for complicated defects in the pediatric population are an important alternative, and safe for consideration.

**Thursday 7 September 2–4 pm**
**Session: Critical Care and Anesthesia**
**O2.8.1**
**Predicting Mortality in Severe Burns: Comparison of Four Mortality Prediction Scores in a Croatian Burn Center**
**Agata Skunca**, Ana Mesic, Dorotea Zagorac, Mirta Ciglar, Goran Sabo
Sestre Milosrdnice University Hospital Center, Zagreb, Croatia
**Aim:**

This study aimed to evaluate and compare the performance of four prognostic scores (Abbreviated Burn Severity Index (ABSI) score, Ryan score, Belgium Outcome Burn Injury (BOBI) score, and revised Baux score (rBaux)) in a Croatian burn center.

**Methods:**

The retrospective study included 120 severely burned patients (89 males, 31 females) with a total body surface area (TBSA) burned ≥ 20% who were admitted to the burn intensive care unit between January 2016 and December 2022. Predicted mortality was calculated using the BOBI score, Ryan score, ABSI, and rBaux score. The relationship between the mortality and prediction scores was estimated using logistic regression. Model performance was assessed using a receiver operating characteristics (ROC) curve.

**Results:**

The mean patient age was 54.67 ± 20.32 years, and the mean TBSA burned was 42.31 ± 19.16, with full-thickness burns present in 101 patients (84.17%). Inhalation injury was detected in 54 patients (45%), with the mortality rate being 48%. All prognostic models had statistically significant discriminating power with an area under the ROC curve (AUROC) of 0.78 (95% CI 0.70–0.86) for rBAUX, 0.77 (95% CI 0.69–0.85) for ABSI, 0.74 (95% CI 0.66–0.82) for Ryan, and 0.73 (95% CI 0.64–0.81) for BOBI. Comparing AUROC between scores showed that rBAUX (*p* = 0.02) had the best discriminating power.

**Conclusions:**

Calculating scores upon arrival can help with early mortality risk assessment and treatment planning. Considering none of the scores showed a high AUROC value, more studies predicting mortality in severely burned patients are needed.

**O2.8.2**
**The Benefits of Nebulized Heparin for Inhalation Injury in Burn Patients**
Naima Habbachi, Hana Fredj, Malek Chroufa, **Amel Mokline**, AmenAllah Messadi
Center de Traumatologie et grands Brûlés de Ben Arous, Ben Arous, Tunisia
**Introduction:**

Smoke inhalation injuries increase overall mortality in burn patients. The contribution of heparin aerosolization remains controversial.

**Aim:**

To assess the contribution of heparin aerosolization in pulmonary burn injury.

**Methods:**

This was a prospective case–control study conducted in the burn unit of Ben Arous from 2018 to 2022. Patients that were intubated for pulmonary burn injury were included. Respiratory injury was assessed using the Murray LIS score. After inclusion, heparin was administered via nebulization for 7 days. The primary endpoints were the occurrence of ARDS and the kinetics of the LIS score and PaO_2_/FiO_2_. The secondary endpoints were duration of invasive mechanical ventilation and occurrence of ventilator-associated pneumonia (VAP). The group (Hep+) was compared with a retrospective group (Hep−) from the same center matched for age, burned skin area, severity scores, initial LIS and PaO_2_/FiO_2_.

**Results:**

During the study period, 25 patients were collected and compared with a control group (n = 23). 

Our study showed that the occurrence of ARDS and the LIS score evolution were comparable between the two groups. Nevertheless, heparin aerosolization improved the PaO_2_/FiO_2_ at H24 and H48.

The duration of mechanical ventilation was comparable.

In the Hep (+) group, the incidence of VAP was 44% (11) vs. 73% (17) in the Hep (−) group with *p* = 0.03.

**Conclusions:**

Our study shows that aerosolized heparin does not prevent the occurrence of ARDS, but allows gas exchange to be improved during the first 48 h. It may reduce the risk of VAP without having an impact on the duration of invasive mechanical ventilation.

**O2.8.3**
**Relieving Burn-Induced Compartment Syndrome via Enzymatic Escharotomy–Debridement: A Case Series**
**Ilaria Mataro**, Carlo Petroccione, Stefano Avvedimento, Romolo Villani, Roberto d’Alessio
Hospital A. Cardarelli, Naples, Italy

Early treatment of circumferential burns of the extremities with a Bromelain-based enzymatic agent NexoBrid^®^ (NXB) may represent a less traumatic and invasive procedure to reduce intra-compartmental pressure, thereby replacing surgical escharotomy.

This case series of 23 patients describes the variation in compartmental pressure in patients with circumferential burns of the extremities treated with Nexobrid.

The primary endpoint of treatment response was the post Nexobrid treatment releasing of pathological pressure to a level <30 mmHg. The second primary endpoint was the time needed to reach a safe compartment pressure less than 30 mmHg.

An additional secondary endpoint was the completeness of the eschar removal from the treated area. 

This study cohort included 23 patients, 15 males and 8 females aged between 19 and 75 years with a mean of 47.08 ± 2.12. All patients had deep circumferential burns with TBSA between 30% and 9%, but without definite clinical signs of BICS (which were excluded and immediately surgically escharotomized). 

Within 2 h of NXB application, the elevated pressures were completely released in all hands with practically normal pressures (<30 mm Hg). An additional small reduction was seen after 4 h (14 mm Hg (ranging from 11 to 16 mm Hg)), which is a reduction of approximately 60% from the initial value. 

This study confirms that Nexobrid is effective in releasing burn-induced compartment syndrome-elevated interstitial compartment pressure to less than 30 mm Hg within 2 h (the primary endpoint). All burns were completely debrided (secondary EP), and no adverse events occurred following NXB application.

**O2.8.4**
**Investigating Changes in Serum Uric Acid Levels as a Predictive Biomarker of Early Acute Kidney Injury in Patients with Severe Burns**
**Sheyda Rimaz**, Zahra Mohtasham-Amiri, Siamak Rimaz, Mohammadreza Mobayen
^1 ^ Burn and Regenerative Medicine Research Center, Guilan University of Medical Sciences, Rasht, Iran
^2 ^ Road Trauma Research Center, Guilan University of Medical Sciences, Rasht, Iran,
^3 ^ Anesthesiology Research Center, Department of Anesthesioly, Guilan University of Medical Sciences, Rasht, Iran
^4 ^ Burn and Regenerative Medicine Research Center, Guilan University of Medical Sciences, Rasht, Iran
**Aim:**

Acute kidney injury (AKI) is a most serious and common complication of severe burns, developing in 0.5–30% cases. We aimed to study the role of serum uric acid (UA) in predicting the occurrence of early AKI.

**Methods:**

This was a prospective observational study. The diagnosis and classification of AKI were performed according to KDIGO criteria. Serum uric acid (UA) and creatinine (Scr), the estimated glomerular filtration rate (eGFR), C-reactive protein (CRP), and Base deficit (BD) were monitored within 48 h after injury in 140 severely burned patients (TBSA = 30–80%).

**Results:**

Within 2 days of burn injury, AKI occurred in 36 of 140 patients (Stage I in 25 cases, Stage II in 10 cases, and Stage III in 1 case). Uric acid, BD, and CRP levels in the AKI patients were significantly higher than the non-AKI patients in the time intervals d0, d1, d2 (*p* < 0.005). A positive correlation was also found between BD and CRP with UA after injury in AKI patients. According to the ROC curves, the UA level (AUC: 0.967, 95% CI: 0.943–0.991) soon after injury (d0), compared with the traditional renal function indicator SCr (AUC: 0.780, 95% CI:0.682–0.879), presented as more valuable for the prediction of early AKI in the early stage of severe burns. In addition, in this study, a serum uric acid level higher than 3.95 mg/dL soon (6–8 h) after injury was a helpful marker for the prediction of the occurrence of early AKI.

**Conclusions:**

The results suggest that the monitoring of UA levels soon after severe burns may be a useful biomarker for predicting the occurrence and progression of early AKI.

**O2.8.5**
**Prognostic Factors in Toxic Epidermal Necrolysis**
Hana Fredj, Amal Aloui, **Amel Mokline**, Sarra Zarrouk, Imen Jemi, Bahija Gasri, Manel Saad, Amen Allah Messadi
Burns Intensive Care Department, Traumatology and Burn Center, Ben Arous, Tunisia
**Aim:**

The aim of our study was to determine the predictive factors of mortality in patients admitted for management of toxic epidermal necrolysis (TEN).

**Methods:**

This was a retrospective and descriptive study conducted within the intensive burn care unit in Tunisia, over a period of 10 years (January 2013–January 2023), including all hospitalized cases of TEN. Demographic, clinical and evolutionary data were collected. Uni and multivariate analyses were performed using SPSS22.

**Results:**

Fifty cases of NET were included. The sex ratio (H/F) was 0.56. The mean age was 41 ± 15 years. The average skin area detached was 38 ± 18.4%. The mucous membrane was affected in all cases. Systemic signs occurred in 74% of cases, especially renal, respiratory, and hematological disorders. The mortality rate was 50% (n = 25). Death was secondary to septic shock in all cases. Age > 43 years (OR: 2.9; CI [1,3,2]; *p* = 0.03; Spc: 0.28; se: 0.85), a detached skin surface area > 39% (OR: 2.18; CI [1.73–4]; *p* = 0.02; Sp:0.12, se:0.84), a delay in resuscitation management > 5.5 days (OR: 4.2; CI: [1.8–3.16]; *p* = 0.04; Sp: 0.23; se: 0.23), the occurrence of infectious complications (OR: 4.1; CI [1.1–4.3], *p* < 0.001), and the use of mechanical ventilation (OR: 6.76; CI [1.04–2.8], *p* = 0.04) were found to be factors of poor prognosis upon multivariate analysis.

**Conclusions:**

NET is associated with high mortality. Early management in intensive care and compliance with asepsis rules could improve its prognosis.

**O2.8.6**
**Lithium Polymer Battery Fire as a New Cause of Polymer Fume Fever**
Charisma Nash, **Peter Berry**, Niall Martin
St Andrew’s Centre for Plastic Surgery and Burns, Broomfield Hospital, Chelmsford, Essex, UK
**Aim:**

The growing popularity of e-bikes and e-scooters has contributed to lithium battery-related fires being the fastest growing fire risk in London; their combustion may cause a unique form of inhalation injury. We discuss an unusual case of a man who experienced physiological derangement following a lithium battery inhalation injury, and review the associated literature.

**Methods:**

A 19-year-old man was admitted with inhalation injury and 15% TBSA burns caused by ignition of an e-bike lithium polymer battery charging in his bedroom. Bronchoscopy revealed erythaema and little soot, but he soon developed severe pyrexia, rigors, hypertension, tachycardia, and lethargy.

**Results:**

The National Poisons Information Service was contacted and suggested that the symptoms were similar to the occupational diseases metal fume fever (MFF) and polymer fume fever (PFF), although PFF is normally reported in the pyrolysis of polytetrafluoroethylene (PTFE)/Teflon^®^. As serum lithium levels remained undetectable, MFF is the less likely cause. The symptoms were managed conservatively and resolved within 48 h.

**Conclusions:**

A further literature review revealed no previous cases that related PFF to lithium battery fires. However, there is evidence that the thermal degradation of lithium polymer batteries releases hydrogen fluoride and phosphoryl fluoride, which are chemicals likely to be responsible for PFF. We therefore conclude this to be the first reported case of PFF caused by the ignition of a lithium polymer battery. With the increasing use of larger lithium batteries in our homes, it is important for those who work in burns services to be aware of this unusual syndrome.

**O2.8.7**
**The Clinical Differentiation of Blood Culture-Positive and -Negative Sepsis in Burn Patients: A Retrospective Cohort Study**
**Dohern Kym**, Jaechul Yoon
Hangang Sacred Heart Hospital, Hallym University Medical Center, Seoul, South Korea
**Aim:**

To investigate clinical differences between blood culture-positive and -negative sepsis in burn patients to improve understanding of sepsis pathophysiology and epidemiology.

**Methods:**

This was a retrospective study of 1643 adult patients (≥18 years) diagnosed with sepsis, divided into two groups based on blood culture results within one week of diagnosis, and admitted to a burn intensive care unit between January 2010 and December 2021.

**Results:**

pH, platelet count, bicarbonate, and haematocrit were significant in both the positive and negative groups. Lymphocyte count and blood urea nitrogen were significant only in the positive group, while lactate dehydrogenase was significant in the negative group. Common Gram-negative bacteria included Acinetobacter baumannii, Pseudomonas aeruginosa, and Klebsiella pneumoniae, while common Gram-positive bacteria were Staphylococcus aureus and S. epidermidis. Carbapenem resistance was associated with unfavorable prognosis in Gram-negative bacteria, except for Pseudomonas aeruginosa.

**Conclusions:**

pH, platelet count, bicarbonate, and haematocrit were routine biomarkers with statistical significance in both groups. Lactate dehydrogenase was significant in the blood-positive group, while red cell distribution width and lymphocyte count were significant in the negative group. Gram-negative bacteria, including Acinetobacter baumannii, Klebsiella pneumoniae, and Pseudomonas aeruginosa, were the most common causes of sepsis. Carbapenem resistance was linked to unfavorable outcomes.

**O2.8.8**
**eMission, an Open-Source Electronic Health Record: an Effort to Improve Quality and Safety in Burnand Reconstructive Surgery Short-Term Medical Missions**
Paul Alfille ^1,2^, **Gennadiy Fuzaylov** ^1,2^, Gennadiy Fuzaylov ^1,2,3^
^1 ^ Massachusetts General Hospital, Boston, MA, USA
^2 ^ Harvard Medical School, Boston, MA, USA
^3 ^ Doctors Collaborating to Help Children, Boston, MA, USA
**Aim:**

We describe a novel electronic health record (EHR), eMission, which is open source, low-resource, easily deployable and robust in remote locations; it also facilitates in-mission patient care and post-mission analysis.

**Methods:**

eMission is a web-based her. Information is stored and accessed locally in the browser for offline usage, and periodically sent, encrypted, to a central database when internet is available for synchronization. On the web application, patients can be identified with a picture, name, or DOB. A print-out is formed for each patient that includes their picture, name, DOB, and a QR barcode.

Information on each patient is split between sections navigated via a home screen that includes demographics, medical history, notes, and operations. Where supported by phones, voice-to-text is available, as well as the uploading of pictures to ease data entry. The web application also allows for post hoc analysis and data archiving without manually searching through individual charts, via export in universal spreadsheet file format.

**Results/Conclusions:**

Short-term medical missions (STMMs) are a common approach to address the lack of access to surgical and anesthesia care in much of the under-resourced world. However, documenting their productivity, safety, and long-term outcomes is a continuing challenge. As a solution, we offer eMission, a program that is smartphone/mobile-based, for use in austere environments, with computer-based data storage for ease of entry and post hoc analysis; it was designed with a simple user friendly interface and patient safety features. This program has been utilized successfully for surgical burn and reconstructive missions in Ukraine, Peru, and Colombia.

**O2.8.9**
**The Association of Platelet Count and Mortality in Severely Burned Adults**
**Ana Mesić**, Agata Škunca, Dorotea Zagorac, Mirta Ciglar, Tihana Magdić Turković
Department of Anesthesiology, Intensive Care and Pain Medicine, Sestre milosrdnice University Hospital Center, Zagreb, Croatia
**Aim:**

To analyze the platelet count in severely burned patients, and its association with mortality.

**Methods:**

Patients eligible for this study were adults with a total body surface area burned of more than 20% who were admitted to the burn intensive care unit from January 2016 to December 2022. Exclusion criteria were electrical injuries, hospital stay < 3 days, and missing data. Some 93 patients were included in the study. Their platelet count was analyzed within 7 days of the injury. Thrombocytopenia was defined as a platelet count of less than 150 × 109/L.

**Results:**

Thrombocytopenia was found in most patients (80%), but it was not associated with mortality. Platelet count on the seventh day after the injury showed a statistically significant difference between deceased and survived patients, as well as the frequently used rBaux score (*p* < 0.0002). Considering that the odds ratio for the rBaux score was estimated to be 1.044 (95% CI of 1.020–1.070), and for platelet count on the seventh day was 0.987 (95% CI of 0.978–0.995), it may be concluded that with an increase of 10 rBaux points, the odds of a lethal outcome are expected to increase by 54%, while with a decrease of platelet count by 10 × 109, the odds of a lethal outcome are expected to increase by 44%. The area under the ROC curve was 0.82.

**Conclusions:**

The platelet count on the seventh day represents a simple and easily accessible test, and when combined with the rBaux score is a strong predictor of mortality.

**O2.8.10**
**Anhydrous Ammonia Burns: Eyes and Lungs Pay a Heavy Price**
Serge Jennes, **Xavier Hoang**, Ghueder Saidane, François Debry
Grand Hôpital De Charleroi, Charleroi, Belgium
**Aim:**

Anhydrous ammonia can cause severe eye and lung injuries, leading to blindness and severe respiratory insufficiency as well as cutaneous burns. We give a quick reminder of the most recent management techniques for these injuries.

**Methods:**

We have reviewed four cases, and the literature.

**Results:**

All four patients were burnt by anhydrous ammonia during their work. All were intubated: three directly in the prehospital setting, and one in the burn wound center after 3 days of high-flow nasal oxygen.

Dysphagia and odynophagia were also present. The patients were ventilated for 7 to 44 days. Two of them showed a prolonged hemorrhagic tracheobronchitis. Their eyes were also severely burnt.

The size of the cutaneous burn wounds varied between 2 and 35% of TBSA, being mainly third-degree burns.

An osteo-odonto-keratoprosthesis was performed on one patient, with a good result for 2 years. A corneal transplantation was performed on other patient with mild success. Autologous plasma was poured into their eyes for the first weeks.

The two other cases had no permanent ocular damage.

One developed a respiratory insufficiency for many weeks, which is still evolving.

**Conclusions:**

Exposure to ammonia results in damage to the skin, eyes and aerodigestive tract. Ocular injuries may result in permanent visual loss. These four cases demonstrate that injury to the respiratory tract is life-threatening, and may cause long-term obstructive lung disease.

**O2.8.11**
**Burns in Pregnancy: An Epidemiological Study of 26 Cases.**
Hana Fredj, Bahija Gasri, **Amel Mokline**, Imen Jemi, Sarra Zarrouk, Manel Saad, Amen Allah Messadi
Burns intensive care department, Traumatology and Burn Center, Ben Arous, Tunisia
**Aim:**

The aim of our study was to determine the epidemiological, clinical, and evolutionary characteristics of burned pregnant women.

**Methods:**

This was a descriptive retrospective study conducted over a period of 15 years (2007–2022), including all burned pregnant women admitted to the Burn Intensive Care Department at a trauma and burn center in Tunisia. Epidemiological, clinical and evolutionary data were collected and analyzed.

**Results:**

Among the 5364 patients admitted, 1517 were female. Some 26 pregnant females were included, representing 7.5% of woman of reproductive age. The mean age was 28 ± 5 years. The term of the pregnancy was greater than 24 weeks in 11 cases. The circumstances of the burns were dominated by domestic accidents (n = 19) followed by suicide attempts (n = 5). They were mainly thermic flame burns (80%). The mean total burned surface area (TBSA) was 32%. Ventilatory support was required in nine cases. The mean length of stay was 9 [2; 42] days. The maternal mortality rate was 31% (n = 8), and the fetal mortality rate was also 31% (n = 8).

**Conclusions:**

The occurrence of burns during pregnancy is associated with high maternal and fetal mortality. Prevention through appropriate education of the pregnant woman is essential.

**Thursday 7 September 2–4 pm**
**Session: Wounds**
**O2.9.1**
**An Implementation Study on Thermal Imaging for Burn Wound Assessment Using the Consolidated Framework for Implementation Research and an Iterative RE-AIM Approach**
**Anouk Pijpe** ^1,2,3^, Jesse de Haan ^1^, Matthea Stoop ^1,2,3^, Paul van Zuijlen ^1,2,3,4,5^

^1^  Association of Dutch Burn Centers, Red Cross Hospital, Beverwijk, The Netherlands
^2^  Amsterdam UMC location Vrije Universiteit Amsterdam, Plastic, Reconstructive and Hand Surgery, Amsterdam, The Netherlands
^3^  Amsterdam Movement Sciences, Tissue Function and Regeneration, Amsterdam, The Netherlands
^4^  Department of Plastic, Reconstructive and Hand Surgery, Red Cross Hospital, Beverwijk, The Netherlands
^5^  Amsterdam UMC location University of Amsterdam, Paediatric Surgical CentreCenter, Emma Children’s Hospital, Amsterdam, The Netherlands

**Aim:**

Thermal imagers provide an accessible diagnostic tool to increase the accuracy of burn wound assessment. This mixed-methods study aimed to assess barriers and facilitators, design implementation strategies, and guide the implementation process of thermal imaging in the outpatient clinic of a burn center.

**Methods:**

This study was conducted between September 2022 and February 2023 in the Red Cross Hospital in Beverwijk-NL. Phase 1 was planning, and included a literature search. In Phase 2, semi-structured interviews guided by the Consolidated Framework for Implementation Research (CFIR) were conducted to identify barriers and facilitators. Based on the barriers, implementation strategies were developed with the CFIR-ERIC Matching Tool, and disseminated to support uptake of the thermal imager. In Phase 3, thermal imaging was implemented in daily practice, and an iterative RE-AIM approach was used to evaluate the implementation process.

**Results:**

Common facilitators included the CFIR constructs of complexity, relative advantage, and needs and resources. Common barriers were knowledge and beliefs, compatibility, and evidence strength and quality. Six implementation strategies were used: creating a formal implementation blueprint, promoting adaptability, developing educational materials, facilitating, conducting ongoing training, and identifying early adopters. Throughout the implementation process, compatibility and available resources remained barriers, resulting in low ratings on RE-AIM dimensions.

**Conclusions:**

This study identified CFIR constructs that impact the implementation of thermal imaging in our outpatient clinic, as well as effective implementation strategies. The findings of this study could be leveraged to guide the implementation of innovations such as thermal imaging in burn care, and to guide improvement of technicalities and usability in clinical practice.

**O2.9.2**
**BTM (Biodegradable Temporising Matrix, Polynovo) Proves Reliable in the Treatment of Large Pyroclastic Burns Following the White Island Volcano Eruption in New Zealand**
**Peter Maitz** ^1,2,3^, Justine O’Hara ^3^, Joanneke Maitz ^1,2,3^
^1 ^ University of Sydney, Sydney, Australia
^2 ^ ANZAC Research Institute, Sydney, Australia
^3 ^ Concord Hospital, Sydney, Australia
**Aim:**

To demonstrate the surgical planning, procedures and results of the use of BTM for pyroclastic burns >40% TBSA. Pyroclastic burns are rare, and combine large thermal burn injuries with chemical and toxic substrate deposition in the wounds and lungs of victims.

**Methods:**

Following the White Island volcano eruption in 2019 in New Zealand, patients with Australian nationality were repatriated to Sydney after intubation and stabilisation. Information exchange with our New Zealand colleagues and a literature search revealed similar surgical planning guidelines as for large thermal burns, with an emphasis on extreme aggressive eschar debridement.

Total eschar debridement of all burn wounds (except burned digits) was achieved in a single procedure in all patients, and all wounds were immediately covered with BTM and Acticoat dressings; no skin grafting was undertaken in the first procedure. All patients improved within the first 24 h following total eschar removal in terms of haemodynamics and ventilator requirements. Debridement and skin grafting of all burned digits was undertaken in the following days. After integration of BTM and neo dermis formation between weeks 2 and 3, staged skin grafting was undertaken.

**Results:**

BTM integrated in all patients, and delamination of the bi-layered BTM and skin grafting could be undertaken after 2 weeks. All skin grafts healed with no revision, and all patients were discharged within 70 days.

**Conclusions:**

BTM proved easy to use, reliable to integrate, and resulted in pliable reconstruction of full-thickness pyroclastic burn wounds.

**O2.9.3**
**A Randomized Controlled Clinical Comparison of Three Wound Dressings**
**David Lumenta** ^1^, Andrzej Hecker ^1^, Birgit Michelitsch ^1^, Herwig Friedl ^2^, Lars Peter Kamolz
^1 ^ Medical University of Graz, Graz, Austria
^2 ^ Graz University of Technology, Graz, Austria
**Aim:**

To compare hydroactive nanocellulose-based, silver-impregnated, and ibuprofen-containing foam wound dressings on split-thickness skin graft donor sites.

**Methods:**

We included 46 patients scheduled for elective surgery, and the assessed criteria were wound healing (time-to-healing, Hollander Wound Evaluation Scale), pain level (Visual Analogue Scale), handling (ease of use), and scar quality (Patient Scar Assessment Scale, Vancouver Scar Scale) after 3, 6, and 12 months.

**Results:**

All dressings fared well (wound healing), with only minor statistically but less clinically relevant differences for pain level favoring the ibuprofen-containing dressing (*p* = 0.002, deltaAIC = 8.1), and user friendliness favoring the nanocellulose-based dressing. Hydroactive nanocellulose and the ibuprofen-containing foam revealed statistically relevant better scar appearances compared to the silver wound dressing (*p* < 0.001, deltaAIC = 14.77).

**Conclusions:**

The dressings performed equally well on all included patients, with the detected statistical differences hinting at future directions of clinical relevance.

**O2.9.4**
**Rapid Vascularized Collagen–Elastin Matrix MatriDerm® Offers Multiple New Surgical Options in Burns and Trauma**
**Markus Öhlbauer**, Britta Wallner
BG Trauma Center Murnau, Murnau, Germany

Despite successful defect coverage by complex flaps, wounds with exposed bradytrophic tissues are particularly highly susceptible to surgical revision. This has led to the development of dermal matrices in order to improve the quality of reconstructed tissue.

MatriDerm^®^, a matrix of collagen and elastin, was first used in 1 mm thickness in one-stage and in 2 mm in two-stage procedures. Due to the rapid vascularization of this matrix, more and more former 2 mm two-stage procedures, it has been shown, could be performed in one stage. Since 2021, MatriDerm^®^ has even been available in 3 mm thickness.

A total of 93 patients treated between 2014 and 2020 for severe soft-tissue defects using STSG in combination with MatriDerm^®^ in one- or two-stage procedures were included in a retrospective study. The defects’ healing was measured via assessment of the take rate. The outcome quality of the scar tissue was assessed using the Vancouver Scar Scale more than 24 months postoperatively.

The overall healing rate (take rate ≥ 75%) was 84%. The majority of postoperative events included healing disturbances such as remaining defects, necrosis, or delayed healing.

A two-year follow-up of these procedures showed an excellent functional outcome; no areas with unstable scars were present, and no surgical scar revisions were required. The patients were still able to wear normal footwear, and a clinical gait analysis showed perfect functional outcome.

The application of the collagen–elastin matrix MatriDerm^®^ to patients with exposed bradytrophic tissue after severe burns or traumatic injuries can be recommended as an excellent reconstruction method, independent of one- or two-stage procedures.

**O2.9.5**
**Balancing Regenerative Medical Approaches and Flap Surgery in the Treatment of Burn Injuries and Their Sequelae**
**Bong-sung Kim**, Carlotta Barbon, Lisanne Grünherz, Nicole Lindenblatt, Pietro Giovanoli
Department of Plastic Surgery and Hand Surgery, University Hospital Zurich, Zurich, Switzerland
**Aim:**

Regenerative medicine offers legitimate solutions in plastic and reconstructive surgery. The treatment of thermally inflicted wounds in particular may benefit from a less invasive regimen. Herein, the value of classic reparative techniques including flap surgery, and the merits of regenerative medicine for acute injuries and post-injury sequelae are discussed.

**Methods:**

Acute burn injuries (n = 8) were treated with isolated fat-derived stromal cells and growth factors upon enzymatic debridement, and the clinical outcome was evaluated. Furthermore, the use of various regenerative dermal substitutes in the treatment of burn scars was investigated (n = 30). Outcomes were compared with the standard treatment, which consists of skin grafts for acute injuries and flap surgery for burn scar reconstruction.

**Results:**

The use of fat-derived stromal cells and growth factors still does not provide stable outcomes, and often results in re-excision and skin grafting. No specific confounders for therapy failures were identified. Dermal substitutes showed favorable vascularization and granulation in static areas. For the reconstruction of joints the neck, however, the failure rate was high. Here, thin perforator flaps areas were utilized.

**Conclusions:**

Based on our current experience, no reliable regenerative medical alternatives to skin grafting for the treatment of deep acute burn injuries are accessible. Dermal substitutes offer feasible solutions for static areas and can act in salvage situations. For critical areas including joints and the neck, thin perforator flaps are the mainstay in the treatment course. In the future, advanced tissue engineering concepts such as epidermal–dermal constructs and other composite solutions may render novel opportunities.

**O2.9.6**
**An Innovated Elastic Compression Hemostasis Technique for Extremity Excision in Patients with Extensive Burns: A Prospective Clinical Randomized Controlled Trial**
**Chuanan Shen**, Xinzhu Liu, Bohan Zhang, Huageng Yuan
Chinese People’s Liberation Army General Hospital, Beijing, China
**Aim:**

To introduce an innovated elastic compression hemostasis technique for extremity excision in extensively burnt patients and investigate its effectiveness.

**Methods:**

A total of 10 patients were included and divided into two groups: the control group (4 patients, 12 extremities), receiving the conventional hemostasis technique; and the experimental group (6 patients, 14 extremities), receiving the innovated technique. General data of the patients were collected, excision sizes measured, hemostasis times recorded, average blood losses per 1%TBSA of the excised wound calculated, and incidence of subcutaneous hematoma and take rate determined.

**Results:**

There was no statistical difference in the baseline data between the two groups. The average blood losses per 1%TBSA of the excised wound in the upper and the lower extremities were (62.1 ± 11.5) mL and (35.6 ± 11.0) mL in the experimental group, significantly less than (94.3 ± 6.9) mL and (82.3 ± 6.2) mL in the control group; this represents a reduction of 34.1% and 56.8%, respectively. The hemostasis times in the upper and the lower extremities were (5.0 ± 0.7) min/1%TBSA and (2.6 ± 0.3) min/1%TBSA, respectively, in the experimental group, which is significantly less than (7.4 ± 0.6) min/1%TBSA and (4.0 ± 0.9) min/1%TBSA, in the control group; this represents a reduction of 31.8% and 34.9%, respectively. The incidence of subcutaneous hematoma was 7.1% and 8.3%, and the take rate (85.9 ± 6.0)% and (86.5 ± 4.8)% in the experimental and the control group, respectively, with no statistically significant differences.

**Conclusions:**

The innovated elastic compression hemostasis technique is a reliable new method that significantly reduces blood loss during extremity excision in patients with extensive burns, and is worth wider understanding and application.

**O2.9.7**
**The Use of Biostatic Human Amnion and Platelet-Rich Plasma in the Topical Treatment of Toxic Epidermal Necrolysis-A Case Report**
**Przemysław Strzelec**, Karolina Ziółkowska, Agnieszka Klama-Baryła, Wojciech Łabuś, Artur Wielgórecki
Dr. Stanisław Sakiel Center For Burn Treatment In Siemianowice Śląskie, Siemianowice Śląskie, Poland
**Aim:**

Toxic epidermal necrolysis (TEN) (also known as Lyell syndrome) and Stevens-Johnson syndrome (SJS) are life-threatening mucocutaneous blistering diseases. They are characterized by generalized blisters and epidermal inflammation, most likely resulting from the administration or interaction of medicines. The aim of this work was to report a potential new method for the treatment of TEN.

**Methods:**

This article presents a case report of a 35-year-old man suffering from TEN covering about 95% of his body surface. Lesions occurred in the patient during antiepileptic therapy, after simultaneously taking amoxicillin (with clavulanic acid) and naproxen, followed by lamotrigine treatment. Standard general treatment was performed. Intravenous feeding was necessary. Due to acute respiratory failure, the patient required mechanical ventilation. Two methods were combined in the topical treatment: the application of platelet-rich plasma (PRP) and a simultaneous biostatic human amnion transplant.

**Results:**

In the presented case, the combination of both methods contributed to a significant acceleration of wound healing. After the application of PRP and biostatic amnion transplantation, the healing of wounds on the back and posterior surfaces of the legs was completed after six days. The surgical treatment most probably contributed to a significant acceleration of wound healing.

**Conclusions:**

The case report shows that topical TEN/SJS treatment with biostatic human amnion and PRP has a positive clinical effect, and may be a new method for the treatment of TEN.

**O2.9.8**
**Comparison of Polyhexanide versus Enzyme Alginogel Wound Dressing: a Retrospective Study**
**Karel Claes**, Ignace De Decker, Petra De Coninck, Kim De Mey, Stan Monstrey
Ghent University Hospital, Ghent, Belgium
**Aim:**

The purpose of this study was to compare the bacterial load and healing time of burn patients treated with Prontosan^®^ Wound Gel X (WGX) with patients treated with our standard-of-care (Flaminal^®^).

**Methods:**

All patients with one or more region of interest (ROI), determined using laser Doppler imaging (LDI) and treated with WGX were compared with a similar group of patients treated in the same period (June 2019–July 2020) with Flaminal^®^. The ROI corresponded to different healing potentials (HP). The standard-of-care wound swabs were taken regularly.

**Results:**

A total of 30 patients (67 ROI) were treated with WGX. These ROI were compared with 31 patients (93 ROI) treated with Flaminal^®^. The distribution of HP categories (HP < 14 d, HP14–21 d and HP > 21 d) in both groups was comparable (*p* = 0.733).

There was a significantly (*p* < 0.001) reduced number of positive swabs for P. Aeruginosa in the WGX group (5 vs. 29; 7.46% vs. 31.18%). Additionally there was a significantly (*p* < 0.01) reduced number of positive swabs in the WGX group for Staph. Aureus (5 vs. 29; 7.46% vs. 31.18%). None of the patients suffered from wound infections.

In the subgroup analysis of conservatively treated patients (HP > 21 d; 35 WGX and 45 Flaminal^®^), there was no statistically significant difference in healing time (mean 22.18 ± 12.05 d vs. 19.13 ± 6.06 d).

**Conclusions:**

This retrospective study confirms the broad spectrum of activity of WGX against Gram-positive and Gram-negative bacteria. Although the incidence of PA and SA was lower in the WGX group, a significant difference in healing time could not be shown.

**O2.9.9**
**Closing the Gap: Healing Acute Complex Wounds Using an Acellular Dermal Substitute**
**Milly Van De Warenburg**, Mariëlle Vehmeijer, Tim De Jong, Stefan Hummelink, Dietmar Ulrich
Radboudumc, Nijmegen, Nederland
**Aim:**

To assess the use of Glyaderm on full-thickness skin defects of different etiologies.

**Methods:**

In this prospective case series, 28 adult patients with acute complex deep soft-tissue defects resulting from different etiologies were treated with Glyaderm, an acellular dermal substitute [1]. Glyaderm is applied in either a one- or two-stage procedure with a split-thickness skin graft for epidermal coverage. The primary outcomes will be the graft take as a percentage of the total covered wound area, and time to complete wound closure.

**Preliminary results:**

Currently, 14 patients have been included and have completed the follow-up period. The mean age of the included population is 59 years (28–83). The etiologies of the acute complex wounds consist of defects after oncological surgery (28.6%), debridement of fasciitis necroticans (14.3%), donor sites after free flap reconstruction (28.6%), trauma (21.4%), and debridement of osteomyelitis (7.1%). The mean affected TBSA was 1.7% (0.2–12%).

Some 5–7 days after application of Glyaderm, a mean take rate of 87.4% (0–100) was observed. The mean time for the wounds to close was 35 days (6–98 days). In 85.7% (n = 12) of the included cases, no complications were observed. In one case (7.1%), an infection occurred after application of Glyaderm, leading to complete loss of the graft. In one case (7.1%), complete loss of the graft occurred because of dehiscence of a free flap close to the wound area.

**Conclusions:**

Glyaderm may prove to be a valid and easy-to-use reconstructive option for defects not suitable for immediate skin grafting.

**O2.9.10**
**Can Fasciotomy Prevent Amputation of a Burned Limb?**
**Nour Zaineb Jaafar** ^1,2^, Hana Fredj ^1,2^, Manel Ben Saad ^1,2^, Amel Mokline ^1,2^, Amenallah Messadi ^1,2^
^1 ^ Faculty of Medicine of Tunis, University Tunis El Manar, tunis, tunisia, Tunis, Tunisia
^2 ^ Intensive care burn department, Traumatology and burn center, Tunisia, Ben Arous, Tunisie
**Background:**

The management of thermal and electrical circumferential burns of the limbs often requires the use of fasciotomy in the hope of saving the limb.

**Aim:**

Our study aims to determine the incidence of fasciotomy in burn patients and to assess its impact on limb survival.

**Methods:**

This was a retrospective study over a two years period (2021–2022). Burned patients who had a fasciotomy were included. Were collected epidemiological and clinical data concerning the nature burns, the site, the delay of the fasciotomy, and the outcome of the limb.

A comparison of patients with thermal burns (G1) was made with patients with high-voltage electrical burns (HVEB) (G2).

**Results:**

A total of 654 patients were enrolled. Briefly, 33 patients had at least one fasciotomy (incidence of 5%). The median age was 33 years, with a gender ratio of 5.6. The median time to admission after burns was 16 h. The circumstances were dominated by domestic accidents (n = 13) and occupational accidents (n = 8). The median total body surface area (TBSA) was 29.5%. G1 included 16 patients versus 17 patients in G2. The most affected limbs were the upper limbs. The progression to limb amputation was significantly higher (n = 0 in G1 versus n = 10 in G2, *p* < 0.001). For G2, eight patients had an early fasciotomy, of which four patients had later amputations. The median length of stay was higher in G2 (21 vs. 13 days, *p* < 0.05).

**Conclusions:**

Fasciotomy in thermal burns saved the limb in all cases. In the case of electrical burns, it saved the limb in only 40% of cases.

**O2.9.11**
**Clinical Experience of Omega 3 Fish Grafts in Full-Thickness Wounds**
**Ariel Aballay**, Jason Bregg, Nurse Mariah Sturges, Nurse Brianna Bell
Allegheny Health Network, Pittsburgh, United States
**Aim:**

Fish skin grafts (FSGs) have been advocated in wound care to support epithelialization and healing by secondary intention. FSG is a relatively new and promising matrix with versatility in managing varying clinical treatment goals. FSG is minimally processed, has the utility to augment cell migration and neovascularization, and is rich in Omega3 fatty acids. Another proposed indication for this xenograft is as bridge therapy when dealing with full-thickness wounds by decreasing the defect’s size and supporting wound bed preparation for closure. Therefore, this case series primarily aims to investigate FSG as a bridge therapy for full-thickness wounds associated with burns, trauma, and surgical wounds.

**Methods:**

In this case series, several patients of varying ages and ethnicities with full-thickness wounds of different sizes related to burns, trauma, and surgical wounds had FSG applied to minimize the depth of deep defects. The changes observed in wound characteristics, depth, infection rate, and graft take were evaluated and tracked.

**Results:**

Overall, in the cohort of patients treated with FSG, wound appearance was positively affected without an increase in infection rate, autograft loss, or other complications, and were advanced to closure/next therapy within 2 weeks on average.

**Conclusions:**

In this case series, the usage of the fish skin graft facilitated the management of these wounds and proved to be autograft-sparing, effectively decreasing the size of the wound defects in a short period of time. This lead to a decrease in length of stay. The results validate using FSG in clinical practice when managing these challenging wounds.

**Friday 8 September 8–9:50 am**
**Session: Pain Management, Psychosocial and Psychiatry**
**O3.1.1**
**Predictors of the Long-Term Quality of Life of Paediatric Patients after Non-Severe Burn Injuries**
**Amira Allahham** ^1,2^, Mark Fear ^1,2^, Fiona Wood ^1,2,3^, Lisa Martin ^1,2,4^, Matthew Cooper ^4^

^1^  University of Western Australia, Perth, Australia
^2^  Fiona Wood Foundation, Perth, Australia
^3^  Burn Service of Western Australia, Perth, Australia
^4^  Perth Children’s Hospital, Perth, Australia
^5^  Telethon Kids Institute, Perth, Australia
**Aim:**

To investigate the epidemiology of paediatric burn injuries in Western Australia and determine which demographic and clinical factors correlate with low quality of life during recovery, and to assess the differences in patients and parents’ scoring of psychosocial function.

**Methods:**

This was a retrospective cohort of paediatric patients with non-severe burns who were assessed using the paediatric quality of life survey (PedsQL). PedsQLs consist of a parent report and a patient report, with a physical function domain (PF) and a psychosocial function domain (PSF). Demographic and clinical data were also collected from the patients.

**Results:**

Regarding the first aim, parents’ report scores were significantly different between age groups (PF: *p* = 0.002, PSF: *p* = 0.001, respectively), burn cause (PF: *p* = 0.004, PSF: *p* = 0.005, respectively), and socioeconomic status groups for the PSF (patient: *p* = 0.015, parent: *p* = 0.032, respectively), and 16.46% of paediatric burn patients had critically low quality-of-life scores. The second aim showed that during early recovery, parents reported poorer PSF for younger children (*p* = 0.01), higher socioeconomic status (*p* = 0.05), and significantly different scores for female patients (*p* < 0.01). In the late recovery cohort, only the age at burn had an effect, where parents had lower scores for older patients (*p* = 0.03).

**Conclusions:**

These data will allow health professionals to accurately assess patients’ quality of life to provide them with services that can aid in their recovery.

**O3.1.2**
**Developing and Piloting Palliative Care Practice Recommendations in the Burn Intensive Care Unit: A Quality Improvement Project**
**Jonathan Bayuo** ^1^, Kyei Baffour
The Hong Kong Polytechnic University, Hung Hom, Hong Kong
**Aim:**

To develop and pilot palliative care practice recommendations in the burns intensive care unit of a tertiary healthcare facility.

**Methods:**

A four-phased multi-method design was employed: (1) a scoping review to ascertain the role of palliative care in burns management; (2) post-bereavement interviews with family members and burn care staff; (3) consultative meeting with palliative and burn care practitioners to formulate practice recommendations; and (4) piloting the practice recommendations using a prospective observational approach. For the pilot phase, a nurse/research assistant was assigned to undertake participant observation following staff training on the practice recommendations. A checklist based on the practice recommendations was created to enable the nurse to record observations from January 2020 to December 2022.

**Results:**

Palliative care practice recommendations in the burn unit should focus on (1) shared decision-making and communication; (2) role support for family caregivers; (3) holistic symptom management at the end of life; (4) bereavement and post-bereavement support for family members; and (5) post-bereavement support for burn care staff. In the pilot phase, it was observed that of the 170 burn patients admitted, 66 persons died. Although several aspects of the practice recommendations were observed, post-bereavement support, symptom management at the end of life, and collaboration across teams are still limited.

**Conclusions:**

There is still room to improve the integration of palliative care in our burn unit. Particularly, there is a need for ongoing staff training and collaboration with palliative care staff.

**O3.1.3**
**Partners’ Social Support is Associated with Depressive Symptoms in Burn Survivors**
Nora de la Parra ^1^, **Helma Hofland** ^2^, Nancy Van Loey ^2^
^1 ^ Red Cross Hospital, Beverwijk, The Netherlands
^2 ^ VSBN Maasstadziekenhuis, Rotterdam, The Netherlands

The aim of this study was to investigate the influence of the partner’s social support on depressive symptoms. This multi-center study included 158 adult burn survivors with a partner. Burn survivors completed the Illness Invalidation Questionnaire, including two subscales (“discounting behaviour” and “lack of understanding”), and Beck’s Depression Inventory at 3 and 6 months post-burn. Linear regression was used to analyse the data. Depressive symptoms 6 months post-burn were significantly associated with discounting behaviour (*p* = 0.001), lack of understanding (*p* = 0.017), TBSA burned (*p* = 0.04), and gender (*p*= 0.002). When controlling for early depression, lack of understanding was the only significant predictor (*p* = 0.03). In conclusion, partners’ discounting behaviour was associated with both early depressive symptoms, suggesting these may be (stable) patterns. Lack of understanding was a risk factor for the maintenance of depressive symptoms, indicating that promoting an understanding attitude may yield beneficial effects on depressive symptoms in the aftermath of a burn injury. This study indicates that early intervention is important, specifically targeting partners with education on how to support their partner.

**O3.1.4**
**Life Satisfaction after Burns**
Maria Fernanda Hutter, **Christian Smolle**, Lars-Peter Kamolz
Medical University of Graz, Graz, Austria
**Objectives:**

Besides health-related quality of life, life satisfaction is also an important outcome parameter for evaluating the long-term consequences of burn injuries for a patient’s life. We conducted a systematic review on life satisfaction after burn injuries among adult burn victims in order to evaluate the currently-used assessment methods, and gain an insight into recovery patterns.

**Methods:**

PubMed, EMBASE, Medline, and Cochrane Library were searched systematically for studies covering life satisfaction after burn injuries among adult burn victims. The screening resulted in the inclusion of 18 studies.

**Results:**

The Satisfaction With Life Scale (SWLS) was the most commonly used assessment tool. Others included The Life Satisfaction Index A (LSI-A) and a non-standardized tool. Recovery patterns varied between studies, and studies looked at several different potential influencing factors.

**Conclusions and implications of key findings:**

Life satisfaction is an outcome parameter that is increasingly used to assess mental wellbeing after burn injury. There is a predominant consensus on the assessment tools. This opens up the possibility of further comparative investigation in the future to better understand factors that influence life satisfaction after burns; this potential knowledge can be used to improve patients’ recovery.

**O3.1.5**
**Self-Harm in Burn Patients Based on Registers and in Medical Records in Finland, 2011–2020**
**Raimo Palmu** ^1^, Lotta Purola, Jyrki Vuola
^1 ^ Helsinki University Hospital, Helsinki, Finland
^2 ^ Oulu University Hospital, Oulu, Finland
^3 ^ Finnish Institute for Health and Welfare, Helsinki, Finland
**Aim:**

To collect data of self-harm burn patients on a national level in Finland and analyze their characteristics.

**Methods:**

First, we went through The National Care Register for Health Care (Hilmo) from 2011 to 2015 to find all patients in Finland having both burn and self-harm ICD10 codes. Then, we investigated the medical records of all patients treated in the National Burn Center (NBC) in Helsinki 2011–2020. The patients admitted to hospital because of self-harm burn injuries were compared to those without self-harm injuries.

**Results:**

The Hilmo register consisted of 3391 adult burn patients admitted to any health care unit during the study period. Compared with non-self-harm patients, self-harm burn patients (N = 82) had a lower mean age and longer hospitalization. According to medical records, self-harm patients (N = 39) admitted to NBC in 2011–2020 had a pre-burn history of psychiatric care, and one-third of them previous self-immolation. Men had more severe burns than women (mean TBSA 46% vs. 14%), and six men died during the first 48 h of care (no female patients died).

**Conclusions:**

Register-based, self-harm burn patients were younger and had longer hospitalizations than other burn patients at all care levels. In the medical records of hospitalized self-harm burn patients, we found clear gender differences in the severity of the burn injury and mortality. Recognizing high-risk patients pre-burn could have a strong preventive impact.

**O3.1.6**
**Lidocaine Infusion Has a 25% Opioid-Sparing Effect on Background Pain after Burns: A Prospective, Randomised, Double-Blind, Controlled Trial**
**Islam Abdelrahman**, Ingrid Steinvall, Moustafa Elmasry, Prof Folke Sjöberg
Linköping University Hospital, Linköping, Sweden
**Title:**

The pain of a burn mainly results from the inflammatory cascade that is induced by the injured tissue, and is classified as background, breakthrough, procedural, and postoperative pain. High doses of opioids are usually needed to treat background pain, so its management includes a combination of types of analgesia to reduce the side effects. Lidocaine given intravenously has been shown in two small, uncontrolled studies to have an appreciable effect on pain after burns.

**Aim:**

This prospective double-blind controlled trial was designed to examine and quantitate the opioid-sparing effect of a continuous lidocaine infusion for the treatment of background pain in burns.

**Methods:**

Adult patients injured with burns of >10 total body surface area burned (TBSA%) and treated with a morphine-based (PCA) approach were randomised to have either a lidocaine infusion starting with a bolus dose followed by continuous infusion, or a placebo infusion, for seven consecutive days. The total daily consumption of opioids and amount of pain (visual analogue score, VAS) were recorded.

**Results:**

We included 19 patients, 10 of whom were given a lidocaine infusion. There were no differences between groups in VAS, TBSA%, time of enrolment to the study since the initial burn, or duration of hospital stay. The opioid consumption in the lidocaine group declined by roughly 25% during the period of the study.

**Conclusions:**

Intravenous infusion of lidocaine was safe and had a 25% opioid0sparing effect in burns when used to treat background pain.

**O3.1.7**
**Optimism and the Extent of Pain during Hospital Admission Predict Pain after Discharge**
**Chloé Balland** ^1^, Nancy van Loey ^2^, Helma Hofland ^2^, Berno van Meijel ^3^, RN, Alette de Jong ^1^
^1 ^ Rode Kruis Ziekenhuis, Beverwijk, The Netherlands
^2 ^ Maasstad Ziekenhuis, Rotterdam, The Netherlands
^3 ^ UMC Amsterdam-VUmc, Amsterdam, The Netherlands
**Aim:**

The aim of this study was to investigate if in-hospital background pain, procedural pain, pain-related anxiety, optimism and posttraumatic stress symptoms predict pain after discharge.

**Methods:**

The study was a multi-center prospective longitudinal cohort study of adults admitted to five burns centers. Pain after discharge was measured over 14 consecutive days. Burn survivors reported overall pain per day on a numeric rating scale (NRS). The mean sum score of all available measurements was used, which was dichotomised (cut-off point > 2 indicating pain after discharge). Two logistic regression analyses were used to test predictors of pain after discharge, one using mean procedural pain (PP) and one using mean background pain (BP) during admission.

**Results:**

The results showed that for the model using BP, burn survivors reporting pain after discharge had a higher BP during admission (*p* < 0.001). In the model including PP, burn survivors reporting pain after discharge had higher PP and scored lower on optimism (respectively, *p* = 0.003 and *p* = 0.02). Background pain showed a stronger effect on post-discharge pain compared to procedural pain.

**Conclusions:**

The results showed that PP, BP, and optimism predict the amount of post-discharge pain. This study suggests that patients with higher pain scores in the hospital may need specific attention regarding pain after discharge, and they may benefit from optimism-inducing interventions.

**O3.1.8**
**Long-Term Mortality after Self-Inflicted Burns**
**Laura Pompermaier**, Ingrid Steinvall, Moustafa Elmasry, Islam Abdelrahman, Folke Sjoberg
Department of Hand Surgery, Plastic Surgery and Burns, Linköping University, Sweden, Linköping, Sweden
**Aim:**

Self-inflicted burns are a small but consistent group among burn patients, with large injuries and conflicting findings regarding their in-hospital mortality. Overall, burn survivors have a shorter life expectancy compared with national controls, but long-term mortality after self-inflicted burns is understudied. The aim of this retrospective study was to investigate possible differences in long-term mortality among survivors after self-inflicted and accidental burns.

**Methods:**

All patients with burns admitted at the Linköping Burn Center and discharged alive between 2000 and 2017 were included, and the end of follow-up was the 26th of April 2021. Those with unknown survival status at that time were excluded. A Cox proportional hazard regression was used to analyse long term mortality adjusted for age and sex.

**Results:**

Among the 1462 patients included in this study, 38 were self-inflicted burns. Overall, the median follow up period was 9.1 years, and crude mortality was 16.1%. The risk-adjusted mortality analysis showed that self-inflicted burns were a factor independently associated with long-term mortality, with a hazard ratio of 2.04 (95% CI 1.11–3.76). Post hoc analysis showed that the effect was most pronounced during the first years after discharge, although it was noticeable over the whole study period.

**Conclusions:**

Long-term risk of mortality after discharge from a burn center was higher in the subgroup of self-inflicted burns compared with accidental burns. The effect was noticeable over the whole study period, although it was most pronounced during the first years after discharge.

**Friday 8 September 8–9:50 am**
**Session: Scars**
**O3.2.1**
**Enhancing Scar Treatment Outcomes with Modified Laser Treatment Approaches: A Study on Hypertrophic and Atrophic Burn Scars**
**Jung hwan Lee**, Yi Jeong Kim
Hangangsoo Plastic Surgery, Seoul, Republic of Korea

Fractional CO_2_ laser treatments provide a safe and effective treatment for scars. The objective of this study is to validate the influence of altering the laser treatment approach on the outcomes of atrophic and hypertrophic burn scars.

This study used the Ultrapulse (Lumenis Ltd., Yokneam, Israel) laser for scar treatment. Hypertrophic scars were treated with high energy and low density in SCAAR Fx mode, while atrophic scars were treated with low energy and high density in Deep Fx mode. Two groups of 40 patients with hypertrophic burns scars were treated, with Group 1 receiving treatment with a continuous wave CO_2_ laser without depth limitation, followed by SCAAR Fx mode, and Group 2 treated only with SCAAR Fx single mode. Twenty patients with atrophic burn scars were treated in two groups, with Group A undergoing 100% overlapping for increased resurfacing area and receiving two passes in Deep Fx mode, and Group B receiving only one pass in Deep Fx mode.

The results showed that Group 1 had significant improvement in scar vascularity, pliability, and height compared to Group 2. Group A also showed more improvement in scar appearance compared to Group B. Patient-reported outcomes revealed better results in scar appearance for both Group 1 and Group A compared to single mode treatment.

In conclusion, combination laser treatment in hypertrophic burn scars and increasing the laser resurfacing area in atrophic burn scars resulted in effective scar height reduction and significant clinical improvement. Modifying laser treatment methods has the potential to enhance treatment outcomes for scars.

**O3.2.2**
**COLOURFUL; Biopsychosocial Post-Burn Follow-Up of Scars in Children: Lessons Learned, Sharing Experience, and Future Plans**
**Jill Meirte** ^1,2^, Mieke Anthonissen ^1,2,3^, Peter Moortgat ^1^, Tibeau Demarbaix ^1^, Ulrike Van Daele ^1,2^, Lieve De Cuyper ^4^, Cindy Lafaire ^4^, Koen Maertens ^1,5^
^1 ^ Oscare, Scar After-care and Research Center, Antwerp, Belgium
^2 ^ Department of Rehabilitation Sciences & Physiotherapy, Research group MOVANT, University of Antwerp, Antwerp, Belgium
^3 ^ Department of Rehabilitation Sciences, University of Leuven, Leuven, Belgium
^4 ^ ZNA Antwerp, Burn Center, Antwerp, Belgium, Antwerp, Belgium
^5 ^ Vrije Universiteit Brussel, Clinical and Lifespan Psychology, Brussels, Belgium, Brussel, Belgium
**Aim:**

To evaluate the long-term effects on the functioning and disability of children with burn injuries, alongside the psychosocial effects that could impair their quality of life.

Gaining insight into functioning and disability may be a challenge, especially when looking at these aspects over several years. Their multidisciplinary care needs to change over time, and children, although resilient, may face a very long rehabilitation period, with changing treatment goals and priorities.

**Methods:**

We have been following up on children and their parents post-burn. In an outpatient setting, children with their parents are invited for a yearly check-up with objective and subjective scar assessment, functional evaluation, and self-reported questionnaires to gain insight into how their functioning, quality of life and disability evolves after their burn injuries and throughout their childhood. We evaluate changes and try to address changing care needs.

**Results:**

Over the years, our approach has changed, and has moved from a limited objective evaluation to a more holistic biopsychosocial coverage of outcome measures, with inclusion of digital questionnaires and choosing more feasible, valid, and reliable scar assessments for our specific population. We keep striving towards a more patient-led, tailored, and holistic approach, with the best possible outcome for every child with unaltered potential and optimal functioning and quality of life.

**Discussion:**

Scar prevention or treatment and patient follow-up after discharge are essential. The follow-up of children post-burn requires teamwork, and a biopsychosocial set of outcome measures will allow us to look at the full picture of health and functioning.

**O3.2.3**
**Platelet-Rich Plasma plus Micro-Needling in Scar Management: Description of an Innovative Technique and Initial Results from 107 Patients**
**Gioia Kouthoofd** ^1^, Ester Peters ^2^, Jean Bart Jaquet ^2^
^1 ^ Surgery Department, Maasstad Hospital, Rotterdam, The Netherlands
^2 ^ Plastic, Reconstructive and Hand Surgery Department, Maasstad Hospital, Rotterdam, The Netherlands
**Aim:**

The aim of this study is to evaluate the safety, clinical effects, and patients’ experience of a combination treatment course of PRP plus micro-needling.

**Methods:**

In this retrospective cohort study, 107 patients with scars (with various etiologies and scar ages) were included. PRP was prepared from conducted venous blood, which was injected under high pressure into the dermis of the affected tissue, using the U225 meso-injector^®^ as an injection and micro-needling device. This treatment was repeated three times with a four-week interval. Patient records were collected, and clinical investigations and interviews were performed to assess the clinical effects, adverse effects, and patient’s experience on treatment days and after a four-week follow-up.

**Results:**

A total of 94 patients underwent three treatments; 21.5% of all patients reported softer and more elastic scars, 7.5% reported better mobility and functionality, 7.5% reported improvement in colour, and 5.6% reported a healthier skin appearance. The adverse effects reported were mild and temporary. Some 23 patients felt stressed before the treatment, 12 patients reported tenderness during the treatment, and 2 rated it as painful. Two patients reported transient itchiness after the first treatment. No serious adverse effects occurred.

**Conclusions:**

PRP plus micro-needling enhanced the pliability, mobility and colour of different scar types and scar ages. Overall, the treatment was well tolerated; no serious adverse effects occurred. Therefore, it can be considered a safe and effective treatment modality in scar management. Further studies should be conducted to testify our initial findings.

**O3.2.4**
**A 6-Month Follow-Up Study on the Efficacy and Tolerance of a Scar Gel Containing Aquaphilus dolomiae Extract (C+ Restore), Dimethicone, and Hyaluronate Acid with a Massage Method on Post-Burning Re-Epidermized Scars**
**Nicolas Frasson**
European Rehabilitation Center for Burns, Wounds and Scars, Lamalou, Les Bains, France
**Objectives:**

Epidermal repair is a complex process used to restore the physiological skin barrier’s function and ensure skin comfort. Hyaluronate acid (HA) is highly recommended as skin regeneration care due to its moisturizing effects. In scar care, silicones are used to improve skin softness and elasticity, especially on post-burn scars. To evaluate the efficacy and tolerance of a scar gel containing C+ restore, dimethicone, and HA (with an educational program including a massage method developed with an expert for post-burn and -surgical re-epidermized scars), we are conducting a study on adults, teenagers and infants. The objective is to improve superficial skin aspects and quality of life.

**Methods:**

This open monocentric study involves 60 subjects (2 to 65 yo) and is conducted in the spirit of good clinical practices, after the patients’ informed consent. Four visits to be conducted: baseline, 3 weeks, 3 and 6 months.

Subjects are to apply the topical gel twice daily on the studied scars (face or body; post-burn ≤ 10% of BSA for minors, and 30% for adults). Subjects are trained to performed the massage technique.

The following assessments were performed: Patient and Observer Scar Assessment Scale 2.0 (POSAS); scar aspect evolution by Investigator’s Global Assessment; soothing effect; subject and investigator’s product satisfaction using a numeric rating scale; Vitropression test; Dermatology Life Quality Index questionnaire (DLQI); Burn-Specific Health Scale-Brief questionnaire (BSHS-B); erythema by chromametry; illustrative photographs; global tolerance; and the product’s cosmetic acceptability.

**Results:**

Recruitment is ongoing. Some 33 subjects are included (16 post-burning, 17 post-surgical), as of March 2023.

**Discussion/Conclusion:**

Product quantity use and the associated economic aspects will be discussed. The results are to be communicated when available.

**O3.2.5**
**The Use of Acellular Dermal Matrices to Treat both Acute and Chronic Burns: Experience in the Burn Unit of the University and Polytechnic Hospital La Fe (Valencia)**
**Pedro Alvedro Ruiz**, Nerea Díaz Ros, Aranzazu Pérez Plaza, Ana Vidal Peraire, María Dolores Pérez del Caz
La Fe University and Polytechnic Hospital, Valencia, Spain
**Aim:**

To present our experience and the protocol of use of acellular dermal matrices for treating both acute burns and chronic sequelae in the Burn Unit of the University and Polytechnic Hospital La Fe (Valencia).

**Methods:**

We performed an observational study collecting patient data treated with Integra^®^ bilayer and Matriderm^®^ monolayer dermal matrices during the last ten years in our institution. The following demographic data were obtained from each patient: age, sex, percentage of body surface area burned, depth, mechanism and location of the area treated with dermal matrices, whether it was an acute burn or sequela, and partial or total loss of the dermal matrices.

**Results:**

We explain how the use of acellular dermal matrices has been implemented in our surgical practice, their indications for use, the surgical technique and our post-surgical multidisciplinary management with rehabilitation, physiotherapy and occupational therapy. We can use them in patients in whom we want to reduce the morbidity of the donor site. We have also seen that they reduce the appearance of pathological scars in our patients. They also constitute a very useful tool in the treatment of chronic sequelae.

**Conclusions:**

The use of dermal matrices is a common and widespread practice in surgical management of burn injuries. Protocolizing their use facilitates the complex decision tree in this group of patients. Esthetic and functional results have improved notably with a decrease in the usual complications prior to its use.

**O3.2.6**
**Possible Benefits of Oral Nutritional Supplementation or Diet in Burns and Scar Management: A Scoping Review**
**Thibau Demarbaix**Thibau Demarbaix ^1^, Ulrike Van Daele ^1,2^, Jill Meirte ^1,2^, Mieke Anthonissen ^1,2,3^, Evert De Rijcker ^1^, Koen Maertens ^1,4^, Peter Moortgat ^1^
^1 ^ Oscare NPO, Antwerp, Belgium
^2 ^ Department of Rehabilitation Sciences & Physiotherapy, Research group MOVANT, Antwerp, Belgium
^3 ^ Department of Rehabilitation Sciences, KU Leuven-University of Leuven, Leuven, Belgium
^4 ^ Vrije Universiteit Brussel, Clinical and Lifespan Psychology, Brussels, Belgium
**Aim:**

The evidence regarding a potential role of oral nutritional supplementation in burns and scar aftercare is limited. In this review, we aim to provide an overview of the possible beneficial role of supplementations in aftercare settings.

**Method:**

After formulating the research question and accompanying key words, a comprehensive search for relevant publications was performed using PubMed, Web of Science, and Google Scholar. Two authors independently identified and checked each study against the inclusion criteria.

**Results:**

After screening, 14 studies were included in the qualitative synthesis. The studies were divided in three categories based on the studied model: human, animal, and in vitro models. Six studies including human subjects showed a link between scar improvement and supplementation with vitamin D and omega-3 fatty acids, or a Solanaceae-free diet and lower omega-6 fatty acid intake.

One study on animals with atopic dermatitis showed lower pruritus levels after supplementation with konjac glucomannan.

Most of the included studies were performed on in vitro models. The results confirmed the beneficial role of vitamin D. Curcumin and quercetin supplementation were linked to decreased fibroblast proliferation. Vitamin C enhances collagen production in healthy as well as keloidal dermal fibroblasts. Chitin stimulates cell proliferation in human fibroblasts and keratinocytes.

**Conclusions:**

Additional oral supplementation might prove to be beneficial in scar management, but many studies were performed on a cellular level, and most results led to hypotheses partially derived from relationships between scars and nutritional compounds. Future in-depth research should focus on trials in human populations and the role of nutrition on specific scar characteristics.

**O3.2.7**
**Autologous Cell Spray Grafting in the Management of Partial-Thickness Burn Wounds**
**Bernd Hartmann**, Simon Kuepper, Claudia Belfekroun
ukb Berlin, Berlin, Germany
**Aim:**

Decreased healing time results in lower inflammation and reduces the risk of hypertrophic scarring. Autologous cell spraying with different preparations of skin cells has been carried out since 2005 in our burn center. We have developed various types of cell preparations and applications over time.

**Methods:**

Since 2005, more than 340 patients have been treated with autologous cells sprayed on partial-thickness burns. Different dressing modalities were tested as well. Some 275 patients were treated with a polylactide membrane.

**Results:**

In the first prospective study on 19 patients with burns to the face and neck, the TBSB was on average 15.1%. The ABSI was on average 6.7 points. The sprayed areas had a mean of 2%. Therefore, patients could be re-evaluated after a mean of 10 months with an average Vancouver Scar Scale of 2.4 ± 2.2 points.

In the second retrospective study on 103 patients, 93 were treated after spraying of skin cells prepared in our own skin bank with different dressing protocols. Debridement with a hydro surgery system or Bromelain gel did not warrant a statistically significantly longer healing time.

**Conclusions:**

Our data show that enzymatic and careful surgical debridement and consecutive application of cell suspensions using a spray technique results in excellent cosmetic outcomes. It has been established as a standard technique clinically, and successfully applied to children and adults with excellent clinical results.

**O3.2.8**
**SkinTERM: Skin Tissue Engineering and Regenerative Medicine, a Marie Skłodowska-Curie Action**
**Willeke Daamen** ^1,2^, Danique Hof ^1,2^, Bouke Boekema ^2,3,4^
^1 ^ Radboud university medical center, Department of Medical BioSciences, Nijmegen, The Netherlands
^2 ^ SkinTERM
^3 ^ Association of Dutch Burn Centers (ADBC), Preclinical Research, Beverwijk, The Netherlands
^4 ^ Amsterdam UMC, Vrije Universiteit Amsterdam, Department of Plastic, Reconstructive and Hand Surgery, Amsterdam, The Netherlands
**Aim:**

The EU-funded innovative training network SkinTERM aims to convert the normal mode of skin repair into skin regeneration by delivering excellently and multidisciplinary trained scientists.

**Methods:**

Current treatments of skin burns and large trauma have serious drawbacks, including pain, mobility-limiting contractures and disfiguring scars. We aim to induce skin organogenesis using key extracellular matrix elements taken from fetal and non-scarring spiny mouse systems, and by employing cells from relevant cellular origins. Furthermore, we investigate methodologies to regenerate skin appendages and take initial steps for clinical translation.

**Results:**

Experiences from the SkinTERM network will be shared, where a solid training program is combined with innovative skin regeneration research.

Regenerative components, including type III collagen and elastin hydrolysates, were applied to construct and characterize three-dimensional collagen biomatrices. Collaborative proteomics and transcriptomics studies of the spiny mouse and human fetal/adult/eschar fibroblasts have started. A human skin organoid model was established to study fibroblast lineages and to propagate sweat gland stem cells. Hair proto-follicle dermal cell cultures were applied in bilayered 3D spheroids. The first protocol for a melanocyte-containing skin construct was designed.

**Conclusions:**

The training and cutting-edge research output from SkinTERM will deliver supradisciplinary and intersectorially trained scientists with knowledge regarding a wide variety of aspects in wound regeneration, which is necessary to drive this research area further towards clinical translation.

This project has received funding from the European Union’s Horizon 2020 research and innovation programme under the Marie Skłodowska-Curie grant agreement No. 955722.

**Friday 8 September 8–9:50 am**
**Session: Infections**
**O3.3.1**
**Five-Year Review of Bacteriological Profile of Patients Treated in a Clinic of Plastic and Reconstructive Surgery in a Bulgarian University Hospital**
**Iren Bogeva-Tsolova** ^1^, Preslava Hristova-Trifonova ^2^

^1^  Department of Surgical diseases, Clinic of Plastic and Reconstructive surgery, Medical University Pleven, Bulgaria, Pleven, Bulgaria
^2^  Department of Microbiology and Virology, Medical University Pleven, Bulgaria, Pleven, Bulgaria

**Introduction:**

Burn unit patients are a high-risk group for wound infections caused by different microorganisms. Since its foundation in 2003, the Clinic of Plastic and Reconstructive Surgery at University Hospital “Dr. Georgi Stranski”, Pleven, Bulgaria had several transformations, and this may be the reason why a microbiological analysis has not been performed so far. The clinic works mainly with burn patients with no more than 20% total body surface area burned, and also patients with soft-tissue defects of different origins.

**Aim:**

To analyze the microbiological profile and susceptibility of the patients treated at the Clinic of Plastic and Reconstructive Surgery over a 5-year period. We avoided the COVID-19 pandemic period in order to present data that reflects the normal work dynamic.

**Methods:**

The data for this study were collected from the patients’ medical records between January, 2016 and December, 2020 in the plastic surgery ward.

**Results:**

A total of 601 patients were hospitalized during the study period. The presence of bacterial isolates was detected in 302 (50.25%) of these individuals. In the majority of the patients (194 (64.24%)), a single bacterial isolate was detected, whereas in 108 (35.76%), a combination of two or more bacterial isolates was confirmed. A total of 408 bacteria were isolated, and the most common species were S. aureus (n = 115); Enterococcus spp. (n = 68); E. coli (n = 41); Acinetobacter spp. (n = 30); K. pneumoniae (n = 28); and P. aeruginosa (n = 15).

**Conclusions:**

Increased antimicrobial resistance among all types of the isolates has emerged as an important concern during the 5-year period.

**O3.3.2**
**The Prevalence of Highly Resistant Micro-Organisms in Repatriated Burn Center Patients over a 35-Year Period**
**Niels Dijkshoorn** ^1^, Daan Yperen ^1^, Margriet Van Baar ^2,3^, Jan Dokter ^1^, Kees Van der Vlies ^1,4^
^1 ^ Burn Center, Maasstad Hospital, Rotterdam, The Netherlands
^2 ^ Association of Dutch Burn Centers, Maasstad Hospital, Rotterdam, The Netherlands
^3 ^ Erasmus MC, University Medical Center Rotterdam, Department of Public Health, Rotterdam, The Netherlands
^4 ^ Trauma Research Unit Department of Surgery, Erasmus MC, University Medical Center Rotterdam, Rotterdam, The Netherlands
**Aim:**

To study highly resistant micro-organism (HRMO) cultures in repatriated patients on and during their burn center admission over a thirty-five year period in The Netherlands.

**Method:**

All patients repatriated from a hospital abroad to the Maasstad Hospital Burn Center (Rotterdam, The Netherlands) between 1987 and 2022 were included. On and during admission, the nose, throat, perineum, and burn wounds of repatriated patients were tested for the presence of three HRMOs: methicillin-resistant Staphylococcus aureus (MRSA), Acinetobacter, and Pseudomonas. The prevalence rates of total HRMOs as well as the separate types were calculated.

**Results:**

The study included 219 repatriated patients (with a median total body surface area (%TBSA) burned of 8.0% (IQR 4.0–16.0)). A total of 41 patients (19%) had one or more HRMOs on or during their burn center admission, including MRSA (n = 22; 10%), Acinetobacter (n = 20; 9%), and Pseudomonas (n = 17; 8%). Thirty patients (14%) were colonized on admission; the other eleven colonized patients (5%) tested positive during admission with median 4.0 days (IQR 2.0–9.0) after admission. No trend was observed in the number of colonized repatriated patients in our burn center between 1987 and 2022.

**Conclusions:**

Almost one out of five of the repatriated patients had one or more HRMOs on or during their burn center admission. This far exceeds Dutch population levels (1%) and highlights the importance of testing repatriated patients for HRMOs on and during their admission to prevent the spread of HRMOs in the hospital.

**O3.3.3**
**Progression of Fungal Wound Colonization to Fungal Wound Infection after Burn Injury: A 15-Year Review**
Kaitlin Pruskowski, **Leopoldo Cancio**
United States Army Institute of Surgical Research, JBSA Fort Sam Houston, USA
**Aim:**

Fungal wound infection (FWI) but not fungal wound colonization (FWC), is an independent predictor of mortality in burn patients. In this study, we examined whether patients with FWC may progress to FWI, and whether progression is associated with increased risk of death.

**Methods:**

FWC is defined as the presence of fungal elements in non-viable tissue, and FWI as the presence of fungal elements in viable tissue or angioinvasion. Patients admitted between 2004 and 2019, initiated on a systemic antifungal out of concern for FWI but wih an initial diagnosis was FWC, were identified. These patients were followed to determine whether FWI developed. This retrospective study was approved by the Institutional Review Board.

**Results:**

Some 117 patients had FWC. Of these, 79.5% did not develop FWI (Group A), and 20.5% did (Group B). Demographic data and burn size did not differ between the groups. The average time from FWC to FWI was 13.9 ± 18.5 days.

For patients who had positive fungal wound cultures, Aspergillus was the most commonly isolated genus, followed by Candida, Fusarium, and Mucor. Group A had a longer hospital LOS than Group B (90.8 ± 73.7 days vs. 61.9 ± 44.3 days; *p* = 0.018), likely due to the higher mortality in Group B (45.2% vs. 75%; *p* = 0.009).

**Conclusions:**

Over 20% of patients who presented with FWC developed FWI, and progression was associated with a significant increase in mortality. Patients with FWC should be frequently reassessed; topical antimicrobials should be changed to include coverage for molds, and aggressive surgical intervention should be performed as indicated.

**O3.3.4**
**Acute Infective Endocarditis in Burn Patients: A Prospective Study**
**Ouissam El Ourf**, Hana Fredj, Hasan ALzain
Traumatology and Burn Center, Tunis, Tunisia
**Introduction:**

In burn patients, infective endocarditis (IE) is under-diagnosed and is often reported postmortem.

The aim of our study was to determine the incidence of IE in burn patients and to describe its clinical, ultrasonographic, bacteriological and evolutionary characteristics.

**Methods:**

This was a prospective descriptive study conducted over a period of 7 months (August 2022–February 2023). All burn patients with sepsis who had a transthoracic (TTE) and/or transesophageal ultrasound (TEE) were included. TTE and/or TEE was performed in all severely burned patients, and repeated at each septic episode. Children and non-burned patients were excluded. An IE was considered when there was a septic state with bacteremia and the appearance of vegetation on ultrasound.

**Results:**

Some 207 patients were hospitalized, 88 of whom presented a sepsis; only 56 patients were included. The diagnosis of IE was retained in four patients, representing an incidence of 2% in all admitted patients, and 4.5% in septic patients. All patients were male, their age ranged from 28 to 70 years, and the average TBSA was 28%. The mean time to IE onset was 9.7 days. Cardiac ultrasound showed vegetation on the native aortic valve in all cases. The causative organisms were Pseudomonas Aeroginosa in two patients (n = 2), Staphylococcus in one patient (n = 1) and Candida Albicans in one patient (n = 1). Only one of our patients survived.

**Conclusions:**

IE is a frequent complication in septic burn patients, with an incidence of 4.5%, and is accompanied by a high mortality rate (three in four patients died).

**O3.3.5**
**The State of Burns Care in Zambia, Southern Africa, A Scoping Review**
**Nancy Kasongo** ^1,2^, Anya Nowbuth ^2^, Kapungwe Kaunda ^3^, Samudani Dhanasekara’s ^4^, Professor Sharmila Dissanaike ^4^
^1 ^ Levy Mwanawasa University Teaching Hospital, Lusaka, Zambia
^2 ^ Pan African Organization for Health Education and Research, Saint Louis, USA
^3 ^ Kafue General Hospital, Lusaka, Zambia
^4 ^ Texas Tech University Health Sciences Center, Lubbock, USA
**Aim:**

To provide a comprehensive analysis of the state of burns care in Zambia, utilizing a multidisciplinary approach.

**Methods:**

A scoping review was undertaken using the African Journals online, Zambia Medical Journal, PubMed, the University of Zambia Thesis Repository, article reference lists, and specific author searches. Data on study characteristics, patient demographics, clinical course, burns prevention, surgery, analgesia, physiotherapy, and training were extracted.

**Results:**

Twenty papers were identified for this review, and two excluded (unavailable online). This review includes 18 papers, published from 1979–2023. This number includes six interventional studies, retrospective studies, and cross-sectional studies, respectively. Some 61% of the studies were conducted in Lusaka, at the Iniversity Teaching Hospital. The total number of burns patients identified in this study were 8710, with comprehensive data on 4368. The mean age was 7.8 years, and the male-to-female ratio was 1. The mean total burnt surface area was <10%, and the mortality rate was 24%, and 100% for TBSA > 40%. The commonest cause of burns was scalding from hot liquids (70%).

The average burn wound infection rate was 37%, and average length of hospital stay was 15.7 days. A high burden of contractures needing surgical intervention contributed nearly 80% of surgical operations on burns. Prevention studies have shown low levels of burns prevention knowledge in communities. Research was non existent on various burns care components.

**Conclusions:**

This study highlights a high disease burden of burns resulting from complications and limited research. Strides to improve care must focus on increasing research funding, mentorship, reliable record storage, health worker training, and community prevention efforts.

**O3.3.6**
**Incidence of Different Types of Infection in the Critical Burn Unit of a Tertiary Hospital**
**Elena Diaz**, Ana Vidal Peraire, Ana Belén Artero Castaño, M. Ángeles Ferre Colomer, Pilar Argente Navarro
Hospital La Fe, Valencia, España
**Aim:**

To assess the incidence and outcomes of infections in a critical burn unit of a tertiary hospital.

**Methods:**

We conducted a retrospective study on patients admitted to our critical burn unit between 2017 and 2022 with a burn percentage > 40%. We collected data during their stay in the unit: demographic, burn percentage, number and type of infections, day of first positive culture, development of septic shock and mortality. For correlation analysis, a multiple regression model using R software was used.

**Results:**

We included a total of 25 patients (20 men/5 women, and a mean age of 44 years old) in the study, with an average burn percentage of 60% and a mortality rate of 40%. During follow-up, 19 (76%) developed at least one infection, and the incidence of any infection in the 20 patients who survived more than 4 days was 95%. The most frequent infection was cutaneous (79%), followed by pneumonia (53%), bacteremia (47%), and UTI (26%). Mortality was negatively correlated with day of first infection, and it was higher in patients with septic shock (44% vs. 9%). No correlation was observed with the type of infection. Burn percentage was not significatively correlated with the presence of septic shock or mortality.

**Conclusions:**

Infections are a common complication among patients admitted to critical burn units. We found that cutaneous infections were the most frequent, followed by pneumonia. Mortality was higher in patients with septic shock, which was present in nearly half of the patients.

**O3.3.7**
**Primary Cutaneous Aspergillosis in Pediatric Burns**
**Doina Iulia Nacea** ^1,2^, Dan Mircea Enescu ^1,2^, Raluca Teodora Tatar ^1,2^, Cristina Stoica ^2^, Maria Tomita ^2^
^1 ^ “Carol Davila” University of Medicine and Pharmacy, Bucharest, Romania
^2 ^ “Grigore Alexandrescu” Clinical Emergency Hospital for Children, Bucharest, Romania
**Aim:**

The aim of the study is to synthesize and share experiences from the Burn Unit from the “Grigore Alexandrescu“ Clinical Emergency Hospital for Children, Bucharest, Romania, regarding the diagnosis and therapeutic management of primary cutaneous Aspergillosis in the burn patient, an uncommon infection associated with increased mortality, morbidity, and treatment costs.

**Methods:**

We performed a retrospective study, analyzing the files of all burn patients admitted to our department. The inclusion criteria were the presence of a burn injury concomitantly with confirmed cutaneous Aspergillosis. The investigated period ran from June 2020 (the date of first positive wound culture for Aspergillus in our department) until March 2023.

**Results:**

We identified four patients, three boys and a girl, with ages between 13 and 17 years. All of them were admitted with deep flame burns ranging from 55% to 90% TBSA. All of them required highly specialized treatment in the ICU, and benefited from extensive excision-grafting surgeries, half of them with meshed graft, and the other half using the Meek graft technique. The suspicion of infection was raised by changes in the appearance of the dressings and wounds. After diagnosis, all of the patients received systemic and topical antifungal treatment. Three of the patients had a favorable evolution, and one of them died.

**Conclusions:**

The early diagnosis of primary cutaneous Aspergillosis, via swab culture monitoring, followed by appropriate treatment, is essential in preventing the transformation of this aggressive opportunistic local fungal infection into sepsis, thereby increasing the patient’s chances of surviving.

**Friday 8 September 1:30–3 pm**
**Session: Organizational Standards and Mass Casualties**
**O3.6.1**
**The Burden of Disease of Fatal and Non-Fatal Burn Injuries for the Full Spectrum of Care in the Netherlands**
**Margriet Van Baar** ^1,2^, Inge Spronk ^1,2^, Robert A. Verheij ^3^, Martien J Panneman ^4^, Juanita Haagsma ^2^

^1^  Association of Dutch Burn Centers, Maasstad Hospital, Rotterdam, The Nederland
^2^  Department of Public Health, Erasmus MC, University Medical Center Rotterdam, Rotterdam, The Nederland
^3^  Nivel, The Netherlands Institute for Health Services Research, Utrecht, The Nederland
^4^  Consumer Safety Institute, Amsterdam, The Nederland

**Aim:**

To estimate the burden of disease of burns for the full spectrum of care in the Netherlands, and explore changes over time.

**Methods:**

General practice, emergency department, hospital, and mortality data from 2014–2018 were collected. Years lived with disability (YLD), years of life lost (YLL), and disability-adjusted life years (DALY) were estimated for each year using a tailored methodology.

**Results:**

Burns resulted in a total of 9278 DALYs (0.54/1000 inhabitants) in the Netherlands in 2018, comprising 7385 YLDs (80%) and 1892YLLs (20%). Burn patients who visited the general practice contributed most DALYs (64%), followed by deceased patients (20%), patients admitted to hospital (14%), and those treated at the emergency department (2%). The burden of disease was comparable in both sexes (4734 DALYs for females; 4544 DALYs for males), though the distribution of DALYs by level of care varied. Females contributed more DALYs at the general practice level, and males at all other levels of care. Among children, boys 0–4 years had the highest burden of disease (784 DALYs); among adults, females 18–34 years old (1319 DALYs). Between 2014 and 2018, there was a marginal increase of 0.8% in the number of DALYs.

**Conclusions:**

Burns cause a substantial burden of disease, with burns requiring care at the general practice level contributing most DALYs. Information on the burden of burns by the full level of care as well as by subgroup is important for the development of tailored burn prevention strategies, and the figures are recommended for use in priority setting and resource allocation.

**O3.6.2**
**The Gas Pipeline Explosion of Ghislenghien in 2004: A Successful Implementation of the Stay and Play Doctrine in the Most Serious Belgian Outdoor Mass Burn Casualty Disaster**
**Serge Jennes**, François Debry, Ghueder Saidane
Grand Hôpital De Charleroi, Charleroi, Belgium
**Aim:**

To highlight the role of triage by burn teams (B-Teams) in a forward medical post (FMP) and in an evacuation hospital, as well as the importance of helicopters in a mass casualty incident (MCI).

**Methods:**

We conducted a retrospective study based on the data of all the injured involved, alongside a literature review.

**Results:**

There were 171 victims, including 24 dead (16 on the spot and 8 later at the hospital) and 147 injured, including many burns and psychological shocks. Some 102 victims were treated at the FMP and the evacuation hospital. There were 69 hospitalizations of more than 72 h. A total of 65 patients were taken care of in 11 burn centers (BC) (6 Belgian and 5 French). 55 victims were burned over 15% TBSAb, including 20 over 40%. Global and hospital mortality was 14% (24/171) and 10% (7/69), respectively. This is low for an outdoor disaster. In terms of evacuation to BC, there were 41 primary evacuations, and 33 evacuations by helicopter, including 23 primary with 1 death in flight. All the victims were hospitalized in BC in the first 6 h post-burn.

**Conclusions:**

The medical management of the Ghislenghien disaster is a textbook case of the stay and play doctrine in disaster medicine. We have not been able to show any statistically significant differences in terms of morbidity/mortality between the FMP and the evacuation hospital, nor between air and ground evacuations. The B-Teams and the helicopters have shown their central role. The cross-border cooperation between France and Belgium worked well.

**O3.6.3**
**Patient or Prisoner? Acute Burn Injuries in Prisoners: The Birmingham Burns Center Experience**
**Arash Rafie** ^1^, Hadyn, K, N Kankam ^1,2^, Amritpal Sandhu ^1^, Elizabeth Chipp ^1^
^1 ^ Department of Burns and Plastic Surgery, University Hospitals Birmingham NHS Foundation Trust, Birmingham, UK, Birmingham, UK
^2 ^ Institute of Inflammation and Ageing, University of Birmingham, Birmingham, UK
**Aim:**

Increased risk of violence and self-harm means prisoners are a vulnerable population with complex health needs. They account for a small proportion of patients with burn injuries, but present a unique set of challenges. This study investigates the incidence, patterns and outcomes of burn injuries in a prison population.

**Methods:**

Prisoners referred from 2010–2021 were identified using the International Burn Injury Database (iBID). Patient demographics, burn injury characteristics, and outcomes were collected. Patients were then stratified based on the mechanism of injury, treatment modality (surgery/conservative), hospital admission (inpatient/outpatient), and compliance with outpatient follow-up, for subgroup analyses.

**Results:**

Sixty-eight prisoners sustained burns during the study period, with a median age of 28.5 years and TBSA of 3%. The majority were male (98.5%) and required hospital admission (75%). Scalds were the most common injury type (77.9%) and assault the most frequent cause of burns (63.2%). Eighteen patients (26.5%) underwent a surgical procedure, and there were two mortalities. Of patients for whom follow-up was planned, 22% attended no appointments, with a further 49% of prisoners missing at least one appointment. Relative to patients managed non-operatively, prisoners undergoing surgery had a longer stay, and all attended outpatient follow-up appointments.

**Conclusions:**

Prisoners represent a unique population with exceptional challenges. Attention should be given to protecting vulnerable patients at risk of assault, education should be given prison staff around burn prevention and first aid, and we should ensure that prisoners are able to access burns follow-up to minimise long term sequelae. Opportunities exist to aid this, such as the adoption of telemedicine.

**O3.6.4**
**The Development and Implementation of an Interactive Patient-Reported Outcome Dashboard in Dutch Burn Care**
**Marjolein Van Der Vlegel** ^1^, L van Dammen ^1^, M van den Berg-van Leeuwen ^3^, M.E van Baar ^3,4^, the National Burn Care, Education & Research group the Netherlands ^2,5,6^
^1 ^ Dutch Burns Foundation, Beverwijk, The Netherlands
^2 ^ Burn Center, Maasstad Hospital, Rotterdam, The Netherlands
^3 ^ Association of Dutch Burn Centers, Maasstad hospital, Rotterdam, The Netherlands
^4 ^ Erasmus MC, University Medical Center Rotterdam, Department of Public Health, Rotterdam, The Netherlands
^5 ^ Burn Center, Red Cross Hospital, The Netherlands
^6 ^ Burn Center, Martini Hospital, Groningen, The Netherlands
**Aim:**

A value-based healthcare (VBHC) framework will be developed and implemented in the Netherlands to improve person-centered burn care. A key component in this is the development of a dashboard for health care professionals (HCPs) and patients to provide insights into outcomes, expected recovery patterns, and to support shared decision making (SDM). Our aim is to develop this person-centered dashboard, to test its usability, and to implement the use of the dashboard in clinical practice in Dutch burn care.

**Methods:**

The three Dutch burn centers have a joint patient-reported outcomes registry (Burn center Outcomes Registry the Netherlands (BORN)). An interactive dashboard was developed, visualizing patient-reported outcomes (PROs). HCP and patient focus groups were conducted to identify needs and inform the dashboard’s design. Usability was tested in think-aloud interviews with HCPs. After a period of use, HCPs feedback was collected for optimal implementation of the dashboard in daily practice.

**Results:**

The developed dashboard includes patient information (age, sex, time since burns), general open questions on the patients’ recovery and PROs on general health, mental health and social outcomes, and scar quality. The development was based on the Dutch Santeon hospitals’ guidelines, and included feedback and suggestions of HCPs and patients.

**Conclusions:**

Visualizing PROs in a dashboard is experienced by HCP as a valuable way of reporting outcome information. A person-centered dashboard is a tool that may stimulate patient activation and support SDM. The next step is to make the dashboard accessible for patients at home.

**O3.6.5**
**The Urgent Need to Achieve an Optimal Strategic Stock of Human Allogeneic Skin Graft Materials in Case of a Mass Disaster in Poland**
**Wojciech Łabuś** ^1^, Przemysław Strzelec ^1^, Karolina Ziółkowska ^1^, Agnieszka Klama-Baryła ^1^, Artur Kamiński ^2^
^1 ^ Stanisław Sakiel Center for Burn Treatment in Siemianowice Śląskie, Siemianowice Śląskie, Poland
^2 ^ National Center for Tissue and Cell Banking, Warsaw, Poland
**Aim:**

A burn is a sudden injury whose immediate or long-term consequences may be life-threatening for the patient. A mass disaster event may involve large numbers of severely burned patients. Non-viable allogeneic human skin grafts may be considered the most suitable skin substitutes in the treatment of such patients. At present, Poland does not have a sufficient supply of human allogeneic skin graft materials to meet the needs arising from a sudden and unforeseen mass disaster.

**Methods:**

This study involved an analysis of selected mass disasters. From this, an estimate was made from a verified casualty profile of the necessary minimum stock of human allogeneic skin graft materials. An insufficient amount of skin results from an inadequate number of skin donors, which in turn results from the current tissue donation system. Therefore, a proposal has been made for the organizational, legal and systemic changes required to improve the situation in Polish transplantology, with a particular emphasis on skin donation.

**Results:**

A tissue-collecting transplantation team should be organized. The rights and obligations of the non-physician transplant team member should be extended. Awareness campaigns and educational schemes should be prepared.

**Conclusions:**

The required essential stock of human allogeneic skin in the event of a mass disaster has been estimated at 600,000 cm^2^.

**O3.6.6**
**Enzymatic Debridement vs. Surgical Debridement for Burn Patients: A Comparison of Real Cost per Patient at a Tertiary Center**
**Patricia Martin-Playa** ^1^, Unay Yilmaz-Bescos ^1^, Iker Ustarroz-Aguirre ^2^, Borja Garcia-Lorenzo ^3^, Jaime Carames-Estefania ^1^
^1 ^ Cruces University Hospital, Bilbao, Spain
^2 ^ Cruces University Hospital, Bilbao, Spain
^3 ^ Kronikgune, Institute for Health Service Research, Barakaldo, Spain
**Aim:**

The use of enzymatic debridement (ED) has proved its clinical effectiveness, and has advantages compared to surgical debridement (SD) on second- and third-degree burns. However, there is scarce literature regarding its real costs. We aim to show the individual cost per patient and of each of the services received during hospital stay.

**Methods:**

A non-randomised, retrospective, observational study was conducted with 80 patients admitted to our Great Burn Unit and divided into two groups (ED and SD). We used a cost-per-patient information system so that the unit cost of healthcare services and the individualised cost per patient until hospital discharge could be calculated. The system includes information related to diagnosis, procedures, and sociodemographic information, and it is divided into more than 30 categories of resource consumption.

**Results:**

Both groups were statistically comparable. The mean cost per patient treated with SD was EUR 44,841 compared to EUR 36,190 with ED. The ED group showed shorter length of stay (EUR 26,101 vs. EUR 33,919), a decreased need for surgical procedures (0.45 vs. 1.28), and a shorter use of the operating theatre (EUR 202 vs. EUR 3000). The mean cost of Nexobrid^®^ per patient was EUR 1636. No other significant differences regarding resource consumption were found.

**Conclusions:**

The cost per major burn patient under ED is lower than under SD, mainly due to shorter hospital stays and decreased operating theatre time. The main predictors of the cost per patient are the type of aetiology, length of stay, and total body surface burned. Further analysis on the cost-effectiveness and social cost of burn patients should be carried out.

**O3.6.7**
**Triage in Burn Mass Casualty Incidents: Evaluation of the Performance of European Burn Assessment Teams in Simulation**
**Amy Hughes** ^1^, Stian Kreken Almeland ^2^, Nicolas Donat ^3^, Thomas Leclerc ^3^, Danae Corblet ^3^
^1 ^ Interburns, Cambridge, UK
^2 ^ Haukeland University Hospital, Bergen, Norway
^3 ^ HIA Percy/Percy Military Teaching Hospital, Clamart, France
**Aim:**

The European response plan to burn mass casualty incidents includes the in-hospital deployment of Burn Assessment Teams to assist in orienting victims. This retrospective study assessed the reliability and reproducibility of their recommendations.

**Methods:**

During a pilot training in January 2020 in the UK, five European teams triaged, following the European Burns Association’s recommendations for burn disasters, 40 simulated patients, including 16 manikins to be examined clinically under realistic conditions and 24 to be triaged by chart. Evacuation priorities (not needed, 3, 2, 1, futile) were compared between teams and with the reference. The contribution of each triage step to the identified sources of variability was analysed.

**Results:**

The recommended evacuation priority was highly reproducible between the five teams (Gwet’s AC2 agreement coefficient 0.95; 95% CI: 0.92–0.97; *p* < 1 × 10^−5^). Similarly, their compliance with the reference was high (AC2 = 0.93; 95% CI: 0.92–0.95). Besides the negligible contribution of the main two triage steps, namely categorisation by outcome/resource ratio and determination of evacuation priority, the main source of residual variability was the imperfect accuracy of the burned surface area assessed on the manikins.

**Conclusions:**

This study is the first to validate a burn disaster triage strategy using simulation. In the event of a disaster requiring the activation of the European burn mass casualty response plan, in-hospital triage by specialised Burn Assessment Teams following European recommendations would provide reproducible and reliable assistance in the orientation of victims and the organisation of medical evacuations.

**O3.6.8**
**Preparing for the Care of Burned Casualties following Nuclear Detonation**
**Leopoldo Cancio**, William Hickerson, James Jeng
US Army Institute of Surgical Research, Fort Sam Houston, TX, USA
**Aim:**

Since World War II, we have recognized that a nuclear detonation (NucDet) could generate thousands of burns. How best to care for the survivors remains challenging. In response to a request from the US Agency for International Development (USAID), the authors convened a Burn Working Group (BWG) from February to March 2023 to develop a concept of operations and a burn supply list (BSL) for a possible tactical NucDet during the current war in Ukraine.

**Methods:**

The BWG consisted of three burn surgeons with battlefield/mass-casualty experience. We met weekly for 6 weeks in consultation with USAID colleagues. We developed a BSL to provide initial care to 5000 burned survivors, intended for NATO Role I use (self-aid, buddy aid, prehospital medic).

**Results:**

We identified the following priorities: assess for other injuries and acute radiation syndrome (ARS); carry out triage; manage pain; expose the patient; cleanse wounds and apply antimicrobial dressings; provide oral resuscitation; keep the patient warm and provide education, emotional, and spiritual support; provide follow-up instructions; and discharge to ambulatory status if possible. The BSL includes oral analgesics, antiseptic solution (chlorhexidine gluconate), silver nylon dressings, gauze dressings, bacitracin (for the face and eyes), World Health Organization Oral Rehydration Salts, and reflective aluminum casualty blankets. We developed laminated instruction cards for use by minimally trained personnel.

**Conclusions:**

We proposed an approach to a NucDet, assuming that minimally trained personnel could perform basic tasks in early burn care. Further work is needed to validate this assumption, and to better understand how best to manage combined injury (burns plus ARS) in an austere environment.

**Friday 8 September 1:30–3 pm**
**Session: Wounds**
**O3.7.1**
**Suprathel after Enzymatic Debridement with Bromelain: Our Experience**
**Nerea Díaz Ros**, Pedro Alvedro, Ana Vidal, Alesssandro Thione, María Dolores Pérez del Caz
Hospital Universitari I Politècnic La Fe, Valencia, Spain
**Introduction:**

suprathel is an alloplastic skin substitute which consists of a synthetic microporous absorbable membrane made up of polylactic copolymer, trimethylene carbonate, and ecapolacto. It is widely used in split-thickness skin graft (STSG) donor sites, superficial and partial-thickness burns, and abrasions for skin re-epithelialization. A particular use of suprathel is covering the bed of deep burns left after an enzymatic debridement with bromelain.

**Aim:**

To review the use of suprathel in deep burns previously debrided with bromelain in our burn unit.

**Methods:**

We reviewed the re-epithelialization rate, the time to complete re-epithelization, the need for surgical intervention after using suprathel, and the functional outcome in patients treated with suprathel over the bed of deep burns previously debrided with bromelain in the last five years in our burn unit.

**Results:**

The use of suprathel in our burn unit has reached high rates of spontaneous re-epithelization of the bed of deep burns that have been enzymatically debrided with a low local infection rate. It has also reduced the need for surgical intervention after enzymatic debridement, and therefore decreased the length of hospital stay and further complications, leading to optimal functional and aesthetic short-term and long-term results for these patients.

**Conclusions:**

Suprathel is a suitable skin substitute for deep burn beds after enzymatic debridement with bromelain, achieving optimal functional and aesthetic results.

**O3.7.2**
**Use of Human Amniotic Membrane as a Temporary Biological Dressing in Toxic Epidermal Necrolysis: Literature Review and Case Report of Our Center**
**Pedro Alvedro Ruiz**, Nerea Díaz Ros, Aranzazu Pérez Plaza, Pilar Corella Estévez, María Dolores Pérez del Caz
La Fe University and Polytechnic Hospital, Valencia, Spain
**Aim:**

To determine whether amniotic membrane application is a suitable option for treating severe skin lesions in patients with TEN.

**Method:**

A review of the literature was carried out using the PubMed and ScienceDirect databases, combining the terms “amniotic membrane”, Lyell’s Syndrome” and “Toxic Epidermal Necrolysis”. After eliminating duplicates, a total of 22 articles were obtained, of which 8 were selected for this study. In addition, a clinical case report of our institution is included.

**Results:**

Human amniotic epithelial stem-cells may promote skin wound healing by accelerating keratinocyte proliferation and migration via the ERK, JNK and AKT signaling pathways. In accordance with these experimental results, we observed an excellent evolution of the wounds of a patient in which amniotic membrane was applied, reaching complete re-epithelialization after 3 weeks of hospitalization in the Burn Unit of our center.

**Conclusions:**

The use of amniotic membrane as a temporary biological dressing may promote re-epithelialization of skin lesions in patients with TEN or burns due to its proliferation and angiogenic factors. In addition, it may reduce wound exudate and pruritus. Nevertheless, more experience and scientific evidence is needed.

**O3.7.3**
**Cold Burns in the United Kingdom: A Cohort Study of Patients Presenting to a Regional Burn Unit**
**Alexander Baldwin**, Deepika Bhojwani, Alexandra Murray
Buckinghamshire Healthcare NHS Trust, Aylesbury, UK
**Aims:**

To assess the aetiology, management and outcomes of cold burns presenting to a regional burn unit.

**Methods:**

This was a retrospective cohort study of consecutive patients over a two-year period (2021–2022). Details regarding injury, management, and outcome were extracted. Statistical analysis was performed using a Kruskal–Wallis test and a Dwass–Steel–Critchlow–Fligner pairwise comparison for non-parametric continuous variables, and a chi-squared or Fisher’s exact test for categorical variables, with Bonferroni correction for pair-wise comparison.

**Results:**

Thirty-five patients (M:F 20:15; median age 23 [IQR 16]) were identified. The most common aetiologies were aerosol (n = 15, 42.9%), recreational nitrous oxide use (n = 6, 17.1%), and environmental (n = 5, 14.3%). Most cases were accidental (n = 21, 60%). Four (11.4%) were sustained during a social media ‘challenge’. Ten (28.6%) were caused by self-harm, of which three (33.3%) were psychiatric ward inpatients. All deliberate injuries were caused by aerosol. Deliberate ‘challenge’ injury patients were younger than those with self-harm (*p* = 0.012) and accidental injuries (*p* = 0.005). A greater proportion of self-harm injuries were in female patients compared to accidental injuries (*p* = 0.004). The median TBSA was 0.4% (IQR 0.3). There were 8 (22.9%) full-thickness, 12 (34.3%) deep-dermal, and 15 (42.9%) superficial partial-thickness injuries. The limbs were most frequently affected (n = 31, 88.6%). Aetiology and whether accidental or deliberate did not affect the TBSA (*p* = 0.62, *p* = 0.94), healing time (*p* = 0.46, *p* = 0.67), depth (*p* = 0.75, *p* = 0.58), or location of burn (*p* = 0.12, *p* = 0.41). Four (11.4%) patients required grafting. The median time to healing was 21 days (IQR 22.75).

**Conclusions:**

A disproportionate number of cold burns are deliberately self-inflicted, either as self-harm or due to a concerning recent trend of social media ‘challenges’ using aerosols.

**O3.7.4**
**The Safety, Efficacy and Clinical Outcomes of Cadaveric Cryopreserved Allograft Skin**
**Andrea Dunkelman MD FACS**
Orange County Burn Center, 1001 North Tustin Ave, USA
**Aim:**

To discuss the use, safety and advantages of cryopreserved cadaveric skin with viable cells.

**Methods:**

Cadaveric allografts are the gold standard for the closure of large burns, offering many advantages over other available biologic dressings. The optimal cadaveric allograft is fresh cadaveric skin, but it is limited by availability and shelf life. Cell viability and structural integrity are essential components for wound bed optimization. Current methods of allograft preservation include cryopreservation and glycerol-preservation. Cryopreservation utilizes 11% glycerol but maintains cell viability, while glycerol-preserved skin utilizes 85% glycerol and results in nonviable cells. There is controversy regarding cell viability and its performance as a biologic dressing. In this presentation, we will review the safety and patient outcomes of the use of cryopreserved skin. We will review the recovery and processing of cryopreserved skin and the present literature and patient case studies using cadaveric viable cell allografts on full-thickness and partial-thickness burns.

**Results:**

Allograft with live cells promotes angiogenesis and dermal regeneration. These viable allografts may decrease mortality risk as well as length of hospital stay. Cryopreserved skin is a safe option, effective in both partial and full-thickness burns, resulting in improved clinical outcomes.

**Conclusions:**

Cryopreserved cadaveric allografts with viable cells are a safe available option for the treatment of large burns, which may result in better clinical outcomes.

**O3.7.5**
**Enzymatic Debridement with Bromelain for Facial Burns: Our Experience**
**Nerea Díaz Ros**, Pedro Alvedro, Ana Vidal, Eva López, María Dolores Pérez del Caz
Hospital Universitari i Politècnic La Fe, Valencia, Spain
**Introduction:**

Facial burns account for up to half of the burns seen in a burn unit. Although most are superficial, the deepest ones have a significant impact on the quality of life of these patients, because the face is involved not only in self-perception and communication, but also in sensitive functions such as hearing, tasting and breathing, all of which can be affected by a facial burn. Therefore, optimal management of facial burns is vital to achieving the best aesthetic and functional results for these patients.

**Aim:**

We reviewed the results of enzymatic debridement with bromelain in patients with facial burns admitted to our unit.

**Methods:**

We conducted a descriptive and retrospective study reviewing all the patients with facial burns admitted to our burn unit within the last six years. We described the type of burn and the total burnt surface, the need for subsequent surgical intervention, and the aesthetic results.

**Results:**

Out of the 319 patients with facial burns admitted in our unit from 2017 to 2023, 82.4% presented with second- and/or third-degree facial burns. The deepest were enzymatically debrided with bromelain (n = 16), and less than half of those eventually needed surgical intervention. In order to evaluate the aesthetic results, we conducted a satisfaction survey and carried out a photographic assessment.

**Conclusions:**

Implementing the use of bromelain results in an early and selective debridement, an actual rate of spontaneous reepithelization, and a reduction in the need for surgical intervention, which leads to optimal functional and aesthetic results for patients with facial burns.

**O3.7.6**
**Biological Selection and Qualification Strategy of an Allogeneic Bank of Human Dermal Fibroblasts Used for the Preparation of Epidermal Substitutes**
**Celine Auxenfans** ^1^, Sebastien Banzet ^2^, Marie Rose Rovere ^1^, Marina Trouillas ^2^
^1 ^ Hospices Civils De Lyon, Lyon, France
^2 ^ IRBA, Clamart, France

Irradiated human allogeneic dermal fibroblasts are used to promote the culture of keratinocytes and the preparation of epidermal substitutes for the treatment of severe burns. We present the biological selection and qualification strategy of this allogeneic cell bank used for hospital exemption products.

Fibroblasts are collected from the foreskin of a healthy, selected child under 10 years old. They are isolated and cultured in a good-manufacturing-practice (GMP) production area and managed in a cell bank system. Fibroblasts extracted from the starting material are cultured until passage 6, and frozen in a master cell bank (MCB). A working cell bank is prepared in passage 9, and irradiated cells are used in passage 11.

The biological selection and qualification were carried out according to European Pharmacopoeia guidelines and a risk-based approach. First, irradiated dermal fibroblasts from three donors were compared on their functional capacity to support keratinocytes’ proliferation, clonogenicity, and the ability to form an epidermal sheet. Fibroblasts from one donor gave results comparable to the feeder layer currently used. Then, we confirmed the fibroblast phenotype as of human origin. Thirdly, to ensure safety, the absence of transmissible infectious agents with contaminations that can come from the donor, from products used during the culture, or from the production environment was verified. Finally, we ensured that the cells possess a normal karyotype.

This qualified allogeneic bank will be used for the production of a cultured autologous or allogenic epidermis in Lyon (authorized) and of human plasma-based epidermal substitute in Percy (authorization request in preparation).

**O3.7.7**
**The Effectiveness of Tranexamic Acid in Burn Patients Undergoing Surgery: A Systematic Review.**
**Joeri Slob** ^1^, Rolf Gigengack ^2^, Margriet van Baar ^1,3^, Seppe Koopman ^4^, Cees van der Vlies ^1,5^
^1 ^ Burn Center Rotterdam, Rotterdam, The Netherlands
^2 ^ Department of Anesthesioloy, Amsterdam UMC, VU Medical Center, Amsterdam, The Netherlands
^3 ^ Association of Dutch Burn Centers, Rotterdam, The Netherlands
^4 ^ Department of Anesthesiology, Maasstad Hospital, Rotterdam, The Netherlands
^5 ^ Department of Surgery, Maasstad Hospital, Rotterdam, The Netherlands
**Aim:**

The aim of this systematic review is to investigate the effectiveness of tranexamic acid to reduce perioperative blood loss compared to standard-of-care in burn patients undergoing burn excisional surgery.

**Methods:**

A systematic review of the literature will be conducted according to the preferred items for systematic reviews and meta-analysis guidelines. The study was registered in PROSPERO database (CRD42023396183), before the search was conducted. The Cochrane Risk of Bias version 2 tool was used for the randomized controlled trials included. The ROBINS-I risk of bias tool was used to assess non-randomized studies of interventions for the cohort studies included. The Joanne Briggs Institute Critical Appraisal Checklist was used for the case reports included.

**Results:**

Seven articles were included in this systematic review: two randomized controlled trials, one prospective cohort study, two retrospective cohort studies, and two case reports. Data extraction on 305 patients in seven studies has been conducted by two independent reviewers. Three predefined outcomes were extracted from the original articles: blood loss, transfused packed red blood cells, and hemoglobin levels postoperatively. Data on the risk of bias and the quality of studies included will be presented at the conference.

**Conclusions:**

Articles investigating the effectiveness of tranexamic acid in reducing perioperative blood loss in burn patients are available. However, a systematic review of literature is absent. This study presents an overview of the evidence and provides directions for future research.

**O3.7.8**
**Attempted Suicide by Self-Immolation in Tunisia: 11 Years after the Revolution**
**Amel Mokline** ^1^, Hana Fraj ^1^, Imene Rjeibi ^2^, Bahija Gasri ^1^, Amen Allah Messadi ^1^
^1 ^ Trauma and Burn Center, Tunis, Tunisia
^2 ^ National Institute of Nutrition, Tunis, Tunisia
**Aim:**

To evaluate the epidemiological, clinical and evolutionary characteristics of burns by immolation in Tunisia.

**Methods:**

A retrospective study was conducted in an intensive burn care department in Tunis, over a period of 11 years after the revolution.

**Results:**

During the study period, 755 patients were included. The mean age was 33.38 years, with a sex ratio of 4.5 (618 M/137 F). Half of the patients were single, 2/3 (74.3%) had an unfavorable or medium socioeconomic level, and 35.8% were unemployed. The educational level was secondary in 46% of cases, and primary in 33.9%. Secondary transfer was noted in 53.6% of cases. Patients came from all regions of Tunisia, with a predominance of those from the Tunis area (37.8%). One third of our patients had a psychiatric history, with the notion of a previous suicide attempt in 5.1% of cases. Alcoholism and/or drug addiction were reported in 17.7% of cases. The act of self-immolation was performed in a public place in 59.2% of cases. TBSA was 41.48%, and TBSA was 7.35. Burns were deep in 66.2% of cases. Facial involvement was noted in 90% of patients. The average length of stay was 17.64 days. Two thirds of patients (72.1%) required intubation and mechanical ventilation. The mortality rate was 57.2%.

**Conclusions:**

In Tunisia, attempted suicide by immolation after the revolution represents a serious public health problem, with an average of 75 cases per year. Specific and urgent preventive measures targeting the main risk factors, in particular precariousness, unemployment, adjustment disorders, and mental illnesses, should be implemented.

**Friday 8 September 1:30–3 pm**
**Session: Pediatrics**
**O3.8.1**
**The Specificities of Burns in Children According to Age Group: Our National Center Experience**
**Amina Karray**, Hana Fredj, Bahija Gasri, Amel Mokline, Amen Allah Messadi
Intensive care burn department, Traumatology and burn center, Tunisia, Ariana, Tunisia
**Introduction:**

There are four phases of childhood (newborn, early childhood, older children, and adolescent). Burn injuries may touch these different age groups.

The aim of the study was to determine the epidemiological and clinical characteristics of different age groups admitted to our burn department.

**Methods:**

This was a retrospective study including burned patients aged less than 18 years old admitted between January 2018 and June 2022. Four groups were defined according to age in years: G1: newborn group (0–2); G2: early childhood group (2–5); G3: older childhood group (5–12); and G4: adolescent group (12–18).

**Results:**

A total of 300 child patients were admitted for burns. The majority of cases were in the older childhood group (33%). The number of burns in newborn group were the lowest (8%). Male gender predominance (61%) was more present among the older childhood and adolescent groups. Domestic accidents were the unique circumstance in the newborn group. The frequency of domestic accidents decreases gradually with age (47% in adolescent group), as opposed to leisure accidents, which increase with age (G1: 0, G2: 2.4%, G3: 11%, G4: 14%). Work accidents and suicide attempts were present only in the adolescent group (7.6% and 23%, respectively). The total burn surface area was, on average, 14%, 24%, 31% and 32%, respectively in G1, G2, G3 and G4.

**Conclusions:**

Education, which is still the main way to prevent burns, must take into account the particularities of each phase of childhood.

**O3.8.2**
**Telemedicine Used to Advance Burn Care in Ukraine: A Case Series Using Dermal Equivalents in One Institution**
**Artem Posunko** ^1^, Professor Valeriy Dihtyar ^1^, Jonathan Friedstat ^2^, Professor Alexei Vlasov ^1^, Professor Gennadiy Fuzaylov ^2^
^1 ^ Regional Medical Center of Family Health, Dnipro, Ukraine
^2 ^ Shriners Children’s Boston, Boston, USA
**Introduction:**

Telemedicine and collaboration with US doctors have the potential to link experts in specialized fields in order to produce better results and advance burn care in Ukraine, especially during the war.

**Methods:**

There were seven children who had surgeries for burns in the Regional Children’s Hospital, Dnipro, Ukraine; their ages ranged from 3 months to 16 years. Five patients presented with post-burn contractures; one child had a giant naevus, and another one had aplasia cutis of the scalp. In the burn scar cases, the scar tissue causing the contracture was dissected, and the limb repositioned in the correct physiological alignment prior to graft. The giant naevus was excised initially, providing an optimal histologically clean margin. After these initial preparations, a dermal equivalent was implanted on the wound surface and sutured in place. In the case with aplasia cutis, a dermal equivalent was directly implanted on the temporal fascia, and fixed with sutures. As the dermal equivalent to be engrafted, a split graft (0.2–0.3 mm thick) was transplanted.

**Results:**

In all cases, engraftment of the dermal equivalent was successful. There was one case with local inflammation, which was successfully resolved by prescribing antibiotics and draining the focus.

**Conclusions:**

These results allow us to conclude that the use of dermal equivalents and telemedicine with US institutions allows us to avoid complex and resource-intensive operations in many cases. However, the method used for application and the criteria for selecting patients require improvement.

**O3.8.3**
**Protocolized Strategy for the Management of Burn Wounds in the Pediatric Patient: Experience in a Spanish Tertiary Hospital**
**Unay Yilmaz**, Elvira Morteruel Arizcuren, Paula Rodriguez Ruiz, Naroa Cabrera Escondrillas, Patricia Martin Playa
Cruces University Hospital, Bilbao, Spain
**Aim:**

Burn injuries in the pediatric patient are a major source of morbidity and mortality, with substantial physiological and psychological impact that requires specialized treatment. This review aims to identify key points in the management of the pediatric burn patients, and to describe our protocol.

**Methods:**

The protocol established in our hospital is described, including initial and subsequent injury assessments, the type of hospital admission, adequate pain control and sedation, indication for enzymatic debridement, and type and frequency of dressing changes. A retrospective review was conducted including pediatric burn patients admitted to Cruces University Hospital (2021–2023).

**Results:**

Approximately 90 patients are evaluated per year, 8–10 of which require admission. The protocol we present involves clinical care from admission to the Emergency Department until hospital discharge. The management requires a multidisciplinary approach (plastic surgery, pediatrics/pediatric ICU, nursery team) in order to guarantee patient comfort, highlighting the role of appropriate sedoanalgesia during both evaluation and treatment, as well as patients’ and parents’ comfort. During admission, the topical agents and dressings for local burn wound care are tailored according to whether or not enzymatic debridement (NexoBrid^®^) or surgical treatment is required.

**Conclusions:**

The management of burns in the pediatric patient is similar to that in adults, but has certain peculiarities that must be considered. Appropriate sedoanalgesia and atraumatic local burn care play an essential role in hindering the physical and psychological stress derived from burn injury. Therefore, it is crucial to establish a multidisciplinary protocol to approach burn care in these patients collaboratively.

**O3.8.4**
**Improving the Transition Pathway from Paediatric to Adult Burns Care at Chelsea and Westminster Hospital (CWH)**
**Miss Georgia Curry** ^1^, Aditi Pandey ^2^, Paul Caine ^2^, Isabel Jones ^2^
^1 ^ Imperial College London, London, UK
^2 ^ Chelsea and Westminster Hospital NHS Foundation Trust, London, UK
**Aim:**

We aimed to assess and improve the current transition pathway from paediatric to adult burns care at CWH.

**Methods:**

Using Cerner, we identified paediatric burns outpatients aged 12–16 years from 01.12.21–28.02.23 who were likely to transition to adult services. We interviewed key stakeholders including burn therapies, psychology, and paediatrics, burns charities and the carers of the identified patients. We researched national transition guidelines and took guidance from successful transition programmes in other trusts.

**Results:**

A total of 121 paediatric outpatients were identified, with 6 likely to transition to adult care. Interviews with key stakeholders identified concerns around when to broach the topic of transition, the extent to which parents should be involved, and the patient’s experience on the adult ward. Four interviews with carers were conducted. Their concerns included medical team consistency and a lack of knowledge about the impending transition. All four agreed a formal transition meeting, online resources, and an introductory departmental tour would ease concerns. We designed intranet guidelines for paediatric staff at CWH and an information leaflet for children undergoing transition and their carers, ensuring equality, diversity, and inclusivity. Advice from national transition guidelines were included in these documents. As a result, 100% (6/6) of staff surveyed reported that our guidelines improved their knowledge of the transition process.

**Conclusions:**

Transitioning patients should have a formal transition meeting and departmental tour. Online resources such as guidelines and information leaflets can help build confidence in staff and transitioning patients. When designing these documents it is important to gather information from key stakeholders.

**O3.8.5**
**A Synthetic Epidermal Substitute in the Treatment of Partial-Thickness Burns in Paediatric Patients: A 3-Year Experience of a Tertiary Center**
**Sara Fernandes**, Leonor Carmo, Inês Teixeira, Miguel Campos, Maria Garcia
Department of Pediatric Surgery, Centro Hospitalar Universitário De São João, Porto, Portugal
**Aim:**

Suprathel^®^ [polylactide membrane (PLM)] is a biosynthetic dressing that mimics the properties of the human epithelium. Reports on its use in paediatric patients show promising results. Herein, we describe the experience of our center with the use of PLM in paediatric patients.

**Methods:**

All paediatric burn patients admitted to the Paediatric Surgery Department between November 2019 and November 2022 and submitted to PLM application were selected. A historical cohort of paediatric burn patients was used for result comparison. Clinical and demographic data were collected retrospectively.

**Results:**

One hundred twenty-two patients with a median age of 1.8 years were included. The median total body surface area was 6% (1–25%),, and burns were mainly mixed-partial thickness. PLM was applied at a median of 4 days post-burn (IQR 2–6), usually under sedation (70/122). After PLM application, the median healing time (HT) was 10 days (IQR 8–14). No correlation was found between HT and the timing of PLM application. Six cases of infection were seen, and two cases showed a failure of integration needing a skin graft. By comparison with the historical cohort with similar demographic and clinical characteristics, after selecting partial-thickness burns (n = 75 historical cohort vs. n = 122 PLM-treated), the grafting rate was significantly lower in PLM-treated patients (1.6% vs. 17.3%; *p* < 0.001).

**Conclusions:**

PLM is a promising treatment for partial-thickness burns, even when applied later during treatment. Its short HT and the lower rate of autologous skin grafts, potentially sparing donor sites, are its main advantages.

**O3.8.6**
**The Efficacy of Therapeutic Interventions on Pediatric Burn Patients’ Height, Weight, Body Composition, and Muscle Strength**
**Maxime Cuijpers** ^1,2,3^, Martin Baartmans ^4^, Koen Joosten ^5^, Paul van Zuijlen ^1,2^, Anouk Pijpe ^1,2,3^
^1 ^ Burn center, Red Cross Hospital, Beverwijk, The Netherlands
^2 ^ Association of Dutch Burn Centers (ADBC), Beverwijk, The Netherlands
^3 ^ Department of plastic, reconstructive and hand surgery, Amsterdam UMC, Amsterdam, The Netherlands
^4 ^ Department of pediatrics, Maasstad Hospital, Rotterdam, The Netherlands
^5 ^ Department of pediatric and neonatal intensive care, Erasmus MC-Sophia Children’s Hospital, Rotterdam, The Netherlands
**Aim:**

To evaluate the efficacy of therapeutic interventions on pediatric burn patients’ height, weight, body composition, and muscle strength.

**Methods:**

A systematic literature search was conducted using PubMed, EMBASE, and Web of Science up to March 2021. We considered interventional studies that reported metrics on the height, weight, body composition, or muscle strength of pediatric burn patients in a peer-reviewed journal. A meta-analysis was performed if two or more trials of clinical homogeneity reported on an outcome measure at the same time point post-burn.

**Results:**

Twenty-four interventional studies were identified, including twenty randomized controlled trials and four non-randomized trials. Most studies were conducted by a single institution. The average burn size was 44.3% (±9.5) of the total body surface area. Three categories of therapeutic interventions could be distinguished: rehabilitative exercise programs, pharmacologic agents, and nutrition support.

**Conclusions:**

Although very diverse in their mechanism of action and results, each of the interventions seemed to have a positive effect on pediatric burn patients’ height, weight, body composition, or muscle strength. In future research, it is important to evaluate the heterogeneity of treatment effects and whether participation in a therapeutic intervention allowed pediatric burn patients to reach the physical and functional status of healthy control subjects.

**O3.8.7**
**A Case Series of Pediatric Burns in a National Referral Center: About 300 Cases**
**Amina Karray**, Hana Fredj, Bahija Gasri, Amel Mokline, Amen Messadi
Intensive care burn department, Traumatology and burn center, Tunisia, Ariana, Tunisia
**Introduction:**

Burn injuries are common emergencies in the pediatric population. Recognition of the circumstances contributing to the occurrence of burn accidents is needed to prevent them.

Our objective was to study the epidemiological and clinical characteristics of children admitted to our burns department.

**Methods:**

This wasa retrospective observational study, including patients aged less than 18 years old admitted for burns between January 2018 and June 2022 to the Burn Care Department of the Burn and Trauma Center in Tunis.

**Results:**

Of a total of 2124 patients admitted for burns, 300 were children (14%). The mean age was 8 years old (ranging from 6 months to 17 years old). There was a male gender predominance (61%). The majority of our patients were victims of thermal burns (90%), mostly caused by flame (44%). A low socioeconomic level was identified in 29% of cases. Domestic accidents were the most frequent circumstance of burns (78%), occurring mostly in the kitchen (37%). The total burn surface area was, on average, 21%. The most affected areas were the trunk and limbs. The mean length of hospital stay was 14 days. Some 68 children (23%) required mechanical ventilation. The mortality rate was 16%.

**Conclusions:**

Burns in children are frequent, occur in the home in most cases (especially in the kitchen), and are accompanied by significant mortality. Prevention must be based on educating parents and children about dangers in the home.

**O3.8.8**
**The Operative Treatment of Pediatric Burns—A Tertiary Center Experience in a Cohort of 139 Children**
**Dorde Kravljanac** ^1,2^, Radoje Simic ^1,2^, Maja Sujica ^1^
^1 ^ Institute for Mother and Child Healthcare of Serbia, Clinic for Pediatric Surgery, Belgrade, Serbia
^2 ^ Medical Faculty, University of Belgrade, Belgrade, Serbia
**Aim:**

To evaluate the applied surgical procedures in burn treatments in our cohort over the last ten years.

**Methods:**

This retrospective study included pediatric burns patients treated via different surgical procedures under general anesthesia. Children were reviewed for age, sex, manner of injury, total body surface area (TBSA) of burn, degree of burn, type of surgical procedure, and length of healing.

**Results:**

The study included 139 children (71 boys, 69 girls) operated upon between 2012–2022. The mean age was 4.69 years (ranging from 1 month to 17 years). Most of these children were injured by hot liquids (62.59%), followed by flame burns (15.62%), contact burns (20 children), and electrical injuries (11 children). The mean TBSA of the burn was 9% (ranging from 1% to 50%). All patients had deep dermal or full-thickness burns. The performed procedures were necrectomy and skin grafting in 94.24% of cases, necrectomy with primary closure in four children, necrectomy with local flaps for closure of the burn area in two patients, and amputations of the hands and reconstruction of the wounds in two cases of severe electrical injury. The average time taken for the burn wound to heal after the operation was 15.32 (ranging from 7 to 59) days.

**Conclusions:**

Operative treatment of pediatric burns is usually reserved for severe cases wherein the burn wound involves the deeper layers of the skin. Early necrectomy and skin grafting are the gold standards for most children. The specific surgical approach will depend on the extent and severity of the burn injury, including amputations in the most severe patients.

**Saturday 9 September 8:30–10 am**
**Session: Wounds**
**O4.1.1**
**A Study of Using Medical Honey Dressings Compared to Staplers for Skin Graft Fixation in Burn Wounds**
**Sheyda Rimaz** ^1^, Mohammadreza Mobayen ^2^, Siamak Rimaz ^3^, Alireza Feizkhah ^4^

^1^  Burn and Regenerative Medicine Research Center, Guilan University of Medical Sciences, Rasht, Iran
^2^  Burn and Regenerative Medicine Research Center, Guilan University of Medical Sciences, Rasht, Iran
^3^  Anesthesiology Research Center, Department of Anesthesioly, Guilan University of Medical Sciences, Rasht, Iran
^4^  Burn and Regenerative Medicine Research Center, Guilan University of Medical Sciences, Rasht, Iran

**Aim:**

A critical element of skin grafts is the adherence of the graft to the wound bed. This study aimed to determine the effect of using medical honey compared to staplers in attaching skin grafts to burn wounds.

**Methods:**

In this clinical trial study, 80 patients with deep second- and third-degree burns with TBSA< 40% underwent skin graft surgery. Patients were randomly divided into two groups of 40 people, with one group using honey and the other a stapler for graft fixation. All wounds were opened on the fifth day of grafting. All patients in the two groups were evaluated for graft rejection, number of hospitalization days, transplanted skin displacement, graft contraction, pain (VAS score), edema, hematoma, itching (5D score), and infection rate.

**Results:**

A total of 80 patients were enrolled, with mean age of 39.29 ± 15.42 years; there were 34 men (42.5%) and 46 women (57.5%). No significant differences were observed in age, TBSA, mechanism of burn, and sex. The mean hospital stay was shorter (*p* = 0.034) and the infection rate, hematoma, edema, pain and itching severity (*p* = 0.000), were lesser in the honey group. The graft contraction rate was lower in the stapler group (*p* = 0.031). Graft rejection was not observed in any group.

**Conclusions:**

The results showed that natural honey is a very effective agent for split-thickness skin graft fixations in burn patients, and reduces the negative aspects of skin grafts such as duration of hospital stay and adverse effects such as pain and itching severity, edema, hematoma, and infection rate.

**O4.1.2**
**Management and Outcomes of SJS/TEN in a Regional Burns Unit in Northern Ireland**
**Lydia De Carvalho**, Abid Rashid, Claire Black, Nicholas Hodgins, Brendan Fogarty, Shakeel Dustagheer, William Beswick
The South Eastern HSC Trust, Belfast, UK
**Aim:**

To review local evidence of the management and outcomes of patients with Stevens–Johnson syndrome (SJS) and toxic epidermal necrolysis (TEN) in a regional burns unit.

**Methods:**

Local records of the regional burns unit in Belfast were reviewed retrospectively for patients who had a diagnosis of Stevens–Johnson syndrome and toxic epidermal necrolysis during 2022–2023. Medical and operational notes were evaluated for age, total body surface area affected, type of dressing applied, and healing outcomes as well as morbidity.

**Results:**

Recent local evidence suggests early debridement of loose epidermis, deroofing blisters, and application of Biobrane^®^.

**Conclusions:**

SJS and TEN are rare and severe skin disorders; UK guidelines suggest blisters are decompressed and the epidermis is left in situ. Local evidence has found success with a more aggressive approach involving debridement and application of biosynthetic skin dressings.

**O4.1.3**
**The Efficiency of Enzymatic Debridement Combined with Negative Pressure Wound Therapy for Deep Burns Treatment—A Clinical Study**
**Camelia Tamas** ^1^, Dan Cristian Moraru ^1^, Clara Larisa Ibanescu ^1^, Irina Mihaela Jemnoschi Hreniuc ^2^, Angela Tecuceanu^2^
^1 ^ Umf Gr. T. Popa Iasi Plastic Surgery Department, Iași, Romania
^2 ^ Umf Gr. T. Popa Iasi Anatomy Department, Iași, Romania
**Aim:**

To evaluate the efficiency of enzymatic debridement using bromelain powder combined with negative pressure wound therapy in the treatment of second- and third-degree burns.

**Method:**

Our retrospective study analyzesd 24 patients (14 men) hospitalized in the Burns Unit of “Sf. Spiridon” Emergency Hospital, Iasi, over a 5-year period (2019–2023).

The patients included in our study suffered thermal (20 cases) or electric (4 cases) injuries, with a total burn surface area ranging from 8 to 45% TBSA. We applied enzymatic debridement therapy within the first 3–5 days after the injury, and we evaluated the burn wound depth before and after the debridement using a laser doppler perfusion imaging system.

We combined enzymatic debridement with negative pressure wound therapy (NPWT) for wounds located on the limbs and trunk (12 cases). For the rest of the patients (12 cases), we associated the enzymatic debridement with local applications of ointments based on low-molecular-weight hyaluronic acid (LMW-HA) in IIA burns (5 cases). For IIB and III burns, we used hyaluronic acid silver powder spray or antibacterial dressings based on dialkylcarbamoyl chloride (7 cases), aiming to remove the bacterial biofilm from the wound surface.

**Results:**

The survival rate was 83.33% (20 patients). The daily wound healing rate for the NPWT group was higher (3.25–2.86% wound surface) compared with the second group (2.64–1.98%).

**Conclusions:**

The combined effect of enzymatic debridement of the burned tissue and NPWT can reduce wound healing time and the hospitalization period.

**O4.1.5**
**Nexobrid Off-Label Use in the Elderly in Vall d’Hebron Burns Unit**
**Alexander Lugilde Guerbek**, Jordi Serracanta Domenech, Joan-Pere Barret Nerin
Vall d’Hebron Hospital, Barcelona, Spain
**Aim:**

We aimed to evaluate the off-label use of Nexobrid enzymatic debridement in the elderly population.

**Methods:**

In our case-report study, we included 42 patients aged 66–94 years with second-degree deep and third-degree burns treated with Nexobrid (NXB) in our unit. We defined age, sex, etiology, total burned surface (TBS), NXB-treated surface, time to Nexobrid use, hospital stay, mortality, infection, time to healing, need for surgery, escharotomy, and transfusion requirements as our primary outcomes.

**Results:**

Most patients were male, and the most common cause for burns were flames. Their mean age was 74.5. The average TBS was 17.74%, with an average NXB-treated surface of 7.49% and a time-to-NXB average of 1.2 days. The average hospital stay was 28.8 days. Mortality was found to be 23.8%. Infection rates were 26.2%, while the need for surgery, escharotomy, and transfusions was 76.2%, 0%, and 23.8%, respectively. Time taken to heal averaged 70.35 days.

**Conclusions:**

The findings suggest that Nexobrid use may result in minimal blood transfusion and a decrease in infection rates in the elderly population, which could lead to a decrease in complications and length of hospital stay. Although the study found a higher mortality rate, this was likely due to the fragility and burn severity, rather than the use of Nexobrid. Finally, the study’s findings contribute to the limited literature on Nexobrid use in elderly patients, providing initial insights into its effectiveness; however, further research is needed to confirm its safety and efficacy.

**O4.1.6**
**Facial Burns: A Refined Clinical Pathway of Conservative Treatment**
**Albin Stritar**, Luka Emeršič, Klemen Lovšin
UMC Ljubljana, Ljubljana, Slovenia
**Aim:**

Full-thickness facial burns present a serious injury of a head with the possibility of thermal trauma damage to the nasal pyramid, eyes, and ears. Deep burns are treated surgically with a delayed necrectomy (prof. Janžeković). At the same time, interventions such as tracheostomy, tarsorrhaphy, and immediate excision of exposed cartilage are indicated.

**Methods:**

Superficial and mid-dermal burns are mostly treated conservatively. According to the protocol, we applied gauzes and changed wet dressings over the first 3 days after the burn injury. Later, we switched to treatment with ointments or special dressings. In our practice, we have also had a lot of good results with treatment with silver sulfadiazine (Dermazin) cream, antibiotic ointments, medical honey, and later with hydrocolloid ointments.

**Results:**

In recent years, we have been using hydrogel (Microdacyn, Prontosan X), applied once a day or every two days after toileting, with saline solution. The application is sterile, while hydrogel is completely harmless to mucous membranes and eyes. The pharmacodynamic action of the gel is beneficial to the facial region. Occasionally, we combine the application with another special dressing to cover. In our experience, the best results are achieved when using nanocellulose preparations (Epiprotect, epicitehydro, FibDex). Both applications have proven to have clinical synergy.

**Conclusions:**

We have achieved great aesthetic and functional outcomes by using proper treatment plans according to local burn wound status. Minimal negative consequences have been observed for superficial or mid-dermal burns in children and adults. The regeneration process does not result in scarring and residuals.

**O4.1.7**
**Epicite (Nanocellulose, Biotechnologically Manufactured) as a First-Line Dressing, followed by EPIFAST (Cryopreserved Cultured Keratinocyte Sheets Able to Release Growth Factors) in Second-Degree Burns**
**Pablo Rodriguez-Ferreyra**, Teresa-Robles Narvaez, Omar Gayosso-Ceron
Instituto De Salud Del Estado De Mexico, Toluca, Mexico

Second-degree burns mostly coexist with superficial and deep areas, and even with third-degree areas; however, the treatment for each area will be different, and we must also consider that a delay in the coverage of the injury can cause desiccation and deepening of the lesion.

**Aim:**

To evaluate of the use of Epicite as a first-line dressing in the first 24 h post-burn, and Epifast (nanocellulose biotechnologically manufacture) for residual areas.

**Materials and Methods:**

Patients with scald burns who arrived for care in the first 24 h post-burn were included; gentle cleaning with saline solution was performed, and Epicite was applied. The area was covered with Vaseline, gauze, and bandage, the lesions were reviewed on the fifth day, and tangential scarectomy of residual eschars and application of Epifast were performed. Finally, the lesions were evaluated after 10 days of evolution.

**Results:**

The 25 patients, with an average age of 4.2 years and who met the inclusion criteria, had average TBSA of 14% initially; after the use of Epicite, the residual area was on average 5% of the TBSA. Upon the final evaluation, total epithelialization was observed, without the need for biotechnologically manufactured nanocellulose. There were no infections or side effects.

**Conclusions:**

The use of Epicite (a nanocellulose biopolymer) as a first-line dressing in the former prevents the drying of the wound and helps the epithelialization of the most superficial areas; with the help of a biological dressing donor of growth factors as a complementary therapy, it is an excellent option for the management of second-degree burns.

**Saturday 9 September 8:30–10 am**
**Session: Organizational Standards and Mass Casualties**
**O4.2.1**
**Major Burn Simulation Training: Setting up a Regional Course**
**Samar Mousa**, Maksim Prokopenko, Mohamed Eldolify, Lorna Murray, Miss Alexandra Murray
Buckinghamshire NHS Trust, Aylesbury, UK
**Aim:**

To establish a major burn simulation course to support the delivery of quality burns care across the Thames Valley region.

**Methods:**

This is a before-and-after questionnaire-based study describing the set-up and introduction of a simulation course in managing acute burns. The title is abbreviated to ‘SOBS’: the Stoke Mandeville and Oxford burn simulation course.

The course content complies with existing standard frameworks including the ‘Emergency Management of Severe Burns’ course, and supports learning particularly for trainees who lost training opportunities through the COVID pandemic. We aim to deliver the course three to four times annually, with costs kept to a minimum for attendees. Our target audience includes clinicians, nurses, and advanced-clinical-practitioners across emergency and surgical care services who are likely to encounter burn injuries.

This is a one-day course using high-fidelity simulation to recreate scenarios encountered during acute burn management. Our course features skills training such as how to carry out an escharotomy using a realistic, validated model, and the debridement and dressing of burn wounds.

**Result:**

Two iterations of the course have been conducted for plastic surgery and anaesthesia trainees. The received collated feedback describes the course as useful (84%), meeting expectations (80%), and producing high overall satisfaction (90%). The feedback comments received were positive, e.g., “really useful teaching, filling a big gap in my knowledge. Would recommend”.

**Conclusions:**

Simulation courses are an effective and innovative way to train healthcare professionals to manage major life-threatening burns cases, allowing learning in a safe environment, maintaining patient safety, and enhancing learning opportunities.

**O4.2.2**
**Assessing the Activity of the French Military Burn Center during the COVID-19 Pandemic 2019–2022**
**Nicolas Donat**, Julie Renner, Matthieu Laurent, Thibault Baudic, Thomas Leclerc
Burn Center, Percy Military Hospital, Clamart, France
**Aim:**

The intensity and duration of COVID-19 crisis undermined the French healthcare system and exposed major logistical and human resource difficulties. We assessed burn care activity at the HIA Percy Burn Center during this period.

**Methods:**

The number of beds available for burn intensive care (BICU) and medico-administrative codes related to burn care for the burn unit were collected from 2019 to 2022.

**Results:**

In 2019, 12 BICU beds were operated and 133 patients treated.

From 2020 to 2021, the available beds varied from six (first wave) or nine (second, third waves) to twelve. In 2022, only nine beds were opened. Ìn 2020, 107 burnt patients were treated, 108 in 2021, and 100 in 2022.

TBSA repartition were similar.

**Conclusions:**

2019 is the benchmark year. Year 2020 was marked by COVID-19 outbreak. The first wave led to a reorganization of critical care within the hospital; the BICU was reduced to six beds in order to free up space and staff to increase capacity. The second and third waves of COVID-19 led to a similar reduction in burn capacity to nine beds. In 2022, the shortage of paramedical staff, especially nurses, led to the closure of three beds. This situation had the worst impact as the bed-reduction was permanent. Difficulties observed in this study are consistent with international experience [1].

Overall, BICU activity was reduced during the COVID pandemic, and was not restored. This is a major concern to ensure burn care availability and skilled burn teams.

**References:**

[1] Laura, P. Impact of COVID-19 on global_burn_care. *Burns* **2022**, *48*, 1301–1310.

**O4.2.3**
**Survey on the Present Burn Care Facilities and a Retrospective Analysis of Burn Injury Incidence in Greece: Data from a Multicenter Study, Stressing the Need to Initiate a National Burn Registry**
**Sofia Papadopoulou**, Eirini Nikolaidou, Kristallo Makarona, Theodora Ligomenou, Zoi Tzimorota
“G. Papanikolaou” Hospital, Thessaloniki, Greece, Thessaloniki, Greece
**Aim:**

The purpose of this observational, multi-center study was to collect data on burn care facilities and to estimate burn injury incidence, as well as the epidemiologic, etiological and clinical aspects of adult hospitalized burn patients in Greece.

**Methods:**

A two-part questionnaire was e-mailed to 13 burn units and plastic surgery departments in Greece. The first part included questions on personnel, facilities, equipment and practices. The second part retrospectively collected demographic, clinical and outcome data of hospitalized burn patients in a set 1-year period.

**Results:**

Of the 13 hospitals, 11 responded (85%). A total of 12 Burns ICU beds were available in dedicated burn units, whereas in three more hospitals, intubated burn patients were treated in the General ICU. From 2 to 8 consultant plastic surgeons work in each center with 0 to 14 residents. There is usually collaboration between plastic surgeons and intensivists on critical care, infection control, nutrition.

Regarding the second part, the 11 centers reported 577 patients (55% men) with an average TBSA 26%. Their average age was 57 years and the most frequent burn types were thermal burns (82%), with chemical and electrical burns representing 4.5% each. The overall mortality reported was 10%.

**Conclusions:**

This pilot questionnaire showed the variability of burn care between centers in Greece and the difficulties of retrospectively collecting data not digitally recorded prospectively. We stress the importance of a National Burn Registry in seeing a precise picture of burn injury incidence and burn care needs, for evaluating our services, and for initiating and implementing targeted prevention programs.

**O4.2.4**
**Thirty-Six Years of the BABI Plan: The Accomplishments and Developments of the Belgian Mass Burn Casualty Disaster Plan**
**Serge Jennes**, Ghueder Saidane, François Debry
Grand Hôpital de Charleroi, Charleroi, Belgium
**Aim:**

To present the lessons learned from the implementation of the BABI plan over the last 36 years.

**Methods:**

We have reviewed six burn disasters that have struck Belgium since the creation of the Belgian Association for Burn Injuries (BABI) in 1987. One of the goals of this association is to maintain and update a coordination plan for burn beds in case of mass casualty incident (MCI) with many burn victims. The goals of the plan are as follows: (1) the rapid and effective warning of all Belgian burn centers (BBC) and an increase in their capacity in beds; (2) to provide a rapid medical response at the place of the incident or in evacuation hospitals, mainly triaged by a B-Team; (3) the organization of the distribution of the victims through the different BBC; and (4) to contact BCs in neighboring countries in the search of burn beds in case of a disaster overwhelming national treatment capacities.

**Results:**

We reviewed the following disasters: (1) the auditorium attack in Brussels in 1990; (2) the Switel hotel fire in Antwerp in 1995; (3) the café fire in Volendam in 2001; (4) the Cockerill explosion in Liège in 2002; (5) the gas pipeline explosion in Ghislenghien in 2004; and (6) the Brussels terror attacks in 2016. There were in total 701 injured; 264 were hospitalized and 75 died (58 outright).

**Conclusions:**

The BABI plan has been efficient in terms of speed of implementation and distribution and evacuation of victims and cross-border cooperation.

**O4.2.5**
**From RE-ENERGIZE to VICToRY: Buildling the First International Consortium for Clinical Trials in Burns**
Kaitlin Pruskowski, **Leopoldo Cancio**, Maureen Dansereau, Christian Stoppe, Daren Heyland
United States Army Institute of Surgical Research, JBSA Fort Sam Houston, USA
**Aim:**

Conducting randomized controlled trials (RCTs) to improve burn care is challenging. To address this challenge, a large, international, multicenter consortium was formed. This unprecedented consortium completed an RCT of glutamine therapy in burns, and is now conducting a RCT of moderately high-dose vitamin C. This abstract will review the logistics of implementing this program of research.

**Methods:**

RE-ENERGIZE (“A Randomized trial of ENtERal Glutamine to minimIZE thermal injury”) was a double-blind RCT that evaluated enteral glutamine initiated within 72 hrs of admission. VICToRY (“VItamin C in Thermal injuRY”) is a double-blind RCT that is evaluating intravenous vitamin C initiated within 24 hrs of admission. All research is approved by local ethics committees/institutional review boards.

**Results:**

RE-ENERGIZE:A total of 125 sites in 38 countries were engaged; 54 sites in 14 countries enrolled patients. In the pilot/feasibility phase, the enrollment rate was 0.96 patients/site/month (range 0.68–1.31), and this decreased to 0.44 patients/site/month (range 0.07–1.74). A total of 1209 patients were enrolled.

VICToRY: A total of 138 sites in 32 countries have been engaged; 11 sites in 4 countries have been activated; and 44 sites in 14 countries are projected to be activated by summer 2023. The enrollment rate is 0.4 patients/site/month (range 0.2–1.0).

**Conclusions:**

RE-ENERGIZE demonstrated the feasibility of international RCTs to improve burn care. The RE-ENERGIZE network is now being leveraged in the VICToRY trial. The key to success is developing and maintaining strong, positive relationships with international partners, and assisting them in overcoming obstacles unique to their environments.

**O4.2.6**
**Impact of Decreasing Burn Bed Capacity on Non-Admission Rates: A 7-Year Retrospective Study in a French Military Burn Centre**
**Nicolas Donat**, Julie Renner, Jean-Vivien Schaal, Matthieu Laurent, Clément Hoffmann
Burn Center, Percy Military Hospital, Clamart, France
**Aims:**

Over recent years, decreasing healthcare staffing has led to decreasing bed capacity in French burn centres, further impacted by temporary reductions due to XDR bacteria importation or to the re-allocation of burn ICU beds to COVID-19 care. This study aims to assess how this has impacted the capacity of our military burn centre to answer the population’s needs.

**Methods:**

The electronic registry of all medical calls to our burn centre was retrospectively analysed from March 2016 (registry opening) to April 2023 and compared with open burn bed capacity. Summary data, expressed as mean per full year, included total calls, admission requests, and rebuttals (for lack of available bed and for absence of indication). Burn bed capacity changes with time and association between burn bed capacity and percentage of admission rebuttal for lack of beds were analysed using linear regression.

**Results:**

Over 7 years, our burn centre received 452 ± 45 calls each year (a yearly increase of+16 calls, *p* = 0.004), of which 208 ± 15 were admission requests (showing no significant upward trend). The yearly mean number of open beds decreased from 13 to 9 (representing a yearly decrease of -0.5 beds, *p* = 0.003). The yearly admission rebuttal rate was 39 ± 2.5% among definite indications (38% with 13 open beds, 42% with 9, variation NS).

**Conclusions:**

Facing similar capacity limitations as civilian burn centres, our centre declined four out of ten admission requests with definite indication, showing an unclear upward trend. A national registry would help to assess whether corresponding patients finally access specialised burn care, thereby calculating the national burn care capacity.

**O4.2.7**
**The Influence of Electron Beam Irradiation on the Extracellular Matrix of Human Allogeneic Skin Grafts**
**Wojciech Łabuś**, Przemysław Strzelec, Karolina Ziółkowska, Agnieszka Klama-Baryła, Artur Wielgórecki
Stanisław Sakiel Centre For Burn Treatment In Siemianowice Śląskie, Siemianowice Śląskie, Poland
**Aim:**

Non-viable allogeneic human skin grafts may be considered the most suitable skin substitutes in the treatment of extensive and deep burns. However, in accordance with biological security, such grafts require a final sterilization prior to clinical application. The aim of the study was to verify the influence of electron beam irradiation in three selected doses, 18 kGy, 25 kGy, and 35 kGy, on the extracellular matrix of human skin.

**Methods:**

Prior to sterilization, the microbiological tests were conducted and revealed contamination in all examined cases. Individual groups were subjected to single electron beam radiation sterilization at proposed doses, and then subjected to microbiological tests again. The results of microbiological testing performed for all irradiation doses used were negative. Only in the control group was a growth of microorganisms observed. FTIR spectrometry tests were conducted, followed by histological evaluation and mechanical tests. In addition, a cost analysis of the radiation sterilization of individual doses was performed.

**Results:**

The results of spectroscopic analysis, mechanical tests and histological staining showed no significant changes in the composition and characteristics of the tested tissues after their irradiation, in comparison to the control samples. The cost analysis showed that irradiation with 18 kGy is the most cost-effective, and 35 kGy is the least favorable.

**Conclusions:**

According to biological risk reduction, the recommended sterilization dose is 35 kGy, despite the higher price compared to the other doses tested.

**Saturday 9 September 8:30–10 am**
**Session: Critical Care and Anesthesia**
**O4.3.1**
**Non-Invasive Physical Plasma of Helium (Generating Highly Reactive Oxygen and Nitrogen Species) Application as an Adjuvant Treatment to Epifast for Superficial and Deep Second-Degree Burns**
**Pablo Rodriguez-Ferreyra** ^1^, Regulo Lopez-Callejas ^2^, Teresa Narvaez-Robles ^1^, Omar Gayosso-Ceron ^1^

^1^  Instituto De Salud Del Estado De Mexico, Toluca, Mexico
^2^  Instituto Nacional de Investigaciones Nucleares, Mexico

**Aim:**

To demonstrate that non-thermal plasma or non-invasive physical plasma (NIPP) as adjuvant therapy to Epifast help to improve the healing process in patients with second-degree burns.

**Methods:**

NIPP of helium generates highly reactive oxygen (hydroxyl radicals (·OH)) and antiseptic and nitrogen species (nitrogen dioxide (NO2)), which are anti-inflammatory. The patients were divided into the following groups: Group 1, who underwent scarectomy in the first 48 h after burn and wound coverage with Epifast, and evaluation of the wound after 5–6 days; and Group 2, who received application of NIPP directly over the wound during the first surgery, coverage with Epifast, NIPP application every 24 h, and evaluation of the wound after 5–6 days.

**Results:**

A total of 40 patients were included, divided into 20 (Group 1), and 20 (Group 2). The total body surface burn (TBSB) was 12.60%(+−) 8.2 for Group 1 and 12.55%(+−) 8.06 for Group 2. There were no adverse effects. DOS, and the use of analgesics and antibiotics were slightly less in the experimental group, although without significant statistical difference. Regarding the grafted patients, the experimental group fared better, since only two patients (10%) were grafted. In comparison, grafts were necessary for eight patients (40%) from the control group, with a relative risk (RR) of 0.25 and a 95% confidence interval (CI) ±0.06–1.0 (*p* = 0.02).

**Conclusions:**

The use of NIPP as an adjuvant therapy of the Epifast reduces the need for graft placement in burn patients by 75%, and there was no incidence of adverse effects; this demonstrates its efficacy and safety for the treatment of superficial and deep second-degree burns.

**O4.3.2**
**A Retrospective Analysis Study: Enzymatic Debridement (ED) in Large Burn Patients**
**Jasminka Minic, Enrico Vigato**, Maurizio Governa
Azienda Ospedaliera Universitaria Integrata Verona, Verona, Italy
**Objective:**

Early surgical debridement is mandatory in treatment of patients with massive burns. Such a procedure is not always possible due to the complexity of the patient, their general condition, hemodynamic stability, and the availability of hospital and human resources. The objective of the study was to evaluate the use of early bromelain-based debridement on a patient population with up to 15% TBSA.

**Method:**

We conducted a retrospective study collecting consecutive adult large burn patients with up to 15% TBSA admitted to Verona Burn Center from 2017 to 2021 and treated with bromelain-based ED. The study sample consisted of 65 subjects treated with Nexobrid (ED). The study was approved by the Institutional Review Board (Number 2214CESC); informed consent was waived due to the retrospective nature of the study. The analysis was carried out using Stata MP17 software.

**Results:**

A total of 65 cases were treated with Nexobrid^®^. The average TBSA was 35.29. The maximum treated area was 70% TBSA. The mean time for debridement was 1.6 days. The mean auto-grafting amount of was 9%; 12 out of 65 patients had sepsis. The average length of hospitalization was 44.21 days. ED was applied at the bedside in 42 cases, and in 13 cases in OR. Only 6 out of 65 cases needed a blood transfusion.

**Conclusions:**

Our protocol could prove a feasible additional alternative to surgical excisional debridement and its associated drawbacks. The data demonstrate significantly reduced blood loss, improved dermal preservation, a reduced need for autografting, a reduction in the number of OR theatre procedures, and the protocol’s potential safety.

**O4.3.3**
**Utilizing Unsupervised Clustering and Latent Class Analysis to Evaluate Clinical Heterogeneity and Predict Mortality in Severely Burned Patients: A Retrospective Cohort Study**
**Jaechul Yoon** ^1^, Dohern Kym ^2^, Jun Hur ^3^
^1 ^ Hangang Sacred Heart Hospital, Hallym University, Seoul, Republic of Korea
^2 ^ Hangang Sacred Heart Hospital, Hallym University, seoul, Republic of Korea
^3 ^ Hangang Sacred Heart Hospital, Hallym University, seoul, Republic of Korea

Burn injuries have clinical heterogeneity and poor prognosis in severely burned patients. Clustering algorithms can provide insight into the mechanisms of disease pathogenesis.

The aim of study was to analyze collected biomarkers in order to understand mortality prediction power, identify clinical meanings or subtypes, and inform treatment decisions in order to improve outcomes.

This retrospective cohort study included patients who were admitted between January 2010 and December 2021. These patients were divided into four subgroups depending on the time of their admission: weeks 1 to 4.

**Results:**

In this study, 22 biomarkers were evaluated, with RDW, bicarbonate, pH, platelets, and lymphocytes being significantly associated with mortality risk. A latent class analysis further demonstrated that pH, platelets, lymphocytes, lactate, and albumin were the worst in the cluster, with the highest risk of mortality; pH and lactate were particularly noteworthy in the first week. During the second week, pH and lymphocytes were found to be significant predictors of mortality risk, while lymphocytes and platelets were meaningful predictors in third week. In the fourth week, pH, platelets, and albumin were considered predictors of mortality risk.

**Conclusions:**

This analysis of biomarkers using clustering algorithms and a latent class analysis can provide valuable insights into the heterogeneity of burn injuries, and improve our ability to predict disease progression and mortality. Our findings suggest that lactate is a indicator of cellular hypoxia in the early stages of shock, while platelets and lymphocytes are indicative of infection. Albumin is a indicator of reduced nutrition, and pH reflects the positive condition of the patient.

**O4.3.4**
**The Use of a High-Flow Nasal Cannula for Critical Burn Patients during Deep Sedation in Enzymatic Bromelain Debridement (Nexobrid^®^): a Preliminary Report**
**Crescenzo Sala**, Francesco Coletta, Maria Notaro, Adele Longobardi, Antonio Tomasello
Burns Intensive Care Unit and Poison Control Center, Naples, Italy
**Object:**

The use of new oxygen supports associated with non-invasive respiratory strategies is well-established in clinical practice, especially after the technique’s extensive application in the management of COVID-19 respiratory failure. The use of a high-flow nasal cannula (HFNC) in patients undergoing procedural sedation and analgesia (PSA) is dramatically increasing. Enzymatic debridement in critical burn patients is a painful treatment that requires an optimal burn pain control protocol as well as deep sedation for the entire duration of the procedure. Both hypnosis and opioid analgesia may lead to significant respiratory depression.

**Methods:**

Fourteen patients undergoing enzymatic debridement under deep sedation were included in this case study. All patients receiving oxygen through HFNC were evaluated. All patients underwent continuous monitoring of vital parameters, antithrombotic prophylaxis with low molecular weight heparins, and fluid therapy calculated using the Parkland formula.

**Results:**

Sedation was successful and well tolerated by all patients, and physicians were able to carry out the enzymatic debridement procedures safely. No severe desaturation events were observed. Continuous monitoring of vital signs was carried out. Neither bradycardia events nor hypotensive or hypertensive events requiring treatment occurred.

**Conclusions:**

Enzymatic debridement procedures did not lead to any serious adverse events. Based on our experience, the administration of O2 by HFNC at an average concentration of 50% was proven safe and efficacious in the management of drug-induced respiratory depression.

**O4.3.5**
**Severe Methemoglobinemia after Using a Local Anesthetic on a Child with a Burn.**
**Serhii Yehorov** ^1^, Artem Posunko ^1^, Alexey Vlasov ^1^, Gennadiy Fuzaylov ^2^
^1 ^ Regional Medical Center for Family Health, Dnipro, Ukraine
^2 ^ Massachusetts General Hospital, Boston, USA
**Introduction:**

The objective of this case report is to present a patient with acquired methemoglobinemia due to poisoning resulting from off-label use of local anesthetics. This toxic effect leads to the disruption of the processes of oxidative phosphorylation in erythrocytes, with the formation of methemoglobin incapable of oxygen transport.

A 10-year-old boy suffered flame burns with a TBSA of up to 60%. He was treated in the ICU of the regional children’s hospital. The child’s general condition acutely deteriorated with central cyanosis, severe tachycardia, arterial hypotension, and a decrease in SpO_2_ to 76% on day six. During systematic examination and laboratory evaluations of the child’s hypoxemia, we decided to analyze his levels of methemoglobin, which turned out to be positive and amounted to 22%. To establish the cause of this, we conducted a complete analysis of the medications that the patient received. The source of intoxication appeared to be an ointment that was used twice a day on a burned surface as a local wound treatment, which contained prilocaine.

**Results:**

Methemoglobinemia symptoms result from inadequate oxygen transport. For the treatment of this condition, methylene blue was used at a dosage of 2 mg/kg, with an extended infusion over 30 min. There was a gradual regression of intoxication, lactic acidosis, and hypoxemia, with complete stabilization of vital functions within 2.5 h of the introduction of the antidote.

**Conclusions:**

Our case highlights the need for in-depth routine monitoring of local anesthetics used on burns in children.

**O4.3.6**
**Bench-to-Bedside Assessment: High-Dose Vitamin C Therapy in Burn Patients with Septic Shock**
**Amel Mokline**, Rayan Nachi, Hana Fraj, Mariem Gargouri, Amen Allah Messadi
Trauma and Burn Center, Tunis, Tunisia
**Aim:**

To assess the impact of high-dose vitamin C (100 mg/Kg/d) in septic burn patients in terms of fluid resuscitation, dose, and duration of catecholamines.

**Methods:**

This was a case–control study conducted in an intensive burn care department in Tunisia over 26 months. We included adult with burns presenting sepsis or septic shock. We excluded pregnant woman and patients under long-term vitamin C therapy. After inclusion, ascorbic acid was prescribed at a dose of 100 mg/kg/day over 4 days. The vitamin C group was compared with a retrospective group (non-vitamin C) from the same center, matched in terms of age, sex, extent and burn severity. The therapeutic management of sepsis was similar for the two groups, with the same hemodynamic objectives (hourly output at 0.5 cc to 1 cc/kg and MAP > 65 mmHg). Results: Some 100 patients were included and divided into two groups: G1 (Vit C+; n = 50) and G2 (Vit C−: n = 50). The patients of the two groups were comparable in terms of sex, age, and severity of burns. Administration of vitamin C, fluid balance (2 mL/Kg/day for G1 vs. 13 mL/kg/day for G2; *p* = 0.008), and doses of noradrenaline (1.8 mg/h vs. 3.5 mg/h; *p* = 0.01) reduced at day 3, and the duration of noradrenaline dependance (4 days for G1 vs. 4.84 days for G2; *p* = 0.28) shortened. Conclusions: High-dose vitamin C therapy was associated with reduced fluid balance, doses of noradrenaline, and duration of dependance in septic shock burns within the first 3 days of sepsis.

## 7. Poster Presentations

**P001**
**Mortality Data of Adult Burns in Single Burn Unit over the Last 17 Years**
**Julia Bartkova** ^1,2^, Dušana Selecka ^2^, Bretislav Lipovy ^1,2^
^1 ^ Department of Burns and Plastic Surgery, University Hospital Brno, Czech Republic, Brno, Czech Republic
^2 ^ Faculty of Medicine, Masaryk University Brno, Czech Republic, Brno, Czech Republic
**Aim:**

The aim of study was to determine the basic epidemiological characteristics of adult mortality data from a single burns unit from the year 2005 to 2022.

**Methods:**

We collected and evaluated data such as age, sex, day and month of injury, manner of burning, burn etiology, extent of the burned area, length of hospitalization, cause of death, and the presence of inhalation trauma.

**Results:**

In the present data, there were 113 deaths due to burn injury. Of these, 68 were male and 45 were female, with an M:F ratio of 1.5:1. The average age was 67.5 years. Seasonal variations showed that deadly incidences of burns occurred mostly in winter 28%, with an indoor to outdoor ratio of 4.9:1. The incidence was more frequent during weekdays (70.8%). The highest incidence for males was on Saturday, and for females was on Thursdays. Accidental burning was observed in 92% of patients. The most common causative agent was flame (72.6%). The average burn extent in all cases was 47% TBSA. The average hospital stay was 9.8 days. Multiple-organ failure was the leading cause of death, at 62%, followed by burn shock, at 29%. In 49% of patients, the condition was complicated by inhalation injury.

**Conclusions:**

We recorded a significant decrease in mortality; in particular, in the last 5 years, we recorded 24 deaths, which is 21% of the total number of burn deaths in the last 17 years. These results point to the constantly improving quality of care in medical burn facilities, and the continuous improvement of their prevention.

**P002**
**Fibrin Spray Delivery of Mesenchymal Stem Cells: An In Vitro Study to Assess Feasibility in Burn Wound Treatment**
**Astrid Bjørke Jenssen** ^1,2^, Samih Mohamed-Ahmed ^3^, Esko Kankuri ^4^, Kamal Mustafa ^3^, Stian Kreken Almeland ^1,2^
^1 ^ Norwegian National Burn Center, Departmet of Plastic, Hand and Reconstructive Surgery, Haukeland University Hospital, Bergen, Norway
^2 ^ Department of Clinical Medicine, Faculty of Medicine, University of Bergen, Bergen, Norway
^3 ^ Center for Translational Oral Research (TOR), Tissue Engineering Group, Department of Clinical Dentistry, Faculty of Medicine, University of Bergen, Bergen, Norway
^4 ^ Department of Pharmacology, Faculty of Medicine, University of Helsinki, Helsinki, Bergen
**Aim:**

Although limited data are available for evaluation of the clinical efficacy of various cellular therapies for burns, treatments using mesenchymal stem cells (MSCs) have shown promise for accelerating healing. The aim of this work was to investigate the in vitro feasibility of a fibrin spray as a vehicle for topical administration of MSCs burn wounds.

**Methods:**

Human bone marrow-derived MSCs (hBMMSCs) were expanded in vitro and used at passage 4. Tisseel (Baxter, IL, USA), a commercially available two-component fibrin glue, was used and diluted for easier administration with spray. Cells were added to the fibrinogen component, which was mixed with the thrombin component and then casted or sprayed into a non-adherent 60 mL Petri-dish using an EasySpray pressure regulator device (Baxter). Spraying, using either 0.5 or 1 bar pressure, was carried out at a 5–10 cm distance from the well. Cell viability was evaluated using live/dead staining. Cell morphology was investigated with phalloidin staining. Cell proliferation was monitored for up to 7 days using a PrestoBlue assay.

**Results:**

Live/dead staining demonstrated the survival of hBMMSCs after casting or spraying, as confirmed by good cell viability after application. For both casted and sprayed groups, cells were well distributed and well spread within the fibrin vehicle. The applications did not affect cell proliferation. Increasing the pressure from 0.5 to 1 bar did not have a negative impact on the cells.

**Conclusions:**

Cell spraying in a fibrin vehicle maintains the viability of hBMMSCs and represents a promising method for applying mesenchymal stem cells to burn wounds.

**P003**
**The Use of Meek Micrografting in the Closure of Small-Area Defects in Burn Victims: A Case Report**
**Kim De Mey**, Ignace De Decker, Henk Hoeksema, Petra De Coninck, Jozef Verbelen, Karel Claes, Stan Monstrey
UZ Gent, Gent, Belgium
**Aim:**

For burn victims with a high total body surface area (TBSA) burn, skin expansion techniques are used to enlarge grafts. The most used of these is the mesh technique. However, when surgeons are confronted with extensive burns, the Meek micrografting method is preferred to ensure a more reliable, regular, and higher expansion rate of grafts. In smaller defects, the mesh technique still remains the gold standard, and it has been reported that the Meek technique for smaller defects can result in improved scar quality, as compared to mesh.

**Methods:**

A 51-year-old male was admitted to the burn center with a scald, and 38% TBSA. Loose skin was debrided, and the wound was treated with povidone iodine solution and dressed with an alginogel in combination with paraffin gauzes. Laser doppler imaging on the third day showed deep burns of the right lower leg; Meek was used. The patient received standard aftercare and was frequently seen in the outpatient scar clinic.

**Results:**

Surgical debridement was followed by allograft application. These were removed 10 days later, and autografting using the Meek technique (1:4) was performed. The Meek gauzes were removed 9 days post-operatively, and complete graft take was achieved. At 1.5 years post-burn, a mature scar can be seen, with no signs of hypertrophy or contracture present. Additionally, no functional limitations were observed in the target area or at the donor site.

**Conclusions:**

The Meek micrograft technique is an excellent option for the closure of relatively small defects, resulting in good graft take, and optimal aesthetic and functional outcomes.

**P004**
**Biologically Relevant Support for Primary Human Keratinocytes, Melanocytes and Fibroblasts Under Xeno-free and Chemically Defined Conditions**
**Mr Boris Eleuteri** ^1^, Malin Kele ^1^, Therese Kallur ^1,2^
^1 ^ BioLamina AB, Sundbyberg, Sweden
^2 ^ BioLamina Inc, Cambridge, USA
**Aim:**

To enable the next generation of safe and efficient skin expansion in vitro by replacing the current, undefined, and often serum-based alternatives by using recombinantly produced laminins for the major skin cells (keratinocytes, melanocytes, and fibroblasts).

**Methods:**

We used recombinantly produced laminins, biolaminins, as cell culture substrates. They provide a biologically relevant support to the major cell types of the skin, and were also from adult donors.

**Results:**

We demonstrate that the effect is laminin isoform-dependent, and provides superior support to the proliferative cells within the skin, also from aged donors, compared to current methods. For example, the use of biolaminin-521 in expanding adult primary keratinocytes resulted in 14 cumulative doublings compared to 8 under standard conditions after 30 days in culture. Furthermore, the use of biolaminins provides better support for the survival and growth of freshly isolated skin cells, resulting in a four times higher cellular yield.

**Conclusions:**

The use of biolaminins allows for a new generation of safe and efficient in vitro expansions of human keratinocytes, melanocytes, and fibroblasts, under completely xenofree and chemically defined conditions. We believe that this method of expanding adult human skin cells may be applicable to cell-based wound care, potentially also expanding its clinical uses.

**P005**
**Seasonal Trends in Burn Injuries Requiring Admission to a Regional Burn Center in Barcelona over a 5-Year Period.**
**Alejandro Grabosky**
Hospital Vall D’Hebron, Barcelona, Spain
**Aim:**

To examine the relationship between seasons and burn injuries requiring admission to a regional burn center in Spain over the last 5 years.

**Methods:**

We will carry out a retrospective review of all patients admitted to our institution from March 2018 to February 2023 (Hosptial Vall d’Hebron, Barcelona, Spain). Patients will be grouped into seasonal cohorts based on admission date. Using data from medical records, we will evaluate changes in patient demographics, mechanisms of injury, total body surface area (TBSA), and mortality. Afterwards, a statistical analysis will be performed.

**Results:**

Results will be presented by season and by year for each variable under study.

**Conclusions:**

What we will learn from this review could be used to better assess the seasonal demands in ours and other burn centers in the southern European region.

**P006**
**Mortality Rate and Related Factors among Inpatients with Burns in the University Hospital in Bangkok, Thailand.**
**Kittipob Kirdpun**, Anchan Ketmek, Pornprom Muangman
Department of Surgery, Faculty of Medicine Siriraj Hospital, Mahidol University, Bangkok, Thailand
**Aim:**

We aimed to determine trends in age- and sex-adjusted deaths among inpatients with burns in the University Hospital in Bangkok, Thailand, between 2017 and 2022. In addition, we also explored the in-hospital mortality rate and associated factors among inpatients with burns.

**Methods:**

A hospital-based retrospective cohort study was conducted in a trauma burn unit at a University Hospital in Bangkok, Thailand. We included inpatients with burns admitted between January 2017 and December 2022. We calculated the mortality rate and 95% confidence interval (CI). A generalized linear model was used to calculate a *p*-value for the trend. Furthermore, a multivariable analysis using the Cox regression model was employed to determine the factors associated with in-hospital morality. A *p*-value less than 0.05 was considered statistically significant.

**Results:**

A total of 240 inpatients with burns were included in the present study, which covered six years. Age- and sex-adjusted deaths were 6.9%, 11.5%, 2.6%, 6.0%, 3.4%, and 8.0% in 2017, 2018, 2019, 2020, 2021, and 2022, respectively (*p* for trend = 0.672). The median follow-up time was 26.5 days; the in-hospital mortality rate was 1.97 (95% CI 1.13–3.21) per 1000 person days. The adjusted hazard ratio (AHR) of in-hospital mortality among patients with burns of a higher age was 1.05 (95% CI 1.01–1.10). Patients with an increase of one percentage point in their total body surface area (TBSA) of burn had a 7% higher mortality rate. In addition, the AHR of in-hospital mortality among patients with fourth-degree burns was 9.00 (95% CI 2.22–36.57) times that of patients with less than fourth-degree burns.

**P009**
**Exploring the Role of Computer-Assisted Decision-Support Tools in Burn Management: A Rapid Review**
**Aqeel Mohamed** ^1^, Mevhibe Hocaoglu ^2^
^1 ^ King’s College London, GKT School of Medical Education, London, UK
^2 ^ Cicely Saunders Institute of Palliative Care, Policy & Rehabilitation, King’s College London, London, UK
**Background:**

The biopsychosocial impact and need for intricate care make burn management and recovery complex, while also being a global public health issue (particularly in low- to middle-income countries). Computer-assisted decision-making tools (CADTs) are used in different clinical scenarios, yet their role in burn management seems limited. The multidimensional impact on the quality of life of patients and those important to them warrants palliative and end-of-life care in burn management.

**Aim:**

To outline the current uses of CADTs in burn management.

**Methods:**

The review followed the PRISMA flow diagram. Studies were extracted from PubMed using the following keywords: (decision making, computer-assisted) AND ((burn units) OR (ICU) OR (intensive care unit) OR (dermatology) OR (emergency medicine)) AND (burns)

The retrieved records were screened based on titles, abstracts, full text access before analysis. The Hawker critical appraisal tool was used for quality assessment.

**Results:**

A total of 13 papers were included in this review. The majority (54%) discussed new software, showcasing the field’s novelty. However, most studies gave fewer specifics regarding average participant age, burn severity, gender, and ethnicity. Using the Hawker critical appraisal tool, we found that the range of scores in the review varied from 21 to 36, with the average being 30/36, suggesting scope for future research improvement.

**Conclusion:**

Burns are a public health issue, and would benefit from palliative care interventions given the biopsychosocial impact they have on patients and those important to them. Further investigation of this impact on diverse populations (e.g., different age groups, burn severity levels, and ethnicities) is critical in translating CADTs into clinical practice.

**P012**
**Identification of Blood-Based Signatures for Inhalation Injury Prognosis in Burns Patients**
**Tarryn Prinsloo** ^1^, Wayne Kleintjes ^2^, Tandi Matsha-Erasmus ^1^, Kareemah Najaar ^1^
^1 ^ Cape Peninsula University of Technology, Cape Town, South Africa
^2 ^ Tygerberg Provincial Hospital, Cape Town, South Africa
**Aim:**

The study aimed to validate the relationship between inhalation injury and mortality, and to analyze differentially expressed miRNAs as inhalation injury prognostic markers.

**Methods:**

Blood samples (n = 59) were collected from burns patients shortly after admission to the Western Cape Provincial Adult Tertiary Burns Center, Tygerberg Hospital, Cape Town, South Africa for an 18-month period between 23 April 2016 and 15 August 2017. The Spearman Rank coefficient (rho) determined the correlation size and direction between varying degrees of inhalation injury and mortality risk. Total RNA extraction, quantitation, and quality analysis was performed on the blood sample. The MiRNA expression profiles of 30 exemplar samples (mild and severe inhalation injury cases) were assessed using high-throughput sequencing (Illumina NextSeq 550). A differential abundance analysis was carried out using the DESeq2 and EdgeR statistical packages. Fold-changes were reported, and *p* < 0.05 indicated significantly differentially expressed miRNA.

**Results:**

A strong, positive correlation (rho = 0.441, *p* < 0.000) was observed between inhalation injury and mortality; there were 10 differentially expressed miRNAs in mild and 23 in severe inhalation injury groups.

**Conclusions:**

Inhalation injury was shown to be a potential co-factor of mortality in burns patients, supporting previous findings. Differentially expressed miRNAs are potential biomarkers for the prognosis of mortality risk. Prognostic ability should be validated using a pathway analysis to determine the links between the differentially expressed miRNAs and the sequelae of potential events associated with inhalation injury in burns patients.

**P013**
**Covalently Immobilized Selenomethionine in Gelatin Methacryloyl Hydrogel Promotes Wound Healing in Aged Skin by Attenuating Ferroptosis via the Arachidonic Acid–GPX4 Axis**
Jiachen Sun, **Chuanan Shen**, Huageng Yuan
Chinese People’s Liberation Army General Hospital, Beijing, China
**Aim:**

Explore the mechanism of epidermal stem cells (EpiSCs) aging to elucidate targets for better wound healing.

**Methods:**

Transcriptome sequencing and untargeted metabolomics were performed, analyzed and verified on epidermises from young and aged mice. GPX4 and arachidonic acid (ARA) were screened out and applied to reveal their effect on aged epidermises. AC-PEG tethers were used to covalently immobilize Selenomethionine (Se-Met) within GelMA hydrogels. A full-thickness skin defect model was established in aged mice to explore the effect of GelMA-Se-Met on wound healing and epidermal ferroptosis.

**Results:**

Transcriptome data revealed metabolic adaptation in the aged epidermis related to ARA, cytochrome P450, glutathione, retinol, and sphingolipids, etc. GSEA analysis showed increased ARA monooxygenase and metabolism. Untargeted metabolomics revealed an upsurge in unsaturated lipids, particularly those of the ARA family. KEGG revealed an enrichment in the biosynthesis of unsaturated lipids and ferroptosis. Both mice and humans exhibit enhanced ferroptosis in aged epidermises, with decreased GPX4. ARA could induce ferroptosis in EpiSCs lacking GPX4. Additional ARA feeding could induce ferroptosis in the EpiSCs of middle-aged mice compared to young mice. Se-Met showed a protective effect against ARA-induced ferroptosis. AC-PEG-functionalized Se-Met retained the native bioactivity of Se-Met, and was stably retained within GelMA for over 7 days. Moreover, 10% GelMA-Se-Met hydrogel promoted wound healing in aged mice, with improved wound healing, increased GPX4, decreased lipid peroxidation, and ARA levels.

**Conclusions:**

Enhanced ferroptosis in aged epidermises may be due to decreased GPX4 and increased ARA, which may lead to delayed wound healing. GelMA-Se-Met hydrogel promoted wound healing by supplementing GPX4 expression and inhibiting ferroptosis.

**P014**
**The Use of Meshed Autografts with Cryopreserved Allografts from an In Vitro Cultured Human Epidermis for Treatment of Full-Thickness Burns: a Case Report**
**Israel De Jesús Silva Saucedo**, Kevin Arias Martínez, Yuri Jiménez Caprielova, Luis Roberto Azcanio Macias
Plastic Surgery Department, UMAE Victorio de la Fuente Narvaez, Mexico City, Mexico
**Aim:**

To demonstrate that in vitro cultured human epidermis allografts promote faster epithelialization of burns.

**Methods:**

In the burns unit of the traumatology hospital “Dr. Victorio de la Fuente Narváez” of the IMSS, allografts of cultured human epidermises have been used successfully in the therapy of a burned patient.

This case report describes the treatment of a female patient of seventy years old with full-thickness burns, using autografts of skin expanded to three to six times their original size, covered with allografts of a cultured epidermis.

**Results:**

In the previously mentioned case, this strategy was used because in a single surgical session it was not possible to cover all the lesions. However, therapy with meshed autografts and cultured epidermal allografts allowed all these lesions to be covered in a single surgical session, with complete re-epithelialization in only 12 days.

**Conclusions:**

It is important to emphasize that the recovery of a burned patient depends on the rapid epithelialization of the affected areas. In the case presented herein, the cryopreserved allografts of a human epidermis cultured in vitro were crucial for the early epithelialization of the donor areas and the meshing of the autografts, thereby reducing the epithelialization time of the affected areas and the length of hospital stay.

**P015**
**The Aftermath of COVID-19 Pandemic in Burn Referral Services**
**Chee Chee Tang**, Christopher Rimmer, Reena Agarwal
University Hospitals of Leicester NHS Trust, Leicester, UK
**Aim:**

Rising to the challenges and burden of the COVID-19 crisis, healthcare settings have been reshaped and adjusted to ensure safe and adequate patient care. This was shown by our experience examining the clinical and epidemiological profile of acute burn patients before, during, and after COVID-19 pandemic.

**Methods:**

This is a retrospective observational study comparing acute burn patients presented in the winter months (December, January, and February) of 2018/2019, 2019/2020 and 2022/23. These three time periods were selected to represent pre-COVID, during COVID, and current trends, respectively. Comparisons were performed using either Pearson’s χ2 for categorical variables or a one-way ANOVA for continuous variables.

**Results:**

A total of 62 patients (female = 34; male = 28), 64 patients (female = 29; male = 35), and 80 patients (female = 42; male = 38), were referred in 2018/19, 2020/21 and 2022/23, respectively. We observed an overall upward trend in burn referrals after the pandemic, especially for patients under 16. There was similar pattern in the mean percentage of total body surface area burned, with 1.1% in 18/19, 1.4% in 20/21, and 1.4% in 22/23. The mechanisms of burns changed, with the majority in 18/19 and 22/23 being scald burns (64.5% vs. 62.5%), but the percentages of contact (40.6%) and scald (43.8%) burns were similarly high (*p* = 0.02) in the 2020/21 period.

**Conclusions:**

This study gives a brief overview of burn patient profiles pre-, during, and post-COVID-19 lockdown. The surge in 22/23 provides evidence of potential increased burn service demand associated with the influence of COVID-19. Changes in lifestyle such as working from home may have led to the changes observed in burn mechanisms.

**P017**
**Systemically Increased Neutrophil Activity and Altered Coagulatory Phenotype after Burn Injury**
**Britt Van Der Leeden** ^1,2^, H. Ibrahim Korkmaz ^3,4,5,6^, Bouke Boekema ^3,6^, Paul van Zuijlen ^3,5,6^, Hans Niessen ^1,7^, Susan Gibbs^4^, Paul Krijnen ^1,7^
^1 ^ Department of Pathology, Amsterdam UMC, Amsterdam, The Netherlands
^2 ^ Amsterdam Infection and Immunity, Amsterdam, The Netherlands
^3 ^ Association of Dutch Burn Centers, Beverwijk, The Netherlands
^4 ^ Department of Molecular Cell Biology and Immunology, Amsterdam, The Netherlands
^5 ^ Red Cross Hospital, Beverwijk, The Netherlands
^6 ^ Department of Plastic, Reconstructive and Hand Surgery, Amsterdam, The Netherlands
^7 ^ Amsterdam Cardiovascular Sciences, Amsterdam, The Netherlands
**Aim:**

This study aimed to investigate the systemic neutrophil response and procoagulatory effects after burn injury.

**Methods:**

Plasma was obtained from burn patients (n = 7; mean total body surface area of 35% burned) at different time points post-burn (1–15 days) and healthy controls. Herein, the levels of neutrophil activation marker (HNE-a1ATC), DNA cleaving protein (DNAse1), nucleosome complex for extracellular DNA and MPO bound to extracellular DNA for neutrophil extracellular traps (NETs), as well as the coagulatory markers prothrombin factor 1.2 (PTF1.2), thrombin anti-thrombin complex, tissue plasminogen activator (tPA), and plasminogen activator inhibitor 1 (PAI-1) were determined with an ELISA. Additionally, correlation analyses of these plasma levels were performed.

**Results:**

Increased plasma levels of HNE-a1ATC, DNAse1, nucleosome complex NETs, tPA, and PAI-1 were found in burn patients compared to healthy controls. HNE-a1ATC, NETs, and PTF1.2 significantly correlated with timing after burn injury. Levels of nucleosomes and PTF12 significantly correlated with burn wound size. Burn wound size and depth, plasma nucleosome and PAI-1 levels significantly correlated with plasma C-reactive protein levels.

**Conclusion:**

Our study shows a systemic increase in neutrophil activity, NET formation and coagulatory markers after burn injury with no clear effect over time, which may be caused by observed local intravascular coagulation post-burn.

**P020**
**Epidemiology of the Critically Ill Burn Patient in a Reference Trauma Center in Catalonia**
Jacinto Baena ^1,2^, **Antonio Bulla** ^1^, Jordi Serracanta ^1^, Marcelino Baguena ^2^, Joan Pere Barret ^1^
^1 ^ Burn Unit Hospital Universitari Vall D’hebrón, Barcelona, Spain
^2 ^ Neurotraumatology Intensive Care Unit of Vall d’Hebron University Hospital, Barcelona, Spain
**Aim:**

To know the real incidence of critical burn patients admitted to a reference Trauma Center.

**Methods:**

We evaluated patients admitted to the burns unit of the Vall d’Hebron University Hospital from 2012 to 2022. A consecutive cohort of burned patients who required invasive mechanical ventilation (IMV) or amines, given their severity on admission, was studied. A total of 443 patients were reviewed and classified as critical burn patients. We collected demographic data, presence of inhalation injury (IH), percentage of total body surface area (%TBSA), burn injury mechanism, and mortality.

**Results:**

With a reference population of 8.6 million inhabitants, we observed 4.68 admissions for burn injuries per 100,000 inhabitants. Of these, 0.88 per 100,000 will meet the criteria for a severe burn patient, and only 0.49 per 100,000 will be considered critically burned with our criteria. Of the total of 443 patients, 70.42% were men (312), and the mean age was 48.96 years, with an SD of 18.41. The average percentage of TBSA was 29.24% with an SD of 21.07. A total of 159 patients (35.9%) concomitantly had smoke inhalation syndrome. The most frequent injury mechanism was flame (53.72%), followed by deflagration/explosion, (31.15%), scalding (6.99%), electrical injury (6.1%), chemical injury (1.58%), and freezing and dermabrasion (0.23%). Overall mortality was 19.6%, representing a total of 87 patients.

**Conclusions:**

Knowing the incidence of critically ill burn patients in our area will lead to better care management and outcomes in our reference units

**P021**
**Evaluation of the Efficacy of Nexobrid in the Treatment of Critically Ill Burn Patients: An Analysis of Trends in Use and Safety**
Jacinto Baena ^1,2^, **Angel Guillermo Arevalo** ^1,2^, Antonio Bulla ^1^, Jordi Serracanta ^1^, Joan Pere Barret ^1^
^1 ^ Burn Unit Hospital Universitari Vall d’Hebrón, Barcelona, Spain
^2 ^ Neurotraumatology Intensive Care Unit of Vall d’Hebron University Hospital, Barcelona, Spain
**Aim:**

To evaluate the use of Nexobrid and its safety in critically ill burn patients.

**Methods:**

We evaluated patients admitted to the burns unit of the Vall d’Hebron University Hospital from 2012 to 2022. A consecutive cohort of burned patients who required invasive mechanical ventilation (IMV) or amines, given their severity on admission, was studied. A total of 444 patients were reviewed and classified as critical burn patients. We collected demographic, presence of inhalation injury (IH), percentage of total body surface area (%tBsA), mortality, and use of enzimatic debridment data.

**Results:**

Of the total of 444 patients, 111 were treated with Nexobrid to debride a burned area. Nexobrid was used to debride 7 critical burn patients in 2015, 16 in 2016, 13 in 2017, 11 in 2018, 19 in 2020, 11 in 2021 and 16 in 2022. Respectively, 14.28% of critical patients received some debridement with Nexobrid in 2015, 34.04% in 2016, 27.08% in 2017, 32.35% in 2018, 51.42% in 2019, 63.33% in 2020, 55% in 2021 and 72.72% in 2022. The % of mean TBSA debrided with Nexobrid from 2015 to 2022 is 16.42%, 17.12%, 7.84%, 11.91%, 13.55%, 12.84%, 20.45% and 19.93%, respectively. Using the logistic regression model, we observed that the use of Nexobrid at the same %TBSA, age, and inhalation does not increase mortality

**Conclusions:**

The use of and percentage of debrided area with Nexobrid has progressively increased. The use of Nexobrid in the critical burn patient is safe.

**P022**
**Mortality Predictor Scores in the Critically Burned Patient: ABSI, Modified ABSI, and Revised Baux Score Nomogram. Which One Is Closest to the Real Mortality?**
Jacinto Baena ^1,2^, **Antonio Bulla** ^1^, Jordi Serracanta ^1^, Marcelino Baguena^2^, Joan Pere Barret ^1^
^1 ^ Burn Unit of the Vall d’Hebron University Hospital, Barcelona, Spain
^2 ^ Neurotraumatology Intensive Care Unit of Vall d’Hebron University Hospital, Barcelona, Spain
**Aim:**

To evaluate which of the predictive mortality scores is most representative of the real mortality in our burn unit.

**Methods:**

Critical burn patients admitted to the burn unit of the Vall d’Hebron University Hospital from 2018 to 2022 were evaluated. A consecutive cohort of burn patients who required invasive mechanical ventilation (IMV) or vasopressors, given their severity on admission (129 patients), was studied. Demographic, mortality, full-thickness burns, and inhalation injury data, and mortality predictor scores (ABSI, modified ABSI (mABSI), revised nomogram Baux, and APACHE II) were collected. We assessed the predictive accuracy of the different scores of mortality using logistic regression. We used the measure of the area under the ROC curve to assess the predictive accuracy of the model.

**Results:**

The results of the area under the curve (AUC) for predicting death in ABSI, mABSI, revised nomogram Baux, and APACHE II were, respectively, 0.7912, 0.7634, 0.8009 and 0.6639.

**Conclusions:**

APACHE II is a poor predictor of mortality in critically ill burn patients.

The results mean that we consider ABSI, mABSI, and the revised nomogram Baux score good tests for predicting death in our burn patients.

The logistic regression analysis seems to suggest that full thickness, inhalatory injury and sex parameters, thanks to modern resuscitation techniques, are less relevant than previously thought.

Baux score seems to be the most accurate model for our data.

**P023**
**Failed Endotracheal Intubation in a Severely Burned Patient with Uncomplicated Airway Management in Previous Surgeries: a Case Report**
**Ana Mesić** ^1^, Mirta Ciglar ^1^, Tatjana Beker ^1^, Aleksanda Munjiza ^2^, Desa Mirela Dobrić ^1^
^1 ^ Department of Anesthesiology, Intensive Care and Pain Medicine, Sestre Milosrdnice University Hospital Center, Zagreb, Croatia
^2 ^ Traumatology Clinic of Sestre Milosrdnice University Hospital Center, Zagreb, Croatia
**Aim:**

To report a case of failed endotracheal intubation in a patient with major burns who had earlier developed subglottic tracheal stenosis.

**Methods:**

A 51-year-old male (50% TBSA, IIb- and III-degree burns of the head, neck, and back) was scheduled for a fifth necrectomy and autotransplantation of the skin. He had mild dyspnea due to inhalation injury and pneumonia. After induction with sufentanil, propofol, and rocuronium, there were no complications with mask ventilation. Direct laryngoscopy was performed and the patient appeared to be Cormack–Lehane grade I, but the 7.5 mm and 7.0 mm internal diameter endotracheal tubes failed to pass the vocal cords. Flexible fiberoptic bronchoscopy revealed granulations that narrowed the subglottic space to approximately 3 mm. After reversing the neuromuscular blockade with sugammadex, the patient was awakened. After a neck CT scan and consultation with an ENT surgeon, the patient was scheduled for a tracheotomy.

**Results:**

The neck CT scan showed significant narrowing of the trachea to a diameter of 6 mm within a length of 32 mm. The ENT surgeon performed a tracheotomy under local anesthesia with sedation. After the burns treatment was completed, tracheal resection and reconstruction were performed, and the patient was discharged home for further rehabilitation.

**Conclusions:**

We find this case report valuable for increasing awareness of the potential for the development of early granulation, which can cause tracheal stenosis in major burn patients undergoing multiple airway manipulations. Additionally, it is every physician’s responsibility to perform tracheal intubation, following preventive guidelines, to minimize the risk of developing postintubation tracheal stenosis.

**P024**
**Evaluating the Efficacy and Safety of Landiolol Hydrochloride for Management of Arrhythmia in Septic Burn Patients.**
**Athina Lavrentieva** ^1^, Vasiliki Karali ^1^, Eirini Nikolaidou ^2^, Theodora Ligomenou ^2^, Milly Bitzani ^1^
^1 ^ Papanikolaou Hospital, Burn ICU, Thessaloniki, Greece
^2 ^ Papanikolaou Hospital, Department of Plastic Surgery and Burn Unit, Thessaloniki, Greece
**Aim:**

To investigate whether landiolol, an ultra-short-acting cardio-selective beta blocker, can safely and effectively control heart rate in critically ill septic burn patients with supraventricular tachyarrhythmias.

**Methods:**

Fourteen septic burn patients who suffered from supraventricular tachyarrhythmias (heart rate ≥ 120 bpm for >1 h) and were admitted to a burn ICU between November 2021 and January 2023 were included. Arterial pressure, heart rate, cardiac rhythm, and cardiac output were recorded at 1, 8 and 24 h after the initiation of tachyarrhythmias. The primary endpoint was heart rate response and the absence of increased vasopressor requirements within the first 24 h of the treatment’s initiation.

**Results:**

The mean age of patients was 45 ± 21 years, the mean total body surface area burned was 37 ± 23%, and the mean sequential organ failure assessment score was 8 ± 5. Paroxysmal atrial fibrillation (five patients), paro¬xysmal atrial tachycardia (two patients), and paroxysmal supraventricular tachycardia (seven patients) were observed during septic episodes. The initial landiolol dose administered was 20 ± 11 mcg/kg/min. Rapid and substantial reduction of heart rate was observed in septic patients treated with landiolol without any deterioration of hemodynamics (cardiac output, arterial pressure). No increase in the patients’ requirement of norepinephrine was observed. Landiolol significantly reduced heart rate (from 125 ± 16 bpm to 85 ± 12 bpm). Conversion to sinus rhythm was observed in three patients with paroxysmal atrial fibrillation. Conclusions: Landiolol safely reduced heart rate and, in part, converted septic burn patients with supraventricular tachyarrhythmias to sinus rhythm.

**P025**
**Blood Management in Burn Surgery: A Retrospective Analysis Study of SOC Vs. Enzymatic Debridement (ED)**
**Jasminka Minic**, Enrico Vigato, Maurizio Governa
Azienda Ospedaliera Universitaria Integrata Verona, Verona, Italy
**Aim:**

Patients with major burn injuries frequently require multiple blood transfusions that increase the delay to primary burn excision. Patient blood management (PBM) represents an international initiative in best practice, as recommended by World Health Organization (WHO) EU commission. Our objective in this study was to assess blood loss eschar removal in two different groups: NXB vs. SOC.

**Methods:**

We conducted a retrospective study comparing consecutive adult burn patients admitted to Verona Burn Center and treated before (n = 22) and after (n = 22) introducing rapid bromelain-based ED. The study sample consisted of 44 subjects, of whom 22 (50.0%) were treated with enzymatic debridement (ED) and 22 (50.0%) were treated surgically (SOC). Patient data were summarized using descriptive statistical methods.

**Results:**

The mean age was 59 years, the patients were 54% male, and the mean TBSA was 23.5%. The two groups were comparable in terms of age, sex, and TBSA. The median time to complete debridement was 7.8 days in the SOC group, and 1.2 days in ED group. The mean number of blood units required after debridement procedure was 1.3 ± 2.0 (0–8); 2.5 ± 2.3 (0–8) in the SOC group; 0.1 ± 0.5 (0–2) in the ED group, *p*-value < 0.0001.

**Conclusion:**

Blood product transfusion has historically been utilized after major burn injury. Transfusion has been implicated in infection and immunosuppression in many disease states. Blood management improves patient outcomes and reduces costs. Enzymatic debridement should be take in account as tool in the blood management.

**P026**
**Initial Fluid Resuscitation in Burn Patients Admitted to the Intensive Care Unit as a Risk Factor for Kidney Injury and Mortality**
Ana Vidal, **Elena Díaz**, Gloria Pérez, Raquel Ferrandis, Pilar Argente
Hospital La Fe, Valencia, Spain
**Aim:**

To e:xamine fluid resuscitation during the first 72 h, and its effect on kidney injury and mortality.

**Methods:**

We conducted a retrospective study in patients with a total burn surface area (TBSA) ≥ 40% admitted to a burn critical care unit between 2017 and 2022. We collected fluids administered during the first 72 h. In the first 24 h, the Parkland formula was applied, and afterwards patients were divided into groups according to the type and amount of fluids administered: a crystalloids group (<5000 mL vs. ≥5000 mL); a colloids (albumin 5%) group (<2500 mL vs. ≥2500 mL) and a total intravenous fluids group (<10,000 mL vs. ≥10,000 mL). A chi-square test was performed to determine the correlation between the groups and renal failure and death.

**Results:**

A total of 25 patients were included (20 men and 5 women, with an average age of 44 years old). Of them, eight patients (32%) developed acute kidney injury (AKI), with three patients (12%) requiring renal replacement therapy. AKI showed more incidence in the groups of crystalloids ≥5000 mL (20% vs. 10%, *p* 0.18), colloids > 2500 mL (28% vs. 4%, *p* 0.2), and total fluids ≥ 10,000 mL (25% vs. 8%, *p* 0.14).

Mortality was higher in patients with AKI (28% vs. 12%).

**Conclusions:**

AKI, which is independently associated with high mortality in burned patients, is still highly prevalent (1). Current studies (2) promote reduced and target-guided fluid therapy, not just therapy guided by urine output, to prevent the occurrence of AKI and reduce mortality.

**P027**
**Prone Positioning Improves Oxygenation in Burn Patients with Severe Acute Respiratory Distress (ARDS): A Series of Three Case Reports**
**Ana Vidal**, Raquel Ferrandis, Pedro Alvedro, Dolores Pérez del Caz, Pilar Argente
Hospital La Fe, Valencia, Spain
**Aim:**

Although prone positioning (PP) has been proven to improve oxygenation in patients with acute respiratory distress syndrome (ARDS), it is challenging in burn patients due to the difficulty of skin protection.

**Methods:**

We assessed three burn patients (TBSA 35%, 20% and 25%) who developed ARDS without response to lung protective ventilation, muscle relaxation, and nitric oxide. The mechanism of burn was deflagration, with inhalation syndrome in only one patient. Regarding personal history, the patients were smokers, and two of them obese.

**Results:**

PP was established on four occasions in all of them. The oxygenation improvement was achieved 2 h after adopting the position. The mean PaO2/FiO2 ratio increased from 75 (+/− 12) to 187 (+/− 86). Patients were volume control-ventilated (6–8 mL/kg) with PEEP 10–14 cmH2O. Days of mechanical ventilation amounted to 32, 39, and 38, respectively.

Two patients had chest burns, so it was important to protect these areas.

No patient died, and they were discharged from the hospital without oxygen therapy or respiratory sequelae.

**Conclusions:**

Prone positioning improves oxygenation in burn patients with ARDS, and may reduce the high mortality described in this scenario. Methods of protecting the skin in PP are yet controversial.

However, a more comprehensive study with a larger sample size is required to make a valid conclusion.

**P028**
**Blood Transfusion in Severe Burn Patients: A Retrospective Review in Burn Critical Care from 2017 to 2022**
Ana Vidal, **Nerea Díaz-Ros**, Raquel Ferrandis, Dolores Pérez del Caz, Pilar Argente
Hospital La Fe, Valencia, Spain

Aim: To determine the factors associated with transfusion requirements and mortality.

**Methods:**

We performed a retrospective study of patients with ≥40% total burn surface area (TBSA) admitted from January 2017 to December 2022. Demographic information and blood transfusion details from the first month were analyzed. Patients were divided into two groups according to red blood cell (RBCs) units transfused (<10 vs. ≥10 units). A chi-square test was performed to determine the correlation between transfusion, TBSA%, and death.

**Results:**

We included 25 patients, of whom 20 patients who survived more than 24 h were studied (10 patients transfused < 10 units; 10 patients transfused ≥ 10 units). In both groups, transfusions were performed from the 6th–7th day, probably in connection with debridement surgeries. Administration of tranexamic acid was performed during surgeries.

We observed that only 25% patients with TBSA 40–60% required > 10 units, in comparison with the 40% of patients with TBSA > 60% (*p* 0.1) who required > 10 units.

Mortality was 10% in patients transfused < 10 units, compared with 20% in patients transfused ≥ 10 units (*p* 0.25).

Statistical analysis did not find significant differences.

**Conclusions:**

Severe burn patients have high transfusion demands. In our study, as in the literature, increased TBSA is associated with increased transfusion and mortality. More data are needed to establish risk factors.

**P029**
**The Sofa Score as a Predictor of in-Hospital Mortality in Critically Ill Burn Patients**
**Waleed Burhamah** ^1^, Ahmad T. Al-Sultan ^2^, Shahad Alabdulmuhsen ^3^, Khaled Altarrah ^3^
^1 ^ University Hospitals Birmingham, West Midlands, UK., Birmingham, UK
^2 ^ Department of Community Medicine and Behavioural Sciences, College of Medicine, Kuwait University, Kuwait, Kuwait
^3 ^ Al Babtain Center for Burns and Plastic Surgery, Ibn Sina Hospital, Sabah Medical region, Jamal Abdul Nasser Street 700035, Kuwait, Kuwait
**Aim:**

To assess the role of the Sequential Organ Failure Assessment (SOFA) score between Day 1 and Day 4 as a predictor of in-hospital mortality in critically ill burn patients.

**Methods:**

This is a retrospective cohort study of adult victims of thermal burns who met the criteria for intensive care burn unit (ICBU) admission and who survived more than 3 days in hospital, during the period 2019–2021. Demographic data along with SOFA scores were measured for the admission day and subsequent 3 days (SOFA 1, SOFA 2, SOFA 3, SOFA 4). Multivariate logistic regression analyses including other variables associated with mortality were performed to calculate adjusted odds ratios (ORs). Additionally, the area under the receiver operating characteristics curve (AUROC) was calculated for mortality, comparing day 1 to day 4 SOFA scores.

**Results:**

A total of 136 patients were included, with a mortality of 16.9%. After adjusting for age, TBSA%, and inhalation injury, Day 1 and Day 2 SOFA scores demonstrated superiority in predicting in-hospital mortality (SOFA1 OR 1.73 (95% CI 1.18–2.54) *p*-value 0.005 and SOFA2 OR 1.50 (95% CI 1.08–2.06) *p*-value 0.015). The AUROC for the prediction of mortality with SOFA1 was 0.82 (95% CI 0.696–0.955) and with SOFA2 was 0.871 (95% CI 0.776–0.966).

**Conclusions:**

In critically ill adult burn patients, early SOFA scores (SOFA 1 and 2) were independently associated with mortality, demonstrating superiority to late changes in SOFA scores (SOFA 3 and 4). This allows for their potential use as an adjunct to the validated prognostic scores commonly utilized to predict mortality.

**P030**
**Extensive Burns in Elderly Patients and Their Adjuvant Treatment with Human Keratinocyte Allografts**
**Gerardo Elias Lujan Alvarez** ^1^
Instituto Mexicano Del Seguro Social, Mexico, Mexico

Burns in older adults pose a significant public health problem due to these patients’ susceptibility to complications, concomitant diseases, malnutrition, and neglect by family members. A burn is severe if it affects 10% or more of the body surface area and involves second-degree or deeper burns, or special areas. The burn unit at the Traumatology Hospital VFN in Mexico City has implemented an early medical and surgical management protocol and the use of cultured human keratinocyte allografts to treat these patients comprehensively. Allografts were used in areas affected by superficial second-degree burns, mixed areas, and donor areas of partial-thickness grafts. The protocol included early fluid resuscitation, antibiotic therapy, adequate nutrition, surgical cleaning, and live-cultured keratinocyte allografts. The use of allografts significantly improved the clinical evolution and prevention of complications in severely burned older adult patients. This therapy is considered an important adjuvant therapeutic armamentarium for improving both patients’ survival and their definitive prognosis.

**P031**
**Sealed Silver Dressing Practices in Paediatric Burn Injured Patients in the Northeast of Scotland**
**Kazem Shukri Al-masri**, Matthew Shott, Jennifer Greenhowe
NHS Grampian-Royal Aberdeen Children’s Hospital, Aberdeen, UK
**Aim:**

Infection prevention is essential in burn management. Burns may require dressing changes, antibiotics, and reconstruction. Regular dressing changes allow for close monitoring. Liberal antibiotic use leads to resistance. We have assessed our use of sealed silver dressings in paediatric patients over a 3-year period. The purpose is to review practices using this technique and to assess their outcomes, focusing on infection rates.

**Methods:**

E-notes were used to collate data retrospectively; said data are routinely submitted to Care of Burns in Scotland database. Data were collected for all patients referred to our paediatric department with injuries between November 2019 and December 2022.

**Results:**

We have treated 187 patients with the ActicoatTM protocol. Application of our silver dressing occurs in theatre under general anaesthetic, and the dressing lasts for 7 days before requiring a change; patients return to theatre every 7 days if further debridement or ActicoatTM dressings are anticipated. This reduces PTSD, pain, dressing changes, and the overall burden on the service and the patient. Prophylactic antibiotics are not routinely prescribed. Furthermore, these patients can usually be managed as outpatients with planned day-case unit admissions; this reduces the duration of inpatient stay. Of our 187 patients, 20 were prescribed antibiotics, 5 suffered infected burns, 1 had an infected donor site, and 2 were treated as TSS. Patients treated for TSS had delayed presentations and were not managed per our usual local protocol.

**Conclusions:**

We concluded that our protocol achieves low rates of infection without routine use of prophylactic antibiotics, and minimizes the burden on the service.

**P032**
**Introduction of a Bacterial Binding Dressing in a UK Burns Centre**
**Nicole Lee**, Paul Crane
Chelsea and Westminster, London, UK
**Aim:**

To introduce and evaluate a bacterial binding dressing within a UK burns centre to explore its use in the reduction of wound colonization and the reduced use of topical antimicrobials, with view to reducing endotoxin release from dead cells in the wound bed to see if this will lead to improved wound healing.

**Methods:**

We introduced a Sorbact dressing, as a primary layer, to wounds that fit the inclusion criteria.

Wound images were recorded in patients notes pre-, during, and post-application. Staff and patient feedback was collected as part of the trial, using a feedback form.

**Results:**

There was a clear visual reduction in wound discoloured exudate; clear molculyte images show a reduction in visual coloured infection and a reduction in areas of colonisation after using the dressing for 6 days, with 1 reapplication of the product during this time. The Sorbact dressing was compared to a comparable wound (on the same patients) with standard dressing practice, i.e., acetic acid or silver, using multiple microbiology; Sorbact showed improved wound bed preparation for further grafting in deeper colonised burn wounds, thereby leading to earlier application of skin graft.

Staff found product easy to use, but felt the sizes of dressings needed to be bigger. Patient feedback indicated no concerns.

**Conclusion:**

Within the cohort of patients treated with Sorbact dressings, the data show that they are effective burn wound decolonising dressings that are easy to use and save costs overall. Further studies with a larger number of patients are required to validate the data, and bigger dressing sizes need to be produced.

**P033**
**Cutaneous Manifestations of Candida Parapsilosis in Burns Patients: A Novel Pathognomonic Clinical Sign for the Burn Surgeon to Look Out For**
**Aasim Murtaza** ^1^, Umran Sarwar ^1^, Preetha Muthayya ^2^, Umair Anwar ^2^
^1 ^ Bradford Royal Infirmary, Bradford, UK
^2 ^ Pinderfields Hospital, Regional Burns Center, Wakefield, UK
**Aim:**

- To present two patients, one with 85% TBSA and the other with 65% TBSA burns, who both developed a superimposed Candida Parapsilosis infection within their burn wounds.

**Methods:**

- A 16-year-old patient with 85% TBSA burns and a 33-year-old patient who had 65% TBSA burns, both of whom had prolonged stays on the Intensive Care Unit and underwent multiple burn operations, who subsequently developed a Candida Parapsilosis infection.
- Both patients grew multi-drug-resistant pseudomonas that required the removal of the biodegradable temporising matrix (BTM), use of multiple antibiotics, topical acetic acid, and sodium hypochlorite.
- Clearly demarcated cutaneous lesions were identified over the torso and lower limbs, and histopathological analysis was performed.
- Both of these cases demonstrated clinical signs of Candida Parapsilosis infection and involved instigation of treatment in a timely manner whilst awaiting candida sensitivities and subsequent anti-fungal therapies.

**Results:**

- We observed discreet erythematous circular lesions with well-defined central ulceration with a halo covering the torso and lower limbs of the burn-affected areas.
- Histopathology of the cutaneous lesions identified Candida Parapsilosis.
- Both patients were Beta-d-glucan positive, with one having a level of 253 pg/mL. (Normal: <83 pg/mL).

**Conclusions:**

- Candida species are becoming more common in patients with severe burns, with Candida Parapsilosis an increasing cause of morbidity and mortality over the past two decades.
- This case report highlights the subtle and novel clinical pathognomonic lesions that can be utilised to rapidly identify Candida Parapsilosis superimposed infections in delayed-healing wounds.

**P040**
**A Simple Mnemonic, B.U.R.N.S., for Burns First Aid**
Grace Tan, **Kok Chai Tan**, Si Jack Chong, Andrea Lim, Chee Liam Foo
SGH, Singapore, Singapore
**Introduction:**

Burn injuries remain common in the world. Singapore sees an average of 220 burn admissions annually. The authors identified that there is a need for more public awareness on first aid burn treatment. The authors devised a simple mnemonic that can be used in burns education for first aid treatment, to be taught to trained first responders who will have the first contact with these burn patients.

The aim was to assess the viability of implementing this mnemonic, B.U.R.N.S, to facilitate first aid education for burns.

**Materials and Methods:**

In the pilot, we presented this mnemonic as a poster to 30 full-time burn care medical professionals. Feedback was then obtained from this group of medical professionals and used to revise the mnemonic. The mnemonic was then subsequently taught to 400 first responders. They were then asked to reiterate the mnemonic to test the ease of remembering the mnemonic. Objective feedback was obtained using a 5-point scoring system.

**Results:**

The results indicated a significant improvement in burn first aid knowledge after the implementation of the mnemonic, with a score of 3.67–4.77. The content was deemed appropriate and easy, and participants were able to recall the content.

**Conclusions:**

The study results suggest that the B.U.R.N.S. mnemonic and visual aid is simple and easy to apply, especially for uniformed personnel. Overall, burns first aid awareness and education can be improved with the implementation of this mnemonic and poster.

**P041**
**An Open-Cohort Observational Study of the Efficacy and Cost-Effectiveness of Integrated Aftercare Models for Burn Patients: A Study Protocol**
**Peter Moortgat** ^1^, Evert De Rijcker ^1^, Jill Meirte ^1,2^, Mieke Anthonissen ^1,2,3^, Lisa Senepart ^1^, Koen Maertens ^1,4^, Thibau Demarbaix ^1^, Ulrike Van Daele ^1,2^, Lieve De Cuyper ^1,5^
^1 ^ Oscare, Organisation for Burns After-care and Research, Antwerp, Belgium
^2 ^ University of Antwerp, Rehabilitation Sciences and Physiotherapy, Antwerp, Belgium
^3 ^ University of Leuven, Faculty of Kinesiology and Rehabilitation Sciences, Leuven, Belgium
^4 ^ Vrije Universiteit Brussel, Department of Clinical and Lifespan Psychology, Brussels, Belgium
^5 ^ ZNA Antwerp, Burn Center, Antwerp, Belgium
**Aim:**

To develop a study-protocol to investigate whether different aftercare models generate sufficient effectiveness to improve burn patients’ quality of life (QoL), more specifically an increase in quality-adjusted-life-years (QALY) one year after discharge from the hospital.

**Method:**

This study is divided into two parts. Study part A is an open-cohort prospective observational study of different aftercare models in terms of the QoL of burn patients. These aftercare models consist of scenarios of multidisciplinary care pathways, which are part of the patients’ usual care. Study part B is a quantitative comparative health economics study.

The population consists of burn patients ≥ 8 years old that meet the national legal criteria for referral to a burn center.

The outcome measures for the prospective observational study are the QoL questionnaires EQ-5D-5L and SF-36, which form the basis for the health economic analyses. Burn-specific QoL is measured with BSHS-B, and scar quality is assessed with POSAS. The patient is expected to keep a diary, wherein all visits to caregivers and all costs made out-of-pocket are registered. Semi-structured interviews with patients and caregivers will identify the facilitators and barriers in the implementation of care. Finally, focus groups will formulate recommendations to optimize the organization of the aftercare of burn patients.

**Results:**

The study was found ethically acceptable by IRB ZNA-Antwerp, Belgium EC 5780–009OG031.

**Conclusions:**

Evaluating the efficacy and cost-effectiveness of different models of burns aftercare is essential in striving towards a full life beyond mere survival for burn patients. This study protocol could serve as a template for comparative burns aftercare research.

**P045**
**Using AnaConDa to Treat Opioid Tolerance in a Severe Burn Patient**
**Maryann Grønstøl**, Knut Eiane
Department of Plastic and Reconstructive Surgery and National Burn Center, Haukeland University Hospital, Bergen, Norway
**Aim:**

To share our experience using AnaConDa to treat opioid tolerance and attempting to improve the pain management of a complex burn patient suffering 85% TBSA.

**Methods:**

Information and data were extracted from the patient’s medical journal, and nurses’ and doctors’ reports. We compiled and gathered experiences through oral discussions and dialogue with doctors and nurses that had treated the patient.

**Results:**

After long-term treatment with high doses of opioids, the patient’s experience of pain became difficult to treat. Critical treatments such as mobilization and daily procedures appeared unmanageable. Opioid rotations, different medical combinations, and other interventions became insufficient over time. For the first time in our burn unit, we decided to use isoflurane through the anesthetic conserving device “AnaConDa”, to resensitize the patient to opioids. The treatment lasted for 7 days. After waking up, the patient suffered from delirium for 7 days, their normal routines were disrupted, and the amount of opioids given escalated due to sustained pain complexity. At 15 days post-treatment, opioid levels were almost back to initial levels.

**Conclusions:**

We found both beneficial and non-beneficial effects from the treatment. The resensitization was effective but temporary. The following delirium made general treatment difficult, and caused psychological downsides for the patient. The AnaConDa treatment did not solve the tolerance permanently, but might have served as a reset, and thereby enabled new strategies regarding pain management.

**P046**
**Eye-Tracked Computer Games as a Method for Pain Perception Alleviation in Chronic Wound Management**
**Wojciech Łabuś**, Przemysław Strzelec, Karolina Ziółkowska, Anna Słaboń, Artur Wielgórecki
Stanisław Sakiel Center for Burn Treatment in Siemianowice Śląskie, Siemianowice Śląskie, Poland
**Aim:**

Chronic pain frequently accompanies the daily lives of many chronic wound patients. The degree of pain experienced significantly increases when performing medical procedures related to wound management. The use of eye-tracked games in order to distract the patient from the painful activities being performed may be an effective procedure.

**Methods:**

Some 40 patients suffering from chronic wounds qualified for the study. Patients engaged in eye-tracking games during dressing changes and wound cleaning. Pain sensations were surveyed. The survey concerned the pain experienced on a daily basis when changing dressings with and without the use of eye-trackers.

**Results:**

On the basis of the obtained results, it was found that eye-trackers significantly reduced the pain experienced during dressing changes compared to the pain caused by these procedures without the use of eye-trackers.

**Conclusions:**

On the basis of the obtained results, it was proposed that eye-trackers be introduced into routine clinical practice during chronic wound management.

**P047**
**Improving the Pain Management of Burns Patients**
**Elena Whiteman**, Daniel Markeson, Francisco Regel Vilas Boas Da Silva, Shyama Chada, Yaldasadat Hashemi
Chelsea and Westminster Hospital, London, UK
**Aim:**

To examine whether patients received analgaesia in line with existing London and Southeast Burn Network guidelines, and determine if specific demographic factors influence pain.

**Methods:**

We examined the records of patients admitted to the Chelsea and Westminster Burns Unit between November 2021 and June 2022, recording patient demographics, pain scores (0–10) before and during dressing change upon admission and during the first change of dressing (COD), and analgaesia given.

**Results:**

Data were collected for 246 patients (167 adult [mean age 52.7; 89 male, 78 female] and 79 paediatric [mean age 4.5]). A total of 426 dressing changes were further analysed (492 total, 66 excluded). Analgaesia requirements were managed appropriately on 157 occasions (52%) in adult patients, and on 53 occasions (43%) in paediatric patients. Overall, pain scores were higher in female patients (mean 1.27 prior, 4.67 during COD) than in male patients (mean 1 prior, 4.17 during COD). Patients aged ≥70 had the lowest mean pain scores (0.99 prior, 3.9 during COD), and patients aged ≤50 years had the highest pain scores (mean 1.27 prior, 4.75 during COD). Black patients had higher pain scores (1.22; 4.59) than Asian (1; 4.46) or white (0.9; 4.06) patients.

**Conclusions:**

Less than 50% of patients in our study received appropriate analgaesia. Staff education on appropriate pain management and a particular emphasis on the variable experiences of pain noted in different patient demographics is needed. We recommend further research into the factors influencing pain thresholds to help guide appropriate analgaesia management, and recommend consideration of ancillary techniques such as virtual reality to improve patient experience.

**P050**
**Outpatient Treatment in Pediatric Patients with Second-Degree Burns with the Cryopreserved Cultured Cutaneous Allograft, Epifast^®^, in the Emergency Department**
**Josue Eder Albavera**, Gladys Elizabeth Medina Guerrero, German De La Torre Leon
Emergency Service, Traumatology Hospital “Dr. Victorio de la Fuente Narváez” IMSS, Ciudad de Mexico, Mexico
**Aim:**

To ascertain the reduction in morbidity and high efficacy of second-degree burns treatment achieved with the cryopreserved cultured cutaneous allograft (CCCA), Epifast^®^, in pediatric patients with outpatient management in emergency department.

**Methods:**

A retrospective and observational study was performed including patients under 17 years of age with second-degree burns treated with CCCA in the emergency department of the traumatology hospital “Dr. Victorio de la Fuente Narváez” IMSS in México City. We reviewed a total of 34 pediatric patients and their records from 1 January 2021 to 31 December 2022.

**Results:**

Of the 34 pediatric patients’ records reviewed, 2 were discarded due to lack of follow-up. A total 32 pediatric patients’ follow-up treatments were analyzed. The mean number of follow-up treatment days was 20.7 (5 to 120 d), and the mean patient age was 5.4 years (8 m to 17 y). The causes of burns were mainly hot liquid scalding, at 78.12% (n = 25); hot liquid immersion, at 9.3% (n = 3); fire, at 6.2% (n = 2); and solar radiation and direct fire, at 3.1% each (n = 1). The mean surface burn percentage was 4.8% (1 to 10%). Some 29 patients had a correct evolution; at 10 days of follow-up, 27 had a total epithelization, 5 had a partial epithelization, and only 3 patients required application of a skin graft. No patient presented with morbidity.

**Conclusions:**

The treatment of second-degree burns with the cryopreserved cultured cutaneous allograft, Epifast^®^, is highly effective as it speeds up epithelization time, avoids morbidity, and reduces the need for skin grafts.

**P051**
**Successful Treatment of Full Body Epidermal Detachment in Toxic Epidermic Necrolysis in a Child, Using Suprathel^®^**
**Sara Fernandes**, Joana Barbosa-Sequeira, Carolina Aquino, Miguel Campos, Maria Garcia
Department of Pediatric Surgery, Centro Hospitalar Universitário de São João, Porto, Portugal
**Aim:**

Toxic epidermal necrolysis (TEN) is a life-threatening disease characterized by the detachment of the epidermis and mucous membranes. Herein, we describe the management of TEN with a detachment of >90% of the total body surface area (TBSA).

**Methods:**

This is a case of an 11-year-old girl with a history of Crohn’s disease and autoimmune hepatitis, who had recently been treated with beta-lactam antibiotics for a case of streptococcal tonsillitis. A few days later, she presented with an erythematous rash on the trunk and extremities. These lesions progressed to detachment and epidermal loss of >90% TBSA, sparing a small area on the foot. Suprathel^®^ was applied to all areas of epithelial loss except the perianal area and genitalia.

**Results:**

The patient remained under sedation for 8 days due to pain. Full epithelization was observed 7 days after Suprathel^®^ application, and the neo-epidermis was progressively exposed days later to prevent new lesions. The patient was discharged home after 17 days, after stabilization of all TEN manifestations.

**Conclusions:**

Several materials have been described as effective in covering exfoliated areas, but skin substitutes are favored. Suprathel^®^ is a synthetic skin substitute that in this case allowed for fast healing and recovery in a severe case of TEN. To the best of our knowledge, this case is part of a small number of cases involving almost all of the body surface area, and one of few successfully treated with Suprathel^®^.

**P052**
**Occlusive Dressings for the Treatment of Wounds and Burns in Newborns**
**Olga Kovalenko**, Alexei Sliepov, Olga Zhaglyuk, Lyudmila Budzinska, Anton Kovalenko
Bohomolets National Medical University, Kyiv, Ukraine
**Aim:**

To optimize the treatment of wounds in children in the first month of life. Method: In 2022, burn center surgeons observed five newborns in different hospitals. Four children were born full-term, and one was premature. Three had burns with hot water, one had a chemical burn, and one newborn had congenital aplasia of the skin. Treatment aimed to prevent infectious complications and sepsis through antibiotic therapy and occlusive dressings.

**Results:**

The burns were full-thickness. Skin aplasia is a full-layer skin defect on the lateral surfaces of the body. The duration of treatment was from 28 to 41 days; by the 23rd day of treatment, the area of the wounds had decreased by 70%, and by 30 days, by 90%. The advantages of occlusive coatings include good protection from the external environment, wound visualization, sealing, and the creation of optimal conditions for the course of the wound healing process. The main element of immune protection in the neonatal period is neutrophilic. In the imprints of wounds, a large amount of NG was found, but with incomplete phagocytosis, the digestive activity of NG was reduced. By the end of treatment, all patients had an inflammatory-regenerative type of cytogram, and a decrease in the frequency of microflora isolation.

**Conclusions:**

The use of occlusive dressings accelerates epithelialization, and reliably protects against infection. In the first and second phases of the wound healing process, with the presence of significant exudation, it is necessary to apply hydrogel dressings in the third phase to form a film cover.

**P053**
**Misleading Burn Injuries after Lightning Strike: The Value of Repeated Clinical Assessment for Adequate Local Treatment**
**Raluca Tatar** ^1,2^, Dan Mircea Enescu ^1,2^, Dan Ioniță ^2^, Andreea Dumitrescu^2^, Iulia Nacea ^1,2^
^1 ^ Carol Davila University of Medicine and Pharmacy, BUCURESTI, România
^2 ^ Grigore Alexandrescu Clinical Emergency Hospital for Children, Bucharest, Romania
**Aims:**

Lightning strike injuries represent rare life-threatening clinical situations that sometimes include burn wounds, which may initially appear as more superficial than they actually are.

**Methods:**

We report the management of a 17-year-old girl who was hit by lightning and thrown away from a rock while she was mountain trekking with her family. She suffered subsequent cardiac arrest and was resuscitated by her father. After initial assessment and first aid at a local hospital, she was transferred to the Plastic Reconstructive Surgery and Burns Department of the “Grigore Alexandrescu” Emergency Hospital for Children, Bucharest, intubated, and machine-ventilated.

**Results:**

Upon arrival, her assessment revealed 15% TBSA partial and full-thickness burns localized on the face, neck, anterior trunk, bilateral groin areas, left knee, right calf, and right anterior foot. She also presented Lichtenberg figures on the right thigh, bilateral pneumothorax, bilateral tympanic membrane perforation, neurological impairment, and minor eye injuries. She was extubated 48 h after admission. The case was managed by a multidisciplinary team. Regarding the burns’ local care, third-degree lesions (1.5% TBSA) were excised and closed with local flaps and STSG. Most of the other burn areas healed conservatively. A second surgery was needed for a 0.5% TBSA area on the right ankle, 3 weeks after admission.

**Conclusions:**

Lightning strikes usually cause superficial burns and punctate areas of third-degree wounds that heal spontaneously. Nonetheless, regular burn depth reassessment is needed, since some cases may develop a slow healing pattern, requiring surgical excision and grafting in areas seeming more superficial at the beginning.

**P055**
**Characteristics of Pediatric Burns after an Earthquake in Turkey**
**Mehmet Demircan**
İnönü University School of Medicine, Malatya, Turkey
**Aim:**

In this study, demographic and clinical comparisons were made between the patients who applied to our pediatric burn unit (group A) within 3 months of the first day of the earthquake in Turkey, and the patients who applied in the same period last year (group B). We revealed the changing patient characteristics and discussed the preventive measures to be taken in terms of child burns after a similar earthquake disaster.

**Methods:**

There were 64 patients in group A and 33 patients in group B (*p* < 0.01). There was no difference between the two groups of male–female ratios (1.2 vs. 1.3; *p* > 0.05). Group A patients were found to be younger (17 vs. 34 months; *p* < 0.01). The numbers scalding and contact burns were found to be higher in group A patients (45 vs. 15; *p* < 0.01, 16 vs. 6; *p* < 0.01). The TBSA in group A was significantly lower (17 vs. 4; *p* < 0.01)

**Results:**

In the post-earthquake period, younger children were burned mostly through scalding and contact. However, it is noteworthy that the number of applications in Group A is close to two times that of Group B.

**Conclusions:**

These results tell us that families who started to live in tents and containers after the earthquake should be informed about scalding and contact burns. It is possible to do this by hanging posters and distributing them in the form of hand brochures, and through videos in the form of public announcements on television or social media.

**P057**
**The Prognostic Effects of RDW on Survival Analysis of Severe Burn Patients**
**Zahra Haghani-Dogahe**, Mohammadreza Mobayen
Guilan University of Medial Sciences, Rasht, Iran
**Aim:**

Severe burns (TBSA > 20%) are associated with many deaths and complications that can have many economic, social, and human burdens on burn victims. There are several factors that can predict the severity of a burn injury. Here, we investigated the predictive effect of RDW on a survival analysis of severe burn patients.

**Methods:**

This study was conducted over one year. We assessed age, sex, TBSA, burn depth, inhalation injury, days dependent on a ventilator, length of hospital stay, CBC and RDW test values on days 1, 3, and 7, and final outcome.

**Results:**

A total of 104 patients were analyzed; 82 (78.8%) were male and 22 (21.2%) were female. The mean age was 41.4 years. In survivors, the mean RDW variable on the first, third and seventh days of measurement was lower compared to the deceased. At a significance level of 5%, a significant difference was observed between the means of this variable in the two groups (*p* = 0.000). The mean of the PLT variable on the first and third days of measurement was higher for surviving patients than for deceased patients. The difference between the mean platelets was not statistically significant (*p* > 0.05). The mean PLT variable on the seventh day of measurement was higher for surviving patients than for deceased patients.

**Conclusions:**

This study showed that the RDW and PLT count of the admitted burn patients are important predictors of the severity and mortality of the patients.

**P058**
**Burns First Aid: A Review of Common Practices in the General Population**
Argyro Pipinia, Eirini Nikolaidou, Eleni Karagergou, Andrew Phillip Mathew Joycey, **Sofia Papadopoulou**
“G. Papanikolaou” Hospital, Thessaloniki, Greece
**Aim:**

The purpose of this study is the identification and documentation of the first aid applied by patients immediately after burns, as well as the initial treatment used by other health professionals prior to patient referral to our department.

**Methods:**

We included burns patients seen at the emergency department and outpatients’ clinic from October 2020 until December 2022. Intubated patients were excluded. After completion of a questionnaire, we examined the type and length of the first aid applied, and the instructions offered by other health professionals.

**Results:**

In total, we included 100 patients, the majority of whom suffered a thermal burn (91%). The first aid measures applied were running water (55%), immersion in water or wet compresses (5%), and the application of various substances (27%); 23% of patients had not applied anything. The majority of patients (67%) were referred by another health facility, of which only 20% had their burn treated with saline washout. For burns with TBSA > 20%, running water was used by a significantly smaller percentage of patients (*p* = 0.025).

**Conclusions:**

A significant percentage of the general population ignores that burn washout with tepid running water is the appropriate first aid treatment for a burn. Additionally, an extensive burn area (TBSA > 20%) is a risk factor for omitting this first aid method. Burn washout with running water is easy, accessible, and significantly reduces both burn depth and morbidity. This practice, however, is not widely known, and the general population needs to be informed and made aware of it and its value.

**P059**
**Development of Delirium: Association with Old Age and Severe Burns Requiring Intensive Burn Care (Although Not Exclusively in Intensive Care)**
**Islam Abdelrahman**, Folke Sjöberg, Ingrid Steinvall, Stefan Irschik, Moustafa Elmasry
Linköping University Hospital, Linköping, Sweden
**Title:**

Delirium is defined as a disturbance of attention and awareness that develops over a short period of time, is a change from the baseline, and typically fluctuates over time. Burn care involves a high prevalence of known risk factors for delirium, such as sedation, inflammation, and prolonged stays in hospital.

Our aim was to explore the extent of delirium and the impact of factors associated with it for adult patients admitted for burn care in hospital.

**Methods:**

This was a retrospective study involving all adult patients admitted with burns over a four-year period. Daily records of the assessment of delirium using the Nursing Delirium Screening Scale (Nu-DESC) were analysed together with age, sex, the (TBSA%), operations and numbers of wound care procedures under anaesthesia, concentrations of plasma C-reactive protein, and other clinical variables.

**Results:**

Some 51 patients (19%) of the total 262 showed signs of delirium, based on Nu-DESC recordings, at least once during their stay in hospital. Signs of delirium were recorded in 42/89 patients (47%) who required intensive care, and in 9/173 (5%) who had standard acute burn care. Independent factors for delirium in the multivariate regression were age over 74 years, number of operations and wound care procedures under anaesthesia, and the need for intensive care. The average duration of hospital stay was 13.2 days longer in the group who had delirium.

**Conclusions:**

We found a strong association between delirium and older age, the need for intensive care, and number of interventions under anaesthesia.

**P061**
**A Guide to Addressing Gender Diversity in Burn Units**
**Sara Guila Fidel Kinori**, Sara García Gonzalez, María Sonsoles Cepeda Diez, Jordi Serracanta Domenech
Hospital Universitari Vall d’Hebron, Barcelona, Spain
**Aim:**

Based on a first study of sexual/gender diversity (2022) guided by the criteria and opinions of the burns care team and adding published guidelines and scientific reviews, we aimed to design a guideline of good practices for the admission and healthcare of people with sexual/gender diversity in burn units.

**Methods:**

- A process for the creation of the guide was designed in phases, initially considering a digital survey to be distributed among all the personnel of the burn unit. The responses of the team, 48% of the total staff, confirmed the main items for inclusive clinical practices, based on International LGTBQ+ guidelines.
- A preliminary draft of the guide was written and submitted for peer-review, along with a focus group discussion of the material. It was also sent to a government office for LGTBQ+ people.

**Results:**

With the results obtained, the final version was written. The text includes the factors of good practice and a glossary that facilitates understanding of the specific vocabulary, alongside an updated bibliography and scientific evidence on these inclusive practices and how they favor the LGTBQ+ population

**Conclusions:**

This guide is the first, according to the consulted bibliography, that considers specific care for LGTBQ+ people in the burn unit. One aspect that must be included is a review carried out by the people of this group, for its definitive validation. This aspect is still pending.

**P063**
**Digitalizing Burn Injury Rehabilitation: Exploring the Potential of Online Support Interventions for Recovery and Psychological Well-Being**
**Christia Huntington**, Pippa Tollow, Catrin Griffiths, Abi McNiven, Diana Harcourt
The University of The West of England, Bristol, UK
**Aim:**

To explore the lived experience of people affected by burn injuries and use the data to create a burn-specific web resource on Healthtalk.org.

**Methods:**

We took a qualitative approach using semi-structured interviews with questions about people’s experiences of having a burn or parenting a child with a burn. We carried out analysis of the data using Braun and Clarke’s six steps of thematic analysis.

**Results:**

Interviews were conducted either online or over the telephone. A total of 36 interviews were completed (11 people burnt as an adult, 13 people burnt as a child, and 12 parents of children with burns). The data were coded using Braun and Clarke’s six steps of thematic analysis. The OSOP (one sheet of paper) method of analysis was then conducted, and broad themes were identified. In all, 23 topic summaries were created under 5 broader headings: burn circumstances, treatment and services, living with a burn, adjusting to life with a burn, and information and support. Write-ups of each topic summary aimed to reflect the shared experiences of those interviewed; these were accompanied by the corresponding interview clips, which will be displayed on the Healthtalk website.

**Conclusions:**

The interview data demonstrated that although the experience of having a burn is unique to the individual, and there are nuances of treatment and impact, there is still an underlying shared experience among those with a burn. The creation of a burn injuries Healthtalk module aims to reflect these shared experiences to provide people with a sense of peer support in the form of an online intervention.

**P070**
**Innovative Solutions for the Rehabilitation of Face Burn Victims in Humanitarian Contexts: A Descriptive Cohort Analysis in Two Different Settings in Gaza and Jordan**
**Mohammed Al Qatrawi** ^1^, Zuheir Hijazi ^2^, Elise Tauveron ^3^, Abed El Hamed Qaradaya ^1^
^1 ^ Medicen Sans Frontier, Gaza City, Palestine, State of
^2 ^ Médecins Sans Frontières (MSF), Amman, Jordan,
^3 ^ Médecins Sans Frontières (MSF) Foundation, Paris, France, Paris, France
**Aim:**

Two humanitarian patient cohorts were described; these cohorts involved facial burn victims treated with transparent facial orthosis, using 3D technology, among multidisciplinary care.

**Methods:**

Access to specialized burn rehabilitation is rare in humanitarian contexts, leaving victims with major sequalae. Médecins Sans Frontières is providing burn care in Gaza and in Jordan. Three-dimensional technologies and telemedicine have been used to facilitate provision of customized and adapted compressive masks as part of these projects, one treating post-acute-phase patients (Gaza) and one post-reconstructive surgery patients (Jordan).

Clinical outcomes were analyzed for patients who completed a 6-month or 1-year treatment. Scores from the Vancouver Scar Scale (VSS) and Patient Scar Assessment Scale (POSAS) were collected at various stages of the treatment, assessing patient satisfaction regarding the orthotic (OPUS) and the daily wearing time of the device.

**Results:**

The cohorts included 73 patients in Gaza and 30 in Jordan, mainly children (80.8%/66.7%) and domestic burns victims (76.7%/73.3%). Orthoses were provided after an average delay of 23.6 days following initial assessment, and were worn daily for an average of 13 h 21 min. Clinical improvement was observed in both groups; the reconstructive-phase cohort showed a higher decrease in VSS (39.35% vs. 29.41%), whereas the post-acute-phase cohort presented a higher decrease in POSAS (40.28% vs. 25.51%). Patients reported a satisfaction rate of 97.4% in Gaza and of 94.4% in Jordan.

**Conclusions:**

Positive outcomes were observed in both cohorts in different proportions with regard to the scar evolution inherent in the stages of care. This shows the beneficial impact that advanced and specialized treatment have, even on patients living in various challenging settings.

**P071**
**Brain Lightening in Large Burns: No Time to Lose Time**
**Bohumil Bakalar** ^1^, Robert Zajíček ^1^, Ivana Štětkářová ^2^, Jiří Horáček ^3^
^1 ^ Prague Burn Center, Charles University 3rd Medical Phaculty, Prague, Czech Republic
^2 ^ Department of Neurology, Charles University 3rd Medical Phaculty, Prague, Czech Republic
^3 ^ National Institute of Mental Health, Charles University 3rd Medical Phaculty, Klecany, Czech Republic
**Aim:**

Critical illness-related polymyo- and polyneuropathy (CIM and CIN, also referred to as as ICU-acquired weakness, ICU-AW) is a common complication accompanying large burns. It severely impairs quality of life for many years after discharge. Existing preventive measures have not been significantly effective when carried out on unconsciousness patients (i.e., passive or electrically stimulated rehabilitation), emphasising the necessity of active brain involvement.

**Methods:**

To introduce a novel physiotherapy plan for the large burns, allowing brain activation and consisting of combination of:

- Early verticalization;
- Illusory movements;
- Repetitive transcranial magnetic stimulation;
- Sedation without benzodiazepines and/or propofol;
- Virtual reality;
- Eye-trackers and robot-assisted therapy and their implementation and timing.

**Results:**

The first experience of a brain-lightening programme in patients with deep burns > 30% TBSA is discussed.

**Conclusions:**

Modern methods of the brain activation are to be used in preventing CIM and CIN in patients with large burns.

**P072**
**Is the Brief Fatigue Inventory a Reliable and Valid Assessment Tool for Swedish Burn Patients? Preliminary Results**
**Sara Enblom** ^1,2^, Fredrik Huss ^1,2^
^1 ^ Burn Center, Department of Plastic and Maxillofacial Surgery, Uppsala University Hospital, Uppsala, Sweden
^2 ^ Department of Surgical Sciences, Plastic Surgery, Uppsala University, Uppsala, Sweden
**Aim:**

To validate the Brief Fatigue Inventory (BFI) for burn injuries in a Swedish population.

**Methods:**

All patients booked for follow-up at the burn center’s outpatient clinic in Uppsala 6 months after burn injury were asked to participate in the study. The inclusion criteria were (a) ≥ 18 years; (b) treated internally at the burn unit for at least 24 h; and (c) managed in Swedish both verbally and in writing. The exclusion criteria were (a) any underlying disease that obstructed the completion of the questionnaires; and (b) the patient choosing not to attend the 12-month follow-up.

Two self-assessment scales were administered to the included patients: the Fatigue Severity Scale (FSS), which has been translated to Swedish but not yet validated for burns; and the Brief Fatigue Inventory (BFI), which is valid and reliable for burns but has not yet been validated in a Swedish population. The assessments were filled out at two time-points: 6 and 12 months after burn injury. The study started in May 2020, and by the end of 2023, we plan to have 70 patients enrolled.

All the included individuals have given their informed consent, and the study was approved by the Swedish Ethical Review Authority (Dnr 2020-00387).

**Results:**

We expect the results to show that BFI is a reliable and valid assessment scale, also in a Swedish cohort. The study is ongoing, and the preliminary data of the patients that have conducted both follow-ups so far will be presented.

**P073**
**The Reliability and Validity of a Marker-Based System for Measuring Joint Range of Motion in Burn Scar Contracture Patients**
**Giyeun Hur** ^1^, Seungyeol Lee ^2^, Soyoung Joo ^3^
^1 ^ Department of plastic surgery, Hangang Sacred Heart Hospital, College of Medicine, Hallym University, Seoul, Republic of Korea
^2 ^ Department of Physical Medicine and Rehabilitation, Soonchunhyang University Bucheon Hospital, College of Medicine, Soonchunhyang University, Bucheon, Republic of Korea
^3 ^ Department of Rehabilitation Medicine, Hangang Sacred Heart Hospital, College of Medicine, Hallym University, Seoul, Republic of Korea
**Aim:**

This study aims to evaluate the reliability and validity of a marker-based system for measuring joint range of motion in burn patients with hypertrophic scar-related joint contractures.

**Methods:**

A study was conducted to measure the range of motion (ROM) in the upper extremities in 48 participants. These participants had joint contractures due to hypertrophic scars after thermal injuries. The evaluation procedure began with a warm-up period and three familiarization trials for each measurement. We measured the active range of motion for each joint using a goniometer and a marker-based system with eight infrared cameras. Participants wore reflective markers and were evaluated twice, one week apart. The motion capture system formed a skeleton model through a dynamic view, allowing for sequential measurement of the ROM of each joint. The study measured the ROM on the dominant side of each participant.

**Results:**

No statistical differences were found between the ROM measurements obtained using the two methods, for all joints measured. The intra-rater reliability of the marker-based system was acceptable for clinical use in all ROMs of shoulder, elbow, and wrist.

**Conclusions:**

The results suggest that the marker-based system can be used as an alternative to the goniometer for measuring ROM in the clinical setting, with acceptable intra-rater reliability.

**P074**
**The Clinical Utility of Exoskeleton Robot Training in Patients with Septic Arthritis after Thermal Injury: A Case Report**
**So Young Joo** ^1^, Youngsuh Yoon ^2^, Seung Yeol Lee ^3^
^1 ^ Hangang Sacred Heart Hospital, Seoul, Republic of Korea
^2 ^ Bishop Gorman high school, Las Vegas, USA
^3 ^ Soonchunhyang University Hospital, Bucheon, Republic of Korea
**Aim:**

Infections of bones and/or joints are uncommon, but delayed diagnosis or improper treatment can result in irreversible joint destruction. Early diagnosis and effective treatment are necessary for avoiding severe outcomes. There is no institution that provides clear protocols for septic arthritis (SA) rehabilitation, and there are few studies on physiotherapy. Although rare in patients with major burns, there are cases wherein SA is diagnosed. In this study, the researchers tried to confirm the clinical effect of robot training in patients with SA caused by burns.

**Methods:**

Two participants were diagnosed with SA after electrical burns. They were unable to walk due to joint pain, limitations of their range of motion (ROM), and muscle weakness before training. Throughout the training program, Rebless^®^ (H-ROBOTICS, KOREA) settings were individually set according the functional level of each patient. The ROM of the joint to be exercised (knee joint or ankle joint) was evaluated, and then the ROM of motion was set. Depending on the degree of muscle strength, it was set to active mode or passive mode before performing strength training and ROM exercise. The patients underwent 30 min of robot training with 30 min conventional therapy, 5 days a week for 8 weeks.

**Results:**

After training, gait function and pain scores were improved without adverse effect on joint ROM and muscle strength.

**Conclusions:**

In this study, it was found that robot training for patients with SA results in an improvement in gait function without deteriorating joint ROM and muscle strength.

**P075**
**Physical Fitness after Burn Injury Remains Decreased Even in the Long Term: the Results of Follow-Up after Burn Rehabilitation**
**Hubert Neubauer**, Annette Stolle, Hans Ziegenthaler, Ulrich Kneser, Leila Harhaus
BG Klinik Ludwigshafen, Ludwigshafen, Germany
**Aim:**

Severe burn injuries can result in relevant restrictions of physical capacity. Research is increasingly focusing on the general long-term consequences faced by burn victims.

**Methods:**

In a prospective trial to investigate the effectiveness of a burn rehabilitation concept, we also examined general physical capacity. Burn victims were comprehensively assessed before and after rehabilitation, and after 3 and 12 months. A bicycle ergometer test was used to measure general physical capacity (Physical Working Capacity, PWC150).

**Results:**

A total of 103 patients with a mean age of 44 years and a mean TBSA of 14.55% were enrolled. The use of beta-blockers was defined as an exclusion criterion; therefore, the test was not performed in 18.4%, 17.5%, 10.4% and 9.8% of cases at the different time points. Only a proportion of the participants reached their individual target workload. In about a quarter of the subjects, the test was discontinued due to exhaustion. The majority of participants showed a reduction in general physical capacity (exhaustion or below-average results) in 70.4%, 75.9%, 63.9%, and 70.4% of cases. A slight correlation was found with TBSA and length of stay.

**Conclusions:**

The PWC150 shows a persistent limitation of general physical performance in the majority of rehabilitants. This indicates that reduced fitness may still persist for a long time. The exclusion criterion of taking beta-blockers excludes the most severely affected patients. This means that the impairment would probably be even greater when taking into account the whole sample. Further studies analyzing the influencing factors and the effectiveness of treatment should follow.

**P076**
**Early Exercise Training following Severe Burn Injury: A Randomised Controlled Trial in China’s Largest Burn Center**
**David R. Schieffelers** ^1^, Eric van Breda ^1^, Weiguo Xie ^3^, Wu Jun ^4^, Ulrike Van Daele ^1,2^
^1 ^ Department of Rehabilitation Sciences and Physiotherapy, Faculty of Medicine and Health Sciences, University of Antwerp, Antwerp, Belgium
^2 ^ OSCARE, Organization for Burns, Scar After-Care and Research, Antwerp, Belgium
^3 ^ Department of Burns, Tongren Hospital of Wuhan University & Wuhan Third Hospital, Wuhan, China
^4 ^ Department of Burn and Plastic Surgery, The First Affiliated Hospital of Shenzhen University, Shenzhen, China
**Aim:**

To investigate the potential effect of exercise training administered during the acute phase of severe burns on muscle wasting and quality of life.

**Methodology:**

Adults ≤ 65 years with burns ≥ 40%TBSA, recruited at the Department of Burns in Wuhan, China, were randomly allocated to receive either standard care (n = 29), or additionally exercise training (n = 29) consisting of resistance and aerobic training 3–5 times per week. Exercise was commenced 8 days [IQR 5–9] after admission and lasted between 6–12 weeks depending on hospital discharge. Ultrasound-derived quadriceps muscle thickness (QMLT), rectus femoris cross-sectional area (RF-CSA), lower limb muscle strength, BSHS-B, and EQ-5D-5L were assessed at baseline and after 6 and 12 weeks. Mixed regression models were used to analyse inter-group changes over time.

**Results:**

Data from 58 subjects (42 [95% CI 40–45] years; 40–94%TBSA range; 86% previously mechanically ventilated) demonstrated a higher retention of QMLT and RF-CSA after 6 weeks (RF-CSA: β-coeff.: 0.05 cm^2^, *p* = 0.045) and a faster recovery from 6–12 weeks (RF-CSA: β-coeff.: 0.04 cm^2^, *p* = 0.016) as a result of exercise. Early exercise led to a 19.5% larger increase in lower limb muscle strength between 6–12 weeks than standard care alone (*p* = 0.001). Besides the significant effect of the BSHS-B domain from 6–12 weeks, group allocation did not significantly impact the assessed quality-of-life parameters. Few minor adverse events were reported in the exercise group.

**Conclusions:**

The results of this study lend support to the inclusion of exercise as an essential part of the acute management of postburn muscle wasting in adults with severe burn injuries.

**P080**
**A Prospective, Single-Centre Randomised Controlled Study for the Effects of Microcurrent Therapy on the Prevention and Healing of Brachioplasty Scars: Preliminary Results**
**Mieke Anthonissen** ^1,2,3^, Thibau Demarbaix ^1^, Cindy Lafaire ^1,4^, Lieve De Cuyper ^1,4^, Jill Meirte ^1,3^, Ulrike Van Daele ^1,3^, Koen Maertens ^1,5^, Peter Moortgat ^1^
^1 ^ OSCARE, Organisation for Burns, Scar After-care and Research, Antwerp, Belgium
^2 ^ KULeuven, Department of Rehabilitation Sciences, Leuven, Belgium
^3 ^ University of Antwerp, Department of Rehabilitation Sciences and Physiotherapy, Antwerp, Belgium
^4 ^ ZNA Stuivenberg, Antwerp, Belgium
^5 ^ Vrije Universiteit Brussel, Department of Clinical and Lifespan Psychology, Brussels, Belgium
**Aim:**

We aim to investigate the effects of microcurrent therapy in patients after bilateral brachioplasty surgery.

**Methods:**

To date, we have enrolled 16 patients, of which 9 patients have completed all assessments. One arm was randomly allocated to the intervention group, and the other arm to the control group. Both groups received standard wound care and hydration. The intervention group received additional microcurrent therapy for 6 weeks. Scars were measured at baseline and after 3 months and 6 months for colour (Mexameter^®^), hydration (Corneometer^®^), elasticity (Cutometer^®^), and POSAS 2.0. Statistical analyses included a general linear mixed model and paired-sample t-tests.

**Results:**

No significant group differences were found over time for objective measurements. However, a positive trend was observed after 3 months for elasticity in the intervention group, compared to a decrease in elasticity within the control group.

For POSAS patients, a significant improvement was observed after 3 months for itching, colour, texture, global opinion, and sum of scores (*p* ≤ 0.048). Analyses for POSAS observers showed significant improvements after 3 months in pliability, surface, global opinions and sum of scores (*p* ≤ 0.040).

**Conclusions:**

The positive trend in elasticity is confirmed by the POSAS observer pliability. The lack of significant differences in objective measurements might be due to the relatively short treatment period and small sample size. Therefore, we aim to expand our sample size to 20 patients. Microcurrent therapy might be useful and safe to start in the early stages after wound closure following brachioplasty. It is advisable to continue microcurrent treatment over a prolonged period.

**P082**
**Personal and Public Involvement in Developing a Health-Literate Scar Treatment Website**
**Evert De Rijcker** ^1^, Jill Meirte ^1,2^, Mieke Anthonissen ^1,2,3^, Lisa Senepart ^1^, Peter Moortgat ^1^, Koen Maertens ^1,4^, Thibau Demarbaix ^1^, Ulrike Van Daele ^1,2^
^1 ^ OSCARE, Organisation for Burns, Scar After-care and Research, Antwerp, Belgium
^2 ^ Department of Rehabilitation Sciences & Physiotherapy, Research group MOVANT, University of Antwerp, Antwerp, Belgium
^3 ^ Department of Rehabilitation Sciences, University of Leuven, Leuven, Belgium
^4 ^ Vrije Universiteit Brussel, Clinical and Lifespan Psychology, Brussels, BelgiumVrije Universiteit Brussel, Clinical and Lifespan Psychology, Brussels, Belgium
**Aim:**

Our aim is to develop an informative website, ‘howtotreatscars.com’, for patients with burn scars who are looking for treatment options for their scars, and to evaluate whether this website meets health-literate criteria.

**Method:**

Patients and caregivers completed five questionnaires, including three subdomains: knowledge transfer, treatment plan agreement, and health literacy aspects. These questionnaires assessed changes in scar management knowledge together with the accessibility and user-friendliness of the website for which the Patient Education Materials Assessment Tool (PEMAT) was used. One questionnaire also investigated the agreement between patients and caregivers in their search for the best treatment option(s).

**Results:**

Out of 20 respondents, 66% of the patients never received scar treatment in a specialized aftercare center. Almost half (43%) of the patients performed a search for treatment options on the internet before visiting their caregiver.

All respondents can be considered digitally literate.

Related to knowledge transfer, we noticed a statistically significant improvement (*p* = 0.004) in the knowledge assessment after visiting the website compared with the pre-visit assessment.

Some 91% of the patients and their respective caregivers agreed on the best therapeutic solution(s).

The PEMAT score was 96%, but 15% of the respondents indicated the usage of too much medical jargon. All respondents found the website to be well designed and user-friendly.

**Conclusions:**

The website induced knowledge transfer and treatment plan agreement, is user-friendly and well designed, and has a high PEMAT score. The remarks on medical jargon encouraged us to develop a second version of our scar treatment website in terms of content, in collaboration with patients and caregivers.

**P083**
**Reconstruction of the Cervical Region in Patients with Severe Postburn Contractures Using Free Flaps: A Case Report**
**Mirela Nistor**, Enara Reola Ramírez, Josep María Martí Ayats, Alba Sobrino Casorrán, Manel Alòs Blanco
Miguel Servet Hospital, Zaragoza, Spain
**Aim:**

To describe our experience with a case of severe postburn contractures in the cervical and inferior facial regions causing debilitating functional deformities.

**Methods:**

A 48-year-old woman who suffered third-degree burns of the upper body including the, face, neck, thorax, abdomen and upper limbs was referred to our burn unit for treatment. The management of the acute phase consisted of various sessions of enzymatic and mechanical debridement and posterior coverage with split-thickness skin grafts. One year after hospital discharge, she developed a severe mentosternal contracture, causing eversion of the lower lip with eating dysfunction associated with an important reduction in cervical range of motion. Initially, a full-thickness scar excision was performed and a synthetic double-layer dermal matrix combined with a split-thickness skin graft was used for the coverage of the skin defect. Due to unsatisfactory results in the postoperative period with recurrence of the mentosternal contracture, a new reconstruction with a SCIP free flap was performed.

**Results:**

Six months after the surgery, the patient is able to accomplish a full extension of the cervical region. The lower lip eversion was corrected, and the patient presents with normal oral function.

**Conclusions:**

The primary aim of the facial and cervical postburn reconstructions is to restore the function of the affected areas, whilst at the same time optimizing aesthetic outcomes (1). Due to its dermal qualities and low donor-site morbidity, the SCIP flap is a good option for this type of reconstruction when there are no other loco-regional options available (2, 3).

**P084**
**A Breakthrough in Eyelid Reconstruction: The Use of Pure Skin Perforator Flaps in Severe Facial Burns**
**Carlos Pueyo-Morillo**, Jordi Serracanta, Antonio Bulla, Danilo Rivas, Juan-Pedro Barret
Vall d’Hebron Hospital, Barcelona, Spain
**Aim:**

Scarring of the eyelids after severe facial burns can cause several complications including incomplete eyelid closure and corneal exposure, which can ultimately lead to keratitis, corneal opacification, and visual impairment. The gold-standard treatment with skin grafting and tarsorrhaphy can sometimes result in poor graft survival, hypertrophic scarring, and graft contraction, which does not resolve the problem.

The pure skin perforator flap is a recent breakthrough in the field of reconstruction. It presents the anatomical characteristics of a full-thickness skin graft while preserving the benefits of a vascularized free flap, allowing no flap contraction and complete survival of the grafted skin.

**Methods:**

We describe the case of a 50-year-old patient affected with severe burns to 22% of the body and face, resulting in recurrent bilateral ectropion despite various skin grafts to both upper eyelids.

**Results:**

We performed bilateral replacement of the upper eyelid skin in two different procedures, one for each eyelid, using a pure skin perforator flap obtained from the groin which was then anastomosed to the superficial temporal vessels in the left eye and the supraorbital vessels in the right eye. This approach yielded successful results, and the patient achieved complete closure of both eyelids without any complications.

**Conclusions:**

In conclusion, the pure skin perforator flap is a viable option for eyelid reconstruction in patients with severe facial burns. It offers several advantages over grafts, such as preserving the vascular supply to the graft and no contraction, making it an excellent choice for extensive eyelid reconstruction.

**P085**
**Combination Treatment Using Non-Ablative Fractional Laser and Intralesional Triamcinolone Injection for Hypertrophic Scars and Keloids**
**Jongweon Shin**
Eunpyeong St. Mary’s Hospital, College of Medicine, The Catholic University of Korea, Seoul, South Korea
**Aim:**

Combinations of various treatment methods have been shown to be more effective than monotherapy when treating hypertrophic scars and keloids. This study was conducted to assess the effectiveness of combination therapy with a non-ablative fractional laser and an intralesional steroid injection.

**Methods:**

A total of 38 patients with hypertrophic scars or keloids were evaluated. The control group of 21 patients received a steroid injection alone, and 17 patients (the combined group) received a 1550 nm erbium glass fractional laser and steroid injection simultaneously. All treatments were scheduled every four weeks. The results were evaluated by analyzing the total number of treatment sessions, Patient and Observer Scar Assessment Scale (POSAS), recurrence rate, and remission period.

**Results:**

The mean number of treatment sessions was statistically fewer in the combined group than the control group (6.95 vs. 5.47, *p* = 0.042). There was a significant difference in the patient’s scale in the combined group (14.62 vs. 22.82, *p* = 0.005); however, the observer’s scale score showed no significant difference (17.92 versus 20.55, *p* = 0.549). The total score showed the same tendency as the patient’s scale (32.54 versus 43.36, *p* = 0.041). The recurrence rate was 38.1% (8/21) and 35.3% (6/17), in the control and combined group, respectively, with no significant difference (*p* = 0.859). However, the mean remission period was statistically longer in the combined group (3.00 months vs. 4.17 months, *p* = 0.042).

**Conclusions:**

Combination therapy with a non-ablative fractional laser and an intralesional steroid injection showed better results for the treatment of hypertrophic scars and keloids, with fewer treatment sessions, better patient satisfaction, and longer remission periods.

**P086**
**Distal Ulnar Artery Fascial Flap: a Vascular Study and Surgical Considerations**
**Albin Stritar**, Klemen Lovšin, Luka Emeršič
UMC Ljubljana, Ljubljana, Slovenia
**Aim:**

The distal ulnar artery flap is a fasciocutaneous flap raised on the ulnar side of the wrist and forearm. Its axial pattern of vascularisation is based on a cutaneous branch of the ulnar artery.

**Methods:**

We performed surgery on five patients, using the distal ulnar arterty fascial flap: three patients were operated upon due to palmar burn scarring, one patient was upon operated by using the wrap-around flap of the median nerve for better regeneration, and one patient was operated upon by using the gliding flap in the wrist area. Our study was additionally supported by a cadaveric dissection study of fascia vascularisation with contrast material.

**Results:**

Our clinical practice has found the fascia to be an excellent gliding flap for tendons and a wrap-around flap for the median nerve. The flap is used for resurfacing in cases such as burn scar resection and finger contracture release. In combination with a dermal matrix and sequenced with a skin graft, the flap proved to be very suitable.

**Conclusions:**

Depending on indications of the selected patients, we employed the fascial flap as the method of choice. A simple arc rotation of the fascial flap combined with an epifascial soft-tissue layer permits the coverage of the palmar dorsal region of the hand. Subcutaneous tissue should be left on the fascia, as the deep fascia is included in the flap.

**P087**
**Evaluation of Healing with the Use of a Cellulose Substitute (Epicite) in Patients with Deep Second-Degree Burns**
**Enrique Antonio Chau Ramos**, Guillermo Cecchi, Andres Wiegering Cecchi, Gustavo Rene Salcedo Molina
**Objective:**

To compare healing between regenerated cellulose skin substitutes (EPICITE) and xenografts in patients with second-degree burns.

**Methods:**

We report 60 cases evaluated in a hospital in Peru between January 2018 and December 2022 in patients between 1 and 60 years of age and without comorbidities, wherein an evaluation of the healing in patients with second-degree burns caused by hot liquid was recorded. This is a comparative, intervention, analytical, prospective, and longitudinal study. The two skin substitutes were used at the same time in all patients. This study has the authorization of each patient through informed consent.

**Results:**

At 90 days, an evaluation was made, showing that better healing was achieved with the synthetic cellulose skin substitute (EPICITE) compared to xenograft. The healing results were evaluated using the Vancouver scale (for vascularity, pigmentation, flexibility, and height); use of the synthetic dermal cellulose substitute resulted in less redness and greater elasticity, which are the most prevalent indicators.

**Conclusions:**

The study showed that the synthetic cellulose skin substitute is an important alternative that favors superior healing in burnt areas; it is more efficient than the xenograft when evaluated and compared on four parameters using the Vancouver International Healing Scale. The results show that (EPICITE) is an efficient alternative in the treatment of second-degree burns and favors a better healing process.

**P090**
**Management of Full-Thickness Skin Defects Due to High-Energy Trauma: Five-Year Clinical Experience with an Acellular Dermal Substitute**
**Maya Argirova** ^1^, Anastasiya Viktorova ^1^, Yolanda Zayakova ^2^
^1 ^ Umhatem “n.i.pirogov”, Sofia, Bulgaria
^2 ^ MMA, MBAL, Varna, Bulgaria
**Aim:**

To analyze prospectively the efficiency of an acellular dermal matrix applied to full-thickness skin defects due to high-energy trauma.

**Methods:**

Retrospective review between August 2018 and December 2022 covering 28 patients with full-thickness skin defects of the upper and lower extremity, treated with an acellular dermal substitute and an unmeshed skin graft in a single-step procedure.

**Results:**

A total of 28 patients were enrolled in this study. The mean age was 21 ± 14.29 years. The causes of the defects included degloving injury and high-energy trauma. The size of the soft-tissue defects was between 50 and 500 cm^2^. There were no complications or inflammatory responses in all cases but three, which resulted in a seroma. All wounds were epithelized within 11 days without additional grafting. The overall survival rate of the matrix and the skin graft was 97%. The average VSS was 1.97.

**Conclusions:**

High-energy trauma is caused by force (traffic injuries, crush, workplace accidents etc.). A high amount of kinetic energy is applied to the tissue. The management of those kinds of injuries is complex, and requires careful planning to provide stable coverage using the safest and least invasive method. Acellular dermal substitutes represent a valuable alternative to other types of defect coverage, and should be considered in the treatment of skin injuries.

**Keywords:**

acellular dermis, artificial skin substitutes, high-energy trauma, soft-tissue defect

**P091**
**Enzymatic Debridement with Nexobrid® in Deep Facial Burns: Our Experience in Vall d’Hebrón Hospital Burn Unit**
**Alex Arteaga Pérez**, Jordi Serracanta Domenech, Joan Pere Barret Nerin
Vall Hebrón Hospital, Barcelona, Spain
**Aim:**

Some of the most frequent sites of burns are the face and neck, with a prevalence of between 27 and 58%. Due to the importance of these anatomical territories in aesthetic and functional terms, it is important to preserve the maximum amount of viable dermis to improve the quality of the scarring process. Enzymatic debridement has revolutionized the management of deep burns, showing favorable results in the literature. Despite this, to date, there are few publications regarding the management of facial burns with enzymatic debridement (Nexobrid^®^) and subsequent wound treatment.

The objective of the study is to present our experience with enzymatic debridement (Nexobrid^®^) performed on deep facial burns.

**Methods:**

From December 2019 to March 2023, 11 enzymatic debridements with (Nexobrid^®^) were performed on patients with deep facial burns in our burn unit.

**Results:**

Data from 11 patients (7 men and 4 women) were collected. Their mean age was 43 years. The most frequent etiology of burns was flame (9). In all patients, a single session of (Nexobrid^®^) was performed. The wound care protocol applied was honey in 10 of 11 patients; 8 patients epithelialized spontaneously, with a mean epithelialization time of 23 days. The other three patients needed surgery only in third-degree burn areas.

**Conclusions:**

Enzymatic debridement with a subsequent wound care protocol with honey has proven to be a safe and effective option in the treatment of deep facial burns, allowing spontaneous epithelialization in up to 73% of patients

**P092**
**A Novel Personalized Burn Treatment: An In Situ Electrospun Nanofiber 3D Scaffold with Cultured Autologous Keratinocytes**
**Marina BenShoshan** ^1^, Ayelet Di Segni ^1^, Moti Harats ^2,3,4^, Claudia M. Barzilay ^5^, Josef Haik ^1,2,3,4^
^1 ^ The Green Skin Engineering Center, The Intensive Care Burn Unit, Sheba Medical Center, Ramat Gan, Israel
^2 ^ The Division of Plastic & Reconstructive Surgery and The Intensive Care Burn Unit, Sheba Medical Center, Ramat Gan, Israel
^3 ^ Sackler School of Medicine, Tel Aviv University, Tel Aviv, Israel
^4 ^ Talpiot Leadership Program, Sheba Medical Center, Tel Hashome, Ramat Gan, Israel
^5 ^ Nanomedic Technologies Ltd., Lod, Israel
**Aim:**

We present the case of a 26-year-old male with full-thickness burns covering 98% of his total body surface area. We propose a novel personalized burn treatment approach: an in situ electrospun nanofiber 3D scaffold with cultured autologous keratinocytes.

**Methods:**

We sprayed an in situ electrospun polymer nanofibrous matrix (EPNM) directly onto cultured epithelial autograft (CEA) areas. In addition, we proposed a personalized treatment for hard-to-heal areas, in which we sprayed suspended autologous keratinocytes integrated with in situ 3D EPNM directly onto the wound bed. This method enables the coverage of larger wound areas than those possible with CEA.

**Results:**

We were able to show that this treatment approach resulted in good epithelization, seen as early as seven days post-CEA grafting, with complete wound closure within three weeks, and to a lesser extent in areas treated with cell spraying. Moreover, in vitro experiments confirmed the feasibility of using keratinocytes embedded within the EPNM. Cell and culture viability, identity, purity, and potency were determined.

**Conclusions:**

These experiments show that the skin cells are viable and can proliferate within the EPNM. The results presented herein are of a promising novel strategy for the development of personalized wound treatment, integrating on-the-spot “printed” EPNM with autologous skin cells, which will be applied at the bedside over deep dermal wounds to accelerate healing time and wound closure.

**P093**
**The Meek Micrograft Technique in a Romanian Functional Burn Center**
**Anca Bordianu** ^1,2^, Irina Zaharia ^1^
^1 ^ “Bagdasar-Arseni” Clinical Emergency Hospital, Bucharest, Romania,
^2 ^ “Carol Davila” University of Medicine and Pharmacy, Bucharest, Romania
**Aim:**

To raise awareness of the clinical application of the Meek micrograft technique on difficult-to-manage patients.

**Methods:**

This paper illustrates four cases of patients with burned lesions, of less than 25% of the total body surface area, admitted to a Romanian Functional Burn Center, with limited therapeutical options. The Meek micrograft technique was applied in combination with mesh skin grafting.

**Results:**

All patients had good outcomes, despite their difficulty in understanding the explained information and the suggested surgical treatment, with fast healing of their recipient sites acting to decrease the infectious and systematic complications associated with burns. This way, they experienced an alleviation of their functional, aesthetic and emotional status, which also improved their cooperation with the medical team.

**Conclusions:**

The Meek micrograft technique might be used in addition to mesh skin grafting in uncooperative patients as an useful tool to increase survival rates.

**P094**
**Using Cryopreserved Cultured Cutaneous Allografts in the Surgical Management of Extensively Burned Patients**
**Jose Joel Casas Beltran**
Mexican Institute of Social Security, Hermosillo, Mexico
**Aim:**

To assess the re-epithelialization time of donor and mesh skin areas covered with cryopreserved cultured cutaneous allografts in patients with extensive burns.

**Methods:**

We assessed the records of patients treated with extensive burns that had an indication for surgical excision and skin grafting covered with cryopreserved cultured cutaneous allografts. The donor site areas taken from the limbs and torso were between 0.008 and 0.015 inches and 4 × 6 inches wide. The graft expansion ratio for burns requiring skin grafting was 1:1.5 < 11% TBSA, 1:3 between 11 and 20% TBSA, and 1.6 or 1.9 for TBSA exceeding 20%. The re-epithelialization time, infection rate, and complications were recorded. The exclusion criteria were patients with previous immunosuppression or diabetes mellitus.

**Results:**

A total of 53 patient records were included. The donor site areas were the limbs (82%) and torso (18%). The average healing time required for donor sites such as the limbs and torso was 7.32 ± 0.13 and 8.04 ± 0.54 days, respectively. The time taken for complete restoration of meshed skin was 9.03 ± 2.3 days for a 1:1.5 expansion ratio, 10.5 ± 1.69 for 1:3, and 13.4 ± 2.0 for a 1.6 expansion ratio. No examples of a 1.9 expansion ratio were collected. There were four cases with graft loss, and two cases with the presence of infection.

**Conclusions:**

The use of cryopreserved cultured skin allografts at the donor site of dermal/epidermal skin grafts allows for faster re-epithelialization of new grafts. It has also demonstrated effectiveness in faster full restoration of mesh areas, and prevents infections, making it a great option for the surgical management of extensively burned patients

**P095**
**Acellular and Lyophilized Fish Dermis for the Management of the Open Abdomen**
**Alfredo Cordova**, Samuel Miller, Kevin Liu, Nicole Greenwood
Sarasota Memorial Hospital, Sarasota, USA
**Aim:**

The management of the open abdomen is challenging, and may carry a significant risk of morbidity, including entero-cutaneous fistula formation. Biologic and synthetic skin substitutes may allow for the development of an optimal wound bed for grafting, or may provide temporary wound coverage. The ultimate goal is to achieve an ideal skin substitute that provides an effective and scar-free wound healing. Decellularized and lyophilized north Atlantic cod fish skin is a promising alternative, as seen in this case series.

**Methods:**

A 32-year-old female with history of alcohol abuse and cirrhosis presented with multiorgan failure, septic shock, and abdominal compartment syndrome. She underwent a decompressive laparotomy and subsequent abdominal closure with a Vicryl mesh. A 51-year-old female underwent a liposuction procedure which was complicated by bowel injury, resulting in an NSTI and septic shock. She underwent multiple wide debridements and subsequently abdominal closure with a Vicryl mesh. Lastly, a 76-year-old female underwent a sigmoid resection with Hartman’s colostomy for perforated diverticulitis. She experienced fascial dehiscence. All these abdominal wounds with underlying bowel injury were resurfaced with Xenograft and subsequent autologous STSG.

**Results:**

Xenograft integration and optimal granulation tissue was evidenced in >95% of the surface area as early as 5 days after the product’s application. This was considered ideal for resurfacing. Skin coverage with meshed STSG revealed nearly 100% skin graft take in all cases.

**Conclusions:**

Decellularized and lyophilized fish dermis may provide an excellent option for wound coverage and enhancing the formation of the optimal wound bed for grafting. Further studies may be carried out to replicate these findings.

**P096**
**Use of Collagen Elastin in Bite Wounds in Psoriatic Patients**
**Jose Cordova-Orrillo**, ruth victoria lobaton–rosas
Clinica Delgado Auna, LIMA, Peru
**Aim:**

Psoriasis is a systemic, chronic inflammatory process in the skin; the use of oral and topical corticosteroids to reduce the progress of and impact that these lesions have on our patients is very common.

Wound healing in these lesions is slow and torpid, producing a deterioration in patients’ quality of life and social relationships.

The objective of this presentation is to propose a new non-surgical tool for this type of patient with complex wounds.

**Methods:**

We present the cases of two patients with sequelae of canine bites in the lower limbs, post-autograft and total loss of the same in both cases. On a chronic wound lasting approximately 1 month, we proceeded to perform topical cures with use of MatriDerm and dressing changes every 10 days.

**Results:**

Total closure of the lesion in 40 days.

**Conclusions:**

MatriDerm is a one-step dermal substitute for full-thickness skin defects in combination with autografts. MatriDerm consists of a type I, III and V bovine collagen fiber sheet that incorporates elastin hydrolyzate that is converted to collagen in the weeks after application. The matrix can be stored at room temperature and is presented in 1 mm, 2 mm, 3 mm thick sheets. There exists no description of an exposed collagen and elastin matrix for wound closure. An option is presented herein for the closure of complex wounds in cases in which the possibility of grafts or flaps does not exist due to the patients’ comorbidities or due to tissue conditions such as long-term psoriasis.

**P097**
**Using Fish to Replace Deep Wound Defects**
**Anthony de Buys Roessingh**, Oana Sciboz, Marie Doan
CHUV, Lausanne, Switzerland
**Purpose of the study:**

Tinea capitis is a dermatophytosis of the scalp whose transmission happens via humans and animals. Its clinical appearance can range from seborrheic dermatitis to kerion. Its treatment is long-term, with oral antifungal medicines and associated shampoo. We report a case of inappropriate incision/excision of a kerion and the use of Kerecis^®^ as a dermal and epidermal substitute to cover the incised area. A case of Verneuil disease with large excision of the skin is also reported, with the use of Kerecis^®^ to cover the incised area.

**Methods:**

A seven-year-old patient was diagnosed with an abscess of the scalp. Incision was carried out by a dermatologist with a final diagnosis of Trichophyton mentagrophytes. The evolution was unfavorable, with significant loss of substance of the scalp. In order to attempt a primary closure of the scalp, we applied a dermal substitute based on fish skin, Kerecis^®^, which is rich in Omega 3 polyunsaturated fatty acids. A large excision of the skin was accomplished in a young woman suffering from Verneuil disease, with the use of Kerecis^®^ as a dermal and epidermal substitute to cover the incised area.

**Results:**

After two to three applications of the fish substitute and multi-weekly dressings, the defects of the scalp (case 1) and of the axillary area (case 2) diminished, with a reduction of the defect and closure of the skin.

**Conclusions:**

Kerecis^®^ is a therapeutic alternative for the treatment of loss of substance in children.

**P098**
**Enzymatic Debridement with Bromelain for Chemical Burns: A Case Report**
**Nerea Díaz Ros**, Pedro Alvedro, Fátima Rodríguez, Ana Vidal, Maria Dolores Pérez del Caz
**Introduction:**

Bromelain consists of an enzyme which selectively removes the eschar of mid- to deep-thickness burns and leaves a suitable bed that facilitates spontaneous re-epithelization. While it is widely used to remove the eschar of burns caused by fire or heat, there is limited experience and, therefore, limited literature about the use of bromelain for chemical burns.

**Aim:**

We describe the results of the use of bromelain for the enzymatic debridement of a chemical burn.

**Methods:**

Our patient presented a third -degree burn that extended from thorax to the abdomen (4% of the total body surface), and other burns of diverse severity in different parts of the body, including the face, right arm and hand, left arm, and both thighs. The total body surface burned was 8%. The mechanism of the burns was chemical (with sulfuric acid), due to a work accident. After 24 h of wet dressing with Prontosan, we applied 7 gr of Nexobrid to 4% of the body surface (thorax and abdomen). We removed Nexobrid after 4 h, and a new wet dressing was applied. The definitive dressing was carried out with Mesitran.

**Results and conclusions:**

The use of bromelain is suitable for the enzymatic debridement of chemical burns, even though studies that compare its efficacy to other types of debridement and further literature are needed to confirm its applicability.

**P099**
**Reverse Circumcision: A Novel Technique for Skin Coverage in Patients with Circular Burn in the Shaft of the Penis**
**Jorge Antonio Guerrero Montes**, Mario Vélez Palafox, Rául Gilberto Rivera Sánchez, Osiris Cristina Chávez Flores, Luis Tamez Pedrosa
Instituto Nacional De Rehabilitacion, Queretaro, Mexico
**Purpose:**

There are various techniques described for the coverage of penile body defects, such as partial-thickness skin grafts, the use of dermal matrices, and the use of locoregional flaps up to radial free flap. These procedures are not innocuous, since they generate secondary defects, aesthetic alterations, and sensitivity. Therefore, we present our technique for the use of excess foreskin as a sensitized skin cover.

**Methods:**

We report the case of a 40-year-old male patient who presented with an electrical burn of 30% TBSA. He presented a circumferential burn of the penis of 4 cm in length. Surgical debridement of the skin from the shaft of the penis to Buck’s fascia was performed, generating a defect of approximately 4 cm in circumferential skin height. The foreskin was proximally pulled, uncovering the glans penis, and the circular defect was completely covered. The base of the penis was sutured with 3-0 monocryl in the subdermal plane with a simple inverted stitch, and in the epidermal plane with a 3-0 monocryl, simple stitch. The shaft of the penis was covered with a Vaseline dressing and a secondary dressing.

**Results:**

The patient’s penis was observed 24 h later, evidencing the vitality of the skin of the shaft, with no signs of hematomas or seromas. The patient reports maintaining sensitivity in the skin of the shaft of the penis from 7 days after surgery, without alterations in micturition or sexual function.

**Conclusions:**

Our reverse circumcision technique is a simple technique that should be considered primarily in any center when faced with skin defects of the shaft of the penis.

**P100**
**Use of Polylactic Acid Dressings in Donor Areas of Skin Graft Patients with Severe Burns to Improve Their Recovery**
**Jorge Antonio Guerrero Montes**, Mario Vélez Palafox, Rául Gilberto Rivera Sánchez, Osiris Cristina Chávez Flores, Luis Tamez Pedroza
Instituto Nacional De Rehabilitación, Ciudad de Mexico, Mexico
**Purpose:**

Major burn patients (>40% TBSA) have few skin injection donor areas for skin coverage; therefore, unusual areas such as the soles of feet and scalp are taken from. The rapid recovery of these areas limits the waiting times between surgeries, which is why new strategies are being sought to improve the recovery process in these patients. We present an example of the use of polylactic acid dressings in these patients.

**Methods:**

We assessed a 38-year-old female patient, who presented with a 60% fire burn and a history of multiple surgeries wherein tangential excision was performed and skin grafts were taken and applied. However, she did not present usual graft donor areas, so the scalp was shaved and partial-thickness skin grafts were taken. An 18 × 23 cm sheet of lactomer–capromer (polylactic acid) was applied, fixed with surgical steel staples, and covered with a Vaseline dressing, gauze dressing, and bandage.

**Results:**

The patient underwent surgery to the grafted areas, and showed complete epithelialization of the scalp after 7 days.

**Conclusions:**

The use of a lactomer–capromer dressing (polylactic acid) improved the epithelialization process of the skin donor area, once again allowing partial-thickness grafting in a patient who needed to accelerate their healing process with scarce skin donor areas.

**P101**
**Post-Trauma, Non-Healing Lesions of the Leg Treated with a New Debriding Compound: A Retrospective Proof-of-Concept Study**
**Dr. Michel Hermans**
Hermans Medical Consulting, Hoorn, The Netherlands
**Aim:**

A new debriding compound (TDA) has been designed to rid wounds of biofilm and necrosis in a one-time intervention, leading to faster granulation tissue development and subsequent healing via secondary intention or grafting. TDA was primarily designed for the treatment of ulcers, but the compound has also been tested in post-trauma, non-healing (PTNH) wounds.

**Methods:**

In a retrospective study, the results of a one-time application of TDA were assessed. TDA was applied once, followed by a Vaseline gauze treatment. The primary outcomes studied were time to complete granulation and adverse events. Time to re-epithelialisation was also assessed.

**Results:**

Nine lesions in nine outpatients (average age: 77.9 years) were studied. Lesions were 15.9 cm^2^ and 5.6 months old on average, and located on the lower leg.

The average time taken to complete granulation was 34.1 days, and time to re-epithelialisation was 69.8 days, with 11.9 visits to the clinic on average. There were no adverse events.

**Conclusion:**

Typically, PTNH lesions take much time to heal and require many interventions. This study showed that a single TDA treatment with subsequent “neutral” dressings leads to healing in a relatively short time, with few clinic visits. The latter aspect may also result in overall cost reductions. We extrapolate and hypothesize that TDA treatment might also be effective for the treatment of old, non-healing burns, and those full-thickness burns that are too small to surgically excise. The fact that TDA can be used in the outpatient clinic or even in home care is an additional advantage.

**P102**
**Reverse Technology in the Treatment of Burns: The Use of Tropical Fruits**
**Helma Hofland** ^1,2^, E.E Zijlstra ^1^
^1 ^ Rotterdam Center for Tropical Medicine, Rotterdam, The Netherlands
^2 ^ Maasstadziekenhuis, Rotterdam, The Netherlands
**Aim and methods:**

We describe how traditional African medicine plays a major role in the treatment of burn injuries in most regions in Africa. While honey is mainly used for its antiseptic effect, other products such as papaya (carcia papya) are used to clean full-thickness wounds of infection and debris, or to treat wounds with hypergranulation. The pulp of mashed papaya is applied on a dry gauze daily or every other day.

**Results:**

Papaya appears to be effective in wound debridement, preventing infection, and stimulating the development of a granulating wound suitable for a split-skin graft. Furthermore, it is also effective in reducing hypergranulation. These natural products have formed the basis of the development of a new treatment modality for burns (NexoBrid^®^, MediWound Ltd.) in high-income settings, as an example of reverse technology. Its mode of action is based on the same enzymatic activity of papaya (and similarly, pineapple and kiwi) with the aim of promote healing via debridement of the wound.

**Conclusion:**

In low-resource settings, natural products derived from tropical fruits are easy to use, safe and effective in the treatment of burns, widely available, and cheap. In these settings, treatment with honey and papaya continues to be the basis of burn care with excellent results. These principles have now been successfully adopted in high-income settings.

**P103**
**A Step-by-Step Guide: How to Apply Dressings Suitable for the Severely Burned Patients**
**Nurse Alice Rimmen**, Nurse Pia Høy
Copenhagen University Hospital, Department of Plastic Surgery and Burns Treatment, 6052, Copenhagen Ø, Denmark
Aim:

To improve dressings for severely burned patients in Denmark that allow the patient to maintain physical functions while preventing infections, managing the level of exudate, and preventing unnecessary pain and unnecessary dressing changes.

**Methods:**

We (Department of Plastic Surgery and Burns Treatment, Copenhagen University Hospital, Denmark) have worked together with the Trauma Department to develop a visual “step-by-step guide” to show how to apply appropriate dressings to every region of the body. We have also produced a “tips and tricks guide” to make the process easier and avoid common mistakes.

These guides have been distributed throughout our own department, trauma department, burns operating theatre and the burns clinic. These guides have also been (and will also be) uploaded to the department’s burns website (brandsår.dk), so that the emergency departments in the rest of the country can benefit from them.

**Results:**

We have already experienced improvements in the initial dressings. Our colleagues have found it easy to imitate pictures from the guide, and they found it very helpful in their daily work. We have only received positive feedback.

**Conclusions:**

The step-by-step guide has been used for a little while in our department. In our future work with burn victims, we will continue to improve dressings and make more guides, if needed.

**P104**
**Clinical Photography in Burns Using a Secure, NHS-Approved App (ISLA)**
**Mehitab Adel Saeed Hussien**, Ted Welman, Daniel Markeson
Chelsea and Westminster Hospital, London, UK
**Aims:**

Photography is a well-established adjunct to the assessment, documentation, and management of burns. Clinical photographs must comply with GDPR regulations outlined in the Data Processing Act, 2018. We aimed to improve photographic documentation of burns to aid MDT communication, track healing, and increase compliance with data requirements.

**Methods:**

A retrospective staff survey was conducted to assess adherence to medical photography standards between January and March 2023. The survey investigated the frequency of clinical photography, the use of consent forms, and the storage and handling of images. Subsequently, an ISLA protocol was designed for the burns service, and implemented alongside the app.

**Results:**

Briefly, 61% of respondents were doctors, and 38% burns nurses. Some 69% of images were taken on personal devices, often with non-documented verbal consent. The majority used WhatsApp to share the photos, which were rarely saved to patient records.

The introduction of ISLA enabled patients and clinicians to upload photos directly onto their e-records, and for consent to be readily taken and recorded. Photos were taken directly on the ISLA website and therefore not stored on personal devices. The application is currently being utilised in all stages of burn care.

**Conclusions:**

We present our experience using ISLA, a user-friendly, NHS-approved, GDPR-compliant solution for photographic documentation. Implementation has significantly improved the quality and security of photographic documentation, benefiting communication, patient care, and education. Future work is required to ensure that photographic quality is standardized in terms of positioning, lighting, and other parameters. Further research will focus on the effectiveness and sustainability of this approach across multiple clinical settings.

**P105**
**The Management of Burns and Trauma Cases Using the Kerecis Fish Skin Graft in the 2020 Nagorno–Karabakh War**
**Steven Jeffery**
202 Field Hospital (British Army Reserve), Bentley Pauncefoot, Nr Bromsgrove, UK
**Aim:**

To demonstrate the effectiveness of Kerecis fish skin grafts in the management of combat injuries in the 2020 Nagorno–Karabakh war.

**Methods:**

The author went to Armenia in September 2020 at the request of the Armenian government. He brought with him a quantity of acellular fish skin grafts (FSGs) from Kerecis™, and utilised these in many cases of burn and blast injury.

**Results:**

Management with FSG induced wound granulation several days sooner in all cases, and even weeks in some instances, allowing a stepdown in the reconstruction ladder with earlier skin grafting procedures and a reduction in the requirement of flap surgery. No infections were reported in any of the cases in which FSG was used.

**Conclusions:**

FSG presents favourable characteristics for use in austere environments. The product is rugged and robust, lightweight, easy to transport, and requires no complex tools or equipment for use other than standard field medical equipment. Further, the minimal training required for use makes FSG an ideal product for use when an overwhelmingly number of patients may need rapid treatment.

The application of FSG achieved early granulation tissue formation and a good coverage of the underlying wound, resulting in low infection risk. However, as opposed to dressings or other temporizing measures, applying FSG begins the wound-healing process rather than simply buying time.

**P106**
**A Staged Approach to Treating Chronic Burn Wounds and Extensive Post-Burn Knee Contracture with Clinical Suspicion of Marjolin’s Ulcer: A Case Report**
**Jeeyoon Kim**, Eun Young Rha
Eunpyeong St. Mary’s Hospital, Seoul, Republic of Korea
**Aim:**

We present a case of a successfully staged treatment for a chronic burn wound with extensive post-burn knee contracture using a free transverse rectus abdominis myocutaneous (TRAM) flap and split-thickness skin graft (STSG).

**Methods:**

A 72-year-old male with post-burn flexion contracture and a 10 × 3 cm ulcer above his right knee joint presented for treatment. He suffered a burn injury as an infant, resulting in chronic post-burn scar contracture (PBSC), growth disturbance, and flexion contracture. Preoperatively, he had limited range of motion (ROM) of 120 degrees, and a weight-bearing imbalance due to limb length discrepancy. Scar contracture release was performed initially, followed by negative pressure wound therapy for a week, allowing rapid ambulation and ROM recovery. After pathological confirmation without malignancy, a 20 × 10 cm muscle-sparing free TRAM flap was used to cover the 40 × 10 cm resultant defect above the knee joint, and STSGs were used on remaining skin defects. The TRAM flap with 12 perforators was preferred over the deep inferior epigastric perforator due to an extensive preoperative skin defect. Intraoperative indocyanine green angiography showed good perfusion throughout Hartrampf’s zones I-IV, except for the distal margin of zone IV.

**Results:**

The reconstructed flap and graft survived, achieved marked ROM improvement with 170 degrees of active knee extension, and improved the weight-bearing imbalance.

**Conclusions:**

We performed staged surgical treatment for a chronic burn wound with extensive PBSC for obtaining sufficient ROM and pathological confirmation. Extensive resection, rapid ROM exercise and staged reconstruction using free myocutaneous flaps and STSG may enable the successful surgical treatment of PBSC.

**P107**
**Treatment of Mine-Explosive Injuries of the Shoulder and Scapular Area Using Latissimus Dorsi Muscle Flap**
**Professor Olga Kovalenko**, Andriy Zhernov, Anton Kovalenko
Bohomolets National Medical University, Kyiv, Ukraine
**Aim:**

Analysis of the efficacy of using the latissimus dorsi muscle flap (LDMF) in mine-explosive lesions of the shoulder and scapular region.

**Methods:**

During 2022, we observed 11 military personnel with mine-explosive wounds of the shoulder and scapular region. The age of patients was from 28 to 56 years. The injuries were combined with penetrating wounds of the chest, and craniocerebral trauma. The defect areas measured up to 20 cm × 12 cm. Seven patients had open multicomminuted fractures of the humerus with tissue defects, and two patients had a 7 cm defect in the humerus. Three patients had fractures of the scapula with tissue defects. One had a traumatic shoulder tear. All the wounded had external fixation devices. All of them underwent VAK-therapy.

**Results:**

Reconstructive surgeries were performed within 1 to 1.5 months of the injury. To close soft-tissue defects, LDMF was used in seven patients, LDMF and bone grafting with a fragment of the fibula were used in two patients with a humerus defect, and a muscle flap was used in two patients, followed by grafting.

**Conclusions:**

The use of an LDM flap in patients with MEI is an effective method of skin restoration in cases of damage to the shoulder and scapular area. It also makes it possible to improve the blood supply of the free bone graft in areas with low vascularity. Keywords: mine-explosive injuries of the humerus, clavicle and scapula; flap of the latissimus dorsi muscle

**P108**
**A Novel Technique of MatriDerm Application to Improve Handling and Ease-of-Use, as Demonstrated with a Case Series.**
**Joanneke Maitz** ^1,2,3^, Lucas Carelli ^2^, Elizabeth Coady ^3^, Duncan Loi ^2^, Peter Maitz ^1,2,3^
^1 ^ Burns & Reconstructive Surgery Research Group, Anzac Research Institute, Sydney, Australia
^2 ^ Burns & Reconstructive Surgery Unit, Concord Repatriation General Hospital, Sydney, Australia
^3 ^ University of Sydney, Sydney, Australia
**Aim:**

MatriDerm^®^, a bovine collagen–elastin dermal template, is currently the only dermal template available that contains elastin. Frequently used in a one-stage procedure with a split-thickness skin graft, this dermal template can improve cell proliferation, accelerate revascularisation, and improve the quality of regenerated tissue. However, its fragile nature makes it a difficult product to handle, especially when in contact with moisture or wound exudate. In this study, a new intra-operative handling technique is described to improve the efficacy and accuracy of the use of fragile dermal templates such as MatriDerm.

**Methods:**

A new application technique was developed by the burns unit of Concord Repatriation General Hospital to deliver MatriDerm onto a well-vascularised wound bed in a single-stage composite step. Five patients, admitted between October 2022 and December 2022 and who received MatriDerm applied with this new technique, are included in this case series.

**Results:**

Using the new composite application technique demonstrated and described in detail, all patients achieved complete wound closure. The application technique allowed for a more efficient operating time, with less correction of skin graft manipulation needed over the MatriDerm. Graft take, wound closure outcomes, and long-term outcomes were not negatively impacted by the technique.

**Conclusions:**

MatriDerm can cause delays in operating time due to its ‘tissue paper’-like handling properties. By applying this composite technique, the ease of use and efficiency were greatly improved. This technique is not only effective for MatriDerm, but may be applicable to any fragile dermal template, thereby improving anaesthetic time and overall operative efficiency.

**P109**
**Facial Burns-Experience and Recommendations of Dr Stanislaw Sakiel Centre for Burn Treatment**
**Karolina Mikuś-zagórska**, Agnieszka Klama-Baryła, Karolina Ziółkowska, Danuta Pawlik-Knapik, Wojciech Łabuś
Dr. Stanisław Sakiel Centre for Burn Treatment In Siemianowice Śląskie, Siemianowice Śląskie, Poland
**Aim:**

Facial burns represent a major clinical challenge. There are a number of therapeutic options for treating a facial burn. The aim of the study was to present the selected procedures used in CLO in the treatment of facial burns.

**Method:**

In 2009, the Dr Stanislaw Sakiel Center for Burn Treatment in Siemianowice Śląskie, Poland began using amniotic membrane grafts for superficial burns, mainly facial burns. Other treatments have also been used, including hydrosurgery and selected skin substitutes.

**Results:**

Studies of the clinical effects of the use of the human amnion showed a different location of the application of this graft. In an adult wound care hospital, the amnion was used to treat wounds extending to the epidermis and dermis, classified as IIA and IIB burns.

**Conclusions:**

Particularly in the case of facial burns, wounds are fully healed after application of the amnion. The great manipulative ability of the amniotic graft also greatly contributes to its clinical use in treating facial burns.

**P110**
**Burn Blisters as Natural Occlusive Dressings? An In Vitro Study**
**Sophia Niedermaier** ^1^, Micheal Cerny ^2^, Ursula Hopfner ^3^, Charlotte Topka ^4^, Niclas Broer ^4^
^1 ^ Universitätsklinikum Schleswig-Holstein Campus Lübeck, Lübeck, Germany
^2 ^ Dr. Lubos Kliniken München, Munich, Germany
^3 ^ Department for Plastic Surgery and Hand Surgery, Klinikum Rechts der Isar, Technical University of Munich, Munich, Germany
^4 ^ Department for Plastic-, Reconstructive-, Hand- and Burn Surgery, Bogenhausen Hospital, Munich, Germany
**Aim:**

The present work measured the influence of burn wound fluid on cell proliferation, inflammation, and angiogenesis to analyze whether burn blisters work as natural occlusive dressings.

**Methods:**

Wound fluid was collected through sterile punction of burn bladders of 29 patients with partial-thickness burns. Blood serum samples were collected from each patient as a control. Eight assimilable patients were selected and their samples were analyzed. A semiquantitative antibody array for multi-cytokine detection focusing on proinflammatory and proangiogenic factors was performed, and the concentrations of IL-8 and IL-6 were measured with an ELISA. The influence of the wound secretion on fibroblasts was assessed via the measurement of cell proliferation (with an Alarmablue assay), cytotoxicity (with an LDH assay), and cell migration (with a scratch assay). The angiogenetic response of endothelial cells (HUVEC) was analyzed with a tube formation assay.

**Results:**

The wound fluid of burn wounds showed a significantly higher cytotoxic potential and lower cell viability of fibroblasts compared to the serum control. There was no difference in cell migration or angiogenesis. Burn blister fluid contained higher levels of the pro-inflammatory mediators IL-6 and IL-8, but this did not reach statistical significance.

**Conclusions:**

Burn wound fluid influences the proliferation and viability of fibroblasts. Like occlusive dressings, intact burn blisters concentrate multiple mediators at the wound site. There was no significant increase in inflammatory mediators, so this study could not find any need to directly deroof burn blisters.

**P111**
**Enzymatic Debridement with NexoBrid® and Its Influence on Management Decisions for Deep Partial-Thickness Burns**
Theodora Ligomenou, Eirini Nikolaidou, **Zoi Tzimorota**, Kristallo Makarona, **Sofia Papadopoulou**
“G. Papanikolaou” Hospital, Thessaloniki, Greece, Thessaloniki, Greece
**Aims:**

Enzymatic debridement of thermal burn wounds with a proteolytic enzyme solution enriched in bromelain, known as NexoBrid^®^, was approved in the European Union in 2012. Ιn Greece, it has been available since 2021. We present our experience of the first 1.5 years of NexoBrid^®^ application.

**Methods:**

We used NexoBrid^®^ on 14 patients (TBSA: 20–50%) and in 18 applications to deep partial-thickness burn wounds. The product is reconstituted and applied with the appropriate analgesic treatment to the burn wounds (5 g/2.5% TBSA), and removed after 4 h. We collected and analyzed the demographic data of patients, the percentage of total body burn surface area treated, the type of burn, time of application, type of analgesia, the healing time, and the need for skin grafting.

**Results:**

No adverse reactions were observed. The treated burn surface area (face, limbs, lower extremities) ranged from 2–20%. The application of NexoBrid^®^ is easy for the physician and effective for the patient, but requires proper pain management and adaptation of the working time flow in the burns ICU. We noticed a reduction in burn wounds that required skin transplantation.

**Conclusions:**

NexoBrid^®^ is a new tool to achieve early non-surgical escharectomy. The next challenge is the appropriate post-application wound management and timing of eventual surgical intervention.

**P112**
**Clinical Effectiveness of Split-Thickness Skin Grafts Using a Porcine-Derived Artificial Dermal Substitute (Insuregraf^®^)**
**Yeonsoon Park**
Bestian Seoul Hospital, Seoul, Republic of Korea
Aim:

To evaluate the clinical effectiveness of one-stage split-thickness skin graft (STSG) procedures using a porcine-derived artificial dermal substitute, Insuregraf^®^ (Hyundai Bioland Co., Republic of Korea) for preventing complications such as joint contractures and hypertrophic scarring following wound healing

**Methods:**

A skin graft was performed on a patient who had suffered third-degree burns measuring 250 cm^2^ on the volar aspects of the forearm and elbow, due to contact burns in September 2022, on the 10th day post-burn.

After removing the eschar, electrocauterization was performed to reduce bleeding. Insuregraf^®^ was then applied to the hemostatic wound area, followed by hydration with saline, and an STSG was performed simultaneously on top of the Insuregraf^®^.

The skin graft was monitored for viability every 3–4 days, and a follow-up observation was conducted on the scar for 7 months.

**Results:**

After two weeks, the graft had completely taken, and for the most part, the scars were flat and well-maintained without significant hypertrophic scarring over a follow-up period of 7 months. There were no symptoms of joint contracture, pain, or itching.

**Conclusions:**

Using a porcine-derived artificial dermal substitute, Insuregraf^®^, for one-stage STSG can minimize hypertrophic scarring and joint contracture.

**P113**
**Acute Kidney Injury in a Severe Burn Patient after Topical Treatment for Wound Contamination**
**Alicia Perez**, Irene Veiga, Alejandro Quinto, Jorge Gamez, Eugenia Lopez-Suso
Complejo Hospitalario Universitario A Coruña, La Coruña, Spain
**Aim:**

To review a rare complication in burn patients based on a clinical case of acute kidney injury due to topical treatment with colistin/gentamicin.

**Methods:**

We assessed a 37-year-old male patient with flame burns over 35% of his body surface area. Enzymatic debridement based on bromelain (Nexobrid^®^) was applied on the day of admission. The patient required ventilator support, vasopressor support with norepinephrine, hemodialysis, and hemofiltration. The patient was extubated on 9th day post-burn (DPB). On the 15th DPB, surgical debridement and grafting were performed.

**Results:**

On the 30th DPB, green exudate consistent with pseudomonas was detected in the dressing of the lower limbs, so topical treatment with colistin and gentamicin was applied. On the 34th DPB, an increase in creatinine was observed. Among the medications prescribed, only metamizole was a possible cause of renal dysfunction. Nephrotoxic drugs were discontinued. Creatinine values deteriorated rapidly and peaked around 9 mg/dL, with preserved diuresis and good volume management. Immune-mediated causes were ruled out, and laboratory tests confirmed supratherapeutic levels of gentamicin, which was identified as the main cause of acute tubulointerstitial necrosis.

**Conclusions:**

Antibiotic ointment is commonly used to suppress bacterial growth on newly grafted tissue, as it is considered non-toxic and maintains the moist wound environment needed for epithelial growth (1). There are very few cases reported in the literature regarding acute tubular necrosis in burn patients caused by topical antibiotic treatment, and currently there are no available treatments (2). Therefore, prevention is the most crucial aspect (3).

**P114**
**Enzymatic Debridement—When? Where? How? Why?**
**Mihaela Pertea**, Madalina-Cristina Fotea, Dorin Sumanaru, Oxana-Madalina Grosu, Stefana Luca
Grigore T Popa, University of Medicine and Pharmacy/Burn Unit, Sf Spiridon, Emergency County Hospital, Iasi, Romania
**Aim:**

This study aims to highlight the indications and results of applying a mixture of proteolytic enzymes with high bromelain content for the treatment of deep burns in a single center, from the perspective of personal experience.

**Methods:**

The study included two groups (one group of 15 patients and another of 30 patients) with burns surfaces between 10 and 45% TBSA and depths of IIB and III. The first group was subject to early excision and skin grafting; for the second group, enzymatic debridement was used. We recorded the effectiveness of enzymatic debridement, the healing time, the need for grafting after debridement, the scars, and patient satisfaction. Both the classic graft harvesting technique with an electrodermatome and the Meek micrografting technique were used.

**Results:**

The enzymatic debridement efficiency was between 85 and 92%. In 8 cases out of 30, skin grafts were necessary. Spontaneous epithelization was noticed in the other 22 cases. The patients recorded less pain, less bleeding, and less damage to surrounding healthy tissues than the group that underwent surgical debridement. The aspect of the scar tissue was better, with relatively good aesthetic results. The present clinical experience shows the several advantages of enzymatic debridement compared with tangential necrectomy.

**Conclusions:**

The introduction of enzymatic debridement as an alternative to surgical debridement for IIB- and III-degree burns has changed standard burn care. This method has the additional benefit of selectivity—the removal of non-viable tissue and the preservation of viable tissue—thereby reducing the number of surgical interventions, infection rates, and days of hospitalization.

**P115**
**Meek Micrografting—A New Old Technique and Its Role and Place in the Treatment of Extensive and Deep Burns**
**Mihaela Pertea**, Stefana Luca, Alexandru Amarandei, Oxana-Madalina Grosu, Madalina-Cristina Fotea
Grigore T Popa, University of Medicine and Pharmacy/Burn Unit, Sf Spiridon, Emergency County Hospital, Iasi, Romania
**Aim:**

The use of the Meek technique has grown on a large scale in the last decades. The aim of this study is to confirm the role and importance of the Meek technique in the therapeutic management of extensive and deep burns.

**Methods:**

We studied a group of 42 patients with deep burns (of the IIB-III degree) and surfaces between 15 and 60% TBSA, produced by flame, hot liquid and contact, with localization on all anatomical regions of the body. In all cases, the indication for early excision and grafting was established. The excision was carried out in a supra-fascial plane or via enzymatic debridement. The grafting was performed using the Meek micrografting technique.

**Results:**

In the studied group, two of the women were pregnant in weeks 22 and 36, with burns of 15% and 18% TBSA respectively. The deep burns were excised early, and the micrografting was performed by expanding in a ratio of 1:3 in 10 cases, 1:4 in 14 cases, 1:6 in 12 cases, and 1:9 in 5 cases, depending on the surface to be grafted and on the existence and quality of the graft donor areas. The micrograft integration rate varied from 82% to 100%.

**Conclusions:**

The Meek micrografting technique is a life-saving solution in the case of deep and extensive burns, but it can also be used in “special” cases (pregnant women, the elderly, children), being able to cover large post-excisional surfaces using narrow donor areas.

**P116**
**Our Experience with Fish Skin Grafts in Full-Thickness Burns—A Clinical Case Series**
**Anna-Lisa Pignet** ^1,2,3^, Martina Carnieletto ^1,2,3^, Lars-Peter Kamolz ^1,2^
^1 ^ Division for Plastic, Aesthetic and Reconstructive Surgery, Medical University of Graz, Graz, Austria
^2 ^ JOANNEUM RESEARCH Forschungsgesellschaft mbH, COREMED—Center of Regenerative and Precision Medicine, Graz, Austria
^3 ^ Research Unit for Tissue Regeneration, Repair and Reconstruction, Division for Plastic, Aesthetic and Reconstructive Surgery, Medical University of Graz, Graz, Austria
**Introduction:**

Fish skin grafts (FSG) have proven to be effective not only in chronic wound healing, but also in burns.

**Aim:**

To perform wound bed modulations using FSG in two patients with heavily colonized full-thickness burns.

**Methods:**

Two patients with heavily colonized full-thickness burns of about 20% TBSA were treated with Bactigras^®^ and FSG, which we meshed on-site. After one week, we performed re-application of the FSG. In the first patient, a young girl who was a war victim in Ukraine, we successfully reduced the bacterial load and increased the amount of granulation tissue on the exposed tendons, so that autologous grafting was finally possible. The second patient underwent deep excision and autologous grafting, which resulted in graft failure. Because the patient was not suitable for another surgery due to various comorbidities, we initiated a wound bed modulation with FSG, again meshed on-site. Only 2 weeks after the first application, the wounds showed good granulation, with only small non-healing spots left, which we decided to micrograft under local anesthesia.

**Results:**

Five weeks after transfer to our center, Patient 1 showed good healing progression in all burned areas, and was able to be transferred to a rehabilitation facility. Patient 2 also showed an excellent healing progression in all burnt areas 2 weeks after grafting.

**Conclusions:**

In our experience, FSG can be used to enhance granulation and vascularization before grafting, or even as an alternative to grafting. Secondly, an adequate graft take rate can be ensured.

**P117**
**Necrotizing Fasciitis within a Burn Center: Lower Limb Salvage**
**Gorka Reyes**, Jordi Serracanta, Carlos Pueyo, Joan-Pere Barret
Hospital Vall d’Hebron, Barcelona, España
**Aim:**

Necrotizing fasciitis (NF) is a rare but rapidly progressive, potentially lethal bacterial infection of the subcutaneous tissue. Many similarities exist between NF, extensive dermatologic pathologies, and burn injuries in terms of the need for meticulous wound care and multidisciplinary management. For this reason, these entities are treated in our burns center.

**Methods:**

We report a case of lower limb NF in a patient with no associated comorbidities. A 42-year-old male patient presented with acute painful swelling of the right knee. After ruling out a fracture, he was discharged with analgesic treatment. Less than 24 h later, he was transferred to the emergency room with hemodynamic instability and hypoxemia.

**Results:**

Five serial debridements were performed. Streptococcus pyogenes was isolated. The patient survived the acute infection and received subsequent reconstruction. For lower extremity salvage, we used a Latissimus dorsi flap, an anterolateral thigh flap, a medial gastrocnemius flap, and skin autografts. For coverage of the superficial femoral vessels, an adductor magnus split was performed. A cutaneous defect on the dorsum of the left hand caused by an extravasation was covered with an SCIP flap. The patient was discharged after three months of hospitalization. The patient is able to walk and is undergoing rehabilitation to regain joint range of motion.

**Conclusions:**

NF is similar to burn wound infection in that treatment requires aggressive surgical debridement, complex wound management, and extensive reconstruction. For this reason, burns centers are ideal sites for the treatment of extensive necrotizing fasciitis.

**P118**
**The Efficacy of Epicite^®^ Hydro-Active Dressings in the Treatment of Burn Wounds: A Multi-Center Study**
**Juan-Bosco Ruiz-Padilla** ^1^, LE Nancy Fonseca-López ^2^
^1 ^ Plastic Surgery Center & Burn Unit, SMA, Mexico
^2 ^ Hospital General Iztapalapa, Secretaría de Salud., CDMX, Mexico
**Introduction:**

Burn wounds can cause significant morbidity and mortality, requiring specialized care to promote healing and prevent complications. Epicite^®^ is a nanocellulose-based dressing that has shown promise in wound healing. This study aimed to evaluate the efficacy of Epicite^®^ in the treatment of burn wounds.

**Methods:**

The medical records of 68 patients with burn wounds treated with Epicite^®^ were reviewed. Of these, 63 patients met the inclusion criteria and were included in the analysis. Data on patient demographics, burn extent and depth, and treatment with Epicite^®^ were collected. The primary outcome measure was re-epithelialization and healing of the wound.

**Results:**

The study population was an average age of 38 years old, with 67% males and 33% females. The maximum extent of the burned body surface area was 37%, and the minimum was 10%. Most patients had deep second-degree burns (60%). Treatment with Epicite^®^ resulted in no infections or complications. The average time to complete re-epithelialization was consistent with the expected clinical results.

**Conclusions:**

This study demonstrates that the use of Epicite^®^ nanocellulose covers in the treatment of burn wounds is safe and effective, with a high percentage of re-epithelialization and no reported complications. Epicite^®^ could be a useful therapeutic option in the management of burn wounds. Further studies with larger sample sizes and longer follow-up periods are needed to confirm these findings.

**Keywords:**

Epicite^®^; nanocellulose; burn wounds; wound healing

**P119**
**Hand Burn Treatment in the Conditions of a Moist Medium**
**Babur Shakirov**, Abdurahim Avasov, Hydoiberdy Karabaev
Samarkand State Medical University, Samarkand, Uzbekistan
**Aim:**

Among thermic injuries of different body parts, hand burns are some of the most common.

**Materials and Methods:**

We analyzed the results of treatment of 31 patients with limited superficial burns of the hand. After primary surgical processing of hand injuries with burns of II-IIIA degree and application of antiseptic solution to all patients, we used sterile disposable polyethylene packs of a large size with a 0.9% sodium chloride solution, fastened with bandages. The evidence of pain syndrome was estimated daily using scores, according to a visual analogous scale (VAS).

**Results:**

In the process of observation, it was established that the pain decreased significantly or was completely eliminated immediately after surrounding the burn injury with the polyethylene pack with sodium chloride solution. In fact, daily bandaging became painless. Assessment of pain syndrome according to VAS showed average scores of 3.8 during the first 24 h, which then decreased to 3.75 scores during the next 24 h, and to 3.06 over the next 3 d 24 h. In patients with second-degree burns, the healing of wounds was noted 5 days later, on average. In patients with third-degree burns, healing required 9 days of treatment. No patients had any clinical sighs of infectious complications during the healing process.

**Discussion:**

The treatment created an optimal microsphere for wound healing, preventing microorganisms’ invasion, retaining sufficient permeability for gases, excluding the possibility of wound floor dryness, and maintaining elasticity.

**P120**
**Deep Burns of Lower Extremities: Epidemiology, Management and Consequences**
**Babur Shakirov** ^1^, Gamshed Karaboev, Erkin Hakimov
Samarkand State Medical University, Samarkand, Uzbekistan
**Aim:**

Severe burns of lower extremities are complex and challenging injuries. The standard of care—necrotomy, necrosectomy with skin grafting—is often associated with poor functional or aesthetic outcomes.

**Methods:**

This is a retrospective study of the epidemiology and management of deep burns of the lower extremities presented to the burn department of RSCUMA and Samarkand Inter-Regional Burn Center, Samarkand, Uzbekistan. A total of 167 were treated, of which 129 were included in this study. Approximately 65% were in the pediatric age group, and the gender distribution varied dramatically for adults and children. Most patients had deep foot burns caused by sandal heaters.

The following methods of treatment were developed: initial surgical debridement of the wound; necrotomy with application of chemotherapeutic medications and early necrectomy; and removal of necrotic tissues and preparation of the wound for early autodermoplasty.

**Results:**

Of all surviving patients, 39.3% came back to the hospital after treatment and burned skin restoration for a new surgery, because they had lost the ability to execute normal movement of their affected extremities. Complications, especially in the fingers and joints, were as follows: contractures, ulcerating scars, and complete and partial dislocations. Of all patients, 89% showed satisfactory results, and 11% had unsatisfactory results. Unsatisfactory results were reported for the children who had irreversible changes in the tissues because more than 5 years had passed since the burn incident.

**Conclusions:**

This effective treatment method contributed to the restoration of lower extremity function in patients; it lessened their joint deformities and post-burn contractures, and also shortened their hospital stay.

**P121**
**The Use of Panthenol and Hyaluronic Acid Creams following Enzymatic Debridement with Nexobrid in Deep Partial-Thickness and Full-Thickness Burns**
**Alexandru-Ionel Tălăban** ^1^, Oliviu Nica ^1,2^, Marius-Eugen Ciurea ^1,2^, Alexandra-Elena Marinescu ^1,2^, Răzvan Mercuț ^1,2^
^1 ^ Emergency County Hospital of Craiova, Craiova, Romania
^2 ^ University of Medicine and Pharmacy of Craiova, Craiova, Romania
**Aim:**

To evaluate the use of hyaluronic acid and panthenol creams in burn wound management following enzymatic debridement with bromelain.

**Methods:**

We conducted a prospective study on the use of less commonly utilized creams containing hyaluronic acid and panthenol, following enzymatic debridement with bromelain (Nexobrid).

This case series included seven adult patients who were admitted for partial-thickness and full-thickness thermal burns and scalds between 10 to 20% TBSA.

**Results:**

Pseudoeschar formation and spontaneous epithelization was monitored in all seven patients. Only four developed pseudoeschars on full-thickness burns, and none of the patients developed them on deep partial-thickness lesions. The full-thickness burns required skin grafting, and secondary healing was observed in the deep partial-thickness burns.

**Conclusions:**

Nexobrid has been shown to promote faster and more complete epithelization compared to surgical debridement of deep partial-thickness burns. The removal of the necrotic tissue reduces the bacterial load in the wound, which further promotes healing. However, pseudoeschars are known to appear in both partial-thickness and full-thickness burns after enzymatic debridement, which delays healing.

The lack of a consensus regarding optimal wound dressings following enzymatic debridement with Nexobrid has prompted us to try previously unexplored options, suh as creams with panthenol and hyaluronic acid.

However, it is important to note that this study had a very small sample size, and additional research is needed to determine whether pseudoeschar prevention and rapid re-epithelization is achievable using these hydrating creams.

**Keywords:**

bromelain; panthenol; hyaluronic acid

**P122**
**Our Clinical Experience in Enzymatic Debridement of Deep Partial-Thickness and Full-Thickness Facial Burns: A Case Presentation**
**Panche Taskov** ^1,2^, Noemi Kristo ^1^, Daniel Pit ^1^, Mihaela Prodan ^1,2^, Zorin Crainiceanu ^1,2^
^1 ^ Plastic and Reconstructive Surgery Clinic—Casa Austria, Emergency County Hospital “Pius Branzeu” Timisoara, Romania, Timisoara, Romania
^2 ^ University of Medicine and Pharmacy “Victor Babes”, Timisoara, Romania
**Aim:**

Our experience over the years with the use of Nexobrid, the effectiveness of the product (as proven by fast, selective, and effective debridement of deep dermal burns), and significant reductions in the need for SOC encouraged us to expand the use of this techniques to facial burns.

Deep facial burns frequently require SOC, but may have results such as unaesthetic scars and post-burn sequelae with functional deficits. The preservation of viable facial layers, especially the viable dermis, is essential for spontaneous epithelization and the absence of unsightly scars. Deep facial burns are often a challenge. Early debridement promises a better aesthetic and functional result.

**Methods:**

We assessed a 25-year-old patient with thermal flame burn injuries of the IIB-III degree and 25% TBSA. We used an internal protocol for EDNX. It should be mentioned that this is the first instance of its off-label use on the face in our unit.

**Results:**

The quality of scars was evaluated using to the Vancouver scale (1-month score: 6, 3-month score: 4, 6-month score: 2).

**Conclusions:**

EDNX is a very good option in the treatment of deep dermal facial burns and an effective alternative to SOC, as demonstrated by the spontaneous epithelization and early reduction of edema, and the preservation of viable tissues. It has proved to be safe also in high-risk patients. Good promising aesthetic results and earlier rehabilitation times are additional reasons to continue using Nexobrid as the first-choice treatment for deep dermal facial burns. The effectiveness of the treatment will be proven by the appearance of scars according to the Vancouver scale, and throughout long-term follow-up with the patient.

**P123**
**Our Clinical Experience in Enzymatic Debridement of Deep Partial-Thickness and Full-Thickness Burns in Combination with HY Tissue Micrografts:**
**A Case Presentation**
**Panche Taskov** ^1,2^, Mihaela Prodan ^1,2^, Fabiana Simion ^1^, Ancuta Pop Coman ^1^, Zorin Crainiceanu ^1,2^
^1 ^ Plastic and Reconstructive Surgery Clinic—Casa Austria, Emergency County Hospital “Pius Branzeu” Timisoara, Romania, Timisoara, Romania,
^2 ^ University of Medicine and Pharmacy “Victor Babes”, Timisoara, Romania
**Aim:**

Covering major burns is a still major challenge, even though several strategies are available so far to deal with this situation.

Our novel concept consists of using EDNX and HTM autologous micrografts, thereby avoiding SOC and cover large BSA.

**Methods:**

Our experience was based on few cases and limited use of EDNX and HTM on up to 5% TBSA, which showed good promising aesthetic and functional results; therefore, there was no need for SOC on the treated lesions, and earlier rehabilitation encouraged us to expand the use of this novel technique up to 25% TBSA.

Our new technique is a regenerative technique that in future may allow us to reduce the need for grafting when skin donor areas are limited, and avoids the use of allografts and xenografts currently unavailable nationally.

Nexobrid has been shown to have unique selectivity for non-viable tissues, while HTM is an innovative technology based on autologous micrografts.

We studied a 39-year-old patient with thermal flame burn injuries if the IIB-III degree, and 45% TBSA.

**Results:**

All areas treated did not require additional surgery and coverage.

In less than 2 weeks, the lesions completely epithelized.

The quality of the scars was evaluated according to the Vancouver scale (1-month score: 6, 3-month score: 4, 6-month score: 2).

The quality of the scars is clearly superior to SOC scar quality.

**Conclusions:**

The need for new techniques and improvement and standardization of current techniques is essential.

The combination of EDNX and HTM is an excellent treatment option for patients with major burns.

This technique will show its efficacy when higher standardization and experience is achieved.

**P124**
**Enzymatic Demarcation—8 Years of Experience from the Dr Stanislaw Sakiel Center for Burn Treatment**
**Karolina Ziółkowska**, Przemysław Strzelec, Wojciech Łabuś, Marcin Gierek, Karolina Mikuś-Zagórska
Dr. Stanisław Sakiel Center For Burn Treatment In Siemianowice Śląskie, Siemianowice Śląskie, Poland
**Aim:**

Enzymatic demarcation has become one of the surgeon’s primary tools in the treatment of burns. In this method, using Nexobrid containing proteolytic enzymes, it was assumed that only the necrotic tissue would be removed, the living tissue would remain intact, and it would then be prepared directly for the next stage of treatment, i.e., covering the wound with a graft or other special dressing. This seems to be the best method of demarcation in burns. In recent years, the Center for Burn Treatment in Siemianowice Śląskie in Poland has, based on its own experience, developed procedures that allow for optimization and speed in the treatment of burns.

**Methods:**

From May 2016 up the present in the Dr Stanislaw Sakiel Center for Burn Treatment in Siemianowice Ślaskie, Poland, enzymatic demarcation has been performed on 128 patients. The surface range oscillated between 1–78% of the total body surface area, and the burns were in te second and third degree, mostly mosaic.

**Results:**

In all analyzed cases, the use of enzymatic demarcation/Nexobrid/was a rational procedure for early demarcation of necrosis, and avoided the need for decompressive incisions. However, secondary necrosis occurred in more than half of cases, despite following the guidelines.

**Conclusions:**

The procedure of enzymatic necrotic tissue debridement has become the golden standard in the therapy of burned patients. However, it should be understood as the first step in the long-term therapy of burned patients, as a number of analyzed patients required traditional surgical demarcation and prolonged hospitalization.

**P130**
**Towards an Increased Quality of Burns Nursing through Tailored Education**
**Marieke Hendriks**, Alette De Jong
Rode Kruis Ziekenhuis, Beverwijk, The Netherlands
**Aim:**

In order to limit staff shortages and to enable carrier development, staff retention. and satisfaction, our aim is to increase the number of nurses that complete the national burns specialization (the first in the international burn field), as well as the ICU specialization, both officially recognized by the College of Dutch Healthcare Studies, through tailored education and associated rewards.

**Methods:**

The national education system for acute care specializations for nurses (such as burn care and intensive care) first requires a basic acute care course consisting of four building blocks (entrustable professional activities (EPAs)). EPAs describe which knowledge, skills, attitudes and levels of supervision are required for a given specialization. Secondly, nurses follow eight burn-specific or eight intensive care-specific EPAs for a recognized diploma per specialization. To facilitate a shorter education process for graduate ICU nurses that aim to become more specialized in burn care, a tailored course of four EPAs has been proposed by and for ICU nurses, leading to a certification. This certification involves an increased salary.

**Results:**

Currently, the first adapted course, consisting of four EPAs and starting this year, is already fully booked, implying that this initiative is widely supported amongst ICU nurses.

**Conclusions:**

This shortened education program corresponds to the needs of nurses and enables a tailored course with an accompanying salary increase, leading to career development, staff retention, and satisfaction.

**P131**
**The Occurrence of Itch after Burns: Is There a Need for Oral Medication?**
**Helma Hofland** ^1,2^
^1 ^ Rotterdam Center for Tropical Medicine, Rotterdam, The Netherlands
^2 ^ Maasstadziekenhuis, Rotterdam, The Netherlands
**Aim:**

This study describes the occurrence and management of itch in adult patients admitted to a burn center in Rotterdam, with follow-up for one year.

**Methods:**

For one month, all adult patients admitted were asked to participate. After the first episode of itching, the Burn Itch Questionnaire (BIQ) was used to record itch frequency until discharge. From the patient’s medical dossier, data on medical history, prescribed medication, and routine usual care were extracted. Follow-up occurred 3, 6, and 12 months after discharge.

**Results:**

In total, 35 patients were admitted (80% male). Most injuries (70%) were induced by flame burns. Some 15 respondents (43%) required an operation. TBSA varied from 0.5% to 31%. The first occurrence of pruritus was seen 3 days after burn injury (range 3–25 days). The score (BIQ) varied from 3 or less in 24 respondents (69%) to more than 8 in 1 respondent (3%). After 3 and 6 months, 40% and 46% of the respondents were without pruritus, respectively; after 12 months, this figure was 60%. Alhydran^®^ based on pure aloe vera was used as a topical agent. After 12 months, 29% still applied this ointment, which was preferred to oral medication. After 12 months, only one patient used antihistamines.

**Conclusions:**

Pruritus is common after burn injury. Although 40% of the respondents still experienced itch after one year, oral medication is often not needed; topical ointments such as Alhydran^®^ are preferred.

